# Can CT Image Reconstruction Parameters Impact the Predictive Value of Radiomics Features in Grading Pancreatic Neuroendocrine Neoplasms?

**DOI:** 10.3390/bioengineering12010080

**Published:** 2025-01-16

**Authors:** Florent Tixier, Felipe Lopez-Ramirez, Alejandra Blanco, Mohammad Yasrab, Ammar A. Javed, Linda C. Chu, Elliot K. Fishman, Satomi Kawamoto

**Affiliations:** 1 The Russell H. Morgan Department of Radiology and Radiological Science, Johns Hopkins Medical Institutions, Baltimore, MD 21287, USA; jlopezr5@jh.edu (F.L.-R.); ablanco5@jhmi.edu (A.B.); myasrab1@jh.edu (M.Y.); lchu1@jhmi.edu (L.C.C.); efishman@jhmi.edu (E.K.F.); skawamo1@jhmi.edu (S.K.); 2 Department of Surgery, The NYU Grossman School of Medicine and NYU Langone Health, New York, NY 10016, USA; ammar.javed@nyulangone.org

**Keywords:** pancreatic neoplasms, pancreatic neuroendocrine tumors, radiomics, reconstruction, contrast-enhanced CT

## Abstract

The WHO grading of pancreatic neuroendocrine neoplasms (PanNENs) is essential in patient management and an independent prognostic factor for patient survival. Radiomics features from CE-CT images hold promise for the outcome and tumor grade prediction. However, variations in reconstruction parameters can impact the predictive value of radiomics. 127 patients with histopathologically confirmed PanNENs underwent CT scans with filtered back projection (B20f) and iterative (I26f) reconstruction kernels. 3190 radiomic features were extracted from tumors and pancreatic volumes. Wilcoxon paired tests assessed the impact of reconstruction kernels and ComBat harmonization efficiency. SVM models were employed to predict tumor grade using the entire set of radiomics features or only those identified as harmonizable. The models’ performance was assessed on an independent dataset of 36 patients. Significant differences, after correction for multiple testing, were observed in 69% of features in the pancreatic volume and 51% in the tumor volume with B20f and I26f kernels. SVM models demonstrated accuracy ranging from 0.67 (95%CI: 0.50–0.81) to 0.83 (95%CI: 0.69–0.94) in distinguishing grade 1 cases from higher grades. Reconstruction kernels alter radiomics features and iterative kernel models trended towards higher performance. ComBat harmonization mitigates kernel impacts but addressing this effect is crucial in studies involving data from different kernels.

## 1. Introduction

Pancreatic neuroendocrine neoplasms (PanNETs) are a heterogeneous group of neoplasms with differing pathological, genetic, and clinical features. The World Health Organization (WHO) classification system for PanNENs is based on the degree of cellular differentiation and cellular proliferation using mitotic index, and Ki-67 proliferation index of the tumor cells. The 2017 WHO classification system describes two categories of PanNENs: well-differentiated pancreatic neuroendocrine tumors (PanNETs) and poorly differentiated pancreatic neuroendocrine carcinoma (PanNECs). PanNETs are well-differentiated tumors with minimal to moderate atypia and lack of necrosis and express intense synaptophysin or chromogranin. A positivity is classified as grade 1, 2, or 3 based on the mitotic index and the Ki-67 index [1]. PanNECs are tumors with high mitotic index and Ki-67 index and are characterized by poorly differentiated tumors consisting of atypical cells with substantial necrosis that are faintly positive for neuroendocrine markers [1]. The tumor grade based on the WHO classification system is an independent prognostic factor for survival in patients with PanNENs [2,3]. Also, the low-grade small PanNETs are indolent tumors with a good prognosis, and patients with small nonfunctioning PanNETs may undergo active surveillance or surgical resection [4]. Therefore, pretreatment prediction of the PanNENs pathological tumor grade is important in determining prognosis and helps to guide the management of patients.

Radiomics analysis has emerged as a valuable tool in constructing prognostic and predictive models in oncology [5], leveraging the capability of radiomic features to capture underlying biological characteristics [6,7]. Machine learning models based on radiomics features have demonstrated valuable clinical applications, supported by a growing body of evidence [8,9,10]. Notably, these models have proven to be effective in applications such as predicting the histological grade of PanNENs in computed tomography (CT) images [11,12,13], offering guidance for follow-up and clinical decision-making. Preoperative tumor grading is essential for the effective clinical management of patients with PanNEN. However, biopsy-based techniques, while commonly used, are not ideal due to their invasive nature and the risk of misclassification due to tumor heterogeneity [14]. Radiomics offers a non-invasive approach to capturing tumor heterogeneity, making it an excellent method for achieving this evaluation. Despite their potential to reduce unnecessary procedures, a limitation hindering widespread clinical adoption lies in the generalizability of the models across diverse scanner configurations and acquisition protocols. Studies have shown the external validity of these models can be impacted by different acquisition parameters [15,16], and the challenge of ensuring the transferability of these models to different imaging characteristics remains.

Aiming to reduce the impact of differences in image characteristics, some pre-processing steps such as harmonizing voxel size or delineating a volume of interest, can be readily addressed prior to radiomics features extraction. However, challenges persist with parameters related to image acquisition and reconstruction settings, as they often vary across institutions, scanner manufacturers, and radiologists. While recent advancements in deep learning can achieve post-reconstruction harmonization for such parameters [17,18], their implementation remains complex, essentially due to time and computational resource requirements associated with training tasks. One compelling approach to reduce the impact of imaging protocol variations is the application of batch harmonizing strategies, such as ComBat. Originally developed to reduce variability in center and batch effects for microarray analysis [19], this method is now widely employed in medical image analysis [20,21,22]. However, the efficacy of such strategies in mitigating the influence of diverse acquisition parameters remains uncertain.

In this study, we assess the impact of two soft tissue reconstruction kernels commonly used in abdominal radiology, filtered back projection kernel (B20f) and iterative reconstruction kernel (I26f) on radiomics features extracted from PanNENs and the whole pancreas and investigate the effectiveness of harmonizing trough ComBat features obtained from one kernel to another. We also evaluate the impact of different reconstruction kernels on the predictive value of radiomics features to distinguish WHO grade 1 from grade 2 or 3 PanNENs.

## 2. Materials and Methods

### 2.1. Patients

**Figure 1:**
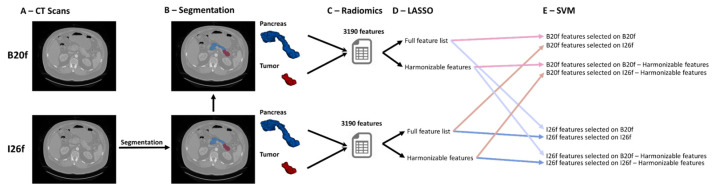
Workflow illustrating the various configurations employed for the SVM in this study. CT scans with B20f and I26f reconstructions were utilized (**A**), and segmentations of the full pancreas and tumor were performed on I26f and transferred to B20f (**B**). Radiomics features were then extracted from both B20f and I26f images of pancreas and tumors (**C**). A LASSO feature selection was applied to choose sets of 10 features from the complete feature list and from features identified as harmonizable by ComBat (**D**). Subsequently, SVM models were constructed on both B20f and I26f images using the different sets of selected features (**E**).

In this retrospective study, we included 127 patients diagnosed with histopathologically confirmed PanNENs from 2012 to 2018. The study was approved by the Institutional Review Board for Human Research and complied with all Health Insurance Portability and Accountability Act regulations. All participants underwent a pancreas protocol CT scan prior to any treatment. The imaging data were acquired using a dual-source multidetector row CT scanner (Siemens Somatom Definition Flash or Siemens Drive), with 100–120 mL of nonionic contrast material (iohexol [Omnipaque 350, GE Healthcare, Princeton, NJ, USA], or Iodixanol [Visipaque 320, GE Healthcare, Princeton, NJ, USA]) administered intravenously through a peripheral venous line at an injection rate of 4–5 mL/sec. Arterial phase acquisition was timed by Bolus Tracking (Siemens Medical Solutions, USA, Inc., Malvern, PA, USA) at 230 HU in the abdominal aorta, followed by a venous phase with 30 s delay. Scan protocols were customized for each patient to minimize radiation dose but were on the order of 120 kVp, effective mAs of 270, and a pitch of 0.6 for the majority of patients (89%). The collimation was 128 × 0.6 mm or 192 × 0.6 mm. All patient images were reconstructed in 0.75 mm slice thickness and 0.5 mm increment using two different kernels in the arterial phase: one filtered back projection kernel (B20f) and one iterative reconstruction kernel (I26f) (Figure 1A). In this study, only the arterial phase is analyzed, because the images were reconstructed using two different kernels only in the arterial phase. PanNENs were classified as grade 1, grade 2, grade 3 PanNET, or PanNEC on the basis of the cellular differentiation and cellular proliferation using mitotic rate and the Ki-67 index on pathological specimens, as described in the classification system of the 2017 World Health Organization (WHO) [23].

### 2.2. Image Segmentation

Images reconstructed with the I26f kernel were segmented by two experienced researchers and reviewed by two experienced radiologists with 7 and 23 years of experience. Manual segmentations were performed to include PanNENs and the entire pancreas uninvolved by PanNENs, as well as structures in the area near the pancreas including pancreatic duct and common bile duct, incidental pancreatic lesions such as cysts (if present), major peripancreatic veins (portal vein, superior mesenteric vein, and splenic vein) and arteries (celiac artery, splenic artery, and superior mesenteric artery) when these vessels are abutting or surrounded by the pancreas or PanNENs.

For clarity, we refer to tumor volume as the volume defined by PanNEN segmentation and as the pancreas volume defined by the volume merging of the pancreatic tissue, pancreatic duct, incidental pancreatic lesions (if present), and PanNENs, and excluding peripancreatic arteries and veins, and the intrapancreatic common bile duct.

Images reconstructed with B20f and I26f kernels shared the same spatial coordinates, allowing the use of identical segmentations for both reconstructions without the need for image registrations (Figure 1B).

### 2.3. Radiomics Features Extraction

Prior to radiomics feature extraction, images, and segmented volumes were resampled to an isotropic voxel of 1 mm^3^ with the c3d tool in ITK-SNAP [24] using a trilinear interpolation for the images and a nearest neighbor interpolation for the segmentation masks.

A total of 3190 radiomics features were extracted from both the tumor volume (1595 features) and the pancreatic volume (1595 features) using the Pyradiomics package [25] (Figure 1C). These features were composed of 28 shape descriptors, and 3162 histogram and texture features resulting from 186 features extracted from original images and images transformed by 16 different convolutional filters (186 ∗ (16 + 1) = 3162 features).

The 186 features comprised of two sets of 93 features each, corresponding to tumor and pancreas volumes. They included 18 histogram features, 24 gray-level co-occurrence matrix (GLCM) features, 14 gray-level differences matrix (GLDM) features, 16 gray-level run-length matrix (GLRLM) features, 16 gray-level size-zone matrix (GLSZM) features, and 5 neighbor gray-tone difference matrix (NGTDM) features. Features extraction was performed following the guidelines outlined by the Image Biomarker Standardization Initiative (IBSI) [26], with a bin width of 25 HU, a 26-connectivity for GLSZM and GLCM, and a distance of 1 mm for GLCM, GLDM, and NGTDM.

The convolutional filtering techniques used were a Wavelets transform (Coiflets 3 wavelets transform with low-pass (L) and high-pass (H) filtering in three spatial coordinates, resulting in eight sub-bands: LLL, LLH, LHL, LHH, HLL, HLH, HHL, and HHH), Laplacian of Gaussian (LoG, with sigma values of 1, 3, 5, and 10 mm), and Gabor filtering (2D axial, kernel size = 8, frequency = 0.5 with four orientations: 0, π/4, π/2, and 3π/4). These convolutional filters are detailed in the Standardized Convolutional Filters for Quantitative Radiomics paper from the Image Biomarker Standardization Initiative (IBSI) [27].

### 2.4. Statistical Analysis and Modeling

The statistical analyses and predictive models were implemented using Python (v3.11.3) with the assistance of the scipy [28] and scikit-learn (sklearn) libraries [29].

Patients were divided into training (70%) and testing (30%) sets using a stratified sampling method to ensure an equivalent proportion of tumor grades in both patient sets. Radiomics features were subsequently extracted from the training set and normalized using minimum and maximum values to a [0–1] scale.

Wilcoxon paired tests were employed to assess the impact of the reconstruction kernels on the radiomic features. To account for the potential increase in Type I errors resulting from multiple hypothesis testing a Bonferroni correction [30] was applied to adjust the *p*-values, ensuring a more stringent control over the overall significance level.

We applied the ComBat algorithm [20,21,22] to harmonize the features extracted on I26f to those extracted on B20f, and vice versa. Subsequently, we assessed the effectiveness of harmonization in mitigating the impact of reconstruction bias on radiomics features using Wilcoxon paired tests. We defined harmonizable features as those for which no statistical differences were identified between features extracted from I26f and B20f reconstructions after harmonization, including features extracted from both tumor and pancreas volumes.

The training set was upsampled to address the imbalance between the proportion of grade 1 cases versus grades 2/3 (Table 1). The Least Absolute Shrinkage and Selection Operator (LASSO) [31] feature selection method was applied to the upsampled I26f and B20f datasets. LASSO was performed with five-fold cross-validation to identify radiomics features pertinent to predicting the grade of PanNENs. The regularization parameter alpha was tuned using a grid search, ranging from 0.01 to 1 in steps of 0.01, with the objective of selecting the minimum alpha value that resulted in the selection of 10 features. LASSO was applied to both the full set of features and the subset of features identified as harmonizable through ComBat (Figure 1D).

Subsequently, radiomics features were extracted from the testing set and normalized using statistics obtained from the training set. It is noteworthy that if features for images in the testing set could extend beyond the observed range in the training set, this leads to normalized features outside of the 0–1 range.

Support Vector Machine (SVM) classifiers with a linear kernel were trained to predict PanNEN grades with the regularization parameter (C) set to 1.0, and five-fold cross-validation to evaluate the generalization performance. The models were retrained using the full training dataset with a probabilistic Platt scaling [32] and ROC curves were generated by varying the decision thresholds to compute AUC and evaluate the models’ performance. Eight models were individually trained on the upsampled I26f and B20f training datasets, employing radiomics features selected by the LASSO on the respective I26f and B20f training datasets, with the full set of features and the subset of features identified as harmonizable by ComBat (Figure 1E). We also investigated four additional models built from features identified as harmonizable (i.e., no significant differences after harmonization) before correction for multiple hypothesis testing. This shorter list of features provides an idea of how models can perform when selecting from only the features with the lowest likelihood of suffering alterations during harmonization. To test the effect of the reconstruction kernel in the feature selection process, we performed the feature selection process using LASSO in the I26b and B20f tests separately and then used these independent lists of features to extract the values from both kernel groups (e.g., the list of features selected on I26f was extracted from both the I26f and B20f datasets and used to train separate models). Different models were compared using evaluation metrics, including accuracy, specificity, sensitivity, precision, and F1 score, computed for both the training and testing sets with 95% confidence intervals (95%CI) computed using a bootstrapping approach with 1000 iterations.

All statistical tests conducted in this study considered *p*-values < 0.05 as indicative of significance.

## 3. Results

### 3.1. Patients

Among 127 patients included, 4 (3.1%) patients were diagnosed with multiple PanNETs, comprising three cases with two PanNETs and one case with three PanNETs. Overall, 61% (81 tumors) had WHO grade 1 PanNETs, 35% (46 tumors) were grade 2, 1% (1 tumor) was grade 3, and 3% (4 tumors) were PanNECs (Table 1). All tumors except two were confirmed by surgical pathology from tumor resection, the remaining tumors (one grade 1 tumor and one grade 2 tumor) were diagnosed by endoscopic ultrasound with fine needle aspiration (FNA). The grade 1 tumor diagnosed by FNA was stable on imaging studies for at least four years. The majority of the tumors were located in the tail (48% or 63 tumors) and 6% (seven patients) had additional cysts. The median tumor volume obtained by manual tumor segmentation was 4.49 cm³, ranging from 0.14 cm³ to 659.00 cm³. Table 1 presents a summary of the demographic and clinical characteristics of all included patients.

### 3.2. Features Altered by Image Reconstruction

The Wilcoxon paired tests revealed significant differences between features extracted from B20f and I26f images in 82% of the features extracted from the pancreatic volume and 77% of those extracted from the tumor volume. After applying the Bonferroni correction, these percentages decreased to 69% and 51% for the pancreas and tumor volume, respectively. Shape features are morphological descriptors and consequently, they were not impacted by the reconstruction kernel (Figure 1A,B).

**Figure 2:**
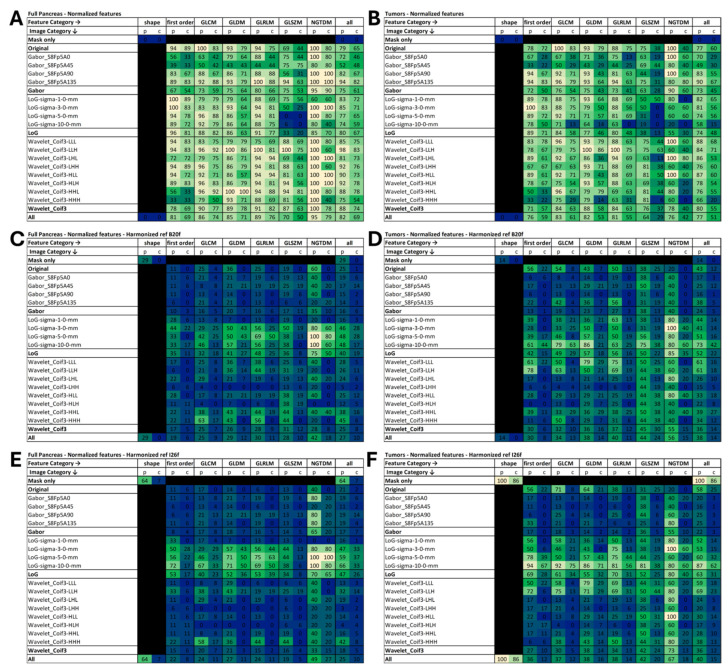
Percentage of features significantly altered by reconstruction on the full pancreas (**A**,**C**,**E**) and on the tumor (**B**,**D**,**F**) volume. Results are shown for images without harmonization (**A**,**B**), images harmonized with B20f as the reference (**C**,**D**), and images harmonized with I26f as the reference (**E**,**F**). Feature categories are presented in columns, and the image convolution used to extract the features is indicated in rows. A blue-to-yellow scale was used to display percentages, with the lower percentages in blue, the middle range in greenish tones, and the highest in yellow.

In the pancreas volume, NGTDM features had the highest number of impacted features, with 95% of the features affected and 79% after Bonferroni correction. On the opposite, GLSZM features showed lower susceptibility, with 70% of the features affected and 50% after Bonferroni correction. Looking at image filtering, wavelet convolutions resulted in the highest percentage of features being affected (88% and 74% after Bonferroni correction). Conversely, Gabor filtering led to the lowest number of impacted features (75% and 61% after the Bonferroni correction). Detailed results are presented in Figure 2A.

In the tumor volume, GLCM features had the highest number of impacted features, with 83% of the features affected and 61% after Bonferroni correction. Similar to results observed in the pancreas volume, GLZSM features exhibited lower susceptibility, with 64% of the features significantly altered and 29% after Bonferroni correction. Analyzing image filtering, original images exhibited the highest percentage of features being affected (77% and 60% after Bonferroni correction). Consistent with results observed in the pancreas volume, Gabor filtering resulted in the lowest number of impacted features (73% and 45% after Bonferroni correction). LoG with a sigma of 10 mm and wavelet filtering with the HHH sub-bands resulted in 58% and 66% of features being impacted, respectively (15% and 20%, respectively, after Bonferroni correction). Detailed results are presented in Figure 2B.

GLSZM features on LoG filtering images with a sigma of 5 mm and 10 mm were found to be unaffected by the kernel reconstruction. Specifically, focusing on the tumor volume, a sigma of 3 mm, and GLRLM and NGTDM features with a sigma of 10 mm were also identified as not significantly altered. (see Figure 2A,B).

ComBat features harmonization resulted in a reduction in the percentage of impacted features, with 27% and 25% of the features found to be impacted in the full pancreas when harmonizing with B20f and I26f as references, respectively. After Bonferroni correction, these percentages dropped to 10% for both reconstruction references. In the tumor volumes, 38% and 40% of the features were found to be impacted with harmonization using B20f and I26f as references, respectively. Following Bonferroni correction, these numbers decreased to 14% and 15%, respectively. ComBat harmonization also influences shape features, leading to features affected by the reconstruction even after Bonferroni correction when using I26f as references (7% and 86% for the pancreas and tumor volumes, respectively). For both pancreas and tumor volume, LoG was identified as the image category for which harmonization had the least impact, particularly for high sigma values, resulting in up to 62% of the features being impacted after harmonization after Bonferroni correction for the tumor volume, with I26f as the reference and LoG with a sigma of 10 mm. Detailed results are presented in Figure 2C–F.

The Wilcoxon results for each individual radiomics feature are available in the Supplementary Table S1. This document also includes a list of features found to be impacted by the reconstruction after Bonferroni correction for any harmonization (tumor volume, pancreas volume, with I26f or with B20f as a reference).

### 3.3. Tumor Grade Prediction Models

#### 3.3.1. Feature Selection

From the 3190 initial features, 1740 (55%) were found to be harmonizable, and 1472 (46%) were found to be harmonizable before accounting for multiple testing corrections.

Tumor grade prediction models were built using the set of 10 features identified by the Least Absolute Shrinkage and Selection Operator (LASSO) on images reconstructed with B20f and I26f kernels using the full set of radiomics features or the subset of features identified as harmonizable (Figure 2D).

**Figure 3:**
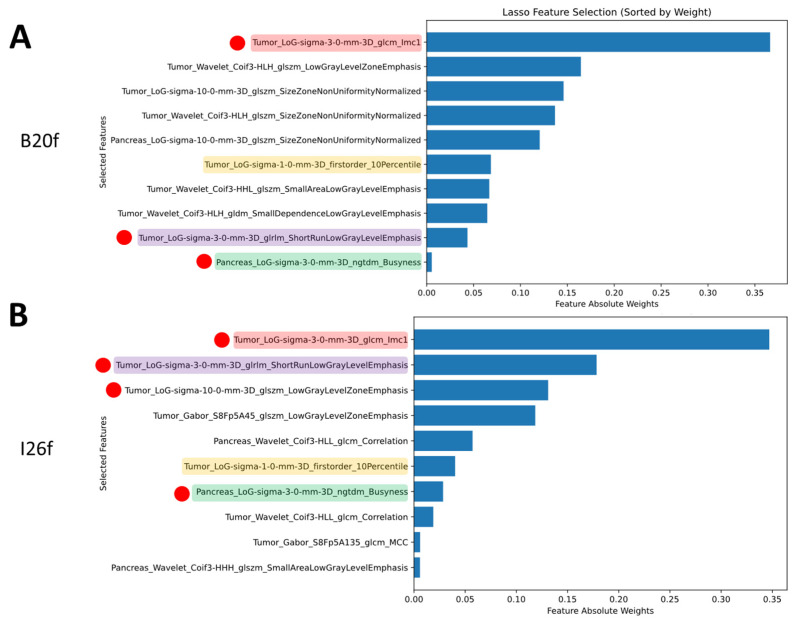
Features selected by LASSO on the entire set of features and their absolute weights on the B20f training cohort (**A**) and the I26f training cohort (**B**). Highlighted are the common features selected on both training datasets. Red dots denote features that were not found to be harmonizable with ComBat harmonization.

With the full set of features on B20f images, the LASSO selected eight features from the tumor volume and two from the pancreas volume. All the selected features were obtained on the images after convolutional filtering (six LoG and four wavelet features). Among the list of selected features, three were found not to be harmonizable with Combat (Figure 3A). On I26f images, the LASSO selected seven features from the tumor volume and three from the pancreas volume. All the selected features were obtained on the images after convolutional filtering (five LoG, three wavelet, and two Gabor features). Among the list of selected features four were found not to be harmonizable with Combat (Figure 3B). Four features were selected in both B20f and I26f images, and three of them were found not to be harmonizable.

**Figure 4:**
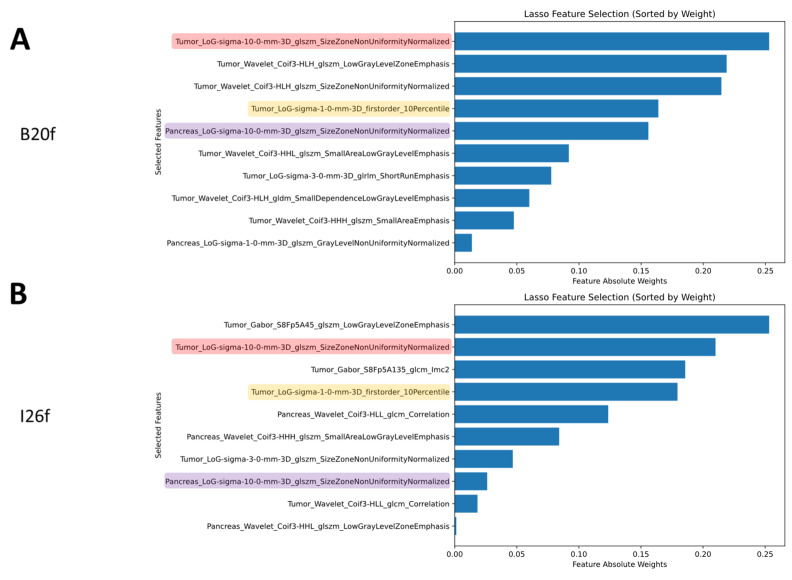
Features selected by LASSO on the features found to be harmonizable and their absolute weights on the B20f training cohort (**A**) and the I26f training cohort (**B**). Highlighted are the common features selected on both training datasets.

Within the subset of harmonizable features, on B20f images, the LASSO selected eight features from the tumor volume and two from the pancreas volume. All the selected features were obtained on the images after convolutional filtering (five LoG and five wavelets features) (Figure 4A). On I26f images, the LASSO selected six features from the tumor volume and four from the pancreas volume. All the selected features were obtained on the images after convolution filtering (four LoG, four wavelet, and two Gabor features) (Figure 4B). Three features were selected in both B20f and I26f images.

The features selected from the subset of harmonizable features identified without correction for multiple testing are similar, in terms of feature categories and associated weights, to the features selected from the subset of harmonizable features identified with Bonferroni correction (Supplementary Figure S1).

#### 3.3.2. SVM Models

Models were found robust by the five-fold cross-validation, exhibiting accuracies slightly lower than those based on the full training dataset (ranging from identical to 0.10 lower performance), with standard deviations between the folds ranging from 0.03 to 0.09 (Supplementary Table S2).

**Figure 5:**
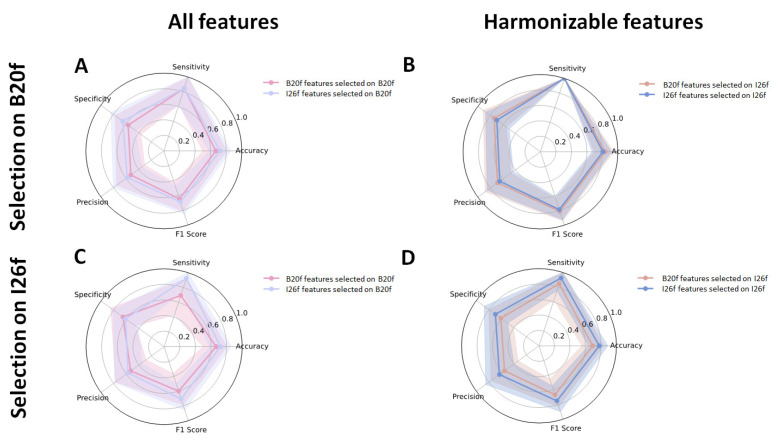
SVM performance evaluation metrics on the testing set for the models using 10 features selected from the entire set of features (**A**,**B**) and the models using only harmonizable features (**C**,**D**). A and C are the models with features selected on B20f and B and D features selected on I26f.

**Figure 6:**
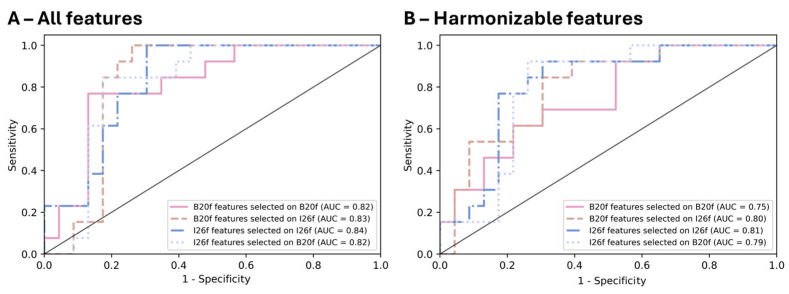
ROC curves generated from probabilistic Platt scaling of SVM models on the testing set. (**A**) Models using features selected from the entire set of features; (**B**) Models using features selected from harmonizable features only.

When focusing on sets of features selected from the full list of radiomics, we found that models built from features selected on I26f led to the highest accuracies: 0.83 (95%CI: 0.69–0.94) and 0.81 (95%CI: 0.67–0.92) for B20f and I26f models respectively, vs. 0.67 (95%CI: 0.50–0.81) and 0.72 (95%CI: 0.58–0.86) for features selected on B20f and models built on B20f and I26f, respectively (Figure 5A,B and Table 2). Models with features selected on I26f led to AUC of 0.83 and 0.84 vs. 0.82 for models with features selected on B20f (Figure 6A).

When employing sets of features selected from the list of harmonizable radiomics features, we observed the highest accuracy of 0.78 (95%CI: 0.64–0.89) for the model constructed on I26f using features selected on I26f. Conversely, the model built on B20f and utilizing features selected on B20f demonstrated the lowest accuracy of 0.67 (95%CI: 0.50–0.81) (Figure 5C,D, and Table 2). Models with features selected on I26f led to AUC of 0.80 and 0.81 vs. 0.75 and 0.79 for models with features selected on B20f (Figure 6B).

Models built from features selected before accounting for multiple testing correction perform slightly lower than models using features from the entire set of harmonizable features with accessories ranging from 0.61 (95% CI 0.44–0.78) to 0.78 (95% CI 0.64–0.89). These results are presented in Supplementary Figures S2 and S3, and Supplementary Table S3.

## 4. Discussion

In this study, we assessed the impact of soft tissue image reconstruction kernels on the radiomics features, explored the possibility of correcting for this effect using ComBat harmonization, and evaluated the predictive value of the radiomic features from images reconstructed with B20f and I26f to distinguish between WHO grade 1 and higher grade PanNENs, including grade 2 or 3 PanNETs and PanNECs. The primary objective was to investigate the reconstruction variability to provide valuable insights to improve the generalizability of PanNENs grading models based on radiomics. However, the results on feature robustness to reconstruction kernel and ComBat harmonization should extend to other radiomic models based on contrast CT features obtained from images reconstructed with iterative or filtered back projection soft tissue reconstruction kernels.

Our results reveal that a substantial proportion of features were biased by the reconstruction kernel, affecting 69% of the features in the pancreas volume and 51% on the tumor volume. This difference can be explained by the nature of radiomics features that seek to characterize heterogeneity. In volumes with low heterogeneity, radiomics features become more sensitive to non-informative factors such as noise or reconstruction parameters, and the features may capture aspects of the image that are not diagnostically relevant. In our study, tumor volumes are more heterogeneous than the entire pancreas, making features extracted from tumor volumes more informative. Heterogeneity is a well-known feature of tumors that can be associated with heterogeneous tumor microenvironments with regional variations in proliferation, metabolic activity, angiogenesis, hypoxia, cell death, and necrosis that are reflected on histopathologic and imaging data [7,33]. Conversely, the effect of reconstruction parameters becomes more noticeable for radiomic features in the pancreas due to its lower heterogeneity.

Erdal B.S. et al. reported 47.12% of features to be stable between iterative (I26f) and filtered back projection (B40f) reconstruction on 28 features extracted from lung nodules of 23 non-contrast-enhanced chest CT scans [34], which is close to the 40% stability we found on the 93 features obtained from original images (no convolution filtering prior to radiomic features extraction) on tumor volumes. Our study presents the advantage of exploring a broader range of radiomics features, including those extracted from filtered images that enhance edges and that were recently introduced in the IBSI guidelines [27]. This notably allowed us to identify that GLSZM features are less impacted than other feature categories, and wavelet filtering with high-pass filters is less impacted than low-pass filters. This can probably be explained by the fact that GLSZM features aim to characterize heterogeneity at a larger scale than other texture categories [35], and high-pass filtering better preserves the edge information of the volume, while low-pass filters blur the image and reduce heterogeneity. Zhao B. et al. assessed the reproducibility of radiomic features across different reconstruction settings, exploring a wider range of images, from sharp to smooth reconstructions [36]. In contrast, our study focuses specifically on kernels commonly used in clinical practice for abdominal soft tissue. Additionally, we have investigated the impact of reconstruction kernels not only on the tumor but also on the entire pancreas, which is particularly relevant given the significant clinical challenge of detecting small lesions in the pancreas [37].

ComBat harmonization proved effective in mitigating the reconstruction effect for many of the features. However, it is noteworthy that such harmonization may introduce variability in shape features, as shown in Figure 2. Consequently, shape features should probably be excluded from the harmonization as variations in image protocols are likely to have a minimal impact on segmentation volumes, and harmonization could potentially introduce more bias. We also identified that ComBat harmonization did not perform well for features obtained from images filtered with LoG, especially for large sigma values. Across all feature categories, up to 15% (for tumor volume and harmonization with I26f as a reference) of the features were found to be statistically different after harmonization. This implies that in studies aiming to build or apply models with images obtained from different reconstructions, caution is needed in selecting features for the model even if using ComBat harmonization. One potential solution could be to remove non-harmonizable features before feature selection, however, this approach may lead to the exclusion of features that are informative, despite the inability of harmonization to correct for the reconstruction effect. This may be the case for features that are more influenced by the classification objective (e.g., grade in our study) than the kernel properties. Furthermore, our study explored harmonization using the standard ComBat method without any covariate, which can increase the risk of reducing the predictive power of the features by smoothing out informative information. While our results could potentially be enhanced by adding tumor grade as a covariate, we chose not to include it, as predicting the grade was the primary objective of the SVM models. Nevertheless, in the context of outcome prediction models, where tumor grade has been histopathologically proven, incorporating grade as a covariate could potentially improve harmonization. Additionally, employing modified ComBat techniques, such as bootstrapped ComBat [22], or more advanced harmonization methods based on deep learning [38,39] could also lead to better results.

In our study, SVM models were evaluated on the testing set using general performance metrics from five-fold cross-validation (Table 2), along with a more comprehensive assessment of model performance across various thresholds (Figure 6). Features selected from the I26f kernel performed better than those selected from the B20f reconstruction images, regardless of the reconstruction that those features would be extracted next. We hypothesize that the filtered back projection kernel (B20f) might induce more noise in the reconstruction than the iterative reconstruction kernel (I26f). This could generate bigger differences between training and testing sets and that could impact the feature selection process, making it harder to validate the models on the testing set. Training models using only harmonizable features seemed to slightly affect the accuracy of the models (up to a 0.14 accuracy reduction in the model built on B20f features from the set selected on I26f). This result highlights the potential trade-off in performance when building models that work with both kernel (harmonizable features) versus models that include non-harmonizable features. Harmonizable features were identified using the Bonferroni correction, which is known to be overly conservative. In the Supplementary Material, we also present models built from features identified as harmonizable before applying multiple hypothesis testing corrections. Although these models showed a slight decrease in accuracy, the impact on performance was not significant and did not alter the interpretation of our findings. As a result, we did not find a need to further refine the selection of harmonizable features. In our experimental design, we chose to investigate some models built from features extracted using a different kernel than the one used for feature selection. This approach ensures that the selected features are not overly dependent on specific image reconstruction parameters. It also allowed us to highlight how a model trained on images from one kernel can be reliably applied to patients imaged with another, using ComBAT harmonization.

Our study is not the only one trying to predict PanNEN grade, Bian Y et al. reported similar results with a sensitivity of 94% and a specificity of 63.5% in identifying patients with grade 2 using radiomics features extracted from contrast-enhanced CT scans from 102 non-functioning PanNET patients [13]. Gu D et al. built a nomogram to differentiate PanNET of grade 1 vs. grade 2 or 3 and from the radiomic features extracted on the arterial phase they reported a model with a sensitivity of 77.8% and a specificity of 81.3% [11]. This study, although conducted on a comparable number of patients, differs from our cohort in crucial aspects, including multicentric data, the exclusion of tumors with a maximum diameter < 10 mm, and a higher proportion of grade 2/3 cases. Zhao Z et al. reported a higher-performing model with a sensitivity of 90.9% and specificity of 88.9% in identifying grade 2 from radiomics features extracted from non-enhanced and contrast-enhanced CT scans [40]. In this study, only non-functional tumors were included, and the authors also investigated CoLIAGe (Cooccurrence of Local Anisotropic Gradient Orientations) features that can capture local entropy patterns [41]. Most of the selected features were obtained in the portal venous phase while in our study features were acquired in the arterial phase only. Also, we have chosen to limit our analysis to features included in the IBSI guidelines and did not incorporate other engineered features, such as CoLIAGe. These differences could explain the variations observed in model performance. Several other studies have investigated tumor grade prediction using other image modalities such as 18F-FDG PET [42], MRI [43] or ultrasound [44]. However, none of these modalities have demonstrated a distinct advantage in predicting the grade compared to contrast-enhanced CT.

Our study has some limitations, including a dataset of only 127 patients (or 132 tumors) that was unbalanced, with a majority of grade 1 cases (61%). We addressed the issue of unbalanced data by upsampling the training datasets used for SVM model training. However, a compromise had to be made between the number of data points in the training set and those available for testing the model. We opted to include only 40 tumors in the testing set (with 13 being grade 2 or 3). Furthermore, this distribution is representative of the patients seen in the clinic making our findings more applicable to real-world scenarios. With this approach, we were able to identify the I26f kernel as more promising than the B20f in grading models, and additional patients would likely narrow the 95% confidence intervals in the results. Additionally, due to limited image availability, another limitation is the fact that we were only able to analyze the effect of two reconstruction kernels from a single vendor (Siemens). However, Dennis M. et al. provide the closest matching kernels for GE, Philips, Siemens, and Toshiba, which can be used to extend our results to kernels from other vendors [45]. Finally, the scan protocol was adjusted for each patient to minimize radiation dose while ensuring consistent image quality. Consequently, patients with higher BMI, who require slightly modified scan protocols, may exhibit differences in radiomic features. Further investigation is needed to quantify this effect. Predictions from the SVM models were made on 40 tumors that were held out from our patient dataset and not used at any stage of training. Although this is a widely accepted standard practice, ideally, model evaluation should be conducted on an independent dataset. However, this was not feasible in our study.

Several other reconstruction techniques exist, including adaptive statistical iterative reconstruction [46] and deep learning CT reconstruction [47]. Each of these methods can produce diverse reconstruction kernels, which contribute to additional image variability, such as differences in smoothing and noise level. Our study provides valuable insights into the impact of two commonly used soft tissue reconstruction kernels in abdominal radiology, one iterative and one filter back projection, where tissue contrast is more critical than sharp edge detection. While smooth kernels may reduce the amount of useful information extracted through radiomics, sharp kernels, on the other hand, introduce noise that can bias radiomics data. Therefore, further investigations are essential to comprehensively explore the effects of different reconstruction kernels in detail.

However, the integration of radiomics into clinical practice necessitates a concerted effort to standardize reconstruction algorithms. This task is particularly challenging given the rapid advancements in scanner technologies, such as photon counting CT, which introduce new complexities for achieving harmonization in radiomics. Nevertheless, these technological shifts also present opportunities to enhance the utility of radiomics [48,49].

Image reconstruction represents one of the numerous challenges for the clinical use of radiomics. To facilitate the translation into clinical practice, it is essential to provide a detailed description of all image processing steps, from data acquisition to modeling, and follow already established guidelines such as those from the IBSI [26,27]. In multicenter studies, various parameters, including CT manufacturer and acquisition settings, can vary and impact radiomic features [15,16]. These additional sources of variability should be considered and must be carefully managed to harmonize images or radiomics features prior to modeling. Furthermore, when interpreting and generalizing radiomics findings across different centers, it is essential to understand precisely how data vary from the datasets used to develop the models.

## 5. Conclusions

In this paper, we explored the influence of two soft tissue reconstruction kernels (I26f and B20f) on radiomics features and their predictive value for determining PanNET grades. Our findings indicate that a substantial number of features are biased by the reconstruction kernel, and I26f showed more promise than B20f for predicting PanNET grades. For studies employing mixed data arising from different reconstruction kernels, it is imperative to address this effect through harmonization techniques, such as ComBat, and by being cautious if using features not identified as harmonizable.

## Figures and Tables

**Table 1:** Patients and tumors’ characteristics.

			Training	Testing	All
Patients			91	36	127
Tumors			96	36	132
Grade					
PanNET		1	58 (60%)	23 (64%)	81 (61 %)
		2	34 (35%)	12 (33%)	46 (35 %)
		3	1 (1%)	0 (0%)	1 (1 %)
PanNEC			3 (3%)	1 (3%)	4 (3%)
Gender					
	Female		52 (57%)	17 (47%)	69 (54%)
	Male		39 (43%)	19 (53%)	58 (46%)
Surgery					
	Yes		95 (99%)	35 (97%)	130 (98%)
	No		1 (1%)	1 (3%)	2 (2%)
Location					
	Head		27 (28%)	12 (33%)	39 (30%)
	Body		17 (18%)	6 (17%)	23 (17%)
	Tail		47 (49%)	16 (44%)	63 (48%)
	Neck		1 (1%)	1 (3%)	2 (2%)
	Uncinate		3 (3%)	1 (3%)	4 (3%)
	Diffuse		1 (1%)	0 (0%)	1 (1%)
Patient with cyst					
	Yes		6 (7%)	1 (3%)	7 (6%)
	No		85 (93%)	35 (97%)	120 (94%)
BMI					
	Underweight (<18.5)		1 (1%)	1 (3%)	2 (2%)
	Healthy Weight [18.5–25]		28 (31%)	9 (25%)	37 (29%)
	Overweight [25–30]		35 (39%)	13 (36%)	48 (38%)
	Obesity [30–40		23 (25%)	12 (33%)	35 (27%)
	Severe Obesity (≥40)		4 (4%)	1 (3%)	5 (4%)
Age					
	Median [range]		61 [23.2–83.4]	59.6 [21.5–82]	61.3 [21.5–83.4]
Functional Type					
	Nonfunctional		18 (19%)	10 (28%)	28 (21%)
	Serotonin		3 (3%)	1 (3%)	4 (3%)
	Insulinoma		6 (6%)	1 (3%)	7 (5%)
	Unknown		69 (72%)	24 (67%)	93 (70%)
Tumor Focality					
	Unifocal		86 (90%)	34 (94%)	120 (91%)
	Multifocal		10 (10%)	2 (6%)	12 (9%)

**Table 2:** SVM performance evaluation metrics. Bolded lines indicate models with the highest accuracies.

LASSO	SVM	Accuracy [95% CI]	Sensitivity [95% CI]	Specificity [95% CI]	Precision [95% CI]	F1 Score [95% CI]
Selection using all features	B20f features selected on B20f	0.67 [0.50–0.81]	0.85 [0.63–1.0]	0.57 [0.36–0.77]	0.52 [0.30–0.75]	0.65 [0.43–0.81]
	I26f features selected on B20f	0.72 [0.58–0.86]	0.85 [0.62–1.0]	0.65 [0.45–0.84]	0.58 [0.35–0.80]	0.69 [0.48–0.85]
	**B20f features selected on I26f**	**0.83 [0.69–0.94]**	**1.0 [1.0–1.0]**	**0.74 [0.55–0.91]**	**0.68 [0.47–0.88]**	**0.81 [0.64–0.94]**
	I26f features selected on I26f	0.81 [0.67–0.92]	1.0 [1.0–1.0]	0.70 [0.50–0.88]	0.65 [0.43–0.85]	0.79 [0.61–0.92]
Selection on harmonizable features	B20f features selected on B20f	0.67 [0.50–0.81]	0.69 [0.43–0.92]	0.65 [0.45–0.84]	0.53 [0.29–0.77]	0.60 [0.36–0.79]
	I26f features selected on B20f	0.72 [0.58–0.86]	0.92 [0.75–1.0]	0.61 [0.41–0.81]	0.57 [0.35–0.78]	0.71 [0.50–0.86]
	B20f features selected on I26f	0.69 [0.56–0.83]	0.85 [0.63–1.0]	0.61 [0.41–0.80]	0.55 [0.33–0.76]	0.67 [0.45–0.83]
	**I26f features selected on I26f**	**0.78 [0.64–0.89]**	**0.92 [0.75–1.0]**	**0.70 [0.50–0.88]**	**0.63 [0.40–0.85]**	**0.75 [0.55–0.90]**

## Data Availability

The datasets analyzed in this study are not publicly available. However, de-identified data may be made available upon reasonable request, subject to IRB approval.

## Supplementary Materials

The following supporting information can be downloaded at: https://www.mdpi.com/article/10.3390/bioengineering12010080/s1, Figure S1: Features selected by LASSO on the features found to be harmonizable before accounting for multiple testing correction and their absolute weights on the B20f training cohort (A) and the I26f training cohort (B); Figure S2: S VM performance evaluation metrics on the testing set for models using 10 features selected from harmonizable features founds before accounting for multiple testing correction. (A) shows the models with features selected on B20f, and (B) shows the models with features selected on I26f; Figure S3: ROC curves generated from probabilistic Platt scaling of SVM models on the testing set using features selected from harmonizable features found before accounting for multiple testing correction; Table S1: Wilcoxon results for each individual radiomics feature. In column D, list of features found to be impacted by the reconstruction kernel after harmonization in at least one of the configurations examined (Tumor—Combat Harmonization—Reference I26f, Pancreas—Combat Harmonization—Reference I26f, Pancreas—Combat Harmonization—Reference B20f, and Tumor—Combat Harmonization—Reference B20f); Table S2: SVM performance on the training set; Table S3: SVM performance on the training and testing sets for models built using features found harmonizable before accounting for multiple testing correction.
