# Supplementary material for: Can CT Image Reconstruction Parameters Impact the Predictive Value of Radiomics Features in Grading Pancreatic Neuroendocrine Neoplasms?
Source: Bioengineering (Basel). 2025 Jan 16;12(1):80. doi: 10.3390/bioengineering12010080 (PMC11763079; doi:10.3390/bioengineering12010080)
Supplement: Supplementary file 1 [file bioengineering-12-00080-s001.zip › Supplementary_Figures_and_Tables/Supplementary_FigureS1.pdf]

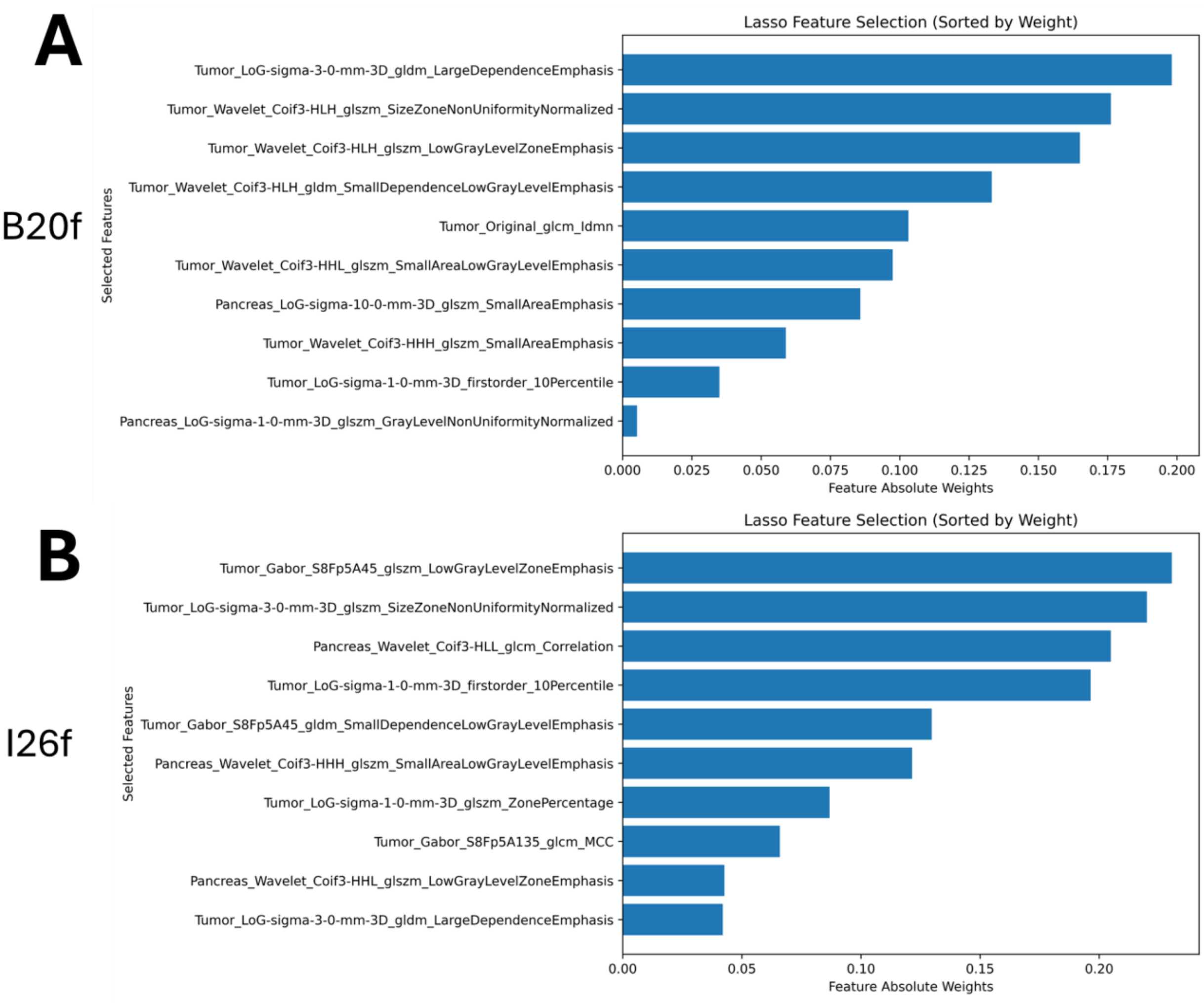

Supplementary Figure S1: Features selected by LASSO on the features found to be harmonizable before accounting for multiple testing correction and their absolute weights on the B20f training cohort (A) and the I26f training cohort (B).
