# Supplementary material for: Can CT Image Reconstruction Parameters Impact the Predictive Value of Radiomics Features in Grading Pancreatic Neuroendocrine Neoplasms?
Source: Bioengineering (Basel). 2025 Jan 16;12(1):80. doi: 10.3390/bioengineering12010080 (PMC11763079; doi:10.3390/bioengineering12010080)
Supplement: Supplementary file 1 [file bioengineering-12-00080-s001.zip › Supplementary_Figures_and_Tables/Supplementary_FigureS2.pdf]

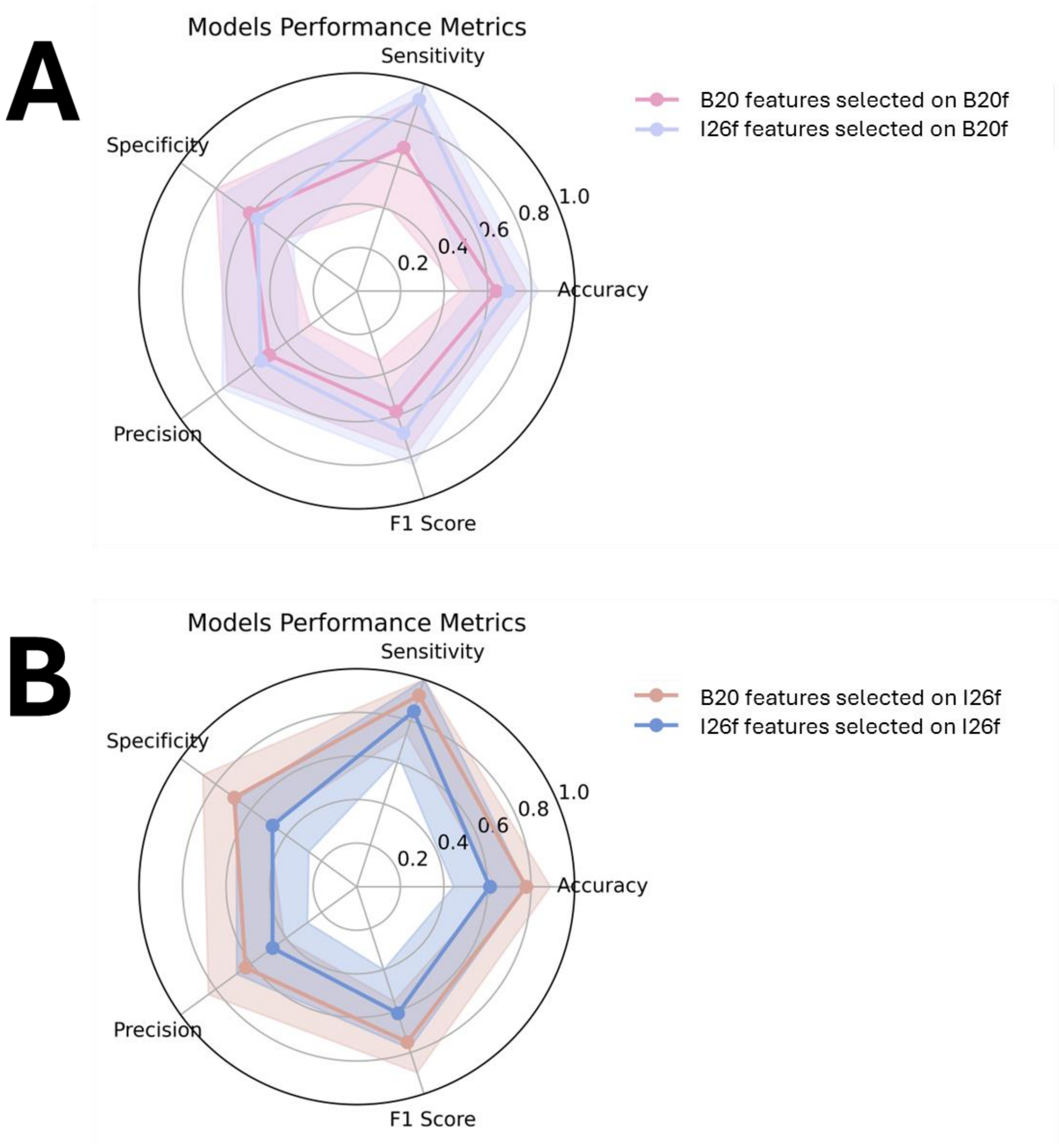

Supplementary Figure S2: SVM performance evaluation metrics on the testing set for models using 10 features selected from harmonizable features founds before accounting for multiple testing correction. (A) shows the models with features selected on B20f, and (B) shows the models with features selected on I26f.
