# Supplementary material for: Can CT Image Reconstruction Parameters Impact the Predictive Value of Radiomics Features in Grading Pancreatic Neuroendocrine Neoplasms?
Source: Bioengineering (Basel). 2025 Jan 16;12(1):80. doi: 10.3390/bioengineering12010080 (PMC11763079; doi:10.3390/bioengineering12010080)
Supplement: Supplementary file 1 [file bioengineering-12-00080-s001.zip › Supplementary_Figures_and_Tables/Supplementary_FigureS3.pdf]

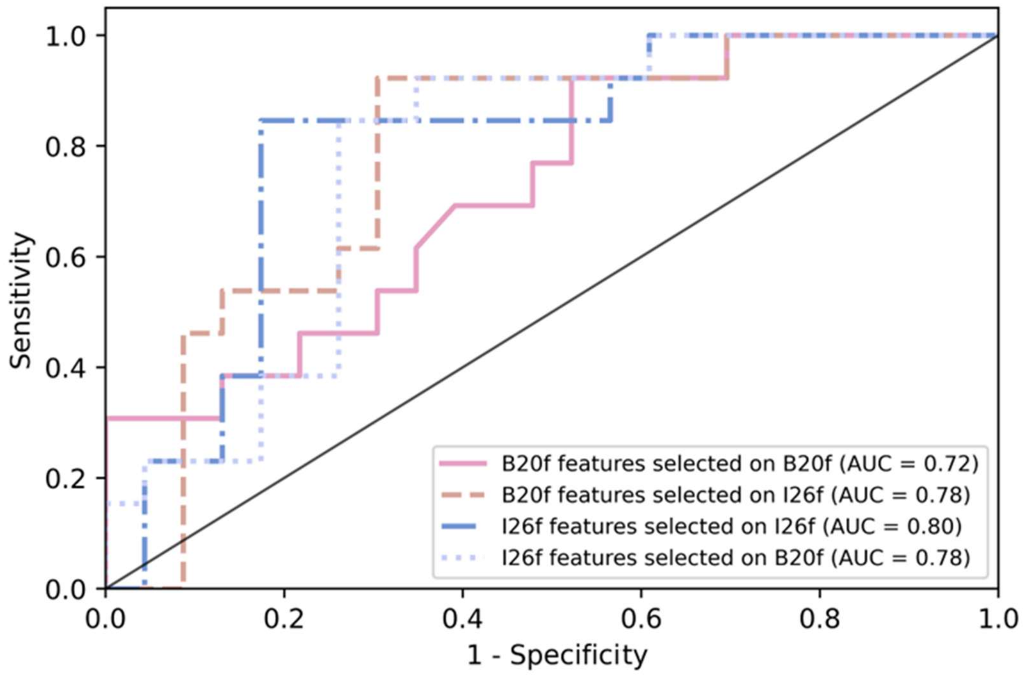

Supplementary Figure S3: ROC curves generated from probabilistic Platt scaling of SVM models on the testing set using features selected from harmonizable features found before accounting for multiple testing correction.
