# Supplementary material for: Can CT Image Reconstruction Parameters Impact the Predictive Value of Radiomics Features in Grading Pancreatic Neuroendocrine Neoplasms?
Source: Bioengineering (Basel). 2025 Jan 16;12(1):80. doi: 10.3390/bioengineering12010080 (PMC11763079; doi:10.3390/bioengineering12010080)
Supplement: Supplementary file 1 [file bioengineering-12-00080-s001.zip › Supplementary_Figures_and_Tables/supplementary_TableS1.pdf]

Image Category	Feature Caterogry	Feature name	Feature Impacted by reconstruction kernel	Pancreas - No Harmonization			Tumor - No Harmonization			Pancreas - Combat Harmonization - Reference B20f			Tumor - Combat Harmonization - Reference B20f			Pancreas - Combat Harmonization - Reference I26f			Tumor - Combat Harmonization - Reference I26f		
				p-value	corrected p-value	z-score	p-value	corrected p-value	z-score	p-value	corrected p-value	z-score	p-value	corrected p-value	z-score	p-value	corrected p-value	z-score	p-value	corrected p-value	z-score
Mask only	Shape	Original_shape_Elongation	TRUE	0.317310508	1	0	0.654720846	1	1	0.449381383	1	2121	0.901120052	1	2294	2.3423E-06	0.00373597	1036	3.45096E-16	5.50428E-13	96
		Original_shape_Flatness	TRUE	0.317310508	1	0	0.179712495	1	0	0.31668807	1	2054	0.00149529	1	1459	0.00043898	0.700173773	1366	4.32851E-07	0.000690398	945
		Original_shape_LeastAxisLength	TRUE	0.317310508	1	0	0.179712495	1	0	0.265034171	1	2023	0.364793447	1	2080	0.733967315	1	2235	4.74159E-14	7.56283E-11	265
		Original_shape_MajorAxisLength	TRUE	0.317310508	1	0	0.179712495	1	0	0.020117175	1	1692	0.924305231	1	2302	0.222257606	1	1994	5.75237E-15	9.17503E-12	191
		Original_shape_Maximum2DDiameterColumn	TRUE	0.317310508	1	0	0.179712495	1	0	0.023247641	1	1707	0.742240365	1	2238	0.080044987	1	1849	5.75237E-15	9.17503E-12	191
		Original_shape_Maximum2DDiameterRow	TRUE	0.317310508	1	0	0.179712495	1	0	0.915601686	1	2299	0.953375076	1	2312	0.000257852	0.411274377	1328	7.83478E-14	1.24965E-10	283
		Original_shape_Maximum2DDiameterSlice	TRUE	0.317310508	1	0	0.179712495	1	0	0.013225555	1	1650	0.334670786	1	2064	0.072187876	1	1836	5.74851E-15	9.16887E-12	191
		Original_shape_Maximum3DDiameter	TRUE	0.317310508	1	0	0.179712495	1	0	0.004122267	1	1543	0.806581231	1	2261	0.003461583	1	1528	5.75237E-15	9.17503E-12	191
		Original_shape_MeshVolume	TRUE	0.317310508	1	0	0.179712495	1	0	0.309674942	1	2050	0.245206692	1	2010	7.56934E-05	0.120730934	1245	3.21285E-15	5.12449E-12	171
		Original_shape_MinorAxisLength	FALSE	0.317310508	1	0	0.654720846	1	1	0.546532102	1	2163	0.806581231	1	2261	0.032238681	1	1742	6.59321E-05	0.105161684	1236
		Original_shape_Sphericity	TRUE	0.317310508	1	0	0.179712495	1	0	0.745002796	1	2239	0.029137327	1	1731	0.00147647	1	1458	7.12231E-13	1.13601E-09	364
		Original_shape_SurfaceArea	TRUE	0.317310508	1	0	0.179712495	1	0	0.12304245	1	1906	0.398587939	1	2097	0.956285864	1	2313	5.58796E-15	8.9128E-12	190
		Original_shape_SurfaceVolumeRatio	FALSE	0.317310508	1	0	0.179712495	1	0	0.666314815	1	2210	0.268183186	1	2025	3.1508E-05	0.050255209	1189	0.029681492	1	1733
		Original_shape_VoxelVolume	TRUE	0.317310508	1	0	0.179712495	1	0	0.302765185	1	2046	0.245206692	1	2010	6.59256E-05	0.105151347	1236	3.21285E-15	5.12449E-12	171
Original	First Order	Original_firstorder_10Percentile	FALSE	7.10126E-16	1.13265E-12	120	2.96619E-16	4.73108E-13	91	0.904013444	1	2295	0.01156559	1	1637	0.442837931	1	2118	0.327404457	1	2060
		Original_firstorder_90Percentile	TRUE	2.28998E-17	3.65252E-14	8	7.46332E-15	1.1904E-11	200	0.157295396	1	1941	3.40688E-06	0.005433974	1057	0.318457508	1	2055	1.54428E-06	0.002463126	1013
		Original_firstorder_Energy	FALSE	7.51076E-13	1.19797E-09	366	2.18788E-10	3.48967E-07	591	0.534444049	1	2158	0.206089528	1	1982	0.190788218	1	1970	0.661011334	1	2208
		Original_firstorder_Entropy	FALSE	1.78053E-17	2.83995E-14	0	3.34814E-16	5.34028E-13	95	0.230675369	1	2000	0.020512828	1	1694	0.33650724	1	2065	0.017534596	1	1678
		Original_firstorder_InterquartileRange	FALSE	2.43835E-17	3.88917E-14	10	3.77846E-16	6.02665E-13	99	0.26818039	1	2025	0.036267099	1	1755	0.415120353	1	2105	0.012694024	1	1646
		Original_firstorder_Kurtosis	FALSE	8.13645E-13	1.29776E-09	369	7.90677E-15	1.26113E-11	202	0.318457508	1	2055	0.019729628	1	1690	0.650449959	1	2204	0.252706887	1	2015
		Original_firstorder_Maximum	TRUE	2.4088E-09	3.84204E-06	695	0.112974522	1	1853	9.22444E-05	0.147129809	1258	0.695788915	1	2221	3.05144E-05	0.048670482	1187	0.701198859	1	2223
		Original_firstorder_MeanAbsoluteDeviation	FALSE	1.78053E-17	2.83995E-14	0	5.42379E-16	8.65094E-13	111	0.593665023	1	2182	0.085218095	1	1857	0.745002796	1	2239	0.030514116	1	1736
		Original_firstorder_Mean	TRUE	0.003670255	1	1533	0.000382329	0.609815128	1356	0.101601052	1	1880	3.3845E-14	5.39828E-11	253	0.901119478	1	2294	6.92756E-12	1.10495E-08	451
		Original_firstorder_Median	TRUE	1.33991E-09	2.13716E-06	669	0.857889624	1	2279	0.909805166	1	2297	7.67305E-12	1.22385E-08	455	0.127544559	1	1911	5.349E-09	8.53165E-06	731
		Original_firstorder_Minimum	FALSE	0.476096548	1	2133	0.436351979	1	2115	0.447194162	1	2120	0.32560271	1	2059	0.869380882	1	2283	0.544106049	1	2162
		Original_firstorder_Range	FALSE	6.6126E-06	0.010547093	1095	0.626950619	1	2195	0.002196209	1	1490	0.912703353	1	2298	0.000967324	1	1425	0.843567126	1	2274
		Original_firstorder_RobustMeanAbsoluteDeviation	FALSE	3.13271E-17	4.99668E-14	18	4.13656E-16	6.59781E-13	102	0.285942489	1	2036	0.036267099	1	1755	0.473837548	1	2132	0.013637986	1	1653
		Original_firstorder_RootMeanSquared	TRUE	4.73999E-14	7.56028E-11	265	2.21585E-11	3.53429E-08	497	0.34204805	1	2068	2.8874E-07	0.00046054	924	0.671636899	1	2212	3.72553E-08	5.94221E-05	822
		Original_firstorder_Skewness	FALSE	2.92726E-13	4.66897E-10	331	7.04152E-08	0.000112312	853	0.390468494	1	2093	0.131239923	1	1915	0.539263037	1	2160	0.138874347	1	1923
		Original_firstorder_TotalEnergy	FALSE	7.51076E-13	1.19797E-09	366	2.18788E-10	3.48967E-07	591	0.532042731	1	2157	0.204781577	1	1981	0.190788218	1	1970	0.663661882	1	2209
		Original_firstorder_Uniformity	FALSE	1.78053E-17	2.83995E-14	0	2.12358E-16	3.3871E-13	80	0.075131659	1	1841	0.005543607	1	1569	0.171713015	1	1954	0.016355601	1	1671
		Original_firstorder_Variance	FALSE	3.77698E-16	6.02428E-13	99	1.81512E-14	2.89512E-11	231	0.904013444	1	2295	0.116100526	1	1898	0.912702845	1	2298	0.012957642	1	1648
	GLCM	Original_glcm_Autocorrelation	FALSE	0.006062098	1	1577	0.001848995	1	1476	0.005994944	1	1576	0.553851692	1	2166	0.003421168	1	1527	0.083900589	1	1855
		Original_glcm_ClusterProminence	TRUE	3.41281E-05	0.0544343	1194	6.23127E-08	9.93888E-05	847	2.13782E-05	0.034098178	1165	4.52806E-16	7.22225E-13	105	0.073353852	1	1838	6.02592E-17	9.61134E-14	39
		Original_glcm_ClusterShade	TRUE	7.71649E-06	0.012307809	1104	0.001268103	1	1446	0.126634033	1	1910	5.11943E-09	8.16548E-06	729	0.031365734	1	1739	2.51017E-11	4.00372E-08	502
		Original_glcm_ClusterTendency	FALSE	4.41526E-17	7.04235E-14	29	1.07868E-15	1.7205E-12	134	0.581082539	1	2177	0.119533081	1	1902	0.492073625	1	2140	0.007806221	1	1600
		Original_glcm_Contrast	FALSE	5.9194E-15	9.44144E-12	192	3.89589E-14	6.21394E-11	258	0.413031558	1	2104	0.036923269	1	1757	0.355202094	1	2075	0.016355601	1	1671
		Original_glcm_Correlation	FALSE	1.28033E-09	2.04213E-06	667	1.03382E-07	0.000164894	872	0.50833395	1	2147	0.145831089	1	1930	0.406802645	1	2101	0.08858513	1	1862
		Original_glcm_DifferenceAverage	FALSE	2.01947E-17	3.22105E-14	4	5.10735E-16	8.14622E-13	109	0.292594211	1	2040	0.015246831	1	1664	0.276157862	1	2030	0.007233074	1	1593
		Original_glcm_DifferenceEntropy	FALSE	7.31693E-16	1.16705E-12	121	5.58796E-15	8.9128E-12	190	0.322015772	1	2057	0.024614402	1	1713	0.410949003	1	2103	0.030514116	1	1736
		Original_glcm_DifferenceVariance	FALSE	4.49665E-07	0.000717215	947	6.23127E-08	9.93888E-05	847	0.46263141	1	2127	0.047223435	1	1785	0.496690774	1	2142	0.035942758	1	1754
		Original_glcm_Id	FALSE	1.78053E-17	2.83995E-14	0	1.61365E-16	2.57377E-13	71	0.181064189	1	1962	0.008602762	1	1609	0.227844631	1	1998	0.011209738	1	1634
		Original_glcm_Idm	FALSE	1.78053E-17	2.83995E-14	0	1.87984E-16	2.99835E-13	76	0.219501177	1	1992	0.008789346	1	1611	0.234968196	1	2003	0.007806221	1	1600
		Original_glcm_Idmn	FALSE	3.19787E-14	5.10061E-11	251	1.01933E-09	1.62583E-06	657	0.331024503	1	2062	0.666316646	1	2210	0.097840858	1	1875	0.287598577	1	2037
		Original_glcm_Idn	FALSE	2.06781E-15	3.29815E-12	156	3.02345E-14	4.8224E-11	249	0.141821526	1	1926	0.223647783	1	1995	0.051882825	1	1796	0.085218095	1	1857
		Original_glcm_Imc1	FALSE	1.5199E-13	2.42424E-10	307	5.94058E-12	9.47522E-09	445	0.38644809	1	2091	0.024848695	1	1714	0.576084726	1	2175	0.049296391	1	1790
		Original_glcm_Imc2	FALSE	4.11924E-11	6.57019E-08	522	3.14897E-09	5.0226E-06	707	0.487479601	1	2138	0.080046751	1	1849	0.362860109	1	2079	0.04641503	1	1783
		Original_glcm_InverseVariance	FALSE	2.88694E-07	0.000460466	924	4.77369E-08	7.61403E-05	834	0.860759099	1	2280	0.501333418	1	2144	0.568626276	1	2172	0.93011237	1	2304
		Original_glcm_JointAverage	FALSE	0.034356859	1	1749	0.015246831	1	1664	0.016031181	1	1669	0.586102738	1	2179	0.002450065	1	1499	0.128462402	1	1912
		Original_glcm_JointEnergy	FALSE	1.78053E-17	2.83995E-14	0	1.18783E-16	1.89459E-13	61	0.019158786	1	1687	0.000538908	0.859558704	1381	0.1239328	1	1907	0.019922939	1	1691
		Original_glcm_JointEntropy	FALSE	1.78053E-17	2.83995E-14	0	3.24834E-16	5.1811E-13	94	0.219501177	1	1992	0.013777781	1	1654	0.274549558	1	2029	0.01156559	1	1637
		Original_glcm_MCC	FALSE	8.97763E-08	0.000143193	865	3.85792E-07	0.000615339	939	0.276157862	1	2030	0.067676407	1	1828	0.198329689	1	1976	0.044059401	1	1777
		Original_glcm_MaximumProbability	FALSE	1.78053E-17	2.83995E-14	0	2.47242E-16	3.94351E-13	85	0.035941675	1	1754	0.000356625	0.568816574	1351	0.178691692	1	1960	0.014495904	1	1659
		Original_glcm_SumAverage	FALSE	0.034356859	1	1749	0.015246831	1	1664	0.016031181	1	1669	0.586102738	1	2179	0.002450065	1	1499	0.128462402	1	1912
		Original_glcm_SumEntropy	FALSE	1.78053E-17	2.83995E-14	0	3.05745E-16	4.87663E-13	92	0.265034171	1	2023	0.016852077	1	1674	0.432054154	1	2113	0.016355601	1	1671
		Original_glcm_SumSquares	FALSE	1.66306E-16	2.65258E-13	72	2.19403E-15	3.49948E-12	158	0.826447574	1	2268	0.068227531	1	1829	0.817918548	1	2265	0.004895674	1	1558
	GLDM	Original_gldm_DependenceEntropy	FALSE	1.73246E-15	2.76327E-12	150	1.60501E-10	2.55999E-07	578	0.025562338	1	1717	0.074536883	1	1840	0.031079295	1	1738	0.045618308	1	1781
		Original_gldm_DependenceNonUniformity	TRUE	1.78053E-17	2.83995E-14	0	1.18783E-16	1.89459E-13	61	0.212717676	1	1987	0.846427649	1	2275	0.055976865	1	1805	7.22103E-10	1.15175E-06	642
		Original_gldm_DependenceNonUniformityNormalized	TRUE	1.78053E-17	2.83995E-14	0	3.77846E-16	6.02665E-13	99	0.445013007	1	2119	0.007076406	1	1591	0.093476316	1	1869	1.69934E-05	0.027104483	1151
		Original_gldm_DependenceVariance	FALSE	1.78053E-17	2.83995E-14	0	1.30236E-16	2.07727E-13	64	0.053670385	1	1800	0.000167257	0.26677482	1298	0.220876309	1	1993	0.019347914	1	1688
		Original_gldm_GrayLevelNonUniformity	TRUE	1.78053E-17	2.83995E-14	0	6.21556E-17	9.91381E-14	40	0.02216013	1	1702	3.00164E-07	0.000478762	926	0.066583553	1	1826	2.3044E-08	3.67551E-05	799
		Original_gldm_GrayLevelVariance	FALSE	4.39194E-16	7.00515E-13	104	2.55178E-14	4.07008E-11	243	0.912702845	1	2298	0.130308955	1	1914	0.895335523	1	2292	0.014349694	1	1658
		Original_gldm_HighGrayLevelEmphasis	FALSE	0.002450065	1	1499	0.000240112	0.3829782	1323	0.006062098	1	1577	0.522494857	1	2153	0.003381182	1	1526	0.073944279	1	1839
		Original_gldm_LargeDependenceEmphasis	FALSE	1.78053E-17	2.83995E-14	0	6.61267E-17	1.05472E-13	42	0.029680538	1	1733	0.001284377	1	1447	0.19453214	1	1973	0.041076227	1	1769
		Original_gldm_LargeDependenceHighGrayLevelEmphasis	FALSE	6.40843E-17	1.02214E-13	41	3.77846E-16	6.02665E-13	99	0.000276839	0.441558746	1333	7.57007E-05	0.120742618	1245	0.445013007	1	2119	0.004519853	1	1551
		Original_gldm_LargeDependenceLowGrayLevelEmphasis	FALSE	9.51047E-14	1.51692E-10	290	2.21464E-14	3.53234E-11	238	0.107057616	1	1887	0.062879272	1	1819	0.679650915	1	2215	0.19079081	1	1970
		Original_gldm_LowGrayLevelEmphasis	FALSE	0.287595761	1	2037	0.277775386	1	2031	0.520118689	1	2152	0.006922791	1	1589	0.480632205	1	2135	0.004677597	1	1554
		Original_gldm_SmallDependenceEmphasis	FALSE	2.8066E-12	4.47653E-09	416	8.94084E-12	1.42606E-08	461	0.614052096	1	2190	0.199610135	1	1977	0.596196407	1	2183	0.123934996	1	1907
		Original_gldm_SmallDependenceHighGrayLevelEmphasis	FALSE	6.75952E-08	0.000107814	851	1.35644E-08	2.16352E-05	774	0.265034171	1	2023	0.661011334	1	2208	0.233531013	1	2002	0.561216583	1	2169
		Original_gldm_SmallDependenceLowGrayLevelEmphasis	FALSE	0.035300421	1	1752	0.027299829	1	1724	0.733967315	1	2235	0.663661882	1	2209	0.764440146	1	2246	0.492076154	1	2140
	GLRLM	Original_glrlm_GrayLevelNonUniformity	TRUE	0.000930214	1	1422	0.795283375	1	2257	0.650449959	1	2204	0.000142299	0.226967057	1287	0.487479601	1	2138	1.67153E-05	0.02666093	1150
		Original_glrlm_GrayLevelNonUniformityNormalized	FALSE	1.78053E-17	2.83995E-14	0	1.93806E-16	3.0912E-13	77	0.419316621	1	2107	0.072189541	1	1836	0.46263141	1	2127	0.056923739	1	1807
		Original_glrlm_GrayLevelVariance	FALSE	1.30734E-11	2.08521E-08	476	5.01631E-11	8.00102E-08	530	0.611486886	1	2189	0.353306456	1	2074	0.546532102	1	2163	0.160540707	1	1944
		Original_glrlm_HighGrayLevelRunEmphasis	FALSE	0.000382299	0.609767035	1356	0.00024357	0.388493523	1324	0.007312227	1	1594	0.581084748	1	2177	0.003585451	1	1531	0.109468172	1	1890
		Original_glrlm_LongRunEmphasis	FALSE	1.78053E-17	2.83995E-14	0	5.49057E-17	8.75747E-14	36	0.002334096	1	1495	8.68271E-05	0.138489282	1254	0.195792016	1	1974	0.05934559	1	1812
		Original_glrlm_LongRunHighGrayLevelEmphasis	TRUE	2.77417E-15	4.4248E-12	166	4.27684E-13	6.82157E-10	345	0.000226716	0.361612524	1319	2.10344E-05	0.033549903	1164	0.097102332	1	1874	0.003845563	1	1537
		Original_glrlm_LongRunLowGrayLevelEmphasis	FALSE	4.47501E-12	7.13765E-09	434	7.29098E-12	1.16291E-08	453	0.246691337	1	2011	0.263473425	1	2022	0.921402696	1	2301	0.539265407	1	2160
		Original_glrlm_LowGrayLevelRunEmphasis	FALSE	0.073942592	1	1839	0.133117321	1	1917	0.529646891	1	2156	0.056449371	1	1806	0.451574659	1	2122	0.063920267	1	1821
		Original_glrlm_RunEntropy	FALSE	9.29124E-16	1.48195E-12	129	6.23062E-13	9.93784E-10	359	0.85215388	1	2277	0.245206692	1	2010	0.892445612	1	2291	0.199610135	1	1977
		Original_glrlm_RunLengthNonUniformity	TRUE	1.78053E-17	2.83995E-14	0	8.73231E-17	1.3928E-13	51	0.880900619	1	2287	0.002169674	1	1489	0.299349052	1	2044	4.98306E-10	7.94799E-07	626
		Original_glrlm_RunLengthNonUniformityNormalized	FALSE	1.78053E-17	2.83995E-14	0	7.48345E-17	1.19361E-13	46	0.189552107	1	1969	0.010527077	1	1628	0.276157862	1	2030	0.016355601	1	1671
		Original_glrlm_RunPercentage	FALSE	1.78053E-17	2.83995E-14	0	6.82051E-17	1.08787E-13	43	0.08389878	1	1855	0.005181184	1	1563	0.218132202	1	1991	0.032239689	1	1742
		Original_glrlm_RunVariance	TRUE	1.78053E-17	2.83995E-14	0	6.41108E-17	1.02257E-13	41	0.000882784	1	1418	1.59068E-05	0.025371402	1147	0.223645078	1	1995	0.063920267	1	1821
		Original_glrlm_ShortRunEmphasis	FALSE	1.78053E-17	2.83995E-14	0	6.41108E-17	1.02257E-13	41	0.1239328	1	1907	0.008419727	1	1607	0.271352173	1	2027	0.032833163	1	1744
		Original_glrlm_ShortRunHighGrayLevelEmphasis	FALSE	1.75609E-05	0.028009607	1153	2.24087E-07	0.000357419	911	0.115252483	1	1897	0.812245491	1	2263	0.086550439	1	1859	0.901120052	1	2294
		Original_glrlm_ShortRunLowGrayLevelEmphasis	FALSE	0.028867544	1	1730	0.017190365	1	1676	0.517750523	1	2151	0.193280825	1	1972	0.455979325	1	2124	0.12938312	1	1913
	GLSZM	Original_glszm_GrayLevelNonUniformity	FALSE	1.5134E-05	0.024138695	1144	1.3537E-08	2.15915E-05	750	0.460408054	1	2126	3.41317E-05	0.05444005	1194	0.432054154	1	2113	3.15113E-05	0.050260563	1189
		Original_glszm_GrayLevelNonUniformityNormalized	FALSE	0.313168582	1	2052	0.448918478	1	2076	0.63996015	1	2200	0.764441481	1	2246	0.64257597	1	2201	0.764441481	1	2246
		Original_glszm_GrayLevelVariance	FALSE	9.96041E-10	1.58868E-06	656	8.17037E-05	0.130317351	1250	0.485191301	1	2137	0.145831089	1	1930	0.388455124	1	2092	0.378486223	1	2087
		Original_glszm_HighGrayLevelZoneEmphasis	FALSE	0.000265296	0.423146599	1330	0.001204893	1	1442	0.049718682	1	1791	0.82360325	1	2267	0.022807198	1	1705	0.72298807	1	2231
		Original_glszm_LargeAreaEmphasis	TRUE	1.66299E-12	2.65247E-09	396	2.4874E-13	3.9674E-10	325	0.661009477	1	2208	1.34726E-05	0.021488873	1137	0.728469951	1	2233	1.67153E-05	0.02666093	1150
		Original_glszm_LargeAreaHighGrayLevelEmphasis	TRUE	5.9389E-12	9.47255E-09	445	1.09549E-11	1.7473E-08	469	0.000463776	0.739723417	1370	1.99198E-07	0.000317721	905	0.000733869	1	1404	5.56416E-06	0.008874832	1085
		Original_glszm_LargeAreaLowGrayLevelEmphasis	TRUE	2.7251E-08	4.34653E-05	807	6.89402E-11	1.0996E-07	543	0.002362607	1	1496	1.10925E-06	0.001769247	995	0.072768953	1	1837	2.65579E-06	0.004235986	1043
		Original_glszm_LowGrayLevelZoneEmphasis	FALSE	0.048042342	1	1787	0.002933622	1	1514	0.360935981	1	2078	0.384450175	1	2090	0.325599883	1	2059	0.440671617	1	2117
		Original_glszm_SizeZoneNonUniformity	FALSE	6.29439E-05	0.100395468	1233	0.00096739	1	1425	0.596196407	1	2183	0.024151552	1	1711	0.551404963	1	2165	0.747771389	1	2240
		Original_glszm_SizeZoneNonUniformityNormalized	FALSE	0.274549558	1	2029	0.820760245	1	2266	0.985422135	1	2323	0.87801893	1	2286	0.962109695	1	2315	0.921403154	1	2301
		Original_glszm_SmallAreaEmphasis	FALSE	0.329209838	1	2061	0.739479817	1	2237	0.988337474	1	2324	0.994168616	1	2326	0.953374804	1	2312	0.944645833	1	2309
		Original_glszm_SmallAreaHighGrayLevelEmphasis	FALSE	0.006479445	1	1583	0.023248447	1	1707	0.94464551	1	2309	0.834998076	1	2271	0.886670109	1	2289	0.682332129	1	2216
		Original_glszm_SmallAreaLowGrayLevelEmphasis	FALSE	0.261913477	1	2021	0.015093962	1	1663	0.626948606	1	2195	0.781221455	1	2252	0.603819901	1	2186	0.834998076	1	2271
		Original_glszm_ZoneEntropy	FALSE	0.189552107	1	1969	0.200893963	1	1978	0.959197507	1	2314	0.904014002	1	2295	0.883784593	1	2288	0.820760245	1	2266
		Original_glszm_ZonePercentage	FALSE	5.74109E-08	9.15703E-05	843	4.29856E-10	6.8562E-07	577	0.539263037	1	2160	0.314927922	1	2053	0.57359345	1	2174	0.202183805	1	1979
		Original_glszm_ZoneVariance	TRUE	1.53674E-12	2.4511E-09	393	2.23047E-13	3.5576E-10	321	0.666314815	1	2210	1.32498E-05	0.021133429	1136	0.728469951	1	2233	1.64416E-05	0.026224301	1149
	NGTDM	Original_ngtdm_Busyness	FALSE	3.25322E-05	0.051888885	1191	0.003302668	1	1524	0.041438317	1	1770	0.020314528	1	1693	0.040001379	1	1766	0.343910701	1	2069
		Original_ngtdm_Coarseness	FALSE	2.42795E-06	0.003872583	1038	0.000230012	0.366869758	1320	0.778416214	1	2251	0.857889624	1	2279	0.473837548	1	2132	0.695788915	1	2221
		Original_ngtdm_Complexity	FALSE	6.9626E-06	0.01110535	1098	2.3044E-08	3.67551E-05	799	0.018421052	1	1683	0.276160667	1	2030	0.008883589	1	1612	0.041439506	1	1770
		Original_ngtdm_Contrast	FALSE	2.29141E-13	3.6548E-10	322	6.73757E-10	1.07464E-06	639	0.647820901	1	2203	0.682332129	1	2216	0.614052096	1	2190	0.440671617	1	2117
		Original_ngtdm_Strength	FALSE	5.07652E-08	8.09705E-05	837	0.01180836	1	1639	0.015556059	1	1666	0.102368728	1	1881	0.188321908	1	1968	0.227847348	1	1998
Laplacian of Gaussian - Sigma 1mm	First Order	LoG-sigma-1-0-mm-3D_firstorder_10Percentile	FALSE	7.97576E-11	1.27213E-07	549	1.57471E-08	2.51166E-05	781	0.544103696	1	2162	0.19203285	1	1971	0.476096548	1	2133	0.085883093	1	1858
		LoG-sigma-1-0-mm-3D_firstorder_90Percentile	FALSE	2.59619E-17	4.14092E-14	12	2.32696E-15	3.71151E-12	160	0.208720952	1	1984	0.030234373	1	1735	0.215412683	1	1989	0.006697982	1	1586
		LoG-sigma-1-0-mm-3D_firstorder_Energy	FALSE	3.0113E-09	4.80302E-06	705	1.63537E-07	0.000260842	895	0.558754064	1	2168	0.000524463	0.83651769	1379	0.629541818	1	2196	0.001059382	1	1432
		LoG-sigma-1-0-mm-3D_firstorder_Entropy	FALSE	1.53674E-12	2.4511E-09	393	5.50017E-12	8.77277E-09	442	0.423537731	1	2109	0.265036963	1	2023	0.419316621	1	2107	0.260365473	1	2020
		LoG-sigma-1-0-mm-3D_firstorder_InterquartileRange	FALSE	1.83751E-17	2.93083E-14	1	1.8385E-15	2.93241E-12	152	0.139849906	1	1924	0.032239689	1	1742	0.112743811	1	1894	0.023248447	1	1707
		LoG-sigma-1-0-mm-3D_firstorder_Kurtosis	FALSE	6.68853E-16	1.06682E-12	118	2.70824E-16	4.31964E-13	88	0.001533301	1	1461	0.002968756	1	1515	0.0023059	1	1494	0.018603804	1	1684
		LoG-sigma-1-0-mm-3D_firstorder_Maximum	FALSE	2.02782E-06	0.003234374	1028	0.59366718	1	2182	0.568626276	1	2172	0.332848451	1	2063	0.380465065	1	2088	0.447196816	1	2120
		LoG-sigma-1-0-mm-3D_firstorder_MeanAbsoluteDeviation	FALSE	3.34682E-16	5.33817E-13	95	3.26377E-13	5.20571E-10	335	0.302765185	1	2046	0.083900589	1	1855	0.313168582	1	2052	0.068227531	1	1829
		LoG-sigma-1-0-mm-3D_firstorder_Mean	FALSE	0.005994944	1	1576	0.852154733	1	2277	0.033434932	1	1746	0.232102801	1	2001	0.01030744	1	1626	0.007473917	1	1596
		LoG-sigma-1-0-mm-3D_firstorder_Median	FALSE	4.73999E-14	7.56028E-11	265	1.09287E-10	1.74312E-07	562	0.085216271	1	1857	0.268183186	1	2025	0.023922148	1	1710	0.001220416	1	1443
		LoG-sigma-1-0-mm-3D_firstorder_Minimum	TRUE	5.49861E-12	8.77029E-09	442	0.002091446	1	1486	2.14068E-06	0.003414382	1031	0.06604497	1	1825	6.79872E-05	0.108439545	1238	0.892446236	1	2291
		LoG-sigma-1-0-mm-3D_firstorder_Range	FALSE	8.81323E-13	1.40571E-09	372	0.040002541	1	1766	0.002602434	1	1504	0.544106049	1	2162	0.000784146	1	1409	0.959197745	1	2314
		LoG-sigma-1-0-mm-3D_firstorder_RobustMeanAbsoluteDeviation	FALSE	1.83751E-17	2.93083E-14	1	8.62099E-15	1.37505E-11	205	0.181064189	1	1962	0.036923269	1	1757	0.164940355	1	1948	0.032239689	1	1742
		LoG-sigma-1-0-mm-3D_firstorder_RootMeanSquared	FALSE	1.06654E-10	1.70114E-07	561	1.60501E-10	2.55999E-07	578	0.489773714	1	2139	0.1110975	1	1892	0.529646891	1	2156	0.105477456	1	1885
		LoG-sigma-1-0-mm-3D_firstorder_Skewness	FALSE	7.68595E-05	0.122590838	1246	1.11693E-09	1.7815E-06	661	0.000794577	1	1410	0.011445874	1	1636	0.010526625	1	1628	0.045618308	1	1781
		LoG-sigma-1-0-mm-3D_firstorder_TotalEnergy	FALSE	3.0113E-09	4.80302E-06	705	1.63537E-07	0.000260842	895	0.558754064	1	2168	0.000517376	0.825214263	1378	0.629541818	1	2196	0.001045762	1	1431
		LoG-sigma-1-0-mm-3D_firstorder_Uniformity	FALSE	3.71693E-15	5.9285E-12	176	3.83911E-13	6.12338E-10	341	0.408872696	1	2102	0.445015666	1	2119	0.266604087	1	2024	0.343910701	1	2069
		LoG-sigma-1-0-mm-3D_firstorder_Variance	FALSE	3.26903E-10	5.2141E-07	608	5.83857E-09	9.31251E-06	735	0.413031558	1	2104	0.084557266	1	1856	0.410949003	1	2103	0.041805502	1	1771
	GLCM	LoG-sigma-1-0-mm-3D_glcm_Autocorrelation	TRUE	0.784027053	1	2253	0.709342184	1	2226	0.50365957	1	2145	1.08356E-05	0.017282715	1124	0.520118689	1	2152	1.46416E-05	0.023353316	1142
		LoG-sigma-1-0-mm-3D_glcm_ClusterProminence	TRUE	0.872258317	1	2284	0.016685134	1	1673	3.57149E-07	0.000569653	935	8.62099E-15	1.37505E-11	205	0.022807198	1	1705	1.81512E-14	2.89512E-11	231
		LoG-sigma-1-0-mm-3D_glcm_ClusterShade	TRUE	0.107057616	1	1887	0.00417036	1	1544	2.63421E-09	4.20156E-06	699	6.57331E-13	1.04844E-09	361	5.2893E-08	8.43643E-05	839	1.0493E-06	0.001673641	992
		LoG-sigma-1-0-mm-3D_glcm_ClusterTendency	FALSE	6.23282E-09	9.94134E-06	738	7.04152E-08	0.000112312	853	0.522492428	1	2153	0.166057422	1	1949	0.48290883	1	2136	0.075133362	1	1841
		LoG-sigma-1-0-mm-3D_glcm_Contrast	FALSE	2.46361E-12	3.92945E-09	411	5.26856E-11	8.40335E-08	532	0.40473886	1	2100	0.063920267	1	1821	0.368670925	1	2082	0.029137327	1	1731
		LoG-sigma-1-0-mm-3D_glcm_Correlation	FALSE	1.87909E-16	2.99714E-13	76	2.88072E-09	4.59474E-06	703	0.421424076	1	2108	0.092058573	1	1867	0.276157862	1	2030	0.032535267	1	1743
		LoG-sigma-1-0-mm-3D_glcm_DifferenceAverage	FALSE	7.76782E-16	1.23897E-12	123	2.77778E-14	4.43056E-11	246	0.263470636	1	2022	0.071612261	1	1835	0.207400881	1	1983	0.049720016	1	1791
		LoG-sigma-1-0-mm-3D_glcm_DifferenceEntropy	FALSE	2.27792E-12	3.63328E-09	408	9.64954E-12	1.5391E-08	464	0.351411621	1	2073	0.154104825	1	1938	0.332845627	1	2063	0.146846314	1	1931
		LoG-sigma-1-0-mm-3D_glcm_DifferenceVariance	FALSE	8.99532E-06	0.014347536	1113	2.38508E-06	0.003804202	1037	0.423537731	1	2109	0.030234373	1	1735	0.485191301	1	2137	0.022374807	1	1703
		LoG-sigma-1-0-mm-3D_glcm_Id	FALSE	3.54973E-17	5.66183E-14	22	2.61707E-15	4.17422E-12	164	0.260362689	1	2020	0.072189541	1	1836	0.146843953	1	1931	0.041076227	1	1769
		LoG-sigma-1-0-mm-3D_glcm_Idm	FALSE	6.40843E-17	1.02214E-13	41	4.69431E-15	7.48743E-12	184	0.26818039	1	2025	0.083248047	1	1854	0.157295396	1	1941	0.04483314	1	1779
		LoG-sigma-1-0-mm-3D_glcm_Idmn	FALSE	1.26247E-10	2.01364E-07	568	3.25357E-05	0.051894394	1191	0.480632205	1	2135	0.714789644	1	2228	0.314925095	1	2053	0.611488968	1	2189
		LoG-sigma-1-0-mm-3D_glcm_Idn	FALSE	2.41979E-13	3.85957E-10	324	2.35596E-09	3.75775E-06	694	0.086550439	1	1859	0.501333418	1	2144	0.038951615	1	1763	0.297653489	1	2043
		LoG-sigma-1-0-mm-3D_glcm_Imc1	FALSE	1.83751E-17	2.93083E-14	1	1.36206E-13	2.17248E-10	303	0.206086878	1	1982	0.018787444	1	1685	0.245203934	1	2010	0.012435164	1	1644
		LoG-sigma-1-0-mm-3D_glcm_Imc2	FALSE	1.95692E-17	3.12129E-14	3	1.04292E-09	1.66346E-06	658	0.192030253	1	1971	0.035620894	1	1753	0.098583866	1	1876	0.011209738	1	1634
		LoG-sigma-1-0-mm-3D_glcm_InverseVariance	FALSE	1.78053E-17	2.83995E-14	0	6.02592E-17	9.61134E-14	39	0.143814428	1	1928	0.026049349	1	1719	0.144818898	1	1929	0.032239689	1	1742
		LoG-sigma-1-0-mm-3D_glcm_JointAverage	TRUE	0.784027053	1	2253	0.284298465	1	2035	0.434198658	1	2114	5.01321E-06	0.007996068	1079	0.436349298	1	2115	6.28024E-06	0.01001699	1092
		LoG-sigma-1-0-mm-3D_glcm_JointEnergy	FALSE	2.59619E-17	4.14092E-14	12	1.9502E-15	3.11057E-12	154	0.240779515	1	2007	0.246694098	1	2011	0.189552107	1	1969	0.285945304	1	2036
		LoG-sigma-1-0-mm-3D_glcm_JointEntropy	FALSE	1.32563E-14	2.11438E-11	220	1.56272E-13	2.49254E-10	308	0.292594211	1	2040	0.164942823	1	1948	0.281021266	1	2033	0.135972309	1	1920
		LoG-sigma-1-0-mm-3D_glcm_MCC	FALSE	3.38064E-11	5.39211E-08	514	1.98942E-10	3.17313E-07	587	0.00500787	1	1560	0.581084748	1	2177	0.001803607	1	1474	0.230678093	1	2000
		LoG-sigma-1-0-mm-3D_glcm_MaximumProbability	FALSE	1.83751E-17	2.93083E-14	1	2.25953E-15	3.60396E-12	159	0.266604087	1	2024	0.135972309	1	1920	0.183459991	1	1964	0.163833866	1	1947
		LoG-sigma-1-0-mm-3D_glcm_SumAverage	TRUE	0.784027053	1	2253	0.284298465	1	2035	0.434198658	1	2114	5.01321E-06	0.007996068	1079	0.436349298	1	2115	6.28024E-06	0.01001699	1092
		LoG-sigma-1-0-mm-3D_glcm_SumEntropy	FALSE	8.13521E-15	1.29757E-11	203	1.00551E-13	1.60378E-10	292	0.340194664	1	2067	0.208723612	1	1984	0.332845627	1	2063	0.211382008	1	1986
		LoG-sigma-1-0-mm-3D_glcm_SumSquares	FALSE	5.46774E-10	8.72105E-07	630	6.95189E-09	1.10883E-05	743	0.487479601	1	2138	0.137901747	1	1922	0.440668947	1	2117	0.052326356	1	1797
	GLDM	LoG-sigma-1-0-mm-3D_gldm_DependenceEntropy	FALSE	0.000377024	0.601353218	1355	0.767230711	1	2247	0.548965864	1	2164	0.994168616	1	2326	0.355202094	1	2075	0.73396881	1	2235
		LoG-sigma-1-0-mm-3D_gldm_DependenceNonUniformity	TRUE	2.47144E-16	3.94194E-13	85	3.50735E-15	5.59422E-12	174	0.761653954	1	2245	0.194534747	1	1973	0.467096035	1	2129	1.8969E-10	3.02556E-07	585
		LoG-sigma-1-0-mm-3D_gldm_DependenceNonUniformityNormalized	FALSE	1.78053E-17	2.83995E-14	0	3.55689E-16	5.67324E-13	97	0.720250106	1	2230	0.419319338	1	2107	0.467096035	1	2129	0.152003684	1	1936
		LoG-sigma-1-0-mm-3D_gldm_DependenceVariance	FALSE	1.78053E-17	2.83995E-14	0	3.34814E-16	5.34028E-13	95	0.320233409	1	2056	0.254225908	1	2016	0.277772578	1	2031	0.515390402	1	2150
		LoG-sigma-1-0-mm-3D_gldm_GrayLevelNonUniformity	TRUE	5.42621E-15	8.65481E-12	189	1.34731E-12	2.14896E-09	388	0.786836709	1	2254	1.03012E-05	0.016430366	1121	0.750540389	1	2241	9.22531E-05	0.147143725	1258
		LoG-sigma-1-0-mm-3D_gldm_GrayLevelVariance	FALSE	6.48962E-08	0.000103509	849	2.94399E-07	0.000469566	925	0.473837548	1	2132	0.156227965	1	1940	0.453773977	1	2123	0.085883093	1	1858
		LoG-sigma-1-0-mm-3D_gldm_HighGrayLevelEmphasis	TRUE	0.64257597	1	2201	0.047881225	1	1747	0.50365957	1	2145	1.08356E-05	0.017282715	1124	0.520118689	1	2152	1.44003E-05	0.022968503	1141
		LoG-sigma-1-0-mm-3D_gldm_LargeDependenceEmphasis	FALSE	1.78053E-17	2.83995E-14	0	3.05745E-16	4.87663E-13	92	0.202181169	1	1979	0.047223435	1	1785	0.144818898	1	1929	0.022807993	1	1705
		LoG-sigma-1-0-mm-3D_gldm_LargeDependenceHighGrayLevelEmphasis	TRUE	1.34077E-11	2.13852E-08	477	9.4074E-12	1.50048E-08	463	0.01030744	1	1626	1.44003E-05	0.022968503	1141	5.14369E-05	0.082041915	1220	0.227847348	1	1998
		LoG-sigma-1-0-mm-3D_gldm_LargeDependenceLowGrayLevelEmphasis	FALSE	0.091353375	1	1866	1.32776E-08	2.11778E-05	773	0.161630372	1	1945	0.947554895	1	2310	0.166054948	1	1949	0.736722621	1	2236
		LoG-sigma-1-0-mm-3D_gldm_LowGrayLevelEmphasis	FALSE	6.10275E-05	0.097338798	1231	0.008329522	1	1606	0.121276744	1	1904	0.149924427	1	1934	0.111095413	1	1892	0.162730537	1	1946
		LoG-sigma-1-0-mm-3D_gldm_SmallDependenceEmphasis	FALSE	1.21178E-12	1.93279E-09	384	2.70775E-10	4.31886E-07	600	0.347646875	1	2071	0.098585834	1	1876	0.345774158	1	2070	0.076946218	1	1844
		LoG-sigma-1-0-mm-3D_gldm_SmallDependenceHighGrayLevelEmphasis	FALSE	0.329209838	1	2061	0.001653155	1	1467	0.742238913	1	2238	0.000503469	0.803033238	1376	0.193278223	1	1972	0.000280814	0.447898	1334
		LoG-sigma-1-0-mm-3D_gldm_SmallDependenceLowGrayLevelEmphasis	FALSE	1.30979E-09	2.08912E-06	668	0.004417384	1	1549	0.601273871	1	2185	0.953375076	1	2312	0.698490298	1	2222	0.606372867	1	2187
	GLRLM	LoG-sigma-1-0-mm-3D_glrlm_GrayLevelNonUniformity	TRUE	3.05624E-16	4.8747E-13	92	1.21996E-13	1.94583E-10	299	0.576084726	1	2175	0.04035776	1	1767	0.930111963	1	2304	4.48676E-09	7.15638E-06	723
		LoG-sigma-1-0-mm-3D_glrlm_GrayLevelNonUniformityNormalized	FALSE	4.19877E-09	6.69704E-06	720	7.97778E-11	1.27246E-07	549	0.770021653	1	2248	0.548968199	1	2164	0.626948606	1	2195	0.427786302	1	2111
		LoG-sigma-1-0-mm-3D_glrlm_GrayLevelVariance	FALSE	0.055044553	1	1803	0.000650888	1	1395	0.676975497	1	2214	0.184669228	1	1965	0.725726477	1	2232	0.18832449	1	1968
		LoG-sigma-1-0-mm-3D_glrlm_HighGrayLevelRunEmphasis	TRUE	0.012693504	1	1646	0.436351979	1	2115	0.50365957	1	2145	1.06546E-05	0.016994013	1123	0.608926434	1	2188	1.44003E-05	0.022968503	1141
		LoG-sigma-1-0-mm-3D_glrlm_LongRunEmphasis	FALSE	1.78053E-17	2.83995E-14	0	1.66373E-16	2.65365E-13	72	0.13123767	1	1915	0.027556048	1	1725	0.152001291	1	1936	0.049296391	1	1790
		LoG-sigma-1-0-mm-3D_glrlm_LongRunHighGrayLevelEmphasis	FALSE	4.14146E-12	6.60563E-09	431	4.14265E-12	6.60752E-09	431	0.002898704	1	1513	0.011209738	1	1634	0.000150925	0.240725406	1291	0.676977277	1	2214
		LoG-sigma-1-0-mm-3D_glrlm_LongRunLowGrayLevelEmphasis	FALSE	0.016851436	1	1674	2.69427E-09	4.29735E-06	700	0.152001291	1	1936	0.855021221	1	2278	0.182259171	1	1963	0.933017355	1	2305
		LoG-sigma-1-0-mm-3D_glrlm_LowGrayLevelRunEmphasis	FALSE	0.000576618	0.919705265	1386	0.104693338	1	1884	0.137899446	1	1922	0.145831089	1	1930	0.116949178	1	1899	0.162730537	1	1946
		LoG-sigma-1-0-mm-3D_glrlm_RunEntropy	FALSE	1.78053E-17	2.83995E-14	0	1.15191E-16	1.8373E-13	60	0.016684498	1	1673	0.054584687	1	1802	0.020712071	1	1695	0.018603804	1	1684
		LoG-sigma-1-0-mm-3D_glrlm_RunLengthNonUniformity	FALSE	2.36302E-17	3.76901E-14	9	2.06858E-15	3.29938E-12	156	0.50365957	1	2145	0.001895314	1	1478	0.272947663	1	2028	0.561216583	1	2169
		LoG-sigma-1-0-mm-3D_glrlm_RunLengthNonUniformityNormalized	FALSE	5.66124E-17	9.02968E-14	37	3.50735E-15	5.59422E-12	174	0.234968196	1	2003	0.046015217	1	1782	0.188321908	1	1968	0.032535267	1	1743
		LoG-sigma-1-0-mm-3D_glrlm_RunPercentage	FALSE	1.95692E-17	3.12129E-14	3	9.86548E-16	1.57354E-12	131	0.223645078	1	1995	0.038952757	1	1763	0.172861744	1	1955	0.021529852	1	1699
		LoG-sigma-1-0-mm-3D_glrlm_RunVariance	FALSE	1.78053E-17	2.83995E-14	0	1.47215E-16	2.34808E-13	68	0.13030671	1	1914	0.02392297	1	1710	0.153049112	1	1937	0.052326356	1	1797
		LoG-sigma-1-0-mm-3D_glrlm_ShortRunEmphasis	FALSE	1.05002E-16	1.67478E-13	57	2.32696E-15	3.71151E-12	160	0.260362689	1	2020	0.045618308	1	1781	0.282655245	1	2034	0.043676778	1	1776
		LoG-sigma-1-0-mm-3D_glrlm_ShortRunHighGrayLevelEmphasis	TRUE	0.478361439	1	2134	7.34183E-05	0.117102255	1243	0.645196229	1	2202	1.03012E-05	0.016430366	1121	0.279393712	1	2032	3.25357E-05	0.051894394	1191
		LoG-sigma-1-0-mm-3D_glrlm_ShortRunLowGrayLevelEmphasis	FALSE	3.36526E-09	5.36758E-06	710	0.000100996	0.161088504	1264	0.245203934	1	2010	0.140835384	1	1925	0.24224803	1	2008	0.148892968	1	1933
	GLSZM	LoG-sigma-1-0-mm-3D_glszm_GrayLevelNonUniformity	TRUE	9.57413E-17	1.52707E-13	54	7.63236E-13	1.21736E-09	349	0.872258317	1	2284	0.043676778	1	1776	0.12304245	1	1906	6.65561E-09	1.06157E-05	741
		LoG-sigma-1-0-mm-3D_glszm_GrayLevelNonUniformityNormalized	FALSE	9.2948E-13	1.48252E-09	374	2.06723E-05	0.032972339	1133	0.225038734	1	1996	0.404741605	1	2100	0.108658557	1	1889	0.331027329	1	2062
		LoG-sigma-1-0-mm-3D_glszm_GrayLevelVariance	FALSE	8.39129E-07	0.001338411	980	0.934916366	1	2258	0.761653954	1	2245	0.345776976	1	2070	0.886670109	1	2289	0.812245491	1	2263
		LoG-sigma-1-0-mm-3D_glszm_HighGrayLevelZoneEmphasis	FALSE	0.033434932	1	1746	0.587865714	1	2134	0.883784593	1	2288	0.000199153	0.317649123	1310	0.271352173	1	2027	0.002480014	1	1500
		LoG-sigma-1-0-mm-3D_glszm_LargeAreaEmphasis	FALSE	4.05684E-15	6.47066E-12	179	1.65088E-13	2.63315E-10	310	0.019158786	1	1687	0.000483261	0.770801791	1373	0.979592079	1	2321	0.01132726	1	1635
		LoG-sigma-1-0-mm-3D_glszm_LargeAreaHighGrayLevelEmphasis	TRUE	8.07247E-12	1.28756E-08	457	3.13933E-11	5.00723E-08	511	0.100083393	1	1878	2.96333E-08	4.72651E-05	811	0.005481384	1	1568	2.50643E-08	3.99775E-05	803
		LoG-sigma-1-0-mm-3D_glszm_LargeAreaLowGrayLevelEmphasis	TRUE	0.001552687	1	1462	3.28889E-08	5.24579E-05	816	0.045617044	1	1781	5.56416E-06	0.008874832	1085	0.239317282	1	2006	8.69579E-06	0.01386978	1111
		LoG-sigma-1-0-mm-3D_glszm_LowGrayLevelZoneEmphasis	FALSE	0.650449959	1	2204	0.499321054	1	2098	0.227844631	1	1998	0.573595689	1	2174	0.185879198	1	1966	0.586102738	1	2179
		LoG-sigma-1-0-mm-3D_glszm_SizeZoneNonUniformity	FALSE	8.67978E-10	1.38443E-06	650	6.95498E-06	0.011093187	1069	0.973763113	1	2319	0.959197745	1	2314	0.563679755	1	2170	8.68271E-05	0.138489282	1254
		LoG-sigma-1-0-mm-3D_glszm_SizeZoneNonUniformityNormalized	FALSE	0.603819901	1	2186	0.480652625	1	2090	0.515387948	1	2150	0.616624097	1	2191	0.50599392	1	2146	0.64519816	1	2202
		LoG-sigma-1-0-mm-3D_glszm_SmallAreaEmphasis	FALSE	0.215412683	1	1989	0.144821246	1	1929	0.536850824	1	2159	0.32560271	1	2059	0.515387948	1	2150	0.415123078	1	2105
		LoG-sigma-1-0-mm-3D_glszm_SmallAreaHighGrayLevelEmphasis	FALSE	0.003981626	1	1540	0.12938312	1	1913	0.193278223	1	1972	0.478364009	1	2134	0.004785265	1	1556	0.109468172	1	1890
		LoG-sigma-1-0-mm-3D_glszm_SmallAreaLowGrayLevelEmphasis	FALSE	0.161630372	1	1945	0.255751271	1	2017	0.629541818	1	2196	0.261916264	1	2021	0.558754064	1	2168	0.295961554	1	2042
		LoG-sigma-1-0-mm-3D_glszm_ZoneEntropy	FALSE	6.27244E-15	1.00045E-11	194	0.804911157	1	2167	0.536850824	1	2159	0.551407288	1	2165	0.962109695	1	2315	0.714789644	1	2228
		LoG-sigma-1-0-mm-3D_glszm_ZonePercentage	FALSE	6.26866E-14	9.99852E-11	275	3.54246E-09	5.65022E-06	539	0.33650724	1	2065	0.169435205	1	1952	0.368670925	1	2082	0.136934407	1	1921
		LoG-sigma-1-0-mm-3D_glszm_ZoneVariance	FALSE	4.17681E-15	6.66201E-12	180	1.21996E-13	1.94583E-10	299	0.019158786	1	1687	0.000503469	0.803033238	1376	0.985422135	1	2323	0.005299541	1	1565
	NGTDM	LoG-sigma-1-0-mm-3D_ngtdm_Busyness	FALSE	2.3423E-06	0.00373597	1036	0.005181184	1	1563	0.676975497	1	2214	0.024382025	1	1712	0.761653954	1	2245	0.001737711	1	1471
		LoG-sigma-1-0-mm-3D_ngtdm_Coarseness	FALSE	0.172861744	1	1955	0.105477456	1	1885	0.71206247	1	2227	0.00988165	1	1622	0.415120353	1	2105	0.003381369	1	1526
		LoG-sigma-1-0-mm-3D_ngtdm_Complexity	FALSE	0.906908687	1	2296	0.020914498	1	1696	0.94173717	1	2308	0.0009425	1	1423	0.875137443	1	2285	0.001032305	1	1430
		LoG-sigma-1-0-mm-3D_ngtdm_Contrast	FALSE	5.21931E-10	8.3248E-07	628	0.000247074	0.39408329	1325	0.494379317	1	2141	0.588619248	1	2180	0.382453054	1	2089	0.568628535	1	2172
		LoG-sigma-1-0-mm-3D_ngtdm_Strength	TRUE	7.95202E-08	0.000126835	859	0.000202054	0.322276633	1311	0.006408141	1	1582	1.63117E-06	0.002601717	1016	0.067674802	1	1828	1.03012E-05	0.016430366	1121
	First Order	LoG-sigma-3-0-mm-3D_firstorder_10Percentile	FALSE	2.77182E-10	4.42105E-07	601	1.8969E-10	3.02556E-07	585	0.143814428	1	1928	0.712064077	1	2227	0.017884186	1	1680	0.006624517	1	1585
		LoG-sigma-3-0-mm-3D_firstorder_90Percentile	TRUE	1.78053E-17	2.83995E-14	0	8.55082E-07	0.001363856	981	0.042173028	1	1772	0.007392848	1	1595	0.007473568	1	1596	1.16598E-07	0.000185973	878
		LoG-sigma-3-0-mm-3D_firstorder_Energy	FALSE	7.86918E-12	1.25513E-08	456	8.10301E-10	1.29243E-06	647	0.918501649	1	2300	0.027556048	1	1725	0.35710697	1	2076	0.037255128	1	1758
		LoG-sigma-3-0-mm-3D_firstorder_Entropy	FALSE	3.898E-17	6.21731E-14	25	1.88223E-13	3.00215E-10	298	0.758870849	1	2244	0.739479817	1	2237	0.761653954	1	2245	0.826448572	1	2268
		LoG-sigma-3-0-mm-3D_firstorder_InterquartileRange	FALSE	2.69399E-15	4.29692E-12	165	1.06681E-10	1.70156E-07	561	0.202181169	1	1979	0.451577303	1	2122	0.406802645	1	2101	0.289258288	1	2038
		LoG-sigma-3-0-mm-3D_firstorder_Kurtosis	TRUE	2.58208E-10	4.11842E-07	598	1.36991E-05	0.021850015	1138	1.05461E-07	0.000168211	873	0.004840309	1	1557	1.1588E-05	0.018482862	1128	0.006198744	1	1579
		LoG-sigma-3-0-mm-3D_firstorder_Maximum	FALSE	0.000169704	0.270678556	1299	2.28517E-07	0.000364485	912	0.00072418	1	1403	0.225041443	1	1996	8.55104E-05	0.136389057	1253	0.027045669	1	1723
		LoG-sigma-3-0-mm-3D_firstorder_MeanAbsoluteDeviation	FALSE	1.30183E-16	2.07642E-13	64	1.14964E-12	1.83368E-09	382	0.731216908	1	2234	0.988337543	1	2324	0.892445612	1	2291	0.81508133	1	2264
		LoG-sigma-3-0-mm-3D_firstorder_Mean	FALSE	0.035941675	1	1754	0.000398573	0.635723176	1359	0.024150725	1	1711	0.111919313	1	1893	0.000226716	0.361612524	1319	0.00100587	1	1428
		LoG-sigma-3-0-mm-3D_firstorder_Median	TRUE	6.6126E-06	0.010547093	1095	4.46757E-05	0.071257762	1211	0.006846787	1	1588	0.408875433	1	2102	2.17297E-05	0.034658818	1166	0.010092764	1	1624
		LoG-sigma-3-0-mm-3D_firstorder_Minimum	TRUE	1.84492E-05	0.029426462	1156	1.17247E-06	0.001870097	998	2.00444E-11	3.19708E-08	493	0.271354974	1	2027	4.10712E-09	6.55085E-06	719	0.148892968	1	1933
		LoG-sigma-3-0-mm-3D_firstorder_Range	TRUE	6.06636E-06	0.009675843	1090	2.2094E-08	3.52399E-05	797	2.03683E-10	3.24874E-07	588	0.938829964	1	2307	4.32728E-11	6.90202E-08	524	0.809412113	1	2262
		LoG-sigma-3-0-mm-3D_firstorder_RobustMeanAbsoluteDeviation	FALSE	1.49439E-15	2.38356E-12	145	3.03531E-12	4.84133E-09	419	0.227844631	1	1998	0.386450864	1	2091	0.451574659	1	2122	0.233533745	1	2002
		LoG-sigma-3-0-mm-3D_firstorder_RootMeanSquared	FALSE	4.71187E-12	7.51543E-09	436	3.54685E-12	5.65723E-09	425	0.668973778	1	2211	0.000470212	0.749988905	1371	0.935922859	1	2306	0.013499446	1	1652
		LoG-sigma-3-0-mm-3D_firstorder_Skewness	TRUE	0.010092326	1	1624	0.029408327	1	1732	1.60824E-08	2.56514E-05	782	0.208723612	1	1984	5.8692E-07	0.000936138	961	0.682332129	1	2216
		LoG-sigma-3-0-mm-3D_firstorder_TotalEnergy	FALSE	7.67091E-12	1.22351E-08	455	8.48449E-10	1.35328E-06	649	0.915601686	1	2299	0.027556048	1	1725	0.359018267	1	2077	0.037255128	1	1758
		LoG-sigma-3-0-mm-3D_firstorder_Uniformity	FALSE	3.2322E-17	5.15536E-14	19	4.43373E-12	7.0718E-09	415	0.756090864	1	2243	0.508336427	1	2147	0.353303643	1	2074	0.687703022	1	2218
		LoG-sigma-3-0-mm-3D_firstorder_Variance	FALSE	1.66306E-16	2.65258E-13	72	7.12231E-13	1.13601E-09	364	0.388455124	1	2092	0.204781577	1	1981	0.116949178	1	1899	0.64782282	1	2203
	GLCM	LoG-sigma-3-0-mm-3D_glcm_Autocorrelation	TRUE	0.436349298	1	2115	0.433508304	1	2069	8.28974E-10	1.32221E-06	648	3.00164E-07	0.000478762	926	7.73653E-10	1.23398E-06	645	0.480634768	1	2135
		LoG-sigma-3-0-mm-3D_glcm_ClusterProminence	TRUE	2.84883E-13	4.54388E-10	330	1.63921E-10	2.61454E-07	558	5.74109E-08	9.15703E-05	843	8.87607E-07	0.001415733	983	2.3423E-06	0.00373597	1036	1.82693E-08	2.91396E-05	788
		LoG-sigma-3-0-mm-3D_glcm_ClusterShade	TRUE	0.608926434	1	2188	0.621534099	1	2147	8.48257E-10	1.35297E-06	649	0.292597031	1	2040	5.11838E-09	8.16381E-06	729	0.009572353	1	1619
		LoG-sigma-3-0-mm-3D_glcm_ClusterTendency	FALSE	1.71465E-16	2.73487E-13	73	2.68682E-11	4.28547E-08	485	0.619196686	1	2192	0.260365473	1	2020	0.234968196	1	2003	0.880901309	1	2287
		LoG-sigma-3-0-mm-3D_glcm_Contrast	FALSE	1.40846E-15	2.2465E-12	143	5.02994E-09	8.02276E-06	705	0.979592079	1	2321	0.720251672	1	2230	0.682330374	1	2216	0.517752969	1	2151
		LoG-sigma-3-0-mm-3D_glcm_Correlation	FALSE	5.86216E-10	9.35015E-07	633	5.31102E-07	0.000847107	929	0.606370763	1	2187	0.295961554	1	2042	0.593665023	1	2182	0.002334234	1	1495
		LoG-sigma-3-0-mm-3D_glcm_DifferenceAverage	FALSE	1.82262E-16	2.90707E-13	75	3.5406E-10	5.64726E-07	590	0.469337277	1	2130	0.30276801	1	2046	0.750540389	1	2241	0.265036963	1	2023
		LoG-sigma-3-0-mm-3D_glcm_DifferenceEntropy	FALSE	7.76782E-16	1.23897E-12	123	4.60016E-09	7.33725E-06	701	0.546532102	1	2163	0.020314528	1	1693	0.959197507	1	2314	0.041439506	1	1770
		LoG-sigma-3-0-mm-3D_glcm_DifferenceVariance	FALSE	6.64619E-15	1.06007E-11	196	3.77695E-08	6.02424E-05	798	0.529646891	1	2156	0.349528846	1	2072	0.260362689	1	2020	0.440671617	1	2117
		LoG-sigma-3-0-mm-3D_glcm_Id	FALSE	1.22436E-16	1.95286E-13	62	9.34436E-11	1.49043E-07	535	0.750540389	1	2241	0.568628535	1	2172	0.284295652	1	2035	0.378486223	1	2087
		LoG-sigma-3-0-mm-3D_glcm_Idm	FALSE	1.42718E-16	2.27636E-13	67	1.59987E-10	2.5518E-07	557	0.758870849	1	2244	0.561216583	1	2169	0.297650666	1	2043	0.378486223	1	2087
		LoG-sigma-3-0-mm-3D_glcm_Idmn	TRUE	1.5105E-07	0.000240924	891	0.000109841	0.175196099	1238	0.000204978	0.326939385	1312	5.19078E-06	0.008279295	1081	1.81484E-05	0.028946758	1155	1.41561E-08	2.25789E-05	776

		LoG-sigma-3-0-mm-3D_glcm_Idn	TRUE	5.74109E-08	9.15703E-05	843	1.55602E-05	0.024818591	1116	1.26007E-05	0.020098094	1133	0.001918871	1	1479	8.08318E-07	0.001289267	978	0.000292988	0.467316067	1337
		LoG-sigma-3-0-mm-3D_glcm_Imc1	TRUE	2.27792E-12	3.63328E-09	408	0.158390157	1	1900	0.583589032	1	2178	3.40688E-06	0.005433974	1057	0.837851369	1	2272	1.77759E-05	0.027555062	1152
		LoG-sigma-3-0-mm-3D_glcm_Imc2	FALSE	9.29124E-16	1.48195E-12	129	1.31671E-10	2.10016E-07	549	0.340194664	1	2067	0.653085267	1	2205	0.938829607	1	2307	0.01651964	1	1672
		LoG-sigma-3-0-mm-3D_glcm_InverseVariance	FALSE	5.32056E-17	8.48629E-14	35	9.92747E-12	1.58343E-08	446	0.390468494	1	2093	0.172864253	1	1955	0.63996015	1	2200	0.236414368	1	2004
		LoG-sigma-3-0-mm-3D_glcm_JointAverage	TRUE	0.566150423	1	2171	0.025692557	1	1679	5.3479E-09	8.5299E-06	731	8.84488E-06	0.014107576	1112	8.28974E-10	1.32221E-06	648	9.22756E-11	1.4718E-07	555
		LoG-sigma-3-0-mm-3D_glcm_JointEnergy	FALSE	1.51729E-16	2.42008E-13	69	1.31735E-11	2.10117E-08	457	0.320233409	1	2056	0.809412113	1	2262	0.139849906	1	1924	0.717518853	1	2229
		LoG-sigma-3-0-mm-3D_glcm_JointEntropy	FALSE	7.03188E-17	1.12159E-13	44	4.20717E-12	6.71044E-09	413	0.458190688	1	2125	0.260365473	1	2020	0.866505176	1	2282	0.440671617	1	2117
		LoG-sigma-3-0-mm-3D_glcm_MCC	FALSE	9.82937E-09	1.56778E-05	759	1.31423E-05	0.020961905	1106	0.189552107	1	1969	0.269765882	1	2026	0.834997126	1	2271	0.004317026	1	1547
		LoG-sigma-3-0-mm-3D_glcm_MaximumProbability	FALSE	2.21918E-17	3.53959E-14	7	7.19742E-10	1.14799E-06	620	0.634741941	1	2198	0.778417474	1	2251	0.197057862	1	1975	0.46486335	1	2128
		LoG-sigma-3-0-mm-3D_glcm_SumAverage	TRUE	0.566150423	1	2171	0.025692557	1	1679	5.3479E-09	8.5299E-06	731	8.84488E-06	0.014107576	1112	8.28974E-10	1.32221E-06	648	9.22756E-11	1.4718E-07	555
		LoG-sigma-3-0-mm-3D_glcm_SumEntropy	FALSE	3.2322E-17	5.15536E-14	19	9.73561E-13	1.55283E-09	358	0.467096035	1	2129	0.370623454	1	2083	0.930111963	1	2304	0.685015585	1	2217
		LoG-sigma-3-0-mm-3D_glcm_SumSquares	FALSE	1.82262E-16	2.90707E-13	75	2.55435E-11	4.07419E-08	483	0.64257597	1	2201	0.311421358	1	2051	0.257280203	1	2018	0.895336131	1	2292
	GLDM	LoG-sigma-3-0-mm-3D_gldm_DependenceEntropy	FALSE	3.03625E-17	4.84282E-14	17	1.07011E-09	1.70683E-06	637	0.220876309	1	1993	0.160540707	1	1944	0.717517273	1	2229	0.32560271	1	2059
		LoG-sigma-3-0-mm-3D_gldm_DependenceNonUniformity	FALSE	5.15788E-17	8.22682E-14	34	2.42768E-07	0.000387215	889	0.009271307	1	1616	0.000254226	0.405489883	1327	0.001991044	1	1482	0.007473917	1	1596
		LoG-sigma-3-0-mm-3D_gldm_DependenceNonUniformityNormalized	FALSE	5.48829E-17	8.75383E-14	36	4.89103E-05	0.078011864	1186	0.313168582	1	2052	0.239320027	1	2006	0.487479601	1	2138	0.19203285	1	1971
Laplacian of Gaussian - Sigma 3mm		LoG-sigma-3-0-mm-3D_gldm_DependenceVariance	FALSE	4.55806E-15	7.27011E-12	183	0.001132724	1	1403	0.611486886	1	2189	0.340197485	1	2067	0.174016209	1	1956	0.764441481	1	2246
		LoG-sigma-3-0-mm-3D_gldm_GrayLevelNonUniformity	FALSE	3.13271E-17	4.99668E-14	18	1.06034E-14	1.69124E-11	197	0.051007892	1	1794	0.003421356	1	1527	0.002864328	1	1512	0.000288876	0.460757417	1336
		LoG-sigma-3-0-mm-3D_gldm_GrayLevelVariance	FALSE	1.87909E-16	2.99714E-13	76	2.48261E-12	3.95977E-09	393	0.353303643	1	2074	0.18832449	1	1968	0.106264183	1	1886	0.64782282	1	2203
		LoG-sigma-3-0-mm-3D_gldm_HighGrayLevelEmphasis	TRUE	0.181064189	1	1962	0.572616238	1	2128	8.10118E-10	1.29214E-06	647	7.9534E-08	0.000126857	859	7.73653E-10	1.23398E-06	645	0.469339873	1	2130
		LoG-sigma-3-0-mm-3D_gldm_LargeDependenceEmphasis	FALSE	4.14869E-17	6.61716E-14	27	1.28399E-11	2.04796E-08	456	0.619196686	1	2192	0.539265407	1	2160	0.239317282	1	2006	0.347649692	1	2071
		LoG-sigma-3-0-mm-3D_gldm_LargeDependenceHighGrayLevelEmphasis	TRUE	5.00724E-09	7.98654E-06	728	1.35801E-07	0.000216602	860	4.67179E-07	0.00074515	949	0.01651964	1	1672	1.25152E-09	1.99617E-06	666	0.003302668	1	1524
		LoG-sigma-3-0-mm-3D_gldm_LargeDependenceLowGrayLevelEmphasis	TRUE	2.12273E-16	3.38575E-13	80	0.693981906	1	2174	2.5166E-06	0.004013984	1040	0.380467848	1	2088	3.24286E-07	0.000517236	930	0.327404457	1	2060
		LoG-sigma-3-0-mm-3D_gldm_LowGrayLevelEmphasis	TRUE	0.002391437	1	1497	2.10182E-05	0.033523965	1134	8.63483E-09	1.37726E-05	753	0.044059401	1	1777	1.50092E-09	2.39397E-06	674	0.04035776	1	1767
		LoG-sigma-3-0-mm-3D_gldm_SmallDependenceEmphasis	FALSE	0.00051034	0.813991712	1377	0.014892622	1	1624	0.421424076	1	2108	0.581084748	1	2177	0.64257597	1	2201	0.374542082	1	2085
		LoG-sigma-3-0-mm-3D_gldm_SmallDependenceHighGrayLevelEmphasis	TRUE	0.00417014	1	1544	0.001224849	1	1409	4.77283E-08	7.61267E-05	834	0.038952757	1	1763	6.65426E-09	1.06136E-05	741	0.01651964	1	1672
		LoG-sigma-3-0-mm-3D_gldm_SmallDependenceLowGrayLevelEmphasis	TRUE	0.017708302	1	1679	0.605890409	1	2141	1.5744E-08	2.51117E-05	781	0.000882845	1	1418	1.94629E-08	3.10434E-05	791	0.000226736	0.361643126	1319
	GLRLM	LoG-sigma-3-0-mm-3D_glrlm_GrayLevelNonUniformity	TRUE	6.62805E-14	1.05717E-10	277	0.022008726	1	1663	0.068780721	1	1830	0.390471262	1	2093	0.142815309	1	1927	4.86832E-10	7.76497E-07	625
		LoG-sigma-3-0-mm-3D_glrlm_GrayLevelNonUniformityNormalized	FALSE	9.29124E-16	1.48195E-12	129	1.27425E-12	2.03242E-09	368	0.02091375	1	1696	0.021948792	1	1701	0.041438317	1	1770	0.06712895	1	1827
		LoG-sigma-3-0-mm-3D_glrlm_GrayLevelVariance	TRUE	1.59933E-11	2.55093E-08	484	1.03091E-10	1.6443E-07	539	0.000190666	0.304112043	1307	0.053220134	1	1799	3.92298E-06	0.006257157	1065	0.653085267	1	2205
		LoG-sigma-3-0-mm-3D_glrlm_HighGrayLevelRunEmphasis	TRUE	0.33650724	1	2065	0.1888468	1	1926	8.67978E-10	1.38443E-06	650	5.76036E-06	0.009187768	1087	7.73653E-10	1.23398E-06	645	0.000805194	1	1411
		LoG-sigma-3-0-mm-3D_glrlm_LongRunEmphasis	FALSE	2.43835E-17	3.88917E-14	10	2.98782E-12	4.76558E-09	400	0.255748494	1	2017	0.072189541	1	1836	0.190788218	1	1970	0.027045669	1	1723
		LoG-sigma-3-0-mm-3D_glrlm_LongRunHighGrayLevelEmphasis	TRUE	6.58204E-10	1.04984E-06	638	1.08892E-08	1.73683E-05	740	5.4746E-05	0.087319916	1224	0.001781464	1	1473	5.10061E-06	0.008135469	1080	0.004468353	1	1550
		LoG-sigma-3-0-mm-3D_glrlm_LongRunLowGrayLevelEmphasis	FALSE	5.48829E-17	8.75383E-14	36	3.27327E-05	0.052208665	1161	0.0030763	1	1518	0.057401419	1	1808	0.000568876	0.907357532	1385	0.04217423	1	1772
		LoG-sigma-3-0-mm-3D_glrlm_LowGrayLevelRunEmphasis	TRUE	0.028867544	1	1730	0.000234653	0.37427189	1289	1.82658E-08	2.9134E-05	788	0.011931431	1	1640	3.36526E-09	5.36758E-06	710	0.01019982	1	1625
		LoG-sigma-3-0-mm-3D_glrlm_RunEntropy	TRUE	6.89227E-11	1.09932E-07	543	0.487608394	1	2093	0.000361604	0.576758323	1352	0.301056715	1	2045	8.54807E-06	0.013634167	1110	0.000439014	0.700227932	1366
		LoG-sigma-3-0-mm-3D_glrlm_RunLengthNonUniformity	FALSE	3.68155E-14	5.87208E-11	256	2.47203E-10	3.94288E-07	575	0.840707747	1	2273	0.327404457	1	2060	0.614052096	1	2190	9.22531E-05	0.147143725	1258
		LoG-sigma-3-0-mm-3D_glrlm_RunLengthNonUniformityNormalized	FALSE	5.5953E-13	8.9245E-10	355	8.28257E-10	1.32107E-06	626	0.634741941	1	2198	0.442840596	1	2118	0.901119478	1	2294	0.402684098	1	2099
		LoG-sigma-3-0-mm-3D_glrlm_RunPercentage	FALSE	8.20648E-17	1.30893E-13	49	1.15862E-11	1.84801E-08	452	0.304482936	1	2047	0.181066738	1	1962	0.645196229	1	2202	0.18832449	1	1968
		LoG-sigma-3-0-mm-3D_glrlm_RunVariance	FALSE	1.95692E-17	3.12129E-14	3	5.78744E-14	9.23097E-11	256	0.252704115	1	2015	0.038265979	1	1761	0.212717676	1	1987	0.013637986	1	1653
		LoG-sigma-3-0-mm-3D_glrlm_ShortRunEmphasis	FALSE	1.11839E-08	1.78384E-05	765	5.98279E-08	9.54255E-05	820	0.674304148	1	2213	0.014349694	1	1658	0.747769965	1	2240	0.041076227	1	1769
		LoG-sigma-3-0-mm-3D_glrlm_ShortRunHighGrayLevelEmphasis	TRUE	4.23113E-10	6.74865E-07	619	0.003744	1	1499	7.73653E-10	1.23398E-06	645	0.00149529	1	1459	7.73653E-10	1.23398E-06	645	2.35738E-05	0.037600274	1171
		LoG-sigma-3-0-mm-3D_glrlm_ShortRunLowGrayLevelEmphasis	TRUE	0.000815828	1	1412	0.000180203	0.287423213	1271	6.73603E-10	1.0744E-06	639	6.90474E-05	0.110130672	1239	1.98894E-10	3.17236E-07	587	3.35916E-05	0.053578601	1193
	GLSZM	LoG-sigma-3-0-mm-3D_glszm_GrayLevelNonUniformity	FALSE	0.230675369	1	2000	0.397318058	1	973	0.046013946	1	1782	0.576085681	1	2175	0.057399962	1	1808	0.029956288	1	1734
		LoG-sigma-3-0-mm-3D_glszm_GrayLevelNonUniformityNormalized	FALSE	0.001189472	1	1441	0.99235469	1	1104	0.113575208	1	1895	0.061851246	1	1817	0.138872039	1	1923	0.02704503	1	1723
		LoG-sigma-3-0-mm-3D_glszm_GrayLevelVariance	FALSE	1.69915E-05	0.027101409	1151	0.452893694	1	988	0.003543721	1	1530	0.206089149	1	1982	0.002223174	1	1491	0.49669293	1	2142
		LoG-sigma-3-0-mm-3D_glszm_HighGrayLevelZoneEmphasis	TRUE	0.616297903	1	2145	0.221209778	1	914	1.95287E-07	0.000311482	904	0.566152368	1	2171	2.78272E-08	4.43844E-05	808	0.415122689	1	2105
		LoG-sigma-3-0-mm-3D_glszm_LargeAreaEmphasis	TRUE	3.64097E-07	0.000580735	936	0.000317601	0.506574237	1310	0.00071461	1	1402	1.78864E-08	2.85287E-05	787	7.56934E-05	0.120730934	1245	2.40932E-09	3.84286E-06	695
		LoG-sigma-3-0-mm-3D_glszm_LargeAreaHighGrayLevelEmphasis	TRUE	0.000162418	0.259057196	1296	0.000588941	1	1572	0.030513145	1	1736	3.94521E-10	6.29261E-07	616	7.56934E-05	0.120730934	1245	2.25641E-08	3.59898E-05	798
		LoG-sigma-3-0-mm-3D_glszm_LargeAreaLowGrayLevelEmphasis	TRUE	1.34064E-07	0.000213833	885	0.00196355	1	1446	2.86159E-05	0.045642374	1183	0.927208316	1	2303	0.000848693	1	1415	0.529649296	1	2156
		LoG-sigma-3-0-mm-3D_glszm_LowGrayLevelZoneEmphasis	TRUE	0.147864568	1	1932	0.058216238	1	809	2.85201E-06	0.00454896	1047	0.895336044	1	2292	2.9033E-06	0.004630758	1048	0.690394164	1	2219
		LoG-sigma-3-0-mm-3D_glszm_SizeZoneNonUniformity	FALSE	0.192030253	1	1971	0.222575672	1	1003.5	0.728469951	1	2233	0.004879416	1	1558	0.812244415	1	2263	0.002470601	1	1500
		LoG-sigma-3-0-mm-3D_glszm_SizeZoneNonUniformityNormalized	FALSE	0.042173028	1	1772	0.006054839	1	878	0.959197507	1	2314	0.815079511	1	2264	0.988337474	1	2324	0.912702483	1	2298
		LoG-sigma-3-0-mm-3D_glszm_SmallAreaEmphasis	FALSE	0.175176423	1	1957	0.028794747	1	1691	0.747769965	1	2240	0.690394409	1	2219	0.79246432	1	2256	0.770022959	1	2248
		LoG-sigma-3-0-mm-3D_glszm_SmallAreaHighGrayLevelEmphasis	FALSE	0.745002796	1	2239	0.940821656	1	2260	0.141821526	1	1926	0.843567126	1	2274	0.102366723	1	1881	0.98542222	1	2323
		LoG-sigma-3-0-mm-3D_glszm_SmallAreaLowGrayLevelEmphasis	FALSE	0.775615099	1	2250	0.027465009	1	1686	0.329209838	1	2061	0.687703022	1	2218	0.345774158	1	2070	0.82360325	1	2267
		LoG-sigma-3-0-mm-3D_glszm_ZoneEntropy	FALSE	0.000753609	1	1406	0.046039055	1	846.5	0.803751758	1	2260	0.837850435	1	2272	0.668973778	1	2211	0.956285609	1	2313
		LoG-sigma-3-0-mm-3D_glszm_ZonePercentage	FALSE	0.134965362	1	1676	0.194685113	1	688	0.725726477	1	2232	0.020512828	1	1694	0.624359996	1	2194	0.056923739	1	1807
		LoG-sigma-3-0-mm-3D_glszm_ZoneVariance	TRUE	3.24286E-07	0.000517236	930	0.000256	0.408320187	1295	0.000763663	1	1407	4.79294E-09	7.64475E-06	726	7.92428E-05	0.126392221	1248	5.469E-10	8.72306E-07	630
	NGTDM	LoG-sigma-3-0-mm-3D_ngtdm_Busyness	TRUE	8.79823E-08	0.000140332	864	1.63647E-05	0.026101723	1119	1.96816E-09	3.13921E-06	686	5.83857E-09	9.31251E-06	735	1.60642E-09	2.56225E-06	677	6.82746E-07	0.00108898	969
		LoG-sigma-3-0-mm-3D_ngtdm_Coarseness	TRUE	6.09819E-09	9.72662E-06	737	0.637350729	1	2153	0.018421052	1	1683	0.04444485	1	1778	1.02621E-08	1.63681E-05	761	0.011445874	1	1636
		LoG-sigma-3-0-mm-3D_ngtdm_Complexity	TRUE	1.60568E-13	2.56107E-10	309	4.65899E-08	7.43109E-05	808	9.16058E-08	0.000146111	866	0.001918871	1	1479	3.49997E-09	5.58246E-06	819	5.73649E-05	0.091496971	1227
		LoG-sigma-3-0-mm-3D_ngtdm_Contrast	TRUE	2.78272E-08	4.43844E-05	808	1.65118E-06	0.002633636	989	0.480632205	1	2135	2.59802E-05	0.041438389	1177	0.423537731	1	2109	2.95586E-06	0.004714593	1049
		LoG-sigma-3-0-mm-3D_ngtdm_Strength	TRUE	1.21335E-07	0.00019353	880	0.381030675	1	2044	1.29942E-08	2.07258E-05	772	0.000323344	0.51573403	1344	9.61969E-09	1.53434E-05	758	1.63537E-07	0.000260842	895
	First Order	LoG-sigma-5-0-mm-3D_firstorder_10Percentile	FALSE	5.02525E-13	8.01527E-10	351	8.79106E-11	1.40217E-07	553	0.425657577	1	2110	0.357109779	1	2076	0.223645078	1	1995	0.032239689	1	1742
		LoG-sigma-5-0-mm-3D_firstorder_90Percentile	TRUE	6.81769E-17	1.08742E-13	43	8.04682E-05	0.128346775	1249	0.000517337	0.825151925	1378	0.001826228	1	1475	0.000108863	0.173636713	1269	2.75507E-09	4.39434E-06	701
		LoG-sigma-5-0-mm-3D_firstorder_Energy	TRUE	1.06777E-11	1.7031E-08	468	1.17483E-10	1.87385E-07	565	0.372576783	1	2084	0.216772059	1	1990	0.921402696	1	2301	3.54685E-12	5.65723E-09	425
		LoG-sigma-5-0-mm-3D_firstorder_Entropy	FALSE	1.95692E-17	3.12129E-14	3	4.31899E-12	6.8888E-09	414	0.137899446	1	1922	0.218134891	1	1991	0.00797692	1	1602	0.002223307	1	1491
		LoG-sigma-5-0-mm-3D_firstorder_InterquartileRange	FALSE	6.21298E-17	9.90971E-14	40	3.36759E-12	5.37131E-09	423	0.687701293	1	2218	0.458193314	1	2125	0.13030671	1	1914	0.458193314	1	2125
		LoG-sigma-5-0-mm-3D_firstorder_Kurtosis	TRUE	0.010416519	1	1627	0.003543914	1	1530	0.00201569	1	1483	0.000600479	0.957764371	1389	6.38868E-06	0.010189947	1093	6.1728E-06	0.009845617	1091
		LoG-sigma-5-0-mm-3D_firstorder_Maximum	TRUE	1.5199E-13	2.42424E-10	307	8.23706E-07	0.001313811	979	0.000138144	0.220339191	1285	0.34205087	1	2068	1.60147E-06	0.00255435	1015	0.011931431	1	1640
		LoG-sigma-5-0-mm-3D_firstorder_MeanAbsoluteDeviation	FALSE	2.36302E-17	3.76901E-14	9	4.48307E-14	7.1505E-11	263	0.197057862	1	1975	0.938829964	1	2307	0.016031181	1	1669	0.460410674	1	2126
		LoG-sigma-5-0-mm-3D_firstorder_Mean	TRUE	0.005420062	1	1567	0.001514234	1	1460	0.125728575	1	1909	0.171715519	1	1954	0.000223484	0.356456223	1318	5.96317E-06	0.009511251	1089
		LoG-sigma-5-0-mm-3D_firstorder_Median	TRUE	0.026294529	1	1720	0.057401419	1	1808	0.048874479	1	1789	0.207403536	1	1983	4.12863E-05	0.065851708	1206	1.0493E-06	0.001673641	992
		LoG-sigma-5-0-mm-3D_firstorder_Minimum	TRUE	1.0066E-12	1.60552E-09	377	1.94735E-12	3.10603E-09	402	3.69543E-05	0.058942101	1199	3.65645E-06	0.005832036	1061	6.27946E-06	0.010015742	1092	0.002665924	1	1506
		LoG-sigma-5-0-mm-3D_firstorder_Range	TRUE	2.47968E-14	3.95509E-11	242	2.9574E-12	4.71706E-09	418	0.001144527	1	1438	0.030234373	1	1735	4.84085E-06	0.007721149	1077	0.000815885	1	1412
		LoG-sigma-5-0-mm-3D_firstorder_RobustMeanAbsoluteDeviation	FALSE	4.41526E-17	7.04235E-14	29	1.25405E-13	2.00021E-10	300	0.438506065	1	2116	0.524874142	1	2154	0.051007892	1	1794	0.524874142	1	2154
		LoG-sigma-5-0-mm-3D_firstorder_RootMeanSquared	TRUE	1.61982E-12	2.58361E-09	395	1.35717E-10	2.16468E-07	571	0.947554589	1	2310	1.13964E-05	0.018177183	1127	0.376508178	1	2086	1.97026E-05	0.031425596	1160
		LoG-sigma-5-0-mm-3D_firstorder_Skewness	TRUE	0.99125297	1	2325	0.162730537	1	1946	0.069901448	1	1832	1.64416E-05	0.026224301	1149	0.364790645	1	2080	0.000616864	0.983897489	1391
		LoG-sigma-5-0-mm-3D_firstorder_TotalEnergy	TRUE	1.06777E-11	1.7031E-08	468	1.17483E-10	1.87385E-07	565	0.372576783	1	2084	0.216772059	1	1990	0.918501649	1	2300	3.5468E-12	5.65723E-09	425
		LoG-sigma-5-0-mm-3D_firstorder_Uniformity	FALSE	2.08398E-17	3.32395E-14	5	2.42829E-11	3.87312E-08	481	0.285942489	1	2036	0.000356625	0.568816574	1351	0.731216908	1	2234	0.001514234	1	1460
		LoG-sigma-5-0-mm-3D_firstorder_Variance	FALSE	1.89629E-17	3.02458E-14	2	5.60829E-14	8.94521E-11	271	0.116098395	1	1898	0.222260307	1	1994	0.0066242	1	1585	0.062364054	1	1818
	GLCM	LoG-sigma-5-0-mm-3D_glcm_Autocorrelation	TRUE	0.12304245	1	1906	0.190098977	1	1927	4.01892E-11	6.41018E-08	521	0.015872022	1	1668	1.38293E-12	2.20578E-09	389	4.51473E-06	0.007200987	1073
		LoG-sigma-5-0-mm-3D_glcm_ClusterProminence	FALSE	3.54973E-17	5.66183E-14	22	3.06778E-12	4.8931E-09	401	0.014349124	1	1658	0.002510151	1	1501	0.000517337	0.825151925	1378	0.351414435	1	2073
		LoG-sigma-5-0-mm-3D_glcm_ClusterShade	TRUE	2.77093E-05	0.044196332	1181	0.019947397	1	1653	9.22444E-05	0.147129809	1258	1.86603E-08	2.97631E-05	789	0.05692229	1	1807	0.000247074	0.39408329	1325
		LoG-sigma-5-0-mm-3D_glcm_ClusterTendency	FALSE	1.95692E-17	3.12129E-14	3	8.28483E-12	1.32143E-08	439	0.168301113	1	1951	0.193280825	1	1972	0.012693504	1	1646	0.057401419	1	1808
		LoG-sigma-5-0-mm-3D_glcm_Contrast	FALSE	2.19321E-15	3.49817E-12	158	3.98835E-10	6.36141E-07	595	0.360935981	1	2078	0.912703353	1	2298	0.812244415	1	2263	0.687703022	1	2218
		LoG-sigma-5-0-mm-3D_glcm_Correlation	TRUE	9.57229E-16	1.52678E-12	130	0.000174947	0.279040851	1269	0.00755543	1	1597	0.100841938	1	1879	2.03119E-07	0.000323975	906	0.000187935	0.299756115	1306
		LoG-sigma-5-0-mm-3D_glcm_DifferenceAverage	FALSE	2.39548E-15	3.82079E-12	161	2.72138E-10	4.34059E-07	579	0.178691692	1	1960	0.997084288	1	2327	0.58861707	1	2180	0.731218417	1	2234
		LoG-sigma-5-0-mm-3D_glcm_DifferenceEntropy	FALSE	1.53927E-15	2.45514E-12	146	1.44466E-08	2.30423E-05	753	0.13030671	1	1914	0.04035776	1	1767	0.653083373	1	2205	0.114413543	1	1896
		LoG-sigma-5-0-mm-3D_glcm_DifferenceVariance	FALSE	1.73246E-15	2.76327E-12	150	1.71723E-08	2.73898E-05	761	0.38644809	1	2091	0.143816769	1	1928	0.950464366	1	2311	0.233533745	1	2002
		LoG-sigma-5-0-mm-3D_glcm_Id	FALSE	2.4669E-15	3.93471E-12	162	2.19156E-10	3.49553E-07	570	0.693088015	1	2220	0.008979537	1	1613	0.090654403	1	1865	0.935923232	1	2306
		LoG-sigma-5-0-mm-3D_glcm_Idm	FALSE	2.4669E-15	3.93471E-12	162	2.24504E-10	3.58084E-07	571	0.701197198	1	2223	0.008151701	1	1604	0.092056673	1	1867	0.927208316	1	2303
		LoG-sigma-5-0-mm-3D_glcm_Idmn	TRUE	8.88146E-10	1.41659E-06	651	1.65118E-06	0.002633636	989	1.06654E-10	1.70114E-07	561	0.036593931	1	1756	1.0926E-10	1.74269E-07	562	2.13805E-05	0.034101958	1165
		LoG-sigma-5-0-mm-3D_glcm_Idn	TRUE	5.86216E-10	9.35015E-07	633	4.82155E-07	0.000769037	924	9.73486E-10	1.55271E-06	655	0.812245491	1	2263	2.5217E-10	4.02212E-07	597	0.04483314	1	1779
		LoG-sigma-5-0-mm-3D_glcm_Imc1	TRUE	2.03559E-05	0.032467595	1162	0.26546893	1	1980	4.43583E-06	0.007075155	1072	6.82746E-07	0.00108898	969	0.00072418	1	1403	7.9534E-08	0.000126857	859
		LoG-sigma-5-0-mm-3D_glcm_Imc2	FALSE	1.83751E-17	2.93083E-14	1	1.45009E-06	0.002312895	982	0.035300421	1	1752	0.380467848	1	2088	5.82561E-05	0.092918525	1228	0.000361633	0.576804165	1352
		LoG-sigma-5-0-mm-3D_glcm_InverseVariance	FALSE	2.85669E-15	4.55643E-12	167	2.2998E-10	3.66818E-07	572	0.163831404	1	1947	0.921403154	1	2301	0.655721116	1	2206	0.747771389	1	2240
		LoG-sigma-5-0-mm-3D_glcm_JointAverage	TRUE	0.017533937	1	1678	0.013856474	1	1617	2.57278E-11	4.10359E-08	503	0.006551778	1	1584	2.16182E-12	3.44811E-09	406	9.16216E-08	0.000146136	866
		LoG-sigma-5-0-mm-3D_glcm_JointEnergy	FALSE	2.47144E-16	3.94194E-13	84	1.57586E-11	2.51349E-08	464	0.918501649	1	2300	0.021323005	1	1698	0.353303643	1	2074	0.018972669	1	1686
		LoG-sigma-5-0-mm-3D_glcm_JointEntropy	FALSE	2.36302E-17	3.76901E-14	9	6.22672E-12	9.93162E-09	428	0.950464366	1	2311	0.904014002	1	2295	0.226438582	1	1997	0.071612261	1	1835
		LoG-sigma-5-0-mm-3D_glcm_MCC	FALSE	2.51905E-09	4.01789E-06	697	0.000271229	0.432610104	1299	0.778416214	1	2251	0.639962105	1	2200	0.786836709	1	2254	0.024151552	1	1711
		LoG-sigma-5-0-mm-3D_glcm_MaximumProbability	FALSE	3.48001E-14	5.55061E-11	254	3.67612E-09	5.86341E-06	691	0.376508178	1	2086	0.092058573	1	1867	0.075732022	1	1842	0.252706887	1	2015
		LoG-sigma-5-0-mm-3D_glcm_SumAverage	TRUE	0.017533937	1	1678	0.013856474	1	1617	2.57278E-11	4.10359E-08	503	0.006551778	1	1584	2.16182E-12	3.44811E-09	406	9.16216E-08	0.000146136	866
		LoG-sigma-5-0-mm-3D_glcm_SumEntropy	FALSE	2.01947E-17	3.22105E-14	4	6.90942E-12	1.10205E-08	432	0.834997126	1	2271	0.855021221	1	2278	0.12482817	1	1908	0.036267099	1	1755
		LoG-sigma-5-0-mm-3D_glcm_SumSquares	FALSE	1.95692E-17	3.12129E-14	3	1.04527E-11	1.66721E-08	448	0.181064189	1	1962	0.222260307	1	1994	0.016031181	1	1669	0.061344092	1	1816
	GLDM	LoG-sigma-5-0-mm-3D_gldm_DependenceEntropy	FALSE	2.59619E-17	4.14092E-14	12	6.86425E-09	1.09485E-05	719	0.402681349	1	2099	0.809412113	1	2262	0.551404963	1	2165	0.586102738	1	2179
		LoG-sigma-5-0-mm-3D_gldm_DependenceNonUniformity	TRUE	9.57413E-17	1.52707E-13	54	7.66795E-08	0.000122304	832	0.000105645	0.168504322	1267	0.04217423	1	1772	3.67729E-09	5.86527E-06	714	8.40478E-06	0.01340563	1109
		LoG-sigma-5-0-mm-3D_gldm_DependenceNonUniformityNormalized	FALSE	6.89185E-16	1.09925E-12	119	2.1762E-06	0.003471044	1004	0.119530921	1	1902	0.133117321	1	1917	0.030795103	1	1737	0.038952757	1	1763
Laplacian of Gaussian - Sigma 5mm		LoG-sigma-5-0-mm-3D_gldm_DependenceVariance	TRUE	0.000164814	0.262877789	1297	0.683050111	1	2170	0.78122021	1	2252	2.60871E-06	0.004160894	1042	0.742238913	1	2238	0.000148733	0.237229908	1290
		LoG-sigma-5-0-mm-3D_gldm_GrayLevelNonUniformity	TRUE	2.28998E-17	3.65252E-14	8	4.23279E-14	6.7513E-11	245	0.634741941	1	2198	0.029408327	1	1732	0.004571654	1	1552	5.65178E-07	0.000901459	959
		LoG-sigma-5-0-mm-3D_gldm_GrayLevelVariance	FALSE	2.01947E-17	3.22105E-14	4	2.98782E-12	4.76558E-09	400	0.115252483	1	1897	0.255751271	1	2017	0.007890975	1	1601	0.086552279	1	1859
		LoG-sigma-5-0-mm-3D_gldm_HighGrayLevelEmphasis	TRUE	0.134061509	1	1918	0.168481382	1	1909	3.64102E-11	5.80743E-08	517	0.012180991	1	1642	1.08998E-12	1.73852E-09	380	2.51694E-06	0.004014526	1040
		LoG-sigma-5-0-mm-3D_gldm_LargeDependenceEmphasis	FALSE	1.73246E-15	2.76327E-12	150	5.84221E-11	9.31833E-08	516	0.829295192	1	2269	0.004317026	1	1547	0.092764314	1	1868	0.682332129	1	2216
		LoG-sigma-5-0-mm-3D_gldm_LargeDependenceHighGrayLevelEmphasis	TRUE	3.96371E-08	6.32211E-05	825	4.39924E-06	0.007016786	1043	1.86567E-08	2.97574E-05	789	0.020914498	1	1696	1.34077E-11	2.13852E-08	477	1.32498E-05	0.021133429	1136
		LoG-sigma-5-0-mm-3D_gldm_LargeDependenceLowGrayLevelEmphasis	TRUE	0.682330374	1	2216	0.130858077	1	1873	1.48303E-11	2.36543E-08	481	1.14686E-10	1.82924E-07	564	1.03367E-12	1.6487E-09	378	4.89341E-13	7.80499E-10	350
		LoG-sigma-5-0-mm-3D_gldm_LowGrayLevelEmphasis	TRUE	6.57391E-07	0.001048539	967	2.3844E-06	0.003803117	1009	2.16061E-11	3.44617E-08	496	4.24641E-07	0.000677303	944	1.3118E-12	2.09232E-09	387	3.28132E-12	5.23371E-09	422
		LoG-sigma-5-0-mm-3D_gldm_SmallDependenceEmphasis	FALSE	6.69489E-05	0.106783469	1237	0.007445095	1	1559	0.544103696	1	2162	0.469339873	1	2130	0.624359996	1	2194	0.442840596	1	2118
		LoG-sigma-5-0-mm-3D_gldm_SmallDependenceHighGrayLevelEmphasis	TRUE	0.007638095	1	1598	0.077254177	1	1804	1.16745E-08	1.86208E-05	767	0.517752969	1	2151	1.0926E-10	1.74269E-07	562	0.698491973	1	2222
		LoG-sigma-5-0-mm-3D_gldm_SmallDependenceLowGrayLevelEmphasis	TRUE	0.000815828	1	1412	0.001753441	1	1437	7.25953E-09	1.15789E-05	745	0.376510966	1	2086	2.576E-09	4.10873E-06	698	0.176344927	1	1958
	GLRLM	LoG-sigma-5-0-mm-3D_glrlm_GrayLevelNonUniformity	TRUE	0.725726477	1	2232	0.688507875	1	2172	7.7925E-08	0.00012429	858	0.131239923	1	1915	5.3979E-11	8.60965E-08	533	1.42033E-12	2.26543E-09	390
		LoG-sigma-5-0-mm-3D_glrlm_GrayLevelNonUniformityNormalized	TRUE	2.7641E-17	4.40874E-14	14	7.86659E-12	1.25472E-08	437	2.95507E-05	0.047133319	1185	2.64033E-05	0.042113249	1178	1.46399E-05	0.023350629	1142	9.62769E-06	0.015356173	1117
		LoG-sigma-5-0-mm-3D_glrlm_GrayLevelVariance	TRUE	2.51605E-17	4.0131E-14	11	6.30109E-14	1.00502E-10	259	0.005928461	1	1575	0.017020483	1	1675	2.24492E-05	0.03580653	1168	0.006551778	1	1584
		LoG-sigma-5-0-mm-3D_glrlm_HighGrayLevelRunEmphasis	TRUE	0.003845356	1	1537	0.004362201	1	1512	1.12329E-11	1.79164E-08	470	0.003421356	1	1527	1.06146E-12	1.69303E-09	379	4.13594E-06	0.006596822	1068
		LoG-sigma-5-0-mm-3D_glrlm_LongRunEmphasis	FALSE	2.70716E-16	4.31792E-13	88	3.80298E-10	6.06576E-07	593	0.051443802	1	1795	0.127546784	1	1911	0.002633928	1	1505	0.05934559	1	1812
		LoG-sigma-5-0-mm-3D_glrlm_LongRunHighGrayLevelEmphasis	TRUE	5.53177E-11	8.82317E-08	534	3.75971E-09	5.99673E-06	692	0.000167242	0.266751349	1298	0.017708966	1	1679	4.48583E-09	7.1549E-06	723	3.78772E-06	0.006041406	1063
		LoG-sigma-5-0-mm-3D_glrlm_LongRunLowGrayLevelEmphasis	TRUE	3.2322E-17	5.15536E-14	19	0.010210827	1	1588	1.24426E-12	1.98459E-09	385	6.72798E-11	1.07311E-07	542	4.77467E-11	7.61559E-08	528	9.83131E-09	1.56809E-05	759
		LoG-sigma-5-0-mm-3D_glrlm_LowGrayLevelRunEmphasis	TRUE	4.43356E-10	7.07154E-07	621	2.11323E-06	0.0003369	882	1.09519E-11	1.74682E-08	469	4.51473E-06	0.007200987	1073	1.03367E-12	1.6487E-09	378	1.56707E-10	2.49948E-07	577
		LoG-sigma-5-0-mm-3D_glrlm_RunEntropy	TRUE	0.016354974	1	1671	0.079138024	1	1807	1.02988E-06	0.001642654	991	0.487482144	1	2138	1.98894E-10	3.17236E-07	587	0.001694948	1	1469
		LoG-sigma-5-0-mm-3D_glrlm_RunLengthNonUniformity	TRUE	2.47968E-14	3.95509E-11	242	1.04527E-11	1.66721E-08	448	0.142815309	1	1927	0.770022959	1	2248	0.28925547	1	2038	2.55956E-08	4.0825E-05	804
		LoG-sigma-5-0-mm-3D_glrlm_RunLengthNonUniformityNormalized	FALSE	1.69622E-13	2.70548E-10	311	3.45714E-10	5.51414E-07	589	0.212717676	1	1987	0.386450864	1	2091	0.679650915	1	2215	0.904014002	1	2295
		LoG-sigma-5-0-mm-3D_glrlm_RunPercentage	FALSE	2.54042E-15	4.05197E-12	163	8.89567E-11	1.41886E-07	533	0.212717676	1	1987	0.642577912	1	2201	0.98250699	1	2322	0.578583315	1	2176
		LoG-sigma-5-0-mm-3D_glrlm_RunVariance	FALSE	3.66227E-17	5.84132E-14	23	5.46575E-12	8.71787E-09	423	0.043295742	1	1775	0.492076154	1	2140	0.002540475	1	1502	0.208723612	1	1984
		LoG-sigma-5-0-mm-3D_glrlm_ShortRunEmphasis	FALSE	2.73445E-12	4.36145E-09	415	4.02418E-06	0.006418574	1038	0.120401356	1	1903	5.14421E-05	0.082050207	1220	0.372576783	1	2084	0.001991165	1	1482
		LoG-sigma-5-0-mm-3D_glrlm_ShortRunHighGrayLevelEmphasis	TRUE	1.89644E-10	3.02483E-07	585	0.275146763	1	1986	2.05154E-12	3.27221E-09	404	0.075733732	1	1842	1.03367E-12	1.6487E-09	378	0.040715651	1	1768
		LoG-sigma-5-0-mm-3D_glrlm_ShortRunLowGrayLevelEmphasis	TRUE	2.70406E-11	4.31298E-08	505	1.68201E-06	0.002682809	990	1.90652E-11	3.04091E-08	491	0.040715651	1	1768	2.59557E-12	4.13993E-09	413	0.013637986	1	1653
	GLSZM	LoG-sigma-5-0-mm-3D_glszm_GrayLevelNonUniformity	FALSE	0.641420725	1	2109	0.040236862	1	388	0.026048477	1	1719	0.004294692	1	1547	0.031079295	1	1738	0.00652076	1	1584
		LoG-sigma-5-0-mm-3D_glszm_GrayLevelNonUniformityNormalized	FALSE	0.335306817	1	1977	0.009461682	1	335	0.053218743	1	1799	0.001235367	1	1444	0.077556634	1	1845	0.001283595	1	1447
		LoG-sigma-5-0-mm-3D_glszm_GrayLevelVariance	FALSE	0.055925065	1	1765	0.018153	1	375	0.614052096	1	2190	0.017528568	1	1678	0.421424076	1	2108	0.11951333	1	1902
		LoG-sigma-5-0-mm-3D_glszm_HighGrayLevelZoneEmphasis	TRUE	0.523184914	1	2108	0.352333782	1	519	1.1395E-05	0.01817504	1127	0.026785426	1	1722	7.49931E-07	0.00119614	974	0.236389332	1	2004
		LoG-sigma-5-0-mm-3D_glszm_LargeAreaEmphasis	TRUE	0.161630372	1	1945	0.021371696	1	1660	0.001251937	1	1445	2.282E-05	0.036397871	1169	0.000187919	0.299730128	1306	4.59437E-06	0.007328021	1074
		LoG-sigma-5-0-mm-3D_glszm_LargeAreaHighGrayLevelEmphasis	TRUE	0.795282205	1	2257	0.12619354	1	1868	1.78524E-05	0.02847451	1154	9.56267E-07	0.001525246	987	5.01257E-06	0.007995052	1079	2.45437E-08	3.91471E-05	802
		LoG-sigma-5-0-mm-3D_glszm_LargeAreaLowGrayLevelEmphasis	TRUE	0.156225546	1	1940	0.518367736	1	2106	0.067127353	1	1827	1.85257E-06	0.002954849	1023	0.00797692	1	1602	1.99198E-07	0.000317721	905
		LoG-sigma-5-0-mm-3D_glszm_LowGrayLevelZoneEmphasis	FALSE	0.442273618	1	2073	0.342129205	1	517	0.001826115	1	1475	0.571088978	1	2173	0.00201569	1	1483	0.32199275	1	2057
		LoG-sigma-5-0-mm-3D_glszm_SizeZoneNonUniformity	FALSE	0.94464551	1	2309	0.973264431	1	766	0.445013007	1	2119	0.00300187	1	1520	0.480632205	1	2135	0.009618309	1	1623
		LoG-sigma-5-0-mm-3D_glszm_SizeZoneNonUniformityNormalized	FALSE	0.892445612	1	2291	0.487419648	1	793	0.566150423	1	2171	0.462590321	1	2127	0.608926434	1	2188	0.54649527	1	2163
		LoG-sigma-5-0-mm-3D_glszm_SmallAreaEmphasis	FALSE	0.784027053	1	2253	0.383051849	1	2045	0.576084726	1	2175	0.895336131	1	2292	0.632139605	1	2197	0.921403154	1	2301
		LoG-sigma-5-0-mm-3D_glszm_SmallAreaHighGrayLevelEmphasis	FALSE	0.750540389	1	2241	0.84985391	1	2229	0.988337474	1	2324	0.255751271	1	2017	0.901119478	1	2294	0.187100185	1	1967
		LoG-sigma-5-0-mm-3D_glszm_SmallAreaLowGrayLevelEmphasis	FALSE	0.558754064	1	2168	0.1661982	1	1907	0.626948606	1	2195	0.473840131	1	2132	0.621776014	1	2193	0.384450175	1	2090
		LoG-sigma-5-0-mm-3D_glszm_ZoneEntropy	FALSE	0.215412683	1	1989	0.492654705	1	638	0.895335523	1	2292	0.536810034	1	2159	0.789649143	1	2255	0.321967302	1	2057
		LoG-sigma-5-0-mm-3D_glszm_ZonePercentage	FALSE	0.460220103	1	1699	0.717519451	1	464	0.915601686	1	2299	0.182261725	1	1963	0.895335523	1	2292	0.332848451	1	2063
		LoG-sigma-5-0-mm-3D_glszm_ZoneVariance	TRUE	0.10547542	1	1885	0.013019555	1	1611	0.000882784	1	1418	7.57007E-05	0.120742618	1245	9.365E-05	0.149371706	1259	4.4364E-06	0.007076064	1072
	NGTDM	LoG-sigma-5-0-mm-3D_ngtdm_Busyness	TRUE	4.97327E-08	7.93236E-05	836	3.42496E-06	0.005462813	1029	3.7321E-11	5.9527E-08	518	0.016519646	1	1672	1.68177E-11	2.68243E-08	486	0.002864491	1	1512
		LoG-sigma-5-0-mm-3D_ngtdm_Coarseness	TRUE	0.031945414	1	1741	0.252933689	1	1972	0.00027294	0.435339526	1332	0.017361732	1	1677	4.64544E-10	7.40948E-07	623	0.000276862	0.441595142	1333
		LoG-sigma-5-0-mm-3D_ngtdm_Complexity	TRUE	1.78437E-15	2.84607E-12	151	7.50151E-07	0.001196491	947	2.47059E-07	0.00039406	916	0.031946416	1	1741	2.5217E-10	4.02212E-07	597	0.962109916	1	2315
		LoG-sigma-5-0-mm-3D_ngtdm_Contrast	TRUE	1.64345E-10	2.6213E-07	579	7.66551E-09	1.22265E-05	724	8.10118E-10	1.29214E-06	647	0.069340852	1	1831	7.41902E-09	1.18333E-05	746	0.069903083	1	1832
		LoG-sigma-5-0-mm-3D_ngtdm_Strength	TRUE	4.98191E-10	7.94615E-07	626	0.09558902	1	1831	2.56888E-07	0.000409737	918	8.45166E-09	1.34804E-05	752	8.67978E-10	1.38443E-06	650	2.32929E-11	3.71521E-08	499
	First Order	LoG-sigma-10-0-mm-3D_firstorder_10Percentile	TRUE	1.54126E-08	2.45831E-05	780	1.97026E-05	0.031425596	1160	0.023695451	1	1709	0.042919946	1	1774	0.480632205	1	2135	2.074E-08	3.30802E-05	794
		LoG-sigma-10-0-mm-3D_firstorder_90Percentile	FALSE	0.012434652	1	1644	0.364793447	1	2080	0.988337474	1	2324	0.094915399	1	1871	0.000882784	1	1418	0.207403536	1	1983
		LoG-sigma-10-0-mm-3D_firstorder_Energy	TRUE	1.50879E-08	2.40653E-05	779	4.67536E-06	0.007457201	1075	0.469337277	1	2130	0.19079081	1	1970	0.025804417	1	1718	1.3754E-11	2.19376E-08	478
		LoG-sigma-10-0-mm-3D_firstorder_Entropy	TRUE	1.61982E-12	2.58361E-09	395	0.000735458	1	1272	0.246691337	1	2011	1.26022E-05	0.020100441	1133	0.002830324	1	1511	3.30668E-07	0.000527416	931
		LoG-sigma-10-0-mm-3D_firstorder_InterquartileRange	TRUE	4.32728E-11	6.90202E-08	524	3.80356E-08	6.06669E-05	823	0.829295192	1	2269	4.01825E-09	6.40911E-06	718	0.054125223	1	1801	1.27797E-12	2.03837E-09	386
		LoG-sigma-10-0-mm-3D_firstorder_Kurtosis	TRUE	0.823602236	1	2267	0.789650343	1	2255	5.80933E-11	9.26587E-08	536	0.353306456	1	2074	8.61791E-15	1.37456E-11	205	0.015872022	1	1668
		LoG-sigma-10-0-mm-3D_firstorder_Maximum	TRUE	4.67476E-06	0.007456247	1075	0.011209738	1	1634	9.01832E-16	1.43842E-12	128	0.032833163	1	1744	6.68853E-16	1.06682E-12	118	0.01132726	1	1635
		LoG-sigma-10-0-mm-3D_firstorder_MeanAbsoluteDeviation	TRUE	1.37503E-11	2.19317E-08	478	7.48402E-08	0.00011937	856	0.15945174	1	1943	6.37162E-09	1.01627E-05	739	0.004028015	1	1541	2.10659E-12	3.36002E-09	405
		LoG-sigma-10-0-mm-3D_firstorder_Mean	FALSE	7.01096E-05	0.111824875	1240	0.023027305	1	1706	0.784027053	1	2253	0.950464655	1	2311	0.002510005	1	1501	8.81546E-05	0.140606643	1255
		LoG-sigma-10-0-mm-3D_firstorder_Median	FALSE	9.79891E-05	0.156292536	1262	0.105477456	1	1885	0.415120353	1	2105	0.956286119	1	2313	0.000153161	0.244291122	1292	0.000301377	0.480696557	1339
		LoG-sigma-10-0-mm-3D_firstorder_Minimum	TRUE	9.53736E-08	0.000152121	868	1.46185E-06	0.002331648	1010	0.012306988	1	1643	0.616624097	1	2191	0.619196686	1	2192	2.3044E-08	3.67551E-05	799
		LoG-sigma-10-0-mm-3D_firstorder_Range	TRUE	3.28829E-08	5.24482E-05	816	1.36784E-07	0.00021817	886	2.48661E-13	3.96614E-10	325	3.05177E-05	0.048675684	1187	9.29124E-16	1.48195E-12	129	5.59754E-10	8.92807E-07	631
		LoG-sigma-10-0-mm-3D_firstorder_RobustMeanAbsoluteDeviation	TRUE	1.06654E-10	1.70114E-07	561	1.03382E-07	0.000164894	872	0.49900798	1	2143	5.83857E-09	9.31251E-06	735	0.035300421	1	1752	1.84782E-12	2.94727E-09	400
		LoG-sigma-10-0-mm-3D_firstorder_RootMeanSquared	TRUE	2.63421E-09	4.20156E-06	699	0.000179914	0.286962801	1303	0.38644809	1	2091	3.19376E-10	5.09404E-07	607	0.003935723	1	1539	4.19964E-09	6.69843E-06	720
		LoG-sigma-10-0-mm-3D_firstorder_Skewness	FALSE	0.425657577	1	2110	0.313171409	1	2052	0.432054154	1	2113	0.00204074	1	1484	0.073353852	1	1838	0.00242073	1	1498
		LoG-sigma-10-0-mm-3D_firstorder_TotalEnergy	TRUE	1.50879E-08	2.40653E-05	779	4.75772E-06	0.00758856	1076	0.469337277	1	2130	0.19079081	1	1970	0.026048477	1	1719	1.34113E-11	2.13911E-08	477
		LoG-sigma-10-0-mm-3D_firstorder_Uniformity	TRUE	1.18013E-12	1.88231E-09	383	0.000823251	1	1280	0.442837931	1	2118	2.42828E-06	0.003873106	1038	0.485191301	1	2137	1.6811E-09	2.68136E-06	679
		LoG-sigma-10-0-mm-3D_firstorder_Variance	TRUE	1.01495E-11	1.61884E-08	466	3.96443E-08	6.32326E-05	825	0.019728911	1	1690	3.63705E-13	5.8011E-10	339	0.000193451	0.308554113	1308	1.09031E-12	1.73904E-09	380
	GLCM	LoG-sigma-10-0-mm-3D_glcm_Autocorrelation	TRUE	0.005797485	1	1573	0.112242146	1	1771	0.000314358	0.501400936	1342	0.080046751	1	1849	6.62324E-08	0.000105641	850	0.260365473	1	2020
		LoG-sigma-10-0-mm-3D_glcm_ClusterProminence	TRUE	8.17186E-11	1.30341E-07	550	6.91218E-06	0.011024932	1012	0.000677493	1	1398	7.20783E-14	1.14965E-10	280	4.79196E-09	7.64317E-06	726	1.11955E-10	1.78568E-07	563
		LoG-sigma-10-0-mm-3D_glcm_ClusterShade	TRUE	0.742238913	1	2238	0.801837792	1	2120	1.73502E-07	0.000276736	898	2.2027E-09	3.5133E-06	691	4.12863E-05	0.065851708	1206	0.015556663	1	1666
		LoG-sigma-10-0-mm-3D_glcm_ClusterTendency	TRUE	2.16182E-12	3.44811E-09	406	9.88184E-06	0.015761528	1032	0.019347206	1	1688	1.53001E-10	2.44036E-07	576	0.000179898	0.286937784	1303	1.41053E-11	2.24979E-08	479
		LoG-sigma-10-0-mm-3D_glcm_Contrast	TRUE	2.576E-09	4.10873E-06	698	0.026915218	1	1608	0.927207892	1	2303	1.93824E-05	0.03091489	1159	0.276157862	1	2030	6.09652E-07	0.000972395	963
		LoG-sigma-10-0-mm-3D_glcm_Correlation	TRUE	4.54559E-11	7.25022E-08	526	0.032359219	1	1627	0.261913477	1	2021	3.12025E-07	0.00049768	928	2.72663E-05	0.04348975	1180	2.43514E-05	0.038840412	1173
		LoG-sigma-10-0-mm-3D_glcm_DifferenceAverage	TRUE	2.75449E-09	4.39341E-06	701	0.027180651	1	1609	0.803751758	1	2260	2.00278E-05	0.031944331	1161	0.314925095	1	2053	6.33136E-07	0.001009851	965
		LoG-sigma-10-0-mm-3D_glcm_DifferenceEntropy	TRUE	9.96041E-10	1.58868E-06	656	0.05276118	1	1680	0.578581096	1	2176	3.05177E-05	0.048675684	1187	0.359018267	1	2077	1.6017E-06	0.002554707	1015
		LoG-sigma-10-0-mm-3D_glcm_DifferenceVariance	TRUE	1.64317E-09	2.62086E-06	678	0.037650505	1	1643	0.753314033	1	2242	3.00321E-05	0.047901168	1186	0.309674942	1	2050	1.15102E-06	0.001835881	997
		LoG-sigma-10-0-mm-3D_glcm_Id	TRUE	2.81661E-09	4.4925E-06	702	0.027990549	1	1612	0.295958732	1	2042	0.001317505	1	1449	0.314925095	1	2053	1.88646E-06	0.003008911	1024
		LoG-sigma-10-0-mm-3D_glcm_Idm	TRUE	2.69369E-09	4.29644E-06	700	0.027990549	1	1612	0.285942489	1	2036	0.001334363	1	1450	0.31668807	1	2054	1.85257E-06	0.002954849	1023
		LoG-sigma-10-0-mm-3D_glcm_Idmn	TRUE	5.33811E-07	0.000851428	956	0.212330166	1	1860	1.00435E-08	1.60194E-05	760	0.081315056	1	1851	1.60462E-10	2.55937E-07	578	1.60332E-07	0.000255729	894
		LoG-sigma-10-0-mm-3D_glcm_Idn	TRUE	8.54956E-07	0.001363655	981	0.231161227	1	1873	3.36662E-12	5.36976E-09	423	8.42286E-05	0.13434461	1252	1.06146E-12	1.69303E-09	379	1.10195E-05	0.017576095	1125
		LoG-sigma-10-0-mm-3D_glcm_Imc1	FALSE	0.068780721	1	1830	0.967908381	1	2175	0.006062098	1	1577	0.085218095	1	1857	0.033434932	1	1746	0.085218095	1	1857
		LoG-sigma-10-0-mm-3D_glcm_Imc2	TRUE	3.38334E-14	5.39643E-11	253	0.002393121	1	1393	0.568626276	1	2172	1.01109E-06	0.001612687	990	0.000561232	0.895164319	1384	3.06039E-07	0.000488133	927
		LoG-sigma-10-0-mm-3D_glcm_InverseVariance	TRUE	3.21906E-09	5.1344E-06	708	0.028265098	1	1613	0.742238913	1	2238	2.06936E-05	0.033006362	1163	0.359018267	1	2077	7.50042E-07	0.001196318	974
		LoG-sigma-10-0-mm-3D_glcm_JointAverage	TRUE	0.000705158	1	1401	0.02511909	1	1601	7.68595E-05	0.122590838	1246	0.105477456	1	1885	7.25953E-09	1.15789E-05	745	0.002223307	1	1491
		LoG-sigma-10-0-mm-3D_glcm_JointEnergy	TRUE	1.99849E-12	3.18759E-09	403	0.000990348	1	1326	0.690392694	1	2219	1.95605E-06	0.003119901	1026	0.271352173	1	2027	1.01933E-09	1.62583E-06	657
		LoG-sigma-10-0-mm-3D_glcm_JointEntropy	TRUE	3.93276E-12	6.27276E-09	429	0.000635796	1	1294	0.458190688	1	2125	3.72153E-06	0.005935836	1062	0.01449533	1	1659	9.34874E-08	0.000149112	867
		LoG-sigma-10-0-mm-3D_glcm_MCC	TRUE	4.87205E-08	7.77092E-05	835	0.148057611	1	1808	0.001144527	1	1438	0.000113874	0.181629346	1272	4.58024E-08	7.30548E-05	832	0.007891338	1	1601
		LoG-sigma-10-0-mm-3D_glcm_MaximumProbability	TRUE	5.8692E-07	0.000936138	961	0.008151841	1	1495	0.532042731	1	2157	1.34087E-07	0.000213869	885	0.046413752	1	1783	3.21974E-09	5.13548E-06	708
		LoG-sigma-10-0-mm-3D_glcm_SumAverage	TRUE	0.000705158	1	1401	0.02511909	1	1601	7.68595E-05	0.122590838	1246	0.105477456	1	1885	7.25953E-09	1.15789E-05	745	0.002223307	1	1491
		LoG-sigma-10-0-mm-3D_glcm_SumEntropy	TRUE	2.88062E-12	4.59458E-09	417	0.000521413	0.831653546	1280	0.408872696	1	2102	3.40688E-06	0.005433974	1057	0.009571932	1	1619	6.89973E-08	0.000110051	852
		LoG-sigma-10-0-mm-3D_glcm_SumSquares	TRUE	3.1963E-12	5.09809E-09	421	1.09882E-05	0.017526187	1038	0.020117175	1	1692	1.76595E-10	2.81669E-07	582	0.000193451	0.308554113	1308	1.59976E-11	2.55162E-08	484
	GLDM	LoG-sigma-10-0-mm-3D_gldm_DependenceEntropy	TRUE	4.71187E-12	7.51543E-09	436	0.007614434	1	1489	0.770021653	1	2248	1.26022E-05	0.020100441	1133	0.015556059	1	1666	3.78445E-07	0.00060362	938
		LoG-sigma-10-0-mm-3D_gldm_DependenceNonUniformity	TRUE	3.29796E-11	5.26025E-08	513	4.10089E-05	0.065409202	1115	0.00322546	1	1522	0.005420334	1	1567	1.07599E-07	0.000171621	874	5.81081E-11	9.26825E-08	536
		LoG-sigma-10-0-mm-3D_gldm_DependenceNonUniformityNormalized	TRUE	5.34212E-10	8.52068E-07	629	0.00483032	1	1450	0.994168581	1	2326	0.007721927	1	1599	0.103911835	1	1883	3.65645E-06	0.005832036	1061
Laplacian of Gaussian - Sigma 10mm		LoG-sigma-10-0-mm-3D_gldm_DependenceVariance	FALSE	0.000421202	0.671816619	1363	0.919124009	1	2159	0.855020384	1	2278	0.026049349	1	1719	0.106264183	1	1886	0.016852077	1	1674
		LoG-sigma-10-0-mm-3D_gldm_GrayLevelNonUniformity	TRUE	1.99901E-13	3.18843E-10	317	7.81491E-06	0.012464783	991	0.489773714	1	2139	0.384450175	1	2090	0.013637437	1	1653	6.44805E-14	1.02846E-10	276
		LoG-sigma-10-0-mm-3D_gldm_GrayLevelVariance	TRUE	7.28894E-12	1.16259E-08	453	2.43239E-05	0.038796585	1055	0.019347206	1	1688	2.18788E-10	3.48967E-07	591	0.000199136	0.317621788	1310	1.1236E-11	1.79214E-08	470
		LoG-sigma-10-0-mm-3D_gldm_HighGrayLevelEmphasis	TRUE	0.003670255	1	1533	0.099663152	1	1677	0.000309966	0.494395054	1341	0.084557266	1	1856	6.23017E-08	9.93713E-05	847	0.376510966	1	2086
		LoG-sigma-10-0-mm-3D_gldm_LargeDependenceEmphasis	TRUE	2.15334E-09	3.43458E-06	690	0.009754583	1	1511	0.272947663	1	2028	0.01651964	1	1672	0.434198658	1	2114	2.94399E-07	0.000469566	925
		LoG-sigma-10-0-mm-3D_gldm_LargeDependenceHighGrayLevelEmphasis	TRUE	0.000107243	0.171052355	1268	0.027718302	1	1611	0.000122682	0.195678463	1277	0.003543914	1	1530	6.66426E-09	1.06136E-05	741	1.01109E-06	0.001612687	990
		LoG-sigma-10-0-mm-3D_gldm_LargeDependenceLowGrayLevelEmphasis	TRUE	3.00853E-06	0.004798608	1050	0.002216866	1	1387	3.94429E-10	6.29114E-07	616	5.64809E-05	0.090087039	1226	1.52084E-11	2.42574E-08	482	1.45193E-07	0.000231583	889
		LoG-sigma-10-0-mm-3D_gldm_LowGrayLevelEmphasis	TRUE	1.31419E-07	0.000209614	884	0.002096726	1	1349	8.17186E-11	1.30341E-07	550	2.64033E-05	0.042113249	1178	2.52874E-12	4.03335E-09	412	8.62379E-08	0.000137549	863
		LoG-sigma-10-0-mm-3D_gldm_SmallDependenceEmphasis	FALSE	0.798102763	1	2258	0.801837792	1	2120	0.823602236	1	2267	0.00937098	1	1617	0.578581096	1	2176	0.027556048	1	1725
		LoG-sigma-10-0-mm-3D_gldm_SmallDependenceHighGrayLevelEmphasis	TRUE	0.979592079	1	2321	0.360803539	1	1947	0.003302485	1	1524	0.040715651	1	1768	5.56346E-06	0.008873714	1085	0.279396521	1	2032
		LoG-sigma-10-0-mm-3D_gldm_SmallDependenceLowGrayLevelEmphasis	TRUE	2.77093E-05	0.044196332	1181	0.225244803	1	1869	1.56669E-10	2.49887E-07	577	0.000409757	0.653562667	1361	9.64688E-12	1.53868E-08	464	7.71744E-06	0.012309313	1104
	GLRLM	LoG-sigma-10-0-mm-3D_glrlm_GrayLevelNonUniformity	TRUE	1.95578E-06	0.003119472	1026	0.310842186	1	1921	0.020313795	1	1693	0.103138991	1	1882	0.032534253	1	1743	5.58796E-15	8.9128E-12	190
		LoG-sigma-10-0-mm-3D_glrlm_GrayLevelNonUniformityNormalized	FALSE	7.31288E-13	1.1664E-09	365	0.032983693	1	1629	0.022374023	1	1703	0.117806995	1	1900	0.271352173	1	2027	0.01019982	1	1625
		LoG-sigma-10-0-mm-3D_glrlm_GrayLevelVariance	TRUE	2.10727E-11	3.3611E-08	495	0.000506735	0.808241532	1278	0.002697964	1	1507	1.06546E-05	0.016994013	1123	2.8016E-06	0.004468549	1046	8.45166E-09	1.34804E-05	752
		LoG-sigma-10-0-mm-3D_glrlm_HighGrayLevelRunEmphasis	TRUE	0.000269092	0.429202479	1331	0.000323209	0.515518956	1247	0.000288852	0.460719658	1336	0.123934996	1	1907	5.85975E-08	9.3463E-05	844	0.218134891	1	1991
		LoG-sigma-10-0-mm-3D_glrlm_LongRunEmphasis	TRUE	2.5217E-10	4.02212E-07	597	0.002891305	1	1408	0.513030982	1	2149	0.015556663	1	1666	0.00051034	0.813991712	1377	9.959E-06	0.015884599	1119
		LoG-sigma-10-0-mm-3D_glrlm_LongRunHighGrayLevelEmphasis	TRUE	1.48081E-07	0.000236189	890	0.001180941	1	1339	4.51415E-06	0.007200063	1073	3.69582E-05	0.058948276	1199	2.61943E-07	0.000417799	919	1.6431E-08	2.62074E-05	783
		LoG-sigma-10-0-mm-3D_glrlm_LongRunLowGrayLevelEmphasis	TRUE	0.706622361	1	2225	0.130644919	1	1791	3.5233E-05	0.056196654	1196	6.19848E-05	0.098865735	1232	4.2831E-06	0.006831538	1070	4.49734E-07	0.000717326	947
		LoG-sigma-10-0-mm-3D_glrlm_LowGrayLevelRunEmphasis	TRUE	4.10712E-09	6.55085E-06	719	0.000721224	1	1303	1.19578E-09	1.90727E-06	664	8.55185E-05	0.136402071	1253	9.6828E-11	1.54441E-07	557	3.85792E-07	0.000615339	939
		LoG-sigma-10-0-mm-3D_glrlm_RunEntropy	TRUE	2.42282E-07	0.00038644	915	0.307197759	1	1919	0.079416897	1	1848	0.077558367	1	1845	3.9926E-06	0.006368197	1066	0.566152692	1	2171
		LoG-sigma-10-0-mm-3D_glrlm_RunLengthNonUniformity	TRUE	5.58743E-09	8.91195E-06	733	0.000156912	0.250274996	1199	0.311418531	1	2051	2.06936E-05	0.033006362	1163	0.94173717	1	2308	1.34113E-11	2.13911E-08	477
		LoG-sigma-10-0-mm-3D_glrlm_RunLengthNonUniformityNormalized	TRUE	2.0736E-08	3.3074E-05	794	0.006187548	1	1471	0.85215388	1	2277	3.34709E-06	0.005338607	1056	0.081953512	1	1852	4.79294E-09	7.64475E-06	726
		LoG-sigma-10-0-mm-3D_glrlm_RunPercentage	TRUE	3.07906E-09	4.9111E-06	706	0.023900539	1	1596	0.863631236	1	2281	1.81505E-05	0.02895002	1155	0.081313277	1	1851	8.45124E-08	0.000134797	862
		LoG-sigma-10-0-mm-3D_glrlm_RunVariance	FALSE	6.28429E-10	1.00234E-06	636	0.000206525	0.329407605	1217	0.183459991	1	1964	0.023696267	1	1709	0.000633617	1	1393	0.000327914	0.523022408	1345
		LoG-sigma-10-0-mm-3D_glrlm_ShortRunEmphasis	FALSE	7.68595E-05	0.122590838	1246	0.816684502	1	2125	0.496690774	1	2142	0.097842819	1	1875	0.077556634	1	1845	0.194534747	1	1973
		LoG-sigma-10-0-mm-3D_glrlm_ShortRunHighGrayLevelEmphasis	TRUE	0.320233409	1	2056	0.067311891	1	1708	1.78524E-05	0.02847451	1154	0.049296391	1	1790	3.5179E-09	5.61106E-06	712	0.000470212	0.749988905	1371
		LoG-sigma-10-0-mm-3D_glrlm_ShortRunLowGrayLevelEmphasis	TRUE	5.9664E-09	9.51641E-06	736	0.244874705	1	1882	2.38748E-11	3.80803E-08	500	0.097842819	1	1875	1.03367E-12	1.6487E-09	378	0.031080278	1	1738
		LoG-sigma-10-0-mm-3D_glszm_GrayLevelNonUniformity	TRUE	0.398585486	1	1444	0.557742686	1	204	0.695787227	1	2221	1.1872E-06	0.001893576	1006	0.520118689	1	2152	1.68497E-06	0.002687524	1025
		LoG-sigma-10-0-mm-3D_glszm_GrayLevelNonUniformityNormalized	FALSE	0.596331165	1	1548	0.359536809	1	215	0.798102763	1	2258	0.000865047	1	1417	0.698490298	1	2222	0.000238027	0.379652619	1323
		LoG-sigma-10-0-mm-3D_glszm_GrayLevelVariance	FALSE	0.007182829	1	1120	0.640161151	1	239	0.017708302	1	1679	0.965004929	1	2316	0.006198443	1	1579	0.455754335	1	2124
		LoG-sigma-10-0-mm-3D_glszm_HighGrayLevelZoneEmphasis	TRUE	0.127692971	1	1372	0.925510561	1	259	0.000110507	0.176257878	1270	0.02959913	1	1733	5.37369E-06	0.00857103	1083	0.001152736	1	1439

	GLSZM	LoG-sigma-10-0-mm-3D_glszm_LargeAreaEmphasis	TRUE	0.311418531	1	2051	0.026717063	1	1570	0.00105931	1	1432	4.87292E-08	7.77231E-05	835	0.00056876	0.907357532	1385	6.23408E-09	9.94336E-06	738
		LoG-sigma-10-0-mm-3D_glszm_LargeAreaHighGrayLevelEmphasis	TRUE	0.857888803	1	2279	0.033823178	1	1594	0.006198443	1	1579	1.78544E-05	0.028477724	1154	0.000476659	0.760270802	1372	3.18124E-07	0.000507407	929
		LoG-sigma-10-0-mm-3D_glszm_LargeAreaLowGrayLevelEmphasis	TRUE	0.632139605	1	2197	0.498064709	1	1965	0.970849136	1	2318	6.38948E-06	0.010191214	1093	0.880900619	1	2287	3.71233E-07	0.000592117	937
		LoG-sigma-10-0-mm-3D_glszm_LowGrayLevelZoneEmphasis	TRUE	0.699484433	1	1618	0.955264678	1	261	0.155161225	1	1939	0.755973695	1	2243	0.126634033	1	1910	2.11597E-06	0.003374978	1031
		LoG-sigma-10-0-mm-3D_glszm_SizeZoneNonUniformity	TRUE	0.273514391	1	1985	0.190856837	1	124	0.834997126	1	2271	2.94913E-05	0.047038701	1218	0.789649143	1	2255	3.71264E-05	0.059216651	1232
		LoG-sigma-10-0-mm-3D_glszm_SizeZoneNonUniformityNormalized	FALSE	0.167334306	1	1908	0.152865349	1	200.5	1	1	2328	0.003563388	1	1531	0.956285864	1	2313	0.02144279	1	1699
		LoG-sigma-10-0-mm-3D_glszm_SmallAreaEmphasis	FALSE	0.169432713	1	1952	0.193408652	1	1805	0.772816901	1	2249	0.840708665	1	2273	0.73947835	1	2237	0.820760245	1	2266
		LoG-sigma-10-0-mm-3D_glszm_SmallAreaHighGrayLevelEmphasis	FALSE	0.427783603	1	2111	0.50799535	1	1969	0.417215377	1	2106	0.000546269	0.871299676	1382	0.38644809	1	2091	0.000668547	1	1397
		LoG-sigma-10-0-mm-3D_glszm_SmallAreaLowGrayLevelEmphasis	FALSE	0.295958732	1	2042	0.0461876	1	1627	0.00755543	1	1597	0.110280459	1	1891	0.007154014	1	1592	0.130308955	1	1914
		LoG-sigma-10-0-mm-3D_glszm_ZoneEntropy	FALSE	0.092196032	1	1629	0.758269421	1	279.5	0.676975497	1	2214	4.07887E-05	0.065057929	1206	0.817918548	1	2265	0.029569062	1	1733
		LoG-sigma-10-0-mm-3D_glszm_ZonePercentage	FALSE	0.483012353	1	1189	0.616391311	1	181	0.898226825	1	2293	0.000361633	0.576804165	1352	0.76722939	1	2247	0.001673931	1	1468
		LoG-sigma-10-0-mm-3D_glszm_ZoneVariance	TRUE	0.274549558	1	2029	0.065502523	1	1666	0.000561232	0.895164319	1384	3.78772E-06	0.006041406	1063	0.00023668	0.377504633	1322	8.45166E-09	1.34804E-05	752
	NGTDM	LoG-sigma-10-0-mm-3D_ngtdm_Busyness	TRUE	3.25322E-05	0.051888885	1191	0.075742223	1	1722	0.000404097	0.644534829	1360	4.2933E-09	6.84781E-06	721	0.000117317	0.187120481	1274	3.85386E-10	6.14691E-07	615
		LoG-sigma-10-0-mm-3D_ngtdm_Coarseness	TRUE	0.223645078	1	1995	0.720153252	1	2092	0.008329143	1	1606	0.030234373	1	1735	6.75952E-08	0.000107814	851	0.012694024	1	1646
		LoG-sigma-10-0-mm-3D_ngtdm_Complexity	TRUE	2.42282E-07	0.00038644	915	0.00805998	1	1494	3.96371E-08	6.32211E-05	825	8.9964E-06	0.014349265	1113	3.1385E-11	5.00591E-08	511	1.17483E-10	1.87385E-07	565
		LoG-sigma-10-0-mm-3D_ngtdm_Contrast	TRUE	8.28059E-08	0.000132075	861	0.055635375	1	1686	1.41991E-12	2.26475E-09	390	5.75994E-07	0.00091871	960	2.16182E-12	3.44811E-09	406	5.98185E-08	9.54105E-05	845
		LoG-sigma-10-0-mm-3D_ngtdm_Strength	TRUE	0.000954804	1	1424	0.870642272	1	2143	1.03367E-12	1.6487E-09	378	0.904014002	1	2295	1.03367E-12	1.6487E-09	378	0.728471475	1	2233
Wavelet Coiflet 3 - LLH	First Order	Wavelet_Coif3-LLH_firstorder_10Percentile	FALSE	1.78053E-17	2.83995E-14	0	3.89964E-17	6.21992E-14	25	0.116949178	1	1899	0.006697982	1	1586	0.026792665	1	1722	0.000361633	0.576804165	1352
		Wavelet_Coif3-LLH_firstorder_90Percentile	FALSE	1.78053E-17	2.83995E-14	0	2.51712E-17	4.0148E-14	11	0.094913468	1	1871	0.005065222	1	1561	0.026542589	1	1721	0.000202054	0.322276633	1311
		Wavelet_Coif3-LLH_firstorder_Energy	FALSE	1.05002E-16	1.67478E-13	57	5.01474E-14	7.99851E-11	267	0.985422135	1	2323	0.001458056	1	1457	0.163831404	1	1947	0.038608071	1	1762
		Wavelet_Coif3-LLH_firstorder_Entropy	FALSE	1.78053E-17	2.83995E-14	0	3.44206E-17	5.49009E-14	21	0.099331372	1	1877	0.003627824	1	1532	0.107057616	1	1887	0.003263947	1	1523
		Wavelet_Coif3-LLH_firstorder_InterquartileRange	FALSE	1.78053E-17	2.83995E-14	0	1.8383E-17	2.93208E-14	1	0.078792819	1	1847	0.003341808	1	1525	0.014204221	1	1657	3.75495E-05	0.059891473	1200
		Wavelet_Coif3-LLH_firstorder_Kurtosis	TRUE	1.83751E-17	2.93083E-14	1	2.25687E-16	3.59971E-13	82	5.64753E-05	0.090078028	1226	4.97416E-08	7.93378E-05	836	9.46488E-06	0.015096488	1116	1.23805E-07	0.000197468	881
		Wavelet_Coif3-LLH_firstorder_Maximum	FALSE	1.30931E-06	0.002088346	1004	0.005181184	1	1563	0.693088015	1	2220	0.026543473	1	1721	0.823602236	1	2267	0.04641503	1	1783
		Wavelet_Coif3-LLH_firstorder_MeanAbsoluteDeviation	FALSE	1.78053E-17	2.83995E-14	0	3.23356E-17	5.15753E-14	19	0.098583866	1	1876	0.007638449	1	1598	0.09929806	1	1764	0.00060862	0.970749095	1390
		Wavelet_Coif3-LLH_firstorder_Mean	FALSE	0.046413752	1	1783	0.095640592	1	1872	0.728469951	1	2233	4.00059E-05	0.063809473	1204	0.988337474	1	2324	0.000297155	0.473962088	1338
		Wavelet_Coif3-LLH_firstorder_Median	FALSE	0.055976865	1	1805	0.736722621	1	2236	0.843566224	1	2274	0.515390402	1	2150	0.73947835	1	2237	0.671638705	1	2212
		Wavelet_Coif3-LLH_firstorder_Minimum	FALSE	2.8066E-12	4.47653E-09	416	0.666316646	1	2210	0.676975497	1	2214	0.717518853	1	2229	0.398585183	1	2097	0.368673722	1	2082
		Wavelet_Coif3-LLH_firstorder_Range	FALSE	9.22524E-11	1.47143E-07	555	0.067676407	1	1828	0.591138569	1	2181	0.025805283	1	1718	0.786836709	1	2254	0.056449371	1	1806
		Wavelet_Coif3-LLH_firstorder_RobustMeanAbsoluteDeviation	FALSE	1.78053E-17	2.83995E-14	0	2.02033E-17	3.22243E-14	4	0.080677108	1	1850	0.002196341	1	1490	0.016354974	1	1671	3.75495E-05	0.059891473	1200
		Wavelet_Coif3-LLH_firstorder_RootMeanSquared	FALSE	5.32056E-17	8.48629E-14	35	1.49496E-15	2.38446E-12	145	0.178691692	1	1960	0.031080278	1	1738	0.152001291	1	1936	0.020914498	1	1696
		Wavelet_Coif3-LLH_firstorder_Skewness	FALSE	0.021529089	1	1699	0.034984537	1	1751	0.161630372	1	1945	0.653085267	1	2205	0.485191301	1	2137	0.982507093	1	2322
		Wavelet_Coif3-LLH_firstorder_TotalEnergy	FALSE	1.05002E-16	1.67478E-13	57	5.01474E-14	7.99851E-11	267	0.988337474	1	2324	0.001458056	1	1457	0.163831404	1	1947	0.038608071	1	1762
		Wavelet_Coif3-LLH_firstorder_Uniformity	FALSE	1.78053E-17	2.83995E-14	0	2.29096E-17	3.65408E-14	8	0.066583553	1	1826	0.000724232	1	1403	0.154102418	1	1938	0.003845563	1	1537
		Wavelet_Coif3-LLH_firstorder_Variance	FALSE	1.30183E-16	2.07642E-13	64	3.3082E-15	5.27658E-12	172	0.370620659	1	2083	0.185881769	1	1966	0.277772578	1	2031	0.141823853	1	1926
	GLCM	Wavelet_Coif3-LLH_glcm_Autocorrelation	TRUE	1.0926E-10	1.74269E-07	562	0.309767768	1	2050	4.21739E-08	6.72674E-05	828	4.47629E-12	7.13969E-09	434	9.79084E-06	0.015616384	1118	1.8969E-10	3.02556E-07	585
		Wavelet_Coif3-LLH_glcm_ClusterProminence	TRUE	5.37369E-06	0.00857103	1083	2.84206E-08	4.53308E-05	809	8.05277E-14	1.28442E-10	284	5.58796E-15	8.9128E-12	190	8.27946E-14	1.32057E-10	285	5.58796E-15	8.9128E-12	190
		Wavelet_Coif3-LLH_glcm_ClusterShade	TRUE	8.99532E-06	0.014347536	1113	0.193280825	1	1972	0.904013444	1	2295	4.05833E-15	6.47303E-12	179	2.97514E-10	4.74535E-07	604	5.58796E-15	8.9128E-12	190
		Wavelet_Coif3-LLH_glcm_ClusterTendency	FALSE	1.38413E-16	2.20769E-13	66	1.14916E-14	1.8329E-11	215	0.368670925	1	2082	0.147866935	1	1932	0.297650666	1	2043	0.1110975	1	1892
		Wavelet_Coif3-LLH_glcm_Contrast	FALSE	6.60994E-17	1.05429E-13	42	3.61131E-15	5.76003E-12	175	0.410949003	1	2103	0.120403523	1	1903	0.271352173	1	2027	0.10160305	1	1880
		Wavelet_Coif3-LLH_glcm_Correlation	FALSE	2.01947E-17	3.22105E-14	4	1.28911E-15	2.05613E-12	140	0.023922148	1	1710	0.00731257	1	1594	0.004218516	1	1545	0.000616864	0.983897489	1391
		Wavelet_Coif3-LLH_glcm_DifferenceAverage	FALSE	1.78053E-17	2.83995E-14	0	3.89964E-17	6.21992E-14	25	0.10547542	1	1885	0.006922791	1	1589	0.024847851	1	1714	0.000387674	0.618339447	1357
		Wavelet_Coif3-LLH_glcm_DifferenceEntropy	FALSE	1.78053E-17	2.83995E-14	0	6.21556E-17	9.91381E-14	40	0.118665424	1	1901	0.006551778	1	1584	0.097102332	1	1874	0.003004275	1	1516
		Wavelet_Coif3-LLH_glcm_DifferenceVariance	FALSE	1.69622E-13	2.70548E-10	311	1.81379E-11	2.89299E-08	489	0.378483438	1	2087	0.167177676	1	1950	0.394514211	1	2095	0.251194202	1	2014
		Wavelet_Coif3-LLH_glcm_Id	FALSE	1.78053E-17	2.83995E-14	0	2.15145E-17	3.43156E-14	6	0.087223821	1	1860	0.001942697	1	1480	0.050575079	1	1793	0.000476695	0.760328922	1372
		Wavelet_Coif3-LLH_glcm_Idm	FALSE	1.78053E-17	2.83995E-14	0	2.15145E-17	3.43156E-14	6	0.090654403	1	1865	0.002362747	1	1496	0.048874479	1	1789	0.000489912	0.781409076	1374
		Wavelet_Coif3-LLH_glcm_Idmn	FALSE	3.4496E-16	5.50211E-13	96	2.6649E-12	4.25051E-09	414	0.234968196	1	2003	0.199610135	1	1977	0.040356591	1	1767	0.049296391	1	1790
		Wavelet_Coif3-LLH_glcm_Idn	FALSE	3.4496E-16	5.50211E-13	96	3.8284E-15	6.10629E-12	177	0.053218743	1	1799	0.074536883	1	1840	0.011209263	1	1634	0.01479227	1	1661
		Wavelet_Coif3-LLH_glcm_Imc1	FALSE	1.89283E-15	3.01906E-12	153	4.64652E-10	7.4112E-07	623	0.141821526	1	1926	0.02860177	1	1729	0.464860741	1	2128	0.035942758	1	1754
		Wavelet_Coif3-LLH_glcm_Imc2	FALSE	0.091353375	1	1866	0.000113874	0.181629346	1272	0.886670109	1	2289	0.527258961	1	2155	0.846426763	1	2275	0.529649296	1	2156
		Wavelet_Coif3-LLH_glcm_InverseVariance	FALSE	1.87909E-16	2.99714E-13	76	2.93927E-14	4.68814E-11	248	0.546532102	1	2163	0.357109779	1	2076	0.010749947	1	1630	0.01156559	1	1637
		Wavelet_Coif3-LLH_glcm_JointAverage	FALSE	3.59199E-06	0.005729228	1060	0.65836503	1	2207	0.394514211	1	2095	0.01479227	1	1661	0.195792016	1	1974	0.010750406	1	1630
		Wavelet_Coif3-LLH_glcm_JointEnergy	FALSE	1.78053E-17	2.83995E-14	0	2.15145E-17	3.43156E-14	6	0.031079295	1	1738	0.000110517	0.176274196	1270	0.281021266	1	2033	0.018241228	1	1682
		Wavelet_Coif3-LLH_glcm_JointEntropy	FALSE	1.78053E-17	2.83995E-14	0	4.0231E-17	6.41685E-14	26	0.091353375	1	1866	0.003627824	1	1532	0.108658557	1	1889	0.002968756	1	1515
		Wavelet_Coif3-LLH_glcm_MCC	FALSE	0.001421586	1	1455	0.860759904	1	2280	0.834997126	1	2271	0.432056844	1	2113	0.733967315	1	2235	0.442840596	1	2118
		Wavelet_Coif3-LLH_glcm_MaximumProbability	FALSE	1.78053E-17	2.83995E-14	0	2.22012E-17	3.5411E-14	7	0.048874479	1	1789	0.000695872	1	1400	0.13123767	1	1915	0.003076473	1	1518
		Wavelet_Coif3-LLH_glcm_SumAverage	FALSE	3.59199E-06	0.005729228	1060	0.65836503	1	2207	0.394514211	1	2095	0.01479227	1	1661	0.195792016	1	1974	0.010750406	1	1630
		Wavelet_Coif3-LLH_glcm_SumEntropy	FALSE	1.78053E-17	2.83995E-14	0	5.16003E-17	8.23025E-14	34	0.107855735	1	1888	0.005299541	1	1565	0.133115054	1	1917	0.005995238	1	1576
		Wavelet_Coif3-LLH_glcm_SumSquares	FALSE	8.46364E-17	1.34995E-13	50	6.09561E-15	9.72249E-12	193	0.408872696	1	2102	0.132176039	1	1916	0.297650666	1	2043	0.099333347	1	1877
	GLDM	Wavelet_Coif3-LLH_gldm_DependenceEntropy	FALSE	1.17833E-15	1.88024E-12	137	1.85984E-11	2.96644E-08	490	0.31668807	1	2054	0.113577317	1	1895	0.632139605	1	2197	0.268183186	1	2025
		Wavelet_Coif3-LLH_gldm_DependenceNonUniformity	FALSE	5.4471E-13	8.68812E-10	354	5.00827E-09	7.98818E-06	728	0.544103696	1	2162	7.92504E-05	0.126404388	1248	0.548965864	1	2164	5.56098E-05	0.088697702	1225
		Wavelet_Coif3-LLH_gldm_DependenceNonUniformityNormalized	FALSE	4.42732E-15	7.06157E-12	182	4.48307E-14	7.1505E-11	263	0.053218743	1	1799	0.089961617	1	1864	0.000546228	0.871234376	1382	0.001716207	1	1470
		Wavelet_Coif3-LLH_gldm_DependenceVariance	FALSE	3.54973E-17	5.66183E-14	22	3.8943E-16	6.2114E-13	100	0.052324979	1	1797	0.034670015	1	1750	0.004571654	1	1552	0.002250576	1	1492
		Wavelet_Coif3-LLH_gldm_GrayLevelNonUniformity	TRUE	1.78053E-17	2.83995E-14	0	1.78129E-17	2.84116E-14	0	0.017019838	1	1675	3.15471E-08	5.03176E-05	814	0.143814428	1	1928	1.44612E-08	2.30656E-05	777
		Wavelet_Coif3-LLH_gldm_GrayLevelVariance	FALSE	1.56442E-16	2.49525E-13	70	3.71829E-15	5.93068E-12	176	0.378483438	1	2087	0.17869423	1	1960	0.279393712	1	2032	0.136934407	1	1921
		Wavelet_Coif3-LLH_gldm_HighGrayLevelEmphasis	TRUE	0.000677493	1	1398	0.008329522	1	1606	4.3054E-08	6.86711E-05	829	4.25106E-12	6.78044E-09	432	9.46488E-06	0.015096488	1116	1.8969E-10	3.02556E-07	585
		Wavelet_Coif3-LLH_gldm_LargeDependenceEmphasis	FALSE	1.78053E-17	2.83995E-14	0	2.15145E-17	3.43156E-14	6	0.041438317	1	1770	0.000117328	0.18713768	1274	0.143814428	1	1928	0.004731276	1	1555
		Wavelet_Coif3-LLH_gldm_LargeDependenceHighGrayLevelEmphasis	TRUE	6.21298E-17	9.90971E-14	40	1.24463E-12	1.98519E-09	385	0.002223174	1	1491	0.000102527	0.163530603	1265	5.28114E-06	0.00842342	1082	8.87607E-07	0.001415733	983
		Wavelet_Coif3-LLH_gldm_LargeDependenceLowGrayLevelEmphasis	FALSE	4.22201E-11	6.7341E-08	523	1.15241E-11	1.8381E-08	471	0.226438582	1	1997	0.078794567	1	1847	0.434198658	1	2114	0.489776251	1	2139
		Wavelet_Coif3-LLH_gldm_LowGrayLevelEmphasis	FALSE	1.39959E-13	2.23235E-10	304	0.014349694	1	1658	0.38644809	1	2091	0.197060479	1	1975	0.442837931	1	2118	0.168303599	1	1951
		Wavelet_Coif3-LLH_gldm_SmallDependenceEmphasis	FALSE	1.89629E-17	3.02458E-14	2	1.30236E-16	2.07727E-13	64	0.172861744	1	1955	0.053220134	1	1799	0.035300421	1	1752	0.004951611	1	1559
		Wavelet_Coif3-LLH_gldm_SmallDependenceHighGrayLevelEmphasis	TRUE	4.00018E-05	0.063802846	1204	1.82693E-08	2.91396E-05	788	6.82644E-07	0.001088817	969	2.05214E-12	3.27317E-09	404	2.02782E-06	0.003234374	1028	9.54808E-13	1.52292E-09	375
		Wavelet_Coif3-LLH_gldm_SmallDependenceLowGrayLevelEmphasis	FALSE	4.83105E-15	7.70553E-12	185	9.46602E-06	0.015098299	1116	0.784027053	1	2253	0.073944279	1	1839	0.183459991	1	1964	0.013091258	1	1649
	GLRLM	Wavelet_Coif3-LLH_glrlm_GrayLevelNonUniformity	TRUE	1.78053E-17	2.83995E-14	0	1.78129E-17	2.84116E-14	0	0.050575079	1	1793	3.468E-05	0.055314644	1195	0.068780721	1	1830	6.10533E-08	9.738E-05	846
		Wavelet_Coif3-LLH_glrlm_GrayLevelNonUniformityNormalized	FALSE	1.78053E-17	2.83995E-14	0	2.29096E-17	3.65408E-14	8	0.076336294	1	1843	0.000763718	1	1407	0.230675369	1	2000	0.011209738	1	1634
		Wavelet_Coif3-LLH_glrlm_GrayLevelVariance	FALSE	9.95142E-15	1.58725E-11	210	3.83911E-13	6.12338E-10	341	0.427783603	1	2111	0.299315875	1	2044	0.442837931	1	2118	0.429918494	1	2112
		Wavelet_Coif3-LLH_glrlm_HighGrayLevelRunEmphasis	TRUE	0.000524423	0.836454623	1379	0.01479227	1	1661	4.39518E-08	7.01032E-05	830	4.25106E-12	6.78044E-09	432	9.79084E-06	0.015616384	1118	1.8969E-10	3.02556E-07	585
		Wavelet_Coif3-LLH_glrlm_LongRunEmphasis	FALSE	1.78053E-17	2.83995E-14	0	2.15145E-17	3.43156E-14	6	0.023247641	1	1707	5.22512E-05	0.083340727	1221	0.281021266	1	2033	0.040002541	1	1766
		Wavelet_Coif3-LLH_glrlm_LongRunHighGrayLevelEmphasis	TRUE	1.99725E-16	3.18561E-13	78	7.9534E-08	0.000126857	859	1.85231E-06	0.002954441	1023	1.00455E-08	1.60225E-05	760	1.26201E-06	0.002012912	1002	6.49076E-08	0.000103528	849
		Wavelet_Coif3-LLH_glrlm_LongRunLowGrayLevelEmphasis	FALSE	2.47465E-05	0.039470668	1174	1.21883E-08	1.94404E-05	769	0.24818505	1	2012	0.368673722	1	2082	0.311418531	1	2051	0.693089717	1	2220
		Wavelet_Coif3-LLH_glrlm_LowGrayLevelRunEmphasis	FALSE	2.55523E-13	4.07559E-10	326	0.027814338	1	1726	0.374539291	1	2085	0.229259611	1	1999	0.415120353	1	2105	0.204781577	1	1981
		Wavelet_Coif3-LLH_glrlm_RunEntropy	FALSE	0.008419345	1	1607	2.8874E-07	0.00046054	924	0.717517273	1	2229	0.736722621	1	2236	0.750540389	1	2241	0.701198859	1	2223
		Wavelet_Coif3-LLH_glrlm_RunLengthNonUniformity	TRUE	1.78053E-17	2.83995E-14	0	6.82051E-17	1.08787E-13	43	0.875137443	1	2285	0.000202054	0.322276633	1311	0.116098395	1	1898	6.8951E-10	1.09977E-06	640
		Wavelet_Coif3-LLH_glrlm_RunLengthNonUniformityNormalized	FALSE	1.78053E-17	2.83995E-14	0	2.15145E-17	3.43156E-14	6	0.05692229	1	1807	0.000332544	0.530407091	1346	0.107855735	1	1888	0.000871343	1	1417
		Wavelet_Coif3-LLH_glrlm_RunPercentage	FALSE	1.78053E-17	2.83995E-14	0	2.15145E-17	3.43156E-14	6	0.046413752	1	1783	0.000169719	0.270702329	1299	0.121276744	1	1904	0.001236125	1	1444
		Wavelet_Coif3-LLH_glrlm_RunVariance	FALSE	1.78053E-17	2.83995E-14	0	2.02033E-17	3.22243E-14	4	0.018786752	1	1685	6.29501E-05	0.10040539	1233	0.299349052	1	2044	0.038265979	1	1761
		Wavelet_Coif3-LLH_glrlm_ShortRunEmphasis	FALSE	1.78053E-17	2.83995E-14	0	2.22012E-17	3.5411E-14	7	0.047630759	1	1786	0.000130209	0.20768297	1281	0.19453214	1	1973	0.004840309	1	1557
		Wavelet_Coif3-LLH_glrlm_ShortRunHighGrayLevelEmphasis	TRUE	0.003150075	1	1520	0.001300844	1	1448	1.05461E-07	0.000168211	873	8.35877E-13	1.33322E-09	370	7.45692E-06	0.011893788	1102	4.71321E-12	7.51757E-09	436
		Wavelet_Coif3-LLH_glrlm_ShortRunLowGrayLevelEmphasis	FALSE	5.76521E-14	9.19552E-11	272	0.003935934	1	1539	0.695787227	1	2221	0.095640592	1	1872	0.772816901	1	2249	0.06712895	1	1827
	GLSZM	Wavelet_Coif3-LLH_glszm_GrayLevelNonUniformity	FALSE	0.012434652	1	1644	0.429918494	1	2112	0.866505176	1	2282	0.000240112	0.3829782	1323	0.857888803	1	2279	3.69582E-05	0.058948276	1199
		Wavelet_Coif3-LLH_glszm_GrayLevelNonUniformityNormalized	FALSE	6.73603E-10	1.0744E-06	639	3.26377E-13	5.20571E-10	335	0.800925959	1	2259	0.202183805	1	1979	0.863631236	1	2281	0.603822015	1	2186
		Wavelet_Coif3-LLH_glszm_GrayLevelVariance	FALSE	1.47699E-08	2.3558E-05	778	1.98942E-10	3.17313E-07	587	0.476096548	1	2133	0.471587042	1	2131	0.661009477	1	2208	0.846427649	1	2275
		Wavelet_Coif3-LLH_glszm_HighGrayLevelZoneEmphasis	TRUE	5.73591E-05	0.091487836	1227	0.274552362	1	2029	7.48271E-08	0.000119349	856	4.96241E-12	7.91505E-09	438	9.46488E-06	0.015096488	1116	9.22756E-11	1.4718E-07	555
		Wavelet_Coif3-LLH_glszm_LargeAreaEmphasis	TRUE	1.78053E-17	2.83995E-14	0	2.15145E-17	3.43156E-14	6	0.00051034	0.813991712	1377	2.30375E-09	3.67448E-06	693	0.222257606	1	1994	1.16768E-08	1.86245E-05	767
		Wavelet_Coif3-LLH_glszm_LargeAreaHighGrayLevelEmphasis	TRUE	2.08398E-17	3.32395E-14	5	7.6819E-15	1.22526E-11	201	2.8016E-06	0.004468549	1046	8.81591E-13	1.40614E-09	372	3.22053E-08	5.13674E-05	815	6.58354E-10	1.05007E-06	638
		Wavelet_Coif3-LLH_glszm_LargeAreaLowGrayLevelEmphasis	TRUE	9.64688E-12	1.53868E-08	464	6.72798E-11	1.07311E-07	542	0.973763113	1	2319	7.78713E-07	0.001242047	976	0.473837548	1	2132	7.98567E-06	0.012737139	1106
		Wavelet_Coif3-LLH_glszm_LowGrayLevelZoneEmphasis	FALSE	4.89845E-09	7.81302E-06	727	0.055045973	1	1803	0.661009477	1	2208	0.892446236	1	2291	0.745002796	1	2239	0.921403154	1	2301
		Wavelet_Coif3-LLH_glszm_SizeZoneNonUniformity	TRUE	4.66468E-16	7.44016E-13	106	6.57331E-13	1.04844E-09	361	0.265034171	1	2023	0.128462402	1	1912	0.013090726	1	1649	8.79975E-08	0.000140356	864
		Wavelet_Coif3-LLH_glszm_SizeZoneNonUniformityNormalized	FALSE	9.22524E-11	1.47143E-07	555	2.30088E-06	0.003669909	1035	0.79246432	1	2256	0.32560271	1	2059	0.663660038	1	2209	0.139852221	1	1924
		Wavelet_Coif3-LLH_glszm_SmallAreaEmphasis	FALSE	1.35683E-10	2.16415E-07	571	2.0281E-06	0.003234818	1028	0.653083373	1	2205	0.299351875	1	2044	0.614052096	1	2190	0.19833231	1	1976
		Wavelet_Coif3-LLH_glszm_SmallAreaHighGrayLevelEmphasis	TRUE	0.006062098	1	1577	0.027045669	1	1723	7.35982E-07	0.001173891	973	1.72501E-11	2.75139E-08	487	1.69915E-05	0.027101409	1151	5.53319E-11	8.82544E-08	534
		Wavelet_Coif3-LLH_glszm_SmallAreaLowGrayLevelEmphasis	FALSE	6.23282E-09	9.94134E-06	738	0.024382025	1	1712	0.334673206	1	2064	0.394516973	1	2095	0.207400881	1	1983	0.219503871	1	1992
		Wavelet_Coif3-LLH_glszm_ZoneEntropy	FALSE	0.001781354	1	1473	0.225041443	1	1996	0.880900619	1	2287	0.786837925	1	2254	0.921402696	1	2301	0.892446236	1	2291
		Wavelet_Coif3-LLH_glszm_ZonePercentage	FALSE	2.65134E-15	4.22889E-12	150	3.40634E-15	5.43312E-12	173	0.823602236	1	2267	0.380467848	1	2088	0.314925095	1	2053	0.099333347	1	1877
		Wavelet_Coif3-LLH_glszm_ZoneVariance	TRUE	1.78053E-17	2.83995E-14	0	2.08487E-17	3.32537E-14	5	0.00051034	0.813991712	1377	2.40932E-09	3.84286E-06	695	0.220876309	1	1993	1.16768E-08	1.86245E-05	767
	NGTDM	Wavelet_Coif3-LLH_ngtdm_Busyness	FALSE	1.57252E-06	0.002508163	1014	0.800927098	1	2259	0.99125297	1	2325	0.025805283	1	1718	0.933016965	1	2305	0.197060479	1	1975
		Wavelet_Coif3-LLH_ngtdm_Coarseness	FALSE	5.4746E-05	0.087319916	1224	0.005481657	1	1568	0.043675548	1	1776	0.222260307	1	1994	0.005928461	1	1575	0.024151552	1	1711
		Wavelet_Coif3-LLH_ngtdm_Complexity	TRUE	9.365E-05	0.149371706	1259	5.00827E-09	7.98818E-06	728	0.434198658	1	2114	2.90828E-05	0.046387135	1184	0.994168581	1	2326	1.61721E-05	0.025794491	1148
		Wavelet_Coif3-LLH_ngtdm_Contrast	FALSE	2.47144E-16	3.94194E-13	85	3.03018E-15	4.83314E-12	169	0.536850824	1	2159	0.134063782	1	1918	0.058853017	1	1811	0.001737711	1	1471
		Wavelet_Coif3-LLH_ngtdm_Strength	FALSE	2.0544E-13	3.27677E-10	318	0.07046905	1	1833	0.167175196	1	1950	0.021738444	1	1700	0.000314358	0.501400936	1342	0.007154355	1	1592
Wavelet Coiflet 3 - LHL	First Order	Wavelet_Coif3-LHL_firstorder_10Percentile	FALSE	2.52172E-10	4.02212E-07	597	1.71951E-09	2.74262E-06	680	0.458190688	1	2125	0.222260307	1	1994	0.539263037	1	2160	0.261916264	1	2021
		Wavelet_Coif3-LHL_firstorder_90Percentile	FALSE	1.52963E-10	2.43977E-07	576	6.28573E-10	1.00257E-06	636	0.480632205	1	2135	0.171715519	1	1954	0.603819901	1	2186	0.214064797	1	1988
		Wavelet_Coif3-LHL_firstorder_Energy	FALSE	0.069901448	1	1832	0.002250576	1	1492	0.772816901	1	2249	0.000517376	0.825214263	1378	0.745002796	1	2239	0.000724232	1	1403
		Wavelet_Coif3-LHL_firstorder_Entropy	FALSE	7.98469E-06	0.012735587	1106	1.76991E-07	0.000282301	899	0.546532102	1	2163	0.281024077	1	2033	0.690392694	1	2219	0.396549303	1	2096
		Wavelet_Coif3-LHL_firstorder_InterquartileRange	FALSE	5.78843E-12	9.23255E-09	444	8.71614E-12	1.39022E-08	460	0.445013007	1	2119	0.160540707	1	1944	0.616622038	1	2191	0.204781577	1	1981
		Wavelet_Coif3-LHL_firstorder_Kurtosis	FALSE	1.78053E-17	2.83995E-14	0	1.78129E-17	2.84116E-14	0	0.018971971	1	1686	0.676977277	1	2214	0.001826115	1	1475	0.04641503	1	1783
		Wavelet_Coif3-LHL_firstorder_Maximum	FALSE	2.77417E-15	4.4248E-12	166	2.11821E-08	3.37855E-05	795	0.266604087	1	2024	0.501333418	1	2144	0.94173717	1	2308	0.666316646	1	2210
		Wavelet_Coif3-LHL_firstorder_MeanAbsoluteDeviation	FALSE	6.10425E-08	9.73627E-05	846	2.50643E-08	3.99775E-05	803	0.501330917	1	2144	0.233533745	1	2002	0.583589032	1	2178	0.30276801	1	2046
		Wavelet_Coif3-LHL_firstorder_Mean	FALSE	0.869380882	1	2283	0.153051512	1	1937	0.154102418	1	1938	0.982507093	1	2322	0.184666663	1	1965	0.927208316	1	2303
		Wavelet_Coif3-LHL_firstorder_Median	FALSE	7.33233E-08	0.000116951	855	0.292597031	1	2040	0.837851369	1	2272	0.947554895	1	2310	0.809411021	1	2262	0.915602177	1	2299
		Wavelet_Coif3-LHL_firstorder_Minimum	FALSE	3.66459E-16	5.84502E-13	98	2.94561E-09	4.69825E-06	704	0.002391437	1	1497	0.02347143	1	1708	0.094192694	1	1870	0.130308955	1	1914
		Wavelet_Coif3-LHL_firstorder_Range	FALSE	6.11337E-16	9.75082E-13	115	2.25267E-09	3.59301E-06	692	0.02091375	1	1696	0.258821048	1	2019	0.239317282	1	2006	0.336510063	1	2065
		Wavelet_Coif3-LHL_firstorder_RobustMeanAbsoluteDeviation	FALSE	8.71372E-12	1.38984E-08	460	9.89779E-12	1.5787E-08	465	0.455979325	1	2124	0.135972309	1	1920	0.598732698	1	2184	0.193280825	1	1972
		Wavelet_Coif3-LHL_firstorder_RootMeanSquared	FALSE	0.05692229	1	1807	0.000398573	0.635723176	1359	0.829295192	1	2269	0.46486335	1	2128	0.866505176	1	2282	0.671638705	1	2212
		Wavelet_Coif3-LHL_firstorder_Skewness	FALSE	1.95287E-07	0.000311482	904	0.007891338	1	1601	7.80425E-05	0.124477811	1247	0.685015585	1	2217	0.002968588	1	1515	0.626950619	1	2195
		Wavelet_Coif3-LHL_firstorder_TotalEnergy	FALSE	0.069901448	1	1832	0.002250576	1	1492	0.770021653	1	2248	0.00053164	0.847965276	1380	0.745002796	1	2239	0.000743732	1	1405
		Wavelet_Coif3-LHL_firstorder_Uniformity	FALSE	8.37266E-11	1.33544E-07	551	4.98306E-10	7.94799E-07	626	0.553849376	1	2166	0.174018724	1	1956	0.685013843	1	2217	0.263473425	1	2022
		Wavelet_Coif3-LHL_firstorder_Variance	FALSE	0.100083393	1	1878	0.002091446	1	1486	0.938829607	1	2307	0.817919593	1	2265	0.909805166	1	2297	0.973763266	1	2319
	GLCM	Wavelet_Coif3-LHL_glcm_Autocorrelation	TRUE	2.25869E-15	3.60261E-12	159	1.12995E-06	0.001802267	996	3.63719E-05	0.058013104	1198	1.39291E-05	0.022216942	1139	0.429915799	1	2112	0.00731257	1	1594
		Wavelet_Coif3-LHL_glcm_ClusterProminence	TRUE	1.38346E-06	0.002206619	1007	0.632141595	1	2197	1.03336E-13	1.64822E-10	293	5.58796E-15	8.9128E-12	190	6.2287E-13	9.93478E-10	359	3.17649E-13	5.0665E-10	334
		Wavelet_Coif3-LHL_glcm_ClusterShade	FALSE	1.24498E-08	1.98574E-05	770	0.005240061	1	1564	0.027044774	1	1723	0.163833866	1	1947	4.75668E-05	0.075869003	1215	0.956286119	1	2313
		Wavelet_Coif3-LHL_glcm_ClusterTendency	FALSE	0.055044553	1	1803	0.000815885	1	1412	0.959197507	1	2314	0.852154733	1	2277	0.950464366	1	2311	0.970849306	1	2318
		Wavelet_Coif3-LHL_glcm_Contrast	FALSE	0.116098395	1	1898	0.003004273	1	1516	0.99125297	1	2325	0.880901309	1	2287	0.976677441	1	2320	0.94173751	1	2308
		Wavelet_Coif3-LHL_glcm_Correlation	FALSE	0.197057862	1	1975	0.024848695	1	1714	0.94173717	1	2308	0.866505949	1	2282	0.962109695	1	2315	0.87801893	1	2286
		Wavelet_Coif3-LHL_glcm_DifferenceAverage	FALSE	2.70332E-06	0.0043118	1044	7.64248E-07	0.001218976	975	0.581082539	1	2177	0.306209971	1	2048	0.606370763	1	2187	0.349528846	1	2072
		Wavelet_Coif3-LHL_glcm_DifferenceEntropy	FALSE	0.000848693	1	1415	9.46602E-06	0.015098299	1116	0.58610055	1	2179	0.347649692	1	2071	0.674304148	1	2213	0.469339873	1	2130
		Wavelet_Coif3-LHL_glcm_DifferenceVariance	FALSE	0.345774158	1	2070	0.144821246	1	1929	0.489773714	1	2139	0.536853203	1	2159	0.906908687	1	2296	0.653085267	1	2205
		Wavelet_Coif3-LHL_glcm_Id	FALSE	1.94215E-10	3.09774E-07	586	3.59749E-09	5.73799E-06	713	0.653083373	1	2205	0.271354974	1	2027	0.753314033	1	2242	0.287598577	1	2037
		Wavelet_Coif3-LHL_glcm_Idm	FALSE	2.34873E-10	3.74622E-07	594	3.51864E-09	5.61223E-06	712	0.663660038	1	2209	0.279396521	1	2032	0.742238913	1	2238	0.287598577	1	2037
		Wavelet_Coif3-LHL_glcm_Idmn	FALSE	2.47144E-16	3.94194E-13	85	1.371E-09	2.18675E-06	670	0.271352173	1	2027	0.255751271	1	2017	0.016031181	1	1669	0.170572507	1	1953
		Wavelet_Coif3-LHL_glcm_Idn	FALSE	2.79056E-16	4.45094E-13	89	2.52948E-12	4.03452E-09	412	0.08389878	1	1855	0.12938312	1	1913	0.016354974	1	1671	0.076338012	1	1843
		Wavelet_Coif3-LHL_glcm_Imc1	FALSE	1.78053E-17	2.83995E-14	0	7.47962E-12	1.193E-08	454	0.017019838	1	1675	0.004895674	1	1558	0.548965864	1	2164	0.010977932	1	1632
		Wavelet_Coif3-LHL_glcm_Imc2	FALSE	1.18735E-16	1.89382E-13	61	1.12995E-06	0.001802267	996	0.058365309	1	1810	0.318460336	1	2055	0.239317282	1	2006	0.349528846	1	2072
		Wavelet_Coif3-LHL_glcm_InverseVariance	FALSE	1.87747E-07	0.000299456	902	4.83229E-05	0.077075069	1216	0.167175196	1	1950	0.655722998	1	2206	0.171713015	1	1954	0.634743919	1	2198
		Wavelet_Coif3-LHL_glcm_JointAverage	FALSE	9.01832E-16	1.43842E-12	128	2.0281E-06	0.003234818	1028	0.04522303	1	1780	0.19079081	1	1970	0.044443606	1	1778	0.223647783	1	1995
		Wavelet_Coif3-LHL_glcm_JointEnergy	FALSE	1.27474E-11	2.03321E-08	475	6.89402E-11	1.0996E-07	543	0.396546544	1	2096	0.10160305	1	1880	0.614052096	1	2190	0.242250782	1	2008
		Wavelet_Coif3-LHL_glcm_JointEntropy	FALSE	6.45105E-07	0.001028943	966	5.74211E-08	9.15866E-05	843	0.553849376	1	2166	0.245206692	1	2010	0.687701293	1	2218	0.349528846	1	2072
		Wavelet_Coif3-LHL_glcm_MCC	FALSE	9.29124E-16	1.48195E-12	129	1.09171E-09	1.74127E-06	660	0.04483189	1	1779	0.284298465	1	2035	0.031945414	1	1741	0.289528288	1	2038
		Wavelet_Coif3-LHL_glcm_MaximumProbability	FALSE	4.47501E-12	7.13765E-09	434	4.71321E-12	7.51757E-09	436	0.539263037	1	2160	0.155163637	1	1939	0.629541818	1	2196	0.163833866	1	1947
		Wavelet_Coif3-LHL_glcm_SumAverage	FALSE	9.01832E-16	1.43842E-12	128	2.0281E-06	0.003234818	1028	0.04522303	1	1780	0.19079081	1	1970	0.044443606	1	1778	0.223647783	1	1995
		Wavelet_Coif3-LHL_glcm_SumEntropy	FALSE	1.31419E-07	0.000209614	884	1.35644E-08	2.16352E-05	774	0.453773977	1	2123	0.240782264	1	2007	0.578581096	1	2176	0.349528846	1	2072
		Wavelet_Coif3-LHL_glcm_SumSquares	FALSE	0.071610604	1	1835	0.00159224	1	1464	0.965022388	1	2316	0.829296174	1	2269	0.979592079	1	2321	0.965022592	1	2316
	GLDM	Wavelet_Coif3-LHL_gldm_DependenceEntropy	FALSE	0.079416897	1	1848	0.001236125	1	1444	0.544103696	1	2162	0.374542082	1	2085	0.650449959	1	2204	0.455981957	1	2124
		Wavelet_Coif3-LHL_gldm_DependenceNonUniformity	FALSE	3.20162E-05	0.05106584	1190	0.06712895	1	1827	0.394514211	1	2095	0.009674479	1	1620	0.35710697	1	2076	0.004571891	1	1552
		Wavelet_Coif3-LHL_gldm_DependenceNonUniformityNormalized	FALSE	3.40643E-06	0.00543326	1057	0.015246831	1	1664	0.98250699	1	2322	0.714789644	1	2228	0.959197507	1	2314	0.469339873	1	2130
		Wavelet_Coif3-LHL_gldm_DependenceVariance	FALSE	9.25067E-14	1.47548E-10	289	2.11289E-07	0.000337005	908	0.539263037	1	2160	0.725728015	1	2232	0.258818266	1	2019	0.362862913	1	2079
		Wavelet_Coif3-LHL_gldm_GrayLevelNonUniformity	FALSE	1.33991E-09	2.13716E-06	669	2.33032E-07	0.000371686	913	0.239317282	1	2006	0.157297821	1	1941	0.271352173	1	2027	0.331027329	1	2062
		Wavelet_Coif3-LHL_gldm_GrayLevelVariance	FALSE	0.12304245	1	1906	0.003543914	1	1530	0.985422135	1	2323	0.880901309	1	2287	0.970849136	1	2318	0.959197745	1	2314
		Wavelet_Coif3-LHL_gldm_HighGrayLevelEmphasis	TRUE	2.94163E-15	4.69191E-12	168	0.000207976	0.331722279	1313	4.32868E-05	0.069042418	1209	2.64033E-05	0.042113249	1178	0.480632205	1	2135	0.009674479	1	1620
		Wavelet_Coif3-LHL_gldm_LargeDependenceEmphasis	FALSE	1.85934E-11	2.96565E-08	490	3.59204E-10	5.7293E-07	612	0.551404963	1	2165	0.18832449	1	1968	0.720250106	1	2230	0.266606881	1	2024
		Wavelet_Coif3-LHL_gldm_LargeDependenceHighGrayLevelEmphasis	TRUE	2.79056E-16	4.45094E-13	89	6.06592E-13	9.67514E-10	358	1.01625E-10	1.62092E-07	559	3.10999E-14	4.96043E-11	250	0.025084067	1	1715	5.34335E-10	8.52265E-07	629
		Wavelet_Coif3-LHL_gldm_LargeDependenceLowGrayLevelEmphasis	FALSE	1.90557E-08	3.03938E-05	790	0.529649296	1	2156	0.775615099	1	2250	0.666316646	1	2210	0.632139605	1	2197	0.739479817	1	2237
		Wavelet_Coif3-LHL_gldm_LowGrayLevelEmphasis	FALSE	3.55549E-16	5.67101E-13	97	5.65178E-07	0.000901459	959	0.329209838	1	2061	0.485193851	1	2137	0.04291873	1	1774	0.553851692	1	2166
		Wavelet_Coif3-LHL_gldm_SmallDependenceEmphasis	FALSE	0.024150725	1	1711	0.000457493	0.729701243	1369	0.58610055	1	2179	0.404741605	1	2100	0.65836316	1	2207	0.492076154	1	2140
		Wavelet_Coif3-LHL_gldm_SmallDependenceHighGrayLevelEmphasis	FALSE	3.03443E-12	4.83992E-09	419	0.024151552	1	1711	0.026542588	1	1721	0.154104825	1	1938	0.832145055	1	2270	0.382455834	1	2089
		Wavelet_Coif3-LHL_gldm_SmallDependenceLowGrayLevelEmphasis	FALSE	5.10536E-16	8.14304E-13	109	0.000398573	0.635723176	1359	0.548965864	1	2164	0.924305231	1	2302	0.019537244	1	1689	0.857889624	1	2279
	GLRLM	Wavelet_Coif3-LHL_glrlm_GrayLevelNonUniformity	FALSE	3.65597E-06	0.005831274	1061	3.57408E-08	5.70066E-05	820	0.351411621	1	2073	0.004122485	1	1543	0.28925547	1	2038	0.051009247	1	1794
		Wavelet_Coif3-LHL_glrlm_GrayLevelNonUniformityNormalized	FALSE	3.92962E-09	6.26774E-06	717	2.57655E-09	4.1096E-06	698	0.480632205	1	2135	0.150961328	1	1935	0.676975497	1	2214	0.32560271	1	2059
		Wavelet_Coif3-LHL_glrlm_GrayLevelVariance	FALSE	0.817918548	1	2265	0.022160909	1	1702	0.709340563	1	2226	0.863632025	1	2281	0.901119478	1	2294	0.800927098	1	2259
		Wavelet_Coif3-LHL_glrlm_HighGrayLevelRunEmphasis	TRUE	2.94163E-15	4.69191E-12	168	0.000174747	0.27872107	1301	4.39738E-05	0.070138192	1210	2.72692E-05	0.043494453	1180	0.478361439	1	2134	0.010092764	1	1624
		Wavelet_Coif3-LHL_glrlm_LongRunEmphasis	FALSE	1.72454E-11	2.75064E-08	487	7.22103E-10	1.15175E-06	642	0.338347727	1	2066	0.08790328	1	1861	0.581082539	1	2177	0.237864064	1	2005
		Wavelet_Coif3-LHL_glrlm_LongRunHighGrayLevelEmphasis	TRUE	2.70716E-16	4.31792E-13	88	2.05214E-12	3.27317E-09	404	1.89644E-10	3.02483E-07	585	3.58061E-14	5.71107E-11	255	0.036266009	1	1755	1.92476E-09	3.06999E-06	685
		Wavelet_Coif3-LHL_glrlm_LongRunLowGrayLevelEmphasis	FALSE	4.29241E-09	6.8464E-06	721	0.994168616	1	2326	0.800925959	1	2259	0.775616375	1	2250	0.593665023	1	2182	0.81508133	1	2264
		Wavelet_Coif3-LHL_glrlm_LowGrayLevelRunEmphasis	FALSE	3.77698E-16	6.02428E-13	99	1.43532E-06	0.002289341	1009	0.360935981	1	2078	0.534446436	1	2158	0.043295747	1	1775	0.611488968	1	2189
		Wavelet_Coif3-LHL_glrlm_RunEntropy	FALSE	2.69811E-13	4.30348E-10	328	0.005928752	1	1575	0.064444519	1	1822	0.944645833	1	2309	0.138872039	1	1923	0.895336131	1	2292
		Wavelet_Coif3-LHL_glrlm_RunLengthNonUniformity	TRUE	1.50092E-09	2.39397E-06	674	5.76036E-06	0.009187768	1087	0.650449959	1	2204	0.208723612	1	1984	0.527256548	1	2155	6.28024E-06	0.01001699	1092
		Wavelet_Coif3-LHL_glrlm_RunLengthNonUniformityNormalized	FALSE	7.59728E-11	1.21177E-07	547	1.50125E-09	2.3945E-06	674	0.603819901	1	2186	0.220879006	1	1993	0.798102763	1	2258	0.255751271	1	2017
		Wavelet_Coif3-LHL_glrlm_RunPercentage	FALSE	4.11924E-11	6.57019E-08	522	6.8951E-10	1.09977E-06	640	0.583589032	1	2178	0.199610135	1	1977	0.786836709	1	2254	0.268183186	1	2025
		Wavelet_Coif3-LHL_glrlm_RunVariance	FALSE	1.15209E-11	1.83759E-08	471	5.10044E-10	8.13521E-07	627	0.322015772	1	2057	0.078174478	1	1846	0.553849376	1	2166	0.220879006	1	1993
		Wavelet_Coif3-LHL_glrlm_ShortRunEmphasis	FALSE	7.59728E-11	1.21177E-07	547	1.3402E-09	2.13763E-06	669	0.480632205	1	2135	0.177516684	1	1959	0.745002796	1	2239	0.295961554	1	2042
		Wavelet_Coif3-LHL_glrlm_ShortRunHighGrayLevelEmphasis	FALSE	2.94163E-15	4.69191E-12	168	0.000584501	0.932278487	1387	0.004677356	1	1554	0.034984537	1	1751	0.906908687	1	2296	0.164942823	1	1948
		Wavelet_Coif3-LHL_glrlm_ShortRunLowGrayLevelEmphasis	FALSE	3.55549E-16	5.67101E-13	97	2.72386E-07	0.000434456	921	0.394514211	1	2095	0.423540439	1	2109	0.034047166	1	1748	0.492076154	1	2140
	GLSZM	Wavelet_Coif3-LHL_glszm_GrayLevelNonUniformity	FALSE	0.563679755	1	2170	0.997084288	1	2327	0.075732022	1	1842	0.034357911	1	1749	0.059838587	1	1813	0.004366944	1	1548
		Wavelet_Coif3-LHL_glszm_GrayLevelNonUniformityNormalized	FALSE	0.134061509	1	1918	0.81508133	1	2264	0.817918548	1	2265	0.772818192	1	2249	0.860759099	1	2280	0.795283375	1	2257
		Wavelet_Coif3-LHL_glszm_GrayLevelVariance	FALSE	8.55104E-05	0.136389057	1253	0.282658057	1	2034	0.069901448	1	1832	0.005862932	1	1574	0.603819901	1	2186	0.054126629	1	1801
		Wavelet_Coif3-LHL_glszm_HighGrayLevelZoneEmphasis	FALSE	2.39548E-15	3.82079E-12	161	4.12906E-05	0.065858525	1206	5.64753E-05	0.090078028	1226	5.47515E-05	0.08732868	1224	0.501330917	1	2144	0.00937096	1	1617
		Wavelet_Coif3-LHL_glszm_LargeAreaEmphasis	FALSE	4.39738E-05	0.070138192	1210	0.000124533	0.198630316	1278	0.285942489	1	2036	0.568628535	1	2172	0.429915799	1	2112	0.513033444	1	2149
		Wavelet_Coif3-LHL_glszm_LargeAreaHighGrayLevelEmphasis	TRUE	2.69399E-15	4.29692E-12	165	4.53935E-10	7.24027E-07	622	9.41436E-09	1.50159E-05	757	1.20346E-10	1.91952E-07	566	0.002169544	1	1489	9.34874E-08	0.000149112	867
		Wavelet_Coif3-LHL_glszm_LargeAreaLowGrayLevelEmphasis	FALSE	0.00014872	0.237208728	1290	0.706623996	1	2225	0.933016965	1	2305	0.005928752	1	1575	0.99125297	1	2325	0.001942697	1	1480
		Wavelet_Coif3-LHL_glszm_LowGrayLevelZoneEmphasis	FALSE	9.57229E-16	1.52678E-12	130	0.023027305	1	1706	0.603819901	1	2186	0.800927098	1	2259	0.060336482	1	1814	0.772818192	1	2249
		Wavelet_Coif3-LHL_glszm_SizeZoneNonUniformity	FALSE	0.145828735	1	1930	0.277775386	1	2031	0.970849136	1	2318	0.005928752	1	1575	0.94173717	1	2308	0.005606187	1	1570
		Wavelet_Coif3-LHL_glszm_SizeZoneNonUniformityNormalized	FALSE	7.78597E-07	0.001241863	976	3.41317E-05	0.05444005	1194	0.598732698	1	2184	0.445015666	1	2119	0.467096035	1	2129	0.492076154	1	2140
		Wavelet_Coif3-LHL_glszm_SmallAreaEmphasis	FALSE	8.39129E-07	0.001338411	980	0.000280814	0.447898	1334	0.603819901	1	2186	0.761655305	1	2245	0.532042731	1	2157	0.655722998	1	2206
		Wavelet_Coif3-LHL_glszm_SmallAreaHighGrayLevelEmphasis	TRUE	1.68203E-15	2.68284E-12	149	2.06507E-06	0.00329379	1029	1.19834E-05	0.019113498	1130	1.47728E-08	2.35626E-05	778	0.626948606	1	2195	0.000223502	0.356486446	1318
		Wavelet_Coif3-LHL_glszm_SmallAreaLowGrayLevelEmphasis	FALSE	1.57352E-14	2.50976E-11	226	0.380467848	1	2088	0.558754064	1	2168	0.304485762	1	2047	0.060336482	1	1814	0.449384032	1	2121
		Wavelet_Coif3-LHL_glszm_ZoneEntropy	FALSE	0.174016209	1	1956	0.006337849	1	1581	0.809411021	1	2262	0.817919593	1	2265	0.693088015	1	2220	0.904014002	1	2295
		Wavelet_Coif3-LHL_glszm_ZonePercentage	FALSE	0.453773977	1	2123	0.033121508	1	1706	0.883784593	1	2288	0.64519816	1	2202	0.898226825	1	2293	0.728471475	1	2233
		Wavelet_Coif3-LHL_glszm_ZoneVariance	FALSE	4.32868E-05	0.069042418	1209	0.000134127	0.213932566	1283	0.282655245	1	2034	0.517752969	1	2151	0.429915799	1	2112	0.515390402	1	2150
	NGTDM	Wavelet_Coif3-LHL_ngtdm_Busyness	FALSE	1.18638E-13	1.89228E-10	298	3.81498E-05	0.060848986	1201	0.189552107	1	1969	0.016852077	1	1674	0.009571932	1	1619	0.000126399	0.20160571	1279
		Wavelet_Coif3-LHL_ngtdm_Coarseness	FALSE	7.47753E-12	1.19267E-08	454	8.1161E-08	0.000129452	860	0.99125297	1	2325	0.013777781	1	1654	0.772816901	1	2249	0.029956836	1	1734
		Wavelet_Coif3-LHL_ngtdm_Complexity	TRUE	1.66587E-14	2.65707E-11	228	4.75772E-06	0.00758856	1076	0.002040617	1	1484	1.17855E-05	0.018797864	1129	0.578581096	1	2176	0.003341808	1	1525
		Wavelet_Coif3-LHL_ngtdm_Contrast	FALSE	1.93728E-16	3.08996E-13	77	2.25267E-09	3.59301E-06	692	0.380465065	1	2088	0.292597031	1	2040	0.052324979	1	1797	0.177516684	1	1959
		Wavelet_Coif3-LHL_ngtdm_Strength	TRUE	2.70716E-16	4.31792E-13	88	6.09943E-09	9.72859E-06	737	7.86918E-12	1.25513E-08	456	5.73649E-05	0.091496971	1227	1.01094E-06	0.001612453	990	0.000980061	1	1426
	First Order	Wavelet_Coif3-LHH_firstorder_10Percentile	FALSE	3.11433E-12	4.96736E-09	420	4.51373E-13	7.1994E-10	347	0.571107293	1	2173	0.245206692	1	2010	0.487479601	1	2138	0.084557266	1	1856
		Wavelet_Coif3-LHH_firstorder_90Percentile	FALSE	3.11433E-12	4.96736E-09	420	8.13894E-13	1.29816E-09	369	0.546532102	1	2163	0.174018724	1	1956	0.496690774	1	2142	0.068782341	1	1830
		Wavelet_Coif3-LHH_firstorder_Energy	TRUE	2.15334E-09	3.43458E-06	690	3.93277E-07	0.000627277	940	0.417215377	1	2106	0.102368728	1	1881	0.179875034	1	1961	3.06039E-07	0.000488133	927
		Wavelet_Coif3-LHH_firstorder_Entropy	FALSE	1.49344E-10	2.38203E-07	575	1.01651E-10	1.62133E-07	559	0.65836316	1	2207	0.240782264	1	2007	0.553849376	1	2166	0.069903083	1	1832
		Wavelet_Coif3-LHH_firstorder_InterquartileRange	FALSE	1.45785E-12	2.32527E-09	391	4.16302E-13	6.64002E-10	344	0.57359345	1	2174	0.17869423	1	1960	0.534444049	1	2158	0.053671783	1	1800
		Wavelet_Coif3-LHH_firstorder_Kurtosis	TRUE	1.78053E-17	2.83995E-14	0	1.38469E-16	2.20858E-13	66	3.82699E-15	6.10406E-12	177	4.48759E-08	7.15771E-05	831	6.84401E-06	0.010916197	1097	8.27062E-09	1.31916E-05	751
		Wavelet_Coif3-LHH_firstorder_Maximum	FALSE	5.09926E-10	8.13332E-07	627	0.64519816	1	2202	0.338347727	1	2066	0.927208316	1	2303	0.58861707	1	2180	0.935923232	1	2306
		Wavelet_Coif3-LHH_firstorder_MeanAbsoluteDeviation	FALSE	4.59195E-12	7.32415E-09	435	1.6203E-12	2.58438E-09	395	0.581082539	1	2177	0.189554694	1	1969	0.539263037	1	2160	0.084557266	1	1856
		Wavelet_Coif3-LHH_firstorder_Mean	FALSE	0.73947835	1	2237	0.513033444	1	2149	0.637348798	1	2199	0.063398004	1	1820	0.527256548	1	2155	0.212720348	1	1987
		Wavelet_Coif3-LHH_firstorder_Median	FALSE	0.033132364	1	1745	0.671638705	1	2212	0.778416214	1	2251	0.062364054	1	1818	0.78122021	1	2252	0.086552279	1	1859
		Wavelet_Coif3-LHH_firstorder_Minimum	FALSE	1.43437E-09	2.28783E-06	672	0.478364009	1	2134	0.329209838	1	2061	0.739479817	1	2237	0.695787227	1	2221	0.892446236	1	2291
		Wavelet_Coif3-LHH_firstorder_Range	FALSE	4.77467E-11	7.61559E-08	528	0.598734834	1	2184	0.364790645	1	2080	0.703909544	1	2224	0.608926434	1	2188	0.714789644	1	2228
		Wavelet_Coif3-LHH_firstorder_RobustMeanAbsoluteDeviation	FALSE	1.53674E-12	2.4511E-09	393	4.51373E-13	7.1994E-10	347	0.541680668	1	2161	0.204781577	1	1981	0.494379317	1	2141	0.080678879	1	1850
		Wavelet_Coif3-LHH_firstorder_RootMeanSquared	FALSE	1.20316E-10	1.91904E-07	566	2.83871E-10	4.52774E-07	602	0.645196229	1	2202	0.142817643	1	1927	0.626948606	1	2195	0.136934407	1	1921
		Wavelet_Coif3-LHH_firstorder_Skewness	FALSE	1.36975E-05	0.021847484	1138	0.973763266	1	2319	0.269763084	1	2026	0.074536883	1	1840	0.255748494	1	2017	0.573595689	1	2174
		Wavelet_Coif3-LHH_firstorder_TotalEnergy	TRUE	2.15334E-09	3.43458E-06	690	4.00902E-07	0.000639438	941	0.417215377	1	2106	0.098585834	1	1876	0.181064189	1	1962	3.06039E-07	0.000488133	927
		Wavelet_Coif3-LHH_firstorder_Uniformity	FALSE	1.42353E-10	2.27054E-07	573	5.01631E-11	8.00102E-08	530	0.679650915	1	2215	0.322018599	1	2057	0.458190688	1	2125	0.053671783	1	1800
		Wavelet_Coif3-LHH_firstorder_Variance	FALSE	3.85296E-10	6.14547E-07	615	5.10044E-10	8.13521E-07	627	0.64257597	1	2201	0.232102801	1	2001	0.522492428	1	2153	0.236414368	1	2004
		Wavelet_Coif3-LHH_glcm_Autocorrelation	FALSE	2.66863E-08	4.25647E-05	806	0.12938312	1	1913	0.294273248	1	2041	0.320236236	1	2056	0.706622361	1	2225	0.318460336	1	2055
		Wavelet_Coif3-LHH_glcm_ClusterProminence	TRUE	0.000132142	0.21076685	1282	5.28181E-06	0.008424486	1082	0.003150075	1	1520	8.05544E-14	1.28484E-10	284	0.146843953	1	1931	6.5749E-07	0.001048697	967
		Wavelet_Coif3-LHH_glcm_ClusterShade	FALSE	0.003585451	1	1531	0.195794628	1	1974	0.709340563	1	2226	0.117806995	1	1900	0.886670109	1	2289	0.611488968	1	2189
		Wavelet_Coif3-LHH_glcm_ClusterTendency	FALSE	1.44584E-08	2.30612E-05	777	1.94666E-08	3.10493E-05	791	0.49900798	1	2143	0.370623454	1	2083	0.340194664	1	2067	0.299351875	1	2044
		Wavelet_Coif3-LHH_glcm_Contrast	FALSE	6.51079E-09	1.03847E-05	740	8.09335E-09	1.29089E-05	750	0.494379317	1	2141	0.349528846	1	2072	0.318457508	1	2055	0.255751271	1	2017
		Wavelet_Coif3-LHH_glcm_Correlation	FALSE	4.84711E-17	7.73114E-14	32	4.54677E-11	7.2521E-08	526	0.313168582	1	2052	0.206089528	1	1982	0.219501177	1	1992	0.123934996	1	1907
		Wavelet_Coif3-LHH_glcm_DifferenceAverage	FALSE	1.32464E-10	2.1128E-07	570	5.81081E-11	9.26825E-08	536	0.556299084	1	2167	0.299351875	1	2044	0.362860109	1	2079	0.046817764	1	1784
		Wavelet_Coif3-LHH_glcm_DifferenceEntropy	FALSE	6.56418E-11	1.04699E-07	541	5.81081E-11	9.26825E-08	536	0.653083373	1	2205	0.210049766	1	1985	0.566150423	1	2171	0.063920267	1	1821
		Wavelet_Coif3-LHH_glcm_DifferenceVariance	FALSE	1.66777E-07	0.00026601	896	1.54102E-07	0.000245792	892	0.432054154	1	2113	0.534446436	1	2158	0.402681349	1	2099	0.753315427	1	2242
		Wavelet_Coif3-LHH_glcm_Id	FALSE	5.66889E-11	9.04187E-08	535	2.84265E-11	4.53402E-08	507	0.687701293	1	2218	0.332848451	1	2063	0.410949003	1	2103	0.046015217	1	1782
		Wavelet_Coif3-LHH_glcm_Idm	FALSE	5.80933E-11	9.26587E-08	536	2.51017E-11	4.00372E-08	502	0.682330374	1	2216	0.331027329	1	2062	0.400630121	1	2098	0.04444485	1	1778

Wavelet Coiflet 3 - LHH	GLCM	Wavelet_Coif3-LHH_glcm_Idmn	FALSE	4.60899E-14	7.35134E-11	264	6.90474E-05	0.110130672	1239	0.619196686	1	2192	0.353306456	1	2074	0.501330917	1	2144	0.349528846	1	2072
		Wavelet_Coif3-LHH_glcm_Idn	FALSE	5.12013E-15	8.1666E-12	187	2.25986E-06	0.003604481	1034	0.34204805	1	2068	0.351414435	1	2073	0.309674942	1	2050	0.338350549	1	2066
		Wavelet_Coif3-LHH_glcm_Imc1	FALSE	0.009881219	1	1622	0.508336427	1	2147	0.901119478	1	2294	0.855021221	1	2278	0.79246432	1	2256	0.927208316	1	2303
		Wavelet_Coif3-LHH_glcm_Imc2	FALSE	0.274549558	1	2029	0.451577303	1	2122	0.92430479	1	2302	0.826448572	1	2268	0.912702845	1	2298	0.860759904	1	2280
		Wavelet_Coif3-LHH_glcm_InverseVariance	FALSE	0.032832142	1	1744	0.918502123	1	2300	0.254223133	1	2016	0.886670766	1	2289	0.294273248	1	2041	0.81508133	1	2264
		Wavelet_Coif3-LHH_glcm_JointAverage	FALSE	4.20863E-06	0.006712764	1069	0.30794064	1	2049	0.329209838	1	2061	0.306209971	1	2048	0.329209838	1	2061	0.299351875	1	2044
		Wavelet_Coif3-LHH_glcm_JointEnergy	FALSE	3.55212E-11	5.66563E-08	516	4.12031E-11	6.5719E-08	522	0.722986518	1	2231	0.309677768	1	2050	0.529646891	1	2156	0.055045973	1	1803
		Wavelet_Coif3-LHH_glcm_JointEntropy	FALSE	7.59728E-11	1.21177E-07	547	5.53318E-11	8.82544E-08	534	0.674304148	1	2213	0.230678093	1	2000	0.546532102	1	2163	0.058854496	1	1811
		Wavelet_Coif3-LHH_glcm_MCC	FALSE	0.006697662	1	1586	0.368673722	1	2082	0.294273248	1	2041	0.434201343	1	2114	0.261913477	1	2021	0.478364009	1	2134
		Wavelet_Coif3-LHH_glcm_MaximumProbability	FALSE	4.03579E-12	6.43708E-09	430	6.58192E-12	1.04982E-08	449	0.63996015	1	2200	0.284298465	1	2035	0.33650724	1	2065	0.035942758	1	1754
		Wavelet_Coif3-LHH_glcm_SumAverage	FALSE	4.20863E-06	0.006712764	1069	0.30794064	1	2049	0.329209838	1	2061	0.306209971	1	2048	0.329209838	1	2061	0.299351875	1	2044
		Wavelet_Coif3-LHH_glcm_SumEntropy	FALSE	1.17454E-10	1.87338E-07	565	1.06681E-10	1.70156E-07	561	0.679650915	1	2215	0.230678093	1	2000	0.548965864	1	2164	0.05934559	1	1812
		Wavelet_Coif3-LHH_glcm_SumSquares	FALSE	9.01641E-09	1.43812E-05	755	1.27217E-08	2.0291E-05	771	0.487479601	1	2138	0.362862913	1	2079	0.332845627	1	2063	0.276160667	1	2030
	GLDM	Wavelet_Coif3-LHH_gldm_DependenceEntropy	FALSE	2.9058E-10	4.63475E-07	603	1.11955E-10	1.78568E-07	563	0.603819901	1	2186	0.246694098	1	2011	0.546532102	1	2163	0.168303599	1	1951
		Wavelet_Coif3-LHH_gldm_DependenceNonUniformity	FALSE	1.0411E-10	1.66056E-07	560	4.79294E-09	7.64475E-06	726	0.311418531	1	2051	0.000724232	1	1403	0.440668947	1	2117	0.00279685	1	1510
		Wavelet_Coif3-LHH_gldm_DependenceNonUniformityNormalized	FALSE	4.8934E-11	7.80497E-08	529	4.03867E-10	6.44168E-07	617	0.614052096	1	2190	0.313171409	1	2052	0.616622038	1	2191	0.392490957	1	2094
		Wavelet_Coif3-LHH_gldm_DependenceVariance	FALSE	6.14039E-10	9.79392E-07	635	1.71951E-09	2.74262E-06	680	0.544103696	1	2162	0.284298465	1	2035	0.655721116	1	2206	0.370623454	1	2083
		Wavelet_Coif3-LHH_gldm_GrayLevelNonUniformity	FALSE	4.43356E-10	7.07154E-07	621	9.0182E-09	1.4384E-05	755	0.440668947	1	2117	0.008419727	1	1607	0.421424076	1	2108	0.003040177	1	1517
		Wavelet_Coif3-LHH_gldm_GrayLevelVariance	FALSE	2.25599E-08	3.5983E-05	798	1.78864E-08	2.85287E-05	787	0.460408054	1	2126	0.402684098	1	2099	0.334673206	1	2064	0.392490957	1	2094
		Wavelet_Coif3-LHH_gldm_HighGrayLevelEmphasis	FALSE	0.021529089	1	1699	0.197060479	1	1975	0.294273248	1	2041	0.320236236	1	2056	0.76722939	1	2247	0.318460336	1	2055
		Wavelet_Coif3-LHH_gldm_LargeDependenceEmphasis	FALSE	4.11924E-11	6.57019E-08	522	1.14686E-10	1.82924E-07	564	0.65836316	1	2207	0.410951736	1	2103	0.419316621	1	2107	0.189554694	1	1969
		Wavelet_Coif3-LHH_gldm_LargeDependenceHighGrayLevelEmphasis	FALSE	2.48661E-13	3.96614E-10	325	1.23931E-05	0.01976695	1132	0.436349298	1	2115	0.251194202	1	2014	0.815080269	1	2264	0.616624097	1	2191
		Wavelet_Coif3-LHH_gldm_LargeDependenceLowGrayLevelEmphasis	FALSE	0.198329689	1	1976	0.029408327	1	1732	0.442837931	1	2118	0.536853203	1	2159	0.451574659	1	2122	0.445015666	1	2119
		Wavelet_Coif3-LHH_gldm_LowGrayLevelEmphasis	FALSE	5.35909E-12	8.54774E-09	441	0.00095487	1	1424	0.522492428	1	2153	0.125730786	1	1909	0.695787227	1	2221	0.123934996	1	1907
		Wavelet_Coif3-LHH_gldm_SmallDependenceEmphasis	FALSE	5.09036E-12	8.11913E-09	439	2.84265E-11	4.53402E-08	507	0.720250106	1	2230	0.142817643	1	1927	0.695787227	1	2221	0.058854496	1	1811
		Wavelet_Coif3-LHH_gldm_SmallDependenceHighGrayLevelEmphasis	FALSE	0.58861707	1	2180	0.009173395	1	1615	0.94173717	1	2308	0.520121127	1	2152	1	1	2328	0.690394409	1	2219
		Wavelet_Coif3-LHH_gldm_SmallDependenceLowGrayLevelEmphasis	FALSE	1.0066E-12	1.60552E-09	377	6.50052E-06	0.010368336	1094	0.534444049	1	2158	0.364793447	1	2080	0.225038734	1	1996	0.13501544	1	1919
	GLRLM	Wavelet_Coif3-LHH_glrlm_GrayLevelNonUniformity	FALSE	4.75506E-10	7.58432E-07	624	1.35644E-08	2.16352E-05	774	0.279393712	1	2032	0.991253021	1	2325	0.263470636	1	2022	0.017020483	1	1675
		Wavelet_Coif3-LHH_glrlm_GrayLevelNonUniformityNormalized	FALSE	5.53177E-11	8.82317E-08	534	5.67034E-11	9.04419E-08	535	0.73672114	1	2236	0.285945304	1	2036	0.596196407	1	2183	0.082599622	1	1853
		Wavelet_Coif3-LHH_glrlm_GrayLevelVariance	FALSE	3.08905E-08	4.92703E-05	813	3.15471E-08	5.03176E-05	814	0.451574659	1	2122	0.394516973	1	2095	0.364790645	1	2080	0.544106049	1	2162
		Wavelet_Coif3-LHH_glrlm_HighGrayLevelRunEmphasis	FALSE	0.021117138	1	1697	0.290924438	1	2039	0.294273248	1	2041	0.320236236	1	2056	0.843566224	1	2274	0.316690898	1	2054
		Wavelet_Coif3-LHH_glrlm_LongRunEmphasis	FALSE	3.29796E-11	5.26025E-08	513	3.76458E-10	6.00451E-07	614	0.65836316	1	2207	0.294276069	1	2041	0.576084726	1	2175	0.242250782	1	2008
		Wavelet_Coif3-LHH_glrlm_LongRunHighGrayLevelEmphasis	FALSE	3.44436E-13	5.49376E-10	337	9.30694E-06	0.014844565	1115	0.24968508	1	2013	0.111919313	1	1893	0.693088015	1	2220	0.685015585	1	2217
		Wavelet_Coif3-LHH_glrlm_LongRunLowGrayLevelEmphasis	FALSE	0.23641163	1	2004	0.028337186	1	1728	0.413031558	1	2104	0.423540439	1	2109	0.421424076	1	2108	0.336510063	1	2065
		Wavelet_Coif3-LHH_glrlm_LowGrayLevelRunEmphasis	FALSE	6.92562E-12	1.10464E-08	451	0.00100587	1	1428	0.492073625	1	2140	0.159454178	1	1943	0.650449959	1	2204	0.158373221	1	1942
		Wavelet_Coif3-LHH_glrlm_RunEntropy	FALSE	7.35982E-07	0.001173891	973	4.87292E-08	7.77231E-05	835	0.451574659	1	2122	0.292597031	1	2040	0.485191301	1	2137	0.257282983	1	2018
		Wavelet_Coif3-LHH_glrlm_RunLengthNonUniformity	TRUE	2.64387E-10	4.21698E-07	599	7.91978E-09	1.26321E-05	749	0.455979325	1	2124	0.024848695	1	1714	0.304482936	1	2047	2.77123E-05	0.044201104	1181
		Wavelet_Coif3-LHH_glrlm_RunLengthNonUniformityNormalized	FALSE	7.06228E-11	1.12643E-07	544	2.38812E-11	3.80904E-08	500	0.603819901	1	2186	0.287598577	1	2037	0.396546544	1	2096	0.116100526	1	1898
		Wavelet_Coif3-LHH_glrlm_RunPercentage	FALSE	4.65875E-11	7.43071E-08	527	4.12031E-11	6.5719E-08	522	0.619196686	1	2192	0.282658057	1	2034	0.434198658	1	2114	0.137901747	1	1922
		Wavelet_Coif3-LHH_glrlm_RunVariance	FALSE	2.16061E-11	3.44617E-08	496	1.42388E-10	2.2711E-07	573	0.598732698	1	2184	0.343910701	1	2069	0.534444049	1	2158	0.311421358	1	2051
		Wavelet_Coif3-LHH_glrlm_ShortRunEmphasis	FALSE	1.29319E-10	2.06264E-07	569	1.06681E-10	1.70156E-07	561	0.671636899	1	2212	0.266606881	1	2024	0.544103696	1	2162	0.12938312	1	1913
		Wavelet_Coif3-LHH_glrlm_ShortRunHighGrayLevelEmphasis	FALSE	0.024613563	1	1713	0.160540707	1	1944	0.661009477	1	2208	0.46486335	1	2128	0.843566224	1	2274	0.359021075	1	2077
		Wavelet_Coif3-LHH_glrlm_ShortRunLowGrayLevelEmphasis	FALSE	4.14146E-12	6.60563E-09	431	0.000794634	1	1410	0.541680668	1	2161	0.255751271	1	2017	0.634741941	1	2198	0.265036963	1	2023
	GLSZM	Wavelet_Coif3-LHH_glszm_GrayLevelNonUniformity	TRUE	2.5217E-10	4.02212E-07	597	7.53508E-09	1.20184E-05	655	0.676975497	1	2214	0.015556577	1	1666	0.676975497	1	2214	2.90824E-05	0.046386423	1184
		Wavelet_Coif3-LHH_glszm_GrayLevelNonUniformityNormalized	FALSE	0.047630759	1	1786	0.09218451	1	1746	0.895335523	1	2292	0.374542082	1	2085	0.857888803	1	2279	0.417218098	1	2106
		Wavelet_Coif3-LHH_glszm_GrayLevelVariance	FALSE	0.374539291	1	2085	0.044873413	1	1662	0.00161221	1	1465	0.001144604	1	1438	0.05277028	1	1798	0.020117902	1	1692
		Wavelet_Coif3-LHH_glszm_HighGrayLevelZoneEmphasis	FALSE	5.91664E-05	0.094370385	1229	0.140684268	1	1801	0.360935981	1	2078	0.857889624	1	2279	0.979592079	1	2321	0.510682113	1	2148
		Wavelet_Coif3-LHH_glszm_LargeAreaEmphasis	TRUE	9.6828E-11	1.54441E-07	557	4.10797E-09	6.55221E-06	719	0.153049112	1	1937	4.89945E-09	7.81463E-06	727	0.225038734	1	1996	0.139852221	1	1924
		Wavelet_Coif3-LHH_glszm_LargeAreaHighGrayLevelEmphasis	TRUE	4.30027E-15	6.85893E-12	181	1.371E-09	2.18675E-06	670	3.26903E-10	5.2141E-07	608	1.01651E-10	1.62133E-07	559	0.030233408	1	1735	1.67153E-05	0.02666093	1150
		Wavelet_Coif3-LHH_glszm_LargeAreaLowGrayLevelEmphasis	FALSE	0.000250605	0.399715044	1326	5.64809E-05	0.090087039	1226	0.380465065	1	2088	3.69582E-05	0.058948276	1199	0.837851369	1	2272	0.216772059	1	1990
		Wavelet_Coif3-LHH_glszm_LowGrayLevelZoneEmphasis	FALSE	2.65543E-06	0.004235418	1043	0.004336026	1	1441	0.144818898	1	1929	0.970849306	1	2318	0.179875034	1	1961	0.950464655	1	2311
		Wavelet_Coif3-LHH_glszm_SizeZoneNonUniformity	FALSE	1.68177E-11	2.68243E-08	486	3.64069E-08	5.8069E-05	772	0.703907896	1	2224	0.042919772	1	1774	0.938829607	1	2307	0.000553716	0.883180474	1383
		Wavelet_Coif3-LHH_glszm_SizeZoneNonUniformityNormalized	FALSE	1.81484E-05	0.028946758	1155	0.001257064	1	1411	0.892445612	1	2291	0.524874142	1	2154	0.806580124	1	2261	0.462634024	1	2127
		Wavelet_Coif3-LHH_glszm_SmallAreaEmphasis	FALSE	9.38501E-07	0.001496909	986	0.008510804	1	1608	0.878018223	1	2286	0.314927922	1	2053	0.778416214	1	2251	0.314927922	1	2053
		Wavelet_Coif3-LHH_glszm_SmallAreaHighGrayLevelEmphasis	FALSE	1.01625E-10	1.62092E-07	559	0.001533398	1	1461	0.400630121	1	2098	0.30276801	1	2046	0.583589032	1	2178	0.918502123	1	2300
		Wavelet_Coif3-LHH_glszm_SmallAreaLowGrayLevelEmphasis	FALSE	0.009674055	1	1620	0.33467603	1	2064	0.34952603	1	2072	0.529649296	1	2156	0.390468494	1	2093	0.578583315	1	2176
		Wavelet_Coif3-LHH_glszm_ZoneEntropy	FALSE	0.157295396	1	1941	0.000427409	0.681717404	1331	0.85215388	1	2277	0.487482144	1	2138	0.826447574	1	2268	0.458193314	1	2125
		Wavelet_Coif3-LHH_glszm_ZonePercentage	FALSE	1.85934E-11	2.96565E-08	490	8.97157E-11	1.43097E-07	474	0.485191301	1	2137	0.202183805	1	1979	0.608926434	1	2188	0.139852221	1	1924
		Wavelet_Coif3-LHH_glszm_ZoneVariance	TRUE	9.6828E-11	1.54441E-07	557	1.24522E-08	1.98613E-05	770	0.150958941	1	1935	3.67805E-09	5.8665E-06	714	0.23641163	1	2004	0.362862913	1	2079
	NGTDM	Wavelet_Coif3-LHH_ngtdm_Busyness	FALSE	0.0017161	1	1470	0.400632873	1	2098	0.784027053	1	2253	0.033740901	1	1747	0.950464366	1	2311	0.008064072	1	1603
		Wavelet_Coif3-LHH_ngtdm_Coarseness	FALSE	8.63483E-09	1.37726E-05	753	8.1161E-08	0.000129452	860	0.451574659	1	2122	0.906909228	1	2296	0.566150423	1	2171	0.002898868	1	1513
		Wavelet_Coif3-LHH_ngtdm_Complexity	FALSE	0.000445061	0.709871805	1367	0.994168616	1	2326	0.663660038	1	2209	0.037926469	1	1760	0.626948606	1	2195	0.047223435	1	1785
		Wavelet_Coif3-LHH_ngtdm_Contrast	FALSE	2.62412E-14	4.18547E-11	244	1.61721E-05	0.025794491	1148	0.501330917	1	2144	0.343910701	1	2069	0.366727585	1	2081	0.396549303	1	2096
		Wavelet_Coif3-LHH_ngtdm_Strength	TRUE	4.01892E-11	6.41018E-08	521	0.017534596	1	1678	0.017884186	1	1680	0.060337982	1	1814	1.5744E-08	2.51117E-05	781	0.125730786	1	1909
Wavelet Coiflet 3 - HLL	First Order	Wavelet_Coif3-HLL_firstorder_10Percentile	FALSE	1.64004E-11	2.61587E-08	485	9.30071E-10	1.48346E-06	653	0.578581096	1	2176	0.236414368	1	2004	0.647820901	1	2203	0.322018599	1	2057
		Wavelet_Coif3-HLL_firstorder_90Percentile	FALSE	1.41014E-11	2.24918E-08	479	6.73757E-10	1.07464E-06	639	0.601273871	1	2185	0.195794628	1	1974	0.682330374	1	2216	0.322018599	1	2057
		Wavelet_Coif3-HLL_firstorder_Energy	FALSE	0.003981626	1	1540	0.014942445	1	1662	0.539263037	1	2160	0.000743732	1	1405	0.553849376	1	2166	0.000584501	0.932278487	1387
		Wavelet_Coif3-HLL_firstorder_Entropy	FALSE	4.29241E-09	6.8464E-06	721	4.41215E-07	0.000703738	946	0.632139605	1	2197	0.227847348	1	1998	0.761653954	1	2245	0.376510966	1	2086
		Wavelet_Coif3-HLL_firstorder_InterquartileRange	FALSE	1.99849E-12	3.18759E-09	403	5.94058E-12	9.47522E-09	445	0.661009477	1	2208	0.164942823	1	1948	0.709340563	1	2226	0.19833231	1	1976
		Wavelet_Coif3-HLL_firstorder_Kurtosis	FALSE	3.2322E-17	5.15536E-14	19	1.95776E-17	3.12262E-14	3	0.000432978	0.690599531	1365	0.520121127	1	2152	0.001552687	1	1462	0.027045669	1	1723
		Wavelet_Coif3-HLL_firstorder_Maximum	FALSE	1.4862E-14	2.37049E-11	224	4.39598E-08	7.01158E-05	830	0.026294529	1	1720	0.004571891	1	1552	0.23641163	1	2004	0.048875799	1	1789
		Wavelet_Coif3-HLL_firstorder_MeanAbsoluteDeviation	FALSE	3.26903E-10	5.2141E-07	608	1.14287E-07	0.000182288	877	0.571107293	1	2173	0.254225908	1	2016	0.701197198	1	2223	0.376510966	1	2086
		Wavelet_Coif3-HLL_firstorder_Mean	FALSE	0.272947663	1	2028	0.642577912	1	2201	0.98250699	1	2322	0.997084288	1	2327	0.921402696	1	2301	0.875138167	1	2285
		Wavelet_Coif3-HLL_firstorder_Median	FALSE	8.87477E-07	0.001415526	983	0.603822015	1	2186	0.886670109	1	2289	0.731218417	1	2234	0.820759216	1	2266	0.676977277	1	2214
		Wavelet_Coif3-HLL_firstorder_Minimum	FALSE	6.40843E-17	1.02214E-13	41	6.49076E-08	0.000103528	849	0.001918754	1	1479	8.29571E-05	0.132316501	1251	0.927207892	1	2303	0.092766222	1	1868
		Wavelet_Coif3-HLL_firstorder_Range	FALSE	2.1296E-15	3.39671E-12	157	1.60855E-08	2.56563E-05	782	0.005420062	1	1567	0.003004273	1	1516	0.327401631	1	2060	0.076338012	1	1843
		Wavelet_Coif3-HLL_firstorder_RobustMeanAbsoluteDeviation	FALSE	2.95654E-12	4.71569E-09	418	1.3077E-11	2.08578E-08	476	0.637348798	1	2199	0.164942823	1	1948	0.728469951	1	2233	0.206089528	1	1982
		Wavelet_Coif3-HLL_firstorder_RootMeanSquared	FALSE	0.00071461	1	1402	0.002169674	1	1489	0.698490298	1	2222	0.58359123	1	2178	0.817918548	1	2265	0.817919593	1	2265
		Wavelet_Coif3-HLL_firstorder_Skewness	TRUE	2.47059E-07	0.00039406	916	0.000930278	1	1422	4.12863E-05	0.065851708	1206	0.226441295	1	1997	1.26201E-06	0.002012912	1002	0.829296174	1	2269
		Wavelet_Coif3-HLL_firstorder_TotalEnergy	FALSE	0.004028015	1	1541	0.014942445	1	1662	0.534444049	1	2158	0.000743732	1	1405	0.551404963	1	2165	0.00059244	0.944941451	1388
		Wavelet_Coif3-HLL_firstorder_Uniformity	FALSE	3.1385E-11	5.00591E-08	511	6.14179E-10	9.79616E-07	635	0.637348798	1	2199	0.18832449	1	1968	0.703907896	1	2224	0.251194202	1	2014
		Wavelet_Coif3-HLL_firstorder_Variance	FALSE	0.002730513	1	1508	0.009471189	1	1618	0.85215388	1	2277	0.340197485	1	2067	0.869380882	1	2283	0.447196816	1	2120
	GLCM	Wavelet_Coif3-HLL_glcm_Autocorrelation	TRUE	2.77683E-14	4.42904E-11	246	2.06507E-06	0.00329379	1029	8.82362E-09	1.40737E-05	754	1.74392E-13	2.78155E-10	312	0.001895198	1	1478	1.33378E-06	0.002127378	1005
		Wavelet_Coif3-HLL_glcm_ClusterProminence	TRUE	0.000371817	0.593048021	1354	0.154104825	1	1938	0.000415426	0.662605087	1362	3.45096E-16	5.50428E-13	96	5.75029E-15	9.17171E-12	191	0.240782264	1	2007
		Wavelet_Coif3-HLL_glcm_ClusterShade	TRUE	4.53789E-05	0.07237942	1212	0.025563198	1	1717	1.08998E-12	1.73852E-09	380	6.57331E-13	1.04844E-09	361	1.03367E-12	1.6487E-09	378	3.2883E-06	0.005244846	1055
		Wavelet_Coif3-HLL_glcm_ClusterTendency	FALSE	0.000859919	1	1416	0.006267949	1	1580	0.878018223	1	2286	0.442840596	1	2118	0.901119478	1	2294	0.487482144	1	2138
		Wavelet_Coif3-HLL_glcm_Contrast	FALSE	0.004624231	1	1553	0.017534596	1	1678	0.92430479	1	2302	0.408875433	1	2102	0.935922859	1	2306	0.568628535	1	2172
		Wavelet_Coif3-HLL_glcm_Correlation	FALSE	0.129380881	1	1913	0.02347143	1	1708	0.970849136	1	2318	0.742240365	1	2238	0.997084271	1	2327	0.725728015	1	2232
		Wavelet_Coif3-HLL_glcm_DifferenceAverage	FALSE	1.98786E-08	3.17064E-05	792	4.92662E-06	0.007857958	1078	0.666314815	1	2210	0.343910701	1	2069	0.73672114	1	2236	0.432056844	1	2113
		Wavelet_Coif3-HLL_glcm_DifferenceEntropy	FALSE	1.10909E-06	0.001768992	995	2.17321E-05	0.034662654	1166	0.698490298	1	2222	0.316690898	1	2054	0.812244415	1	2263	0.471587042	1	2131
		Wavelet_Coif3-HLL_glcm_DifferenceVariance	FALSE	0.568626276	1	2172	0.55630139	1	2167	0.581082539	1	2177	0.010092764	1	1624	0.880900619	1	2287	0.284298465	1	2035
		Wavelet_Coif3-HLL_glcm_Id	FALSE	5.3979E-11	8.60965E-08	533	3.59749E-09	5.73799E-06	713	0.733967315	1	2235	0.268183186	1	2025	0.786836709	1	2254	0.316690898	1	2054
		Wavelet_Coif3-HLL_glcm_Idm	FALSE	5.95317E-11	9.4953E-08	537	4.38899E-09	7.00044E-06	722	0.728469951	1	2233	0.261916264	1	2021	0.778416214	1	2251	0.30276801	1	2046
		Wavelet_Coif3-HLL_glcm_Idmn	FALSE	4.52629E-16	7.21943E-13	105	4.39372E-13	7.00799E-10	346	0.21137934	1	1986	0.453776615	1	2123	0.061850811	1	1817	0.104693338	1	1884
		Wavelet_Coif3-HLL_glcm_Idn	FALSE	3.34682E-16	5.33817E-13	95	2.32696E-15	3.71151E-12	160	0.12482817	1	1908	0.179877577	1	1961	0.041438317	1	1770	0.042919946	1	1774
		Wavelet_Coif3-HLL_glcm_Imc1	FALSE	1.78053E-17	2.83995E-14	0	1.06807E-11	1.70357E-08	468	0.016031181	1	1669	0.040002541	1	1766	0.311418531	1	2051	0.069340852	1	1831
		Wavelet_Coif3-HLL_glcm_Imc2	FALSE	9.00201E-17	1.43582E-13	52	4.76254E-07	0.000759625	950	0.076944492	1	1844	0.332848451	1	2063	0.257280203	1	2018	0.355204905	1	2075
		Wavelet_Coif3-HLL_glcm_InverseVariance	FALSE	2.15334E-09	3.43458E-06	690	2.2027E-09	3.5133E-06	691	0.206086878	1	1982	0.410951736	1	2103	0.254223133	1	2016	0.359021075	1	2077
		Wavelet_Coif3-HLL_glcm_JointAverage	FALSE	8.13521E-15	1.29757E-11	203	1.01109E-06	0.001612687	990	0.050575079	1	1793	0.00535963	1	1566	0.410949003	1	2103	0.10160305	1	1880
		Wavelet_Coif3-HLL_glcm_JointEnergy	FALSE	7.47753E-12	1.19267E-08	454	7.23822E-11	1.1545E-07	545	0.536850824	1	2159	0.117806995	1	1900	0.690392694	1	2219	0.242250782	1	2008
		Wavelet_Coif3-HLL_glcm_JointEntropy	FALSE	5.59625E-10	8.92602E-07	631	1.01325E-07	0.000161614	871	0.593665023	1	2182	0.214064797	1	1988	0.73672114	1	2236	0.33467603	1	2064
		Wavelet_Coif3-HLL_glcm_MCC	FALSE	1.36734E-15	2.18091E-12	142	1.50125E-09	2.3945E-06	674	0.485191301	1	2137	0.758872214	1	2244	0.534444049	1	2158	0.739479817	1	2237
		Wavelet_Coif3-HLL_glcm_MaximumProbability	FALSE	2.10597E-12	3.35903E-09	405	2.73525E-12	4.36272E-09	415	0.619196686	1	2192	0.137901747	1	1922	0.637348798	1	2199	0.132176039	1	1916
		Wavelet_Coif3-HLL_glcm_SumAverage	FALSE	8.13521E-15	1.29757E-11	203	1.01109E-06	0.001612687	990	0.050575079	1	1793	0.00535963	1	1566	0.410949003	1	2103	0.10160305	1	1880
		Wavelet_Coif3-HLL_glcm_SumEntropy	FALSE	2.2399E-10	3.57264E-07	592	3.08962E-08	4.92794E-05	813	0.517750523	1	2151	0.189554694	1	1969	0.629541818	1	2196	0.297653489	1	2043
		Wavelet_Coif3-HLL_glcm_SumSquares	FALSE	0.001942578	1	1480	0.010638221	1	1629	0.909805166	1	2297	0.447196816	1	2120	0.904013444	1	2295	0.541683029	1	2161
	GLDM	Wavelet_Coif3-HLL_gldm_DependenceEntropy	FALSE	7.22947E-05	0.115310022	1242	0.000226736	0.361643126	1319	0.417215377	1	2106	0.263473425	1	2022	0.553849376	1	2166	0.360938787	1	2078
		Wavelet_Coif3-HLL_gldm_DependenceNonUniformity	FALSE	0.100839947	1	1879	0.970849306	1	2318	0.359018267	1	2077	0.06604497	1	1825	0.353303643	1	2074	0.236414368	1	2004
		Wavelet_Coif3-HLL_gldm_DependenceNonUniformityNormalized	FALSE	0.160538263	1	1944	0.189554694	1	1969	0.73947835	1	2237	0.938829964	1	2307	0.784027053	1	2253	0.924305231	1	2302
		Wavelet_Coif3-HLL_gldm_DependenceVariance	FALSE	8.97763E-08	0.000143193	865	1.26022E-05	0.020100441	1133	0.927207892	1	2303	0.642577912	1	2201	0.784027053	1	2253	0.505996405	1	2146
		Wavelet_Coif3-HLL_gldm_GrayLevelNonUniformity	FALSE	1.38979E-10	2.21672E-07	572	2.77736E-07	0.000442988	922	0.294273248	1	2041	0.003585646	1	1531	0.314925095	1	2053	0.005660404	1	1571
		Wavelet_Coif3-HLL_gldm_GrayLevelVariance	FALSE	0.005605908	1	1570	0.014942445	1	1662	0.930111963	1	2304	0.353306456	1	2074	0.94464551	1	2309	0.478364009	1	2134
		Wavelet_Coif3-HLL_gldm_HighGrayLevelEmphasis	TRUE	5.92845E-14	9.45587E-11	273	0.000177313	0.282813705	1302	9.61969E-09	1.53434E-05	758	1.74392E-13	2.78155E-10	312	0.001895198	1	1478	1.30949E-06	0.002088643	1004
		Wavelet_Coif3-HLL_gldm_LargeDependenceEmphasis	FALSE	1.8133E-11	2.89221E-08	489	2.40563E-10	3.83698E-07	595	0.747769965	1	2240	0.271354974	1	2027	0.826447574	1	2268	0.277775386	1	2031
		Wavelet_Coif3-HLL_gldm_LargeDependenceHighGrayLevelEmphasis	TRUE	4.69872E-17	7.49446E-14	31	2.03381E-14	3.24392E-11	235	2.70406E-11	4.31298E-08	505	7.10699E-12	1.13357E-08	452	0.717517273	1	2229	4.2933E-09	6.84781E-06	721
		Wavelet_Coif3-HLL_gldm_LargeDependenceLowGrayLevelEmphasis	FALSE	8.55104E-05	0.136389057	1253	0.775616375	1	2250	0.967935548	1	2317	0.43850874	1	2116	0.997084271	1	2327	0.421426789	1	2108
		Wavelet_Coif3-HLL_gldm_LowGrayLevelEmphasis	FALSE	2.16973E-13	3.46072E-10	320	3.01193E-09	4.80404E-06	705	0.722986518	1	2231	0.467098637	1	2129	0.603819901	1	2186	0.476099125	1	2133
		Wavelet_Coif3-HLL_gldm_SmallDependenceEmphasis	FALSE	0.000124522	0.198612192	1278	0.001552786	1	1462	0.829295192	1	2269	0.378486223	1	2087	0.915601686	1	2299	0.423540439	1	2109
		Wavelet_Coif3-HLL_gldm_SmallDependenceHighGrayLevelEmphasis	TRUE	8.09174E-09	1.29063E-05	750	0.002306037	1	1494	5.66073E-06	0.009028867	1086	2.98739E-11	4.76488E-08	509	0.012693504	1	1646	0.00417036	1	1544
		Wavelet_Coif3-HLL_gldm_SmallDependenceLowGrayLevelEmphasis	FALSE	2.21387E-14	3.53112E-11	238	7.04152E-08	0.000112312	853	0.50365957	1	2145	0.933017355	1	2305	0.218132202	1	1991	0.460410674	1	2126
	GLRLM	Wavelet_Coif3-HLL_glrlm_GrayLevelNonUniformity	FALSE	3.44076E-09	5.48801E-06	711	1.48106E-07	0.000236229	890	0.388455124	1	2092	0.116951316	1	1899	0.309674942	1	2050	0.006130228	1	1578
		Wavelet_Coif3-HLL_glrlm_GrayLevelNonUniformityNormalized	FALSE	7.78428E-11	1.24159E-07	548	2.75507E-09	4.39434E-06	701	0.517750523	1	2151	0.155163637	1	1939	0.695787227	1	2221	0.338350549	1	2066
		Wavelet_Coif3-HLL_glrlm_GrayLevelVariance	FALSE	0.094913468	1	1871	0.072770625	1	1837	0.988337474	1	2324	0.097104285	1	1874	0.869380882	1	2283	0.327404457	1	2060
		Wavelet_Coif3-HLL_glrlm_HighGrayLevelRunEmphasis	TRUE	5.76521E-14	9.19552E-11	272	5.06449E-05	0.080778641	1219	9.61969E-09	1.53434E-05	758	1.79235E-13	2.8588E-10	313	0.001848881	1	1476	1.28563E-06	0.002050587	1003
		Wavelet_Coif3-HLL_glrlm_LongRunEmphasis	FALSE	1.34077E-11	2.13852E-08	477	4.98306E-10	7.94799E-07	626	0.392488192	1	2094	0.127546784	1	1911	0.637348798	1	2199	0.268183186	1	2025
		Wavelet_Coif3-HLL_glrlm_LongRunHighGrayLevelEmphasis	TRUE	6.02342E-17	9.60735E-14	39	5.15706E-14	8.22552E-11	268	2.21526E-11	3.53334E-08	497	1.42033E-12	2.26543E-09	390	0.668973778	1	2211	8.88346E-10	1.41691E-06	651
		Wavelet_Coif3-HLL_glrlm_LongRunLowGrayLevelEmphasis	FALSE	3.10074E-05	0.049456814	1188	0.909805691	1	2297	0.988337474	1	2324	0.355204905	1	2075	0.950464366	1	2311	0.30794064	1	2049
		Wavelet_Coif3-HLL_glrlm_LowGrayLevelRunEmphasis	FALSE	2.69811E-13	4.30348E-10	328	8.27062E-09	1.31916E-05	751	0.73672114	1	2236	0.576086955	1	2175	0.645196229	1	2202	0.591140737	1	2181
		Wavelet_Coif3-HLL_glrlm_RunEntropy	FALSE	8.88146E-10	1.41659E-06	651	0.02347143	1	1708	0.160538263	1	1944	0.703909544	1	2224	0.215412683	1	1989	0.731218417	1	2234
		Wavelet_Coif3-HLL_glrlm_RunLengthNonUniformity	FALSE	4.23113E-10	6.74865E-07	619	5.96317E-06	0.009511251	1089	0.601273871	1	2185	0.013777781	1	1654	0.561214295	1	2169	0.245206692	1	2010
		Wavelet_Coif3-HLL_glrlm_RunLengthNonUniformityNormalized	FALSE	3.7321E-11	5.9527E-08	518	8.29161E-10	1.32251E-06	648	0.722986518	1	2231	0.276160667	1	2030	0.849289327	1	2276	0.327404457	1	2060
		Wavelet_Coif3-HLL_glrlm_RunPercentage	FALSE	2.57278E-11	4.10359E-08	503	5.22052E-10	8.32673E-07	628	0.73672114	1	2236	0.251194202	1	2014	0.857888803	1	2279	0.309677768	1	2050
		Wavelet_Coif3-HLL_glrlm_RunVariance	FALSE	7.67091E-12	1.22351E-08	455	4.3322E-10	6.90986E-07	620	0.455979325	1	2124	0.134063782	1	1918	0.679650915	1	2215	0.242250782	1	2008
		Wavelet_Coif3-HLL_glrlm_ShortRunEmphasis	FALSE	4.01892E-11	6.41018E-08	521	8.68174E-10	1.38474E-06	650	0.568626276	1	2172	0.215415364	1	1989	0.812244415	1	2263	0.34205087	1	2068
		Wavelet_Coif3-HLL_glrlm_ShortRunHighGrayLevelEmphasis	TRUE	3.35234E-13	5.34699E-10	336	0.000470212	0.749988905	1371	3.34665E-06	0.005337905	1056	1.99908E-12	3.18853E-09	403	0.003004104	1	1516	2.282E-05	0.036397871	1169
		Wavelet_Coif3-HLL_glrlm_ShortRunLowGrayLevelEmphasis	FALSE	1.5199E-13	2.42424E-10	307	8.68174E-10	1.38474E-06	650	0.417215377	1	2106	0.933017355	1	2305	0.322015772	1	2057	0.747771389	1	2240
	GLSZM	Wavelet_Coif3-HLL_glszm_GrayLevelNonUniformity	FALSE	0.004074894	1	1542	0.026295407	1	1720	0.184666663	1	1965	0.10160305	1	1880	0.413031558	1	2104	9.50847E-05	0.151660129	1260
		Wavelet_Coif3-HLL_glszm_GrayLevelNonUniformityNormalized	FALSE	0.035619817	1	1753	0.938829964	1	2307	0.976677441	1	2320	0.962109916	1	2315	0.985422135	1	2323	0.921403154	1	2301
		Wavelet_Coif3-HLL_glszm_GrayLevelVariance	TRUE	2.683E-05	0.042793917	1179	0.02347143	1	1708	0.023922148	1	1710	5.33892E-07	0.000851558	956	0.515387948	1	2150	0.005928752	1	1575
		Wavelet_Coif3-HLL_glszm_HighGrayLevelZoneEmphasis	TRUE	2.93826E-14	4.68653E-11	248	3.36596E-09	5.36871E-06	710	1.27192E-08	2.02871E-05	771	1.69677E-13	2.70635E-10	311	0.003040006	1	1517	3.06039E-07	0.000488133	927
		Wavelet_Coif3-HLL_glszm_LargeAreaEmphasis	TRUE	1.66777E-07	0.00026601	896	2.39596E-05	0.038215557	1172	0.021529089	1	1699	2.30375E-09	3.67448E-06	693	0.311418531	1	2051	5.62575E-08	8.97308E-05	842
		Wavelet_Coif3-HLL_glszm_LargeAreaHighGrayLevelEmphasis	TRUE	3.44061E-17	5.48777E-14	21	7.62006E-14	1.2154E-10	282	1.24293E-11	1.98247E-08	474	6.56585E-11	1.04725E-07	541	0.112743811	1	1894	5.95469E-11	9.49773E-08	537
		Wavelet_Coif3-HLL_glszm_LargeAreaLowGrayLevelEmphasis	FALSE	0.382453054	1	2089	0.374542082	1	2085	0.674304148	1	2213	0.001552786	1	1462	0.703907896	1	2224	0.000784201	1	1409
		Wavelet_Coif3-HLL_glszm_LowGrayLevelZoneEmphasis	FALSE	2.21913E-12	3.53951E-09	407	1.10925E-06	0.001769247	995	0.71478805	1	2228	1	1	2328	0.728469951	1	2233	0.959197745	1	2314
		Wavelet_Coif3-HLL_glszm_SizeZoneNonUniformity	FALSE	0.860759099	1	2280	0.257282983	1	2018	0.976677441	1	2320	0.276160667	1	2030	0.959197507	1	2314	0.668975597	1	2211
		Wavelet_Coif3-HLL_glszm_SizeZoneNonUniformityNormalized	FALSE	8.54956E-07	0.001363655	981	8.79975E-08	0.000140356	864	0.492073625	1	2140	0.496693289	1	2142	0.436349298	1	2115	0.517752969	1	2151
		Wavelet_Coif3-HLL_glszm_SmallAreaEmphasis	FALSE	6.95618E-07	0.001109511	970	7.2066E-06	0.011494519	1100	0.513030982	1	2149	0.714789644	1	2228	0.447194162	1	2120	0.661011334	1	2208
		Wavelet_Coif3-HLL_glszm_SmallAreaHighGrayLevelEmphasis	TRUE	9.95142E-15	1.58725E-11	210	1.6811E-09	2.68136E-06	679	5.46639E-09	8.71889E-06	732	1.08508E-14	1.7307E-11	213	0.001334276	1	1450	2.51968E-07	0.000401889	917
		Wavelet_Coif3-HLL_glszm_SmallAreaLowGrayLevelEmphasis	FALSE	1.06654E-10	1.70114E-07	561	0.000421234	0.67186889	1363	0.510679643	1	2148	0.976677577	1	2320	0.58861707	1	2180	0.950464655	1	2311
		Wavelet_Coif3-HLL_glszm_ZoneEntropy	FALSE	0.489773714	1	2139	0.000351682	0.560932518	1350	0.950464366	1	2311	0.632141595	1	2197	0.962109695	1	2315	0.73396881	1	2235
		Wavelet_Coif3-HLL_glszm_ZonePercentage	FALSE	0.012824709	1	1647	0.011002365	1	1595	0.668973778	1	2211	0.714789644	1	2228	0.883784593	1	2288	0.832146021	1	2270
		Wavelet_Coif3-HLL_glszm_ZoneVariance	TRUE	1.70108E-07	0.000271322	897	2.64033E-05	0.042113249	1178	0.020712071	1	1695	2.25267E-09	3.59301E-06	692	0.307937813	1	2049	5.62575E-08	8.97308E-05	842
	NGTDM	Wavelet_Coif3-HLL_ngtdm_Busyness	FALSE	5.58743E-09	8.91195E-06	733	5.22512E-05	0.083340727	1221	0.637348798	1	2199	0.000992889	1	1427	0.546532102	1	2163	7.45513E-05	0.118909262	1244
		Wavelet_Coif3-HLL_ngtdm_Coarseness	FALSE	3.68155E-14	5.87208E-11	256	9.34874E-08	0.000149112	867	0.878018223	1	2286	0.01132726	1	1635	0.99125297	1	2325	0.045618308	1	1781
		Wavelet_Coif3-HLL_ngtdm_Complexity	TRUE	4.14146E-12	6.60563E-09	431	4.26144E-05	0.067969908	1208	0.001368589	1	1452	8.58045E-11	1.36858E-07	552	0.177514152	1	1959	2.06507E-06	0.00329379	1029
		Wavelet_Coif3-HLL_ngtdm_Contrast	FALSE	2.79056E-16	4.45094E-13	89	1.09031E-12	1.73904E-09	380	0.427783603	1	2111	0.423540439	1	2109	0.12482817	1	1908	0.042545706	1	1773
		Wavelet_Coif3-HLL_ngtdm_Strength	TRUE	2.77417E-15	4.4248E-12	166	2.52231E-10	4.02308E-07	597	8.29491E-05	0.132303831	1251	4.16581E-07	0.000664447	943	2.77182E-10	4.42105E-07	601	0.000568919	0.907425136	1385
Wavelet Coiflet 3 - HLH	First Order	Wavelet_Coif3-HLH_firstorder_10Percentile	FALSE	2.52874E-12	4.03335E-09	412	6.4155E-12	1.02327E-08	448	0.728469951	1	2233	0.19833231	1	1976	0.650449959	1	2204	0.080046751	1	1849
		Wavelet_Coif3-HLH_firstorder_90Percentile	FALSE	2.52874E-12	4.03335E-09	412	6.75258E-12	1.07704E-08	450	0.690392694	1	2219	0.174018724	1	1956	0.671636899	1	2212	0.107857792	1	1888
		Wavelet_Coif3-HLH_firstorder_Energy	TRUE	3.94429E-10	6.29114E-07	616	3.34709E-06	0.005338607	1056	0.58610055	1	2179	0.289258288	1	2038	0.329209838	1	2061	1.23916E-06	0.001976466	1001
		Wavelet_Coif3-HLH_firstorder_Entropy	FALSE	1.68074E-09	2.68078E-06	679	1.38571E-08	2.21022E-05	775	0.685013843	1	2217	0.263473425	1	2022	0.553849376	1	2166	0.127546784	1	1911
		Wavelet_Coif3-HLH_firstorder_InterquartileRange	FALSE	1.08998E-12	1.73852E-09	380	1.24463E-12	1.98519E-09	385	0.679650915	1	2215	0.154104825	1	1938	0.65836316	1	2207	0.08858513	1	1862
		Wavelet_Coif3-HLH_firstorder_Kurtosis	TRUE	1.78053E-17	2.83995E-14	0	4.01363E-16	6.40174E-13	101	5.42621E-15	8.65481E-12	189	0.725728015	1	2232	1.23899E-06	0.001976184	1001	3.40688E-06	0.005433974	1057
		Wavelet_Coif3-HLH_firstorder_Maximum	FALSE	1.1493E-12	1.83313E-09	382	0.00211723	1	1487	0.445013007	1	2119	0.449384032	1	2121	0.820759216	1	2266	0.563682033	1	2170
		Wavelet_Coif3-HLH_firstorder_MeanAbsoluteDeviation	FALSE	4.24984E-12	6.7785E-09	432	1.27509E-11	2.03376E-08	475	0.709340563	1	2226	0.194534747	1	1973	0.674304148	1	2213	0.125730786	1	1909
		Wavelet_Coif3-HLH_firstorder_Mean	FALSE	0.50833395	1	2147	0.434201343	1	2114	0.956285864	1	2313	0.388457896	1	2092	0.953374804	1	2312	0.480634768	1	2135
		Wavelet_Coif3-HLH_firstorder_Median	FALSE	0.212717676	1	1987	0.462634024	1	2127	0.855020384	1	2278	0.248187814	1	2012	0.895335523	1	2292	0.272950465	1	2028
		Wavelet_Coif3-HLH_firstorder_Minimum	FALSE	6.39775E-13	1.02044E-09	360	0.114413543	1	1896	0.529646891	1	2156	0.494381839	1	2141	0.834997126	1	2271	0.98542222	1	2323
		Wavelet_Coif3-HLH_firstorder_Range	FALSE	1.74336E-13	2.78065E-10	312	0.012180991	1	1642	0.548965864	1	2164	0.666316646	1	2210	0.904013444	1	2295	0.767230711	1	2247
		Wavelet_Coif3-HLH_firstorder_RobustMeanAbsoluteDeviation	FALSE	1.38293E-12	2.20578E-09	389	1.11959E-12	1.78575E-09	381	0.687701293	1	2218	0.170527507	1	1953	0.661009477	1	2208	0.094194617	1	1870
		Wavelet_Coif3-HLH_firstorder_RootMeanSquared	FALSE	5.95317E-11	9.4953E-08	537	2.81721E-09	4.49345E-06	702	0.626948606	1	2195	0.210049766	1	1985	0.619196686	1	2192	0.197060479	1	1975
		Wavelet_Coif3-HLH_firstorder_Skewness	TRUE	0.00417014	1	1544	0.118667577	1	1901	1.45808E-10	2.32563E-07	574	0.478364009	1	2134	4.67476E-06	0.007456247	1075	0.901120052	1	2294
		Wavelet_Coif3-HLH_firstorder_TotalEnergy	TRUE	3.94429E-10	6.29114E-07	616	3.52955E-06	0.005629633	1059	0.593665023	1	2182	0.290924438	1	2039	0.325599883	1	2059	1.21654E-06	0.001940379	1000
		Wavelet_Coif3-HLH_firstorder_Uniformity	FALSE	1.11668E-09	1.7811E-06	661	1.29968E-08	2.07298E-05	772	0.676975497	1	2214	0.386450864	1	2091	0.438506065	1	2116	0.131239923	1	1915
		Wavelet_Coif3-HLH_firstorder_Variance	FALSE	2.13601E-10	3.40694E-07	590	2.7256E-08	4.34734E-05	807	0.596196407	1	2183	0.301056715	1	2045	0.473837548	1	2132	0.193280825	1	1972
	GLCM	Wavelet_Coif3-HLH_glcm_Autocorrelation	FALSE	3.7637E-10	6.0031E-07	614	0.183462551	1	1964	0.161630372	1	1945	0.024382025	1	1712	0.92430479	1	2302	0.62177805	1	2193
		Wavelet_Coif3-HLH_glcm_ClusterProminence	FALSE	0.001803607	1	1474	0.000763718	1	1407	0.072187876	1	1836	0.000134127	0.213932566	1283	0.199607508	1	1977	0.097842819	1	1875
		Wavelet_Coif3-HLH_glcm_ClusterShade	FALSE	0.58610055	1	2179	0.840708665	1	2273	0.039298906	1	1764	0.01479227	1	1661	0.218132202	1	1991	0.781221455	1	2252
		Wavelet_Coif3-HLH_glcm_ClusterTendency	FALSE	9.92912E-08	0.000158369	870	3.00893E-06	0.004799245	1050	0.476096548	1	2133	0.462634024	1	2127	0.290921619	1	2039	0.301056715	1	2045
		Wavelet_Coif3-HLH_glcm_Contrast	FALSE	3.80287E-08	6.06558E-05	823	1.75436E-06	0.002798205	1020	0.460408054	1	2126	0.449384032	1	2121	0.277772578	1	2031	0.255751271	1	2017
		Wavelet_Coif3-HLH_glcm_Correlation	FALSE	3.15022E-16	5.0246E-13	93	5.59754E-10	8.92807E-07	631	0.382453054	1	2089	0.092058573	1	1867	0.212717676	1	1987	0.024614402	1	1713
		Wavelet_Coif3-HLH_glcm_DifferenceAverage	FALSE	9.29862E-10	1.48313E-06	653	1.54156E-08	2.45878E-05	780	0.581082539	1	2177	0.33467603	1	2064	0.347646875	1	2071	0.100085376	1	1878
		Wavelet_Coif3-HLH_glcm_DifferenceEntropy	FALSE	5.34212E-10	8.52068E-07	629	4.89945E-09	7.81463E-06	727	0.695787227	1	2221	0.245206692	1	2010	0.571107293	1	2173	0.125730786	1	1909
		Wavelet_Coif3-HLH_glcm_DifferenceVariance	FALSE	1.06878E-06	0.001704697	993	7.71744E-06	0.012309313	1104	0.447194162	1	2120	0.611488968	1	2189	0.380465065	1	2088	0.717518853	1	2229
		Wavelet_Coif3-HLH_glcm_Id	FALSE	2.18735E-10	3.48883E-07	591	3.93043E-09	6.26904E-06	717	0.679650915	1	2215	0.390471262	1	2093	0.360935981	1	2078	0.100841938	1	1879
		Wavelet_Coif3-HLH_glcm_Idm	FALSE	2.5217E-10	4.02212E-07	597	4.01825E-09	6.40911E-06	718	0.666314815	1	2210	0.378486223	1	2087	0.353303643	1	2074	0.094915399	1	1871
		Wavelet_Coif3-HLH_glcm_Idmn	FALSE	4.95409E-16	7.90177E-13	108	0.000457493	0.729701243	1369	0.551404963	1	2165	0.290924438	1	2039	0.455979325	1	2124	0.265036963	1	2023
		Wavelet_Coif3-HLH_glcm_Idn	FALSE	1.34236E-16	2.14107E-13	65	3.15113E-05	0.050260563	1189	0.360935981	1	2078	0.266606881	1	2024	0.332845627	1	2063	0.252706887	1	2015
		Wavelet_Coif3-HLH_glcm_Imc1	FALSE	0.002391437	1	1497	0.082599622	1	1853	0.938829607	1	2307	0.944645833	1	2309	0.789649143	1	2255	0.800927098	1	2259
		Wavelet_Coif3-HLH_glcm_Imc2	FALSE	0.037254019	1	1758	0.561216583	1	2169	0.947554589	1	2310	0.714789644	1	2228	0.915601686	1	2299	0.714789644	1	2228
		Wavelet_Coif3-HLH_glcm_InverseVariance	FALSE	2.24051E-07	0.000357361	911	8.42286E-05	0.13434461	1252	0.382453054	1	2089	0.139852221	1	1924	0.269763084	1	2026	0.080678879	1	1850
		Wavelet_Coif3-HLH_glcm_JointAverage	FALSE	1.73502E-07	0.000276736	898	0.362862913	1	2079	0.35710697	1	2076	0.093478231	1	1869	0.314925095	1	2053	0.106266226	1	1886
		Wavelet_Coif3-HLH_glcm_JointEnergy	FALSE	2.08584E-10	3.32692E-07	589	1.79892E-09	2.86927E-06	682	0.73947835	1	2237	0.327404457	1	2060	0.460408054	1	2126	0.124830373	1	1908
		Wavelet_Coif3-HLH_glcm_JointEntropy	FALSE	5.86216E-10	9.35015E-07	633	5.00827E-09	7.98818E-06	728	0.679650915	1	2215	0.260365473	1	2020	0.532042731	1	2157	0.106266226	1	1886
		Wavelet_Coif3-HLH_glcm_MCC	FALSE	2.65543E-06	0.004235418	1043	0.000421234	0.67186889	1363	0.581082539	1	2177	0.539265407	1	2160	0.52487172	1	2154	0.578583315	1	2176
		Wavelet_Coif3-HLH_glcm_MaximumProbability	FALSE	1.24293E-11	1.98247E-08	474	4.03867E-10	6.44168E-07	617	0.637348798	1	2199	0.404741605	1	2100	0.307937813	1	2049	0.10160305	1	1880
		Wavelet_Coif3-HLH_glcm_SumAverage	FALSE	1.73502E-07	0.000276736	898	0.362862913	1	2079	0.35710697	1	2076	0.093478231	1	1869	0.314925095	1	2053	0.106266226	1	1886
		Wavelet_Coif3-HLH_glcm_SumEntropy	FALSE	8.67978E-10	1.38443E-06	650	8.09335E-09	1.29089E-05	750	0.693088015	1	2220	0.258821048	1	2019	0.541680668	1	2161	0.122159286	1	1905
		Wavelet_Coif3-HLH_glcm_SumSquares	FALSE	6.48962E-08	0.000103509	849	2.17992E-06	0.003476969	1032	0.469337277	1	2130	0.445015666	1	2119	0.28925547	1	2038	0.279396521	1	2032
	GLDM	Wavelet_Coif3-HLH_gldm_DependenceEntropy	FALSE	5.71107E-09	9.10916E-06	734	1.51075E-07	0.000240964	891	0.650449959	1	2204	0.301056715	1	2045	0.58861707	1	2180	0.251194202	1	2014
		Wavelet_Coif3-HLH_gldm_DependenceNonUniformity	TRUE	5.34212E-10	8.52068E-07	629	6.49076E-08	0.000103528	849	0.901119478	1	2294	5.76036E-06	0.009187768	1087	0.904013444	1	2295	7.85046E-06	0.01252148	1105
		Wavelet_Coif3-HLH_gldm_DependenceNonUniformityNormalized	FALSE	5.21931E-10	8.3248E-07	628	2.96333E-08	4.72651E-05	811	0.706622361	1	2225	0.473840131	1	2132	0.626948606	1	2195	0.432056844	1	2113
		Wavelet_Coif3-HLH_gldm_DependenceVariance	FALSE	9.5143E-10	1.51753E-06	654	6.51211E-09	1.03868E-05	740	0.728469951	1	2233	0.289258288	1	2038	0.71206247	1	2227	0.258821048	1	2019
		Wavelet_Coif3-HLH_gldm_GrayLevelNonUniformity	FALSE	2.30326E-09	3.6737E-06	693	3.37118E-07	0.000537704	932	0.94173717	1	2308	0.000146559	0.23376137	1289	0.803751758	1	2260	0.000257874	0.411308599	1328
		Wavelet_Coif3-HLH_gldm_GrayLevelVariance	FALSE	1.76962E-07	0.000282255	899	3.78722E-06	0.006041406	1063	0.471584453	1	2131	0.46486335	1	2128	0.302765185	1	2046	0.279396521	1	2032
		Wavelet_Coif3-HLH_gldm_HighGrayLevelEmphasis	FALSE	0.001300759	1	1448	0.46486335	1	2128	0.149922047	1	1934	0.024382025	1	1712	0.915601686	1	2299	0.889557769	1	2290
		Wavelet_Coif3-HLH_gldm_LargeDependenceEmphasis	FALSE	1.49344E-10	2.38203E-07	575	5.58857E-09	8.91377E-06	733	0.722986518	1	2231	0.548968199	1	2164	0.388455124	1	2092	0.323807424	1	2058
		Wavelet_Coif3-HLH_gldm_LargeDependenceHighGrayLevelEmphasis	FALSE	1.83781E-15	2.93131E-12	152	0.000220313	0.35139885	1317	0.031945414	1	1741	0.00135142	1	1451	0.73947835	1	2237	0.421426789	1	2108
		Wavelet_Coif3-HLH_gldm_LargeDependenceLowGrayLevelEmphasis	FALSE	0.050575079	1	1793	0.023248447	1	1707	0.415120353	1	2105	0.093478231	1	1869	0.421424076	1	2108	0.083900589	1	1855
		Wavelet_Coif3-HLH_gldm_LowGrayLevelEmphasis	FALSE	7.40866E-14	1.18168E-10	281	0.008602762	1	1609	0.139849906	1	1924	0.08790328	1	1861	0.141821526	1	1926	0.072770625	1	1837
		Wavelet_Coif3-HLH_gldm_SmallDependenceEmphasis	FALSE	1.52084E-11	2.42574E-08	482	1.23277E-10	1.96627E-07	567	0.728469951	1	2233	0.255751271	1	2017	0.621776014	1	2193	0.099333347	1	1877
		Wavelet_Coif3-HLH_gldm_SmallDependenceHighGrayLevelEmphasis	FALSE	0.421424076	1	2108	0.004468353	1	1550	0.478361439	1	2134	0.402684098	1	2099	0.912702845	1	2298	0.322018599	1	2057
		Wavelet_Coif3-HLH_gldm_SmallDependenceLowGrayLevelEmphasis	FALSE	4.97352E-15	7.93277E-12	186	0.000642222	1	1394	0.690392694	1	2219	0.812245491	1	2263	0.058853017	1	1811	0.601275996	1	2185
	GLRLM	Wavelet_Coif3-HLH_glrlm_GrayLevelNonUniformity	FALSE	6.51079E-09	1.03847E-05	740	1.01109E-06	0.001612687	990	0.653083373	1	2205	0.00024357	0.388493523	1324	0.568626276	1	2172	0.000134127	0.213932566	1283
		Wavelet_Coif3-HLH_glrlm_GrayLevelNonUniformityNormalized	FALSE	6.14039E-10	9.79392E-07	635	4.10797E-09	6.55221E-06	719	0.73947835	1	2237	0.314927922	1	2053	0.527256548	1	2155	0.132176039	1	1916
		Wavelet_Coif3-HLH_glrlm_GrayLevelVariance	FALSE	1.54076E-07	0.000245751	892	3.2883E-06	0.005244846	1055	0.473837548	1	2132	0.467098637	1	2129	0.374539291	1	2085	0.408875433	1	2102
		Wavelet_Coif3-HLH_glrlm_HighGrayLevelRunEmphasis	FALSE	0.001737603	1	1471	0.374542082	1	2085	0.139849906	1	1924	0.024382025	1	1712	0.915601686	1	2299	0.837852303	1	2272
		Wavelet_Coif3-HLH_glrlm_LongRunEmphasis	FALSE	3.1187E-10	4.97433E-07	606	9.21513E-09	1.46981E-05	756	0.73947835	1	2237	0.447196816	1	2120	0.616622038	1	2191	0.322018599	1	2057
		Wavelet_Coif3-HLH_glrlm_LongRunHighGrayLevelEmphasis	FALSE	2.06781E-15	3.29815E-12	156	0.000318834	0.508540797	1343	0.004895423	1	1558	0.000773897	1	1408	0.709340563	1	2226	0.297653489	1	2043
		Wavelet_Coif3-HLH_glrlm_LongRunLowGrayLevelEmphasis	FALSE	0.063918715	1	1821	0.022374807	1	1703	0.40473886	1	2100	0.083900589	1	1855	0.429915799	1	2112	0.069340852	1	1831
		Wavelet_Coif3-HLH_glrlm_LowGrayLevelRunEmphasis	FALSE	6.81527E-14	1.08704E-10	278	0.009572353	1	1619	0.169432713	1	1952	0.116951316	1	1899	0.192030253	1	1971	0.100841938	1	1879
		Wavelet_Coif3-HLH_glrlm_RunEntropy	FALSE	3.28787E-06	0.005244155	1055	3.92349E-06	0.00625797	1065	0.63996015	1	2200	0.215415364	1	1989	0.663660038	1	2209	0.223647783	1	1995
		Wavelet_Coif3-HLH_glrlm_RunLengthNonUniformity	FALSE	8.88146E-10	1.41659E-06	651	6.09652E-07	0.000972395	963	0.935922859	1	2306	0.00149529	1	1459	0.970849136	1	2318	0.674305941	1	2213
		Wavelet_Coif3-HLH_glrlm_RunLengthNonUniformityNormalized	FALSE	1.94215E-10	3.09774E-07	586	2.01338E-09	3.21134E-06	687	0.64257597	1	2201	0.410951736	1	2103	0.400630121	1	2098	0.195794628	1	1974
		Wavelet_Coif3-HLH_glrlm_RunPercentage	FALSE	1.76552E-10	2.81601E-07	582	3.14897E-09	5.0226E-06	707	0.663660038	1	2209	0.434201343	1	2114	0.440668947	1	2117	0.243725589	1	2009
		Wavelet_Coif3-HLH_glrlm_RunVariance	FALSE	9.00446E-11	1.43621E-07	554	1.04875E-08	1.67275E-05	762	0.687701293	1	2218	0.517752969	1	2151	0.553849376	1	2166	0.413034288	1	2104
		Wavelet_Coif3-HLH_glrlm_ShortRunEmphasis	FALSE	1.0191E-09	1.62547E-06	657	5.96761E-09	9.51834E-06	736	0.709340563	1	2226	0.360938787	1	2078	0.532042731	1	2157	0.219503871	1	1992
		Wavelet_Coif3-HLH_glrlm_ShortRunHighGrayLevelEmphasis	FALSE	0.001781354	1	1473	0.239320027	1	2006	0.28925547	1	2038	0.106266226	1	1886	0.576084726	1	2175	0.849290197	1	2276
		Wavelet_Coif3-HLH_glrlm_ShortRunLowGrayLevelEmphasis	FALSE	3.89456E-14	6.21183E-11	258	0.00937098	1	1617	0.34204805	1	2068	0.147866935	1	1932	0.406802645	1	2101	0.150961328	1	1935
	GLSZM	Wavelet_Coif3-HLH_glszm_GrayLevelNonUniformity	TRUE	5.22303E-12	8.33073E-09	440	1.13118E-10	1.80423E-07	483	0.325599883	1	2059	2.28164E-05	0.036392146	1169	0.761653954	1	2245	0.223643918	1	1995
		Wavelet_Coif3-HLH_glszm_GrayLevelNonUniformityNormalized	FALSE	3.63719E-05	0.058013104	1198	0.000104676	0.166958903	1173	0.761653954	1	2245	0.406805386	1	2101	0.786836709	1	2254	0.573595689	1	2174
		Wavelet_Coif3-HLH_glszm_GrayLevelVariance	FALSE	0.004571654	1	1552	0.723022358	1	2093	0.015713037	1	1667	0.496693289	1	2142	0.347646875	1	2071	0.038608071	1	1762

	GLSZM	Wavelet_Coif3-HLH_glszm_HighGrayLevelZoneEmphasis	FALSE	2.35293E-08	3.75292E-05	800	0.508644468	1	2013	[illegible]	[illegible]	[illegible]	[illegible]	[illegible]	[illegible]	[illegible]	[illegible]	[illegible]	[illegible]	[illegible]	[illegible]
		Wavelet_Coif3-HLH_glszm_LargeAreaEmphasis	TRUE	1.68319E-10	2.68469E-07	580	2.63477E-09	4.20246E-06	699	[illegible]	[illegible]	[illegible]	[illegible]	[illegible]	[illegible]	[illegible]	[illegible]	[illegible]	[illegible]	[illegible]	[illegible]
		Wavelet_Coif3-HLH_glszm_LargeAreaHighGrayLevelEmphasis	TRUE	1.42718E-16	2.27636E-13	67	5.22052E-10	8.32673E-07	628	[illegible]	[illegible]	[illegible]	[illegible]	[illegible]	[illegible]	[illegible]	[illegible]	[illegible]	[illegible]	[illegible]	[illegible]
		Wavelet_Coif3-HLH_glszm_LargeAreaLowGrayLevelEmphasis	TRUE	0.000489874	0.781349579	1374	8.81546E-05	0.140606643	1255	[illegible]	[illegible]	[illegible]	[illegible]	[illegible]	[illegible]	[illegible]	[illegible]	[illegible]	[illegible]	[illegible]	[illegible]
		Wavelet_Coif3-HLH_glszm_LowGrayLevelZoneEmphasis	FALSE	5.10061E-06	0.008135469	1080	0.731652921	1	2096	[illegible]	[illegible]	[illegible]	[illegible]	[illegible]	[illegible]	[illegible]	[illegible]	[illegible]	[illegible]	[illegible]	[illegible]
		Wavelet_Coif3-HLH_glszm_SizeZoneNonUniformity	TRUE	8.07247E-12	1.28756E-08	457	4.40813E-09	7.03097E-06	632	[illegible]	[illegible]	[illegible]	[illegible]	[illegible]	[illegible]	[illegible]	[illegible]	[illegible]	[illegible]	[illegible]	[illegible]
		Wavelet_Coif3-HLH_glszm_SizeZoneNonUniformityNormalized	FALSE	0.009777148	1	1621	0.005939794	1	1467.5	[illegible]	[illegible]	[illegible]	[illegible]	[illegible]	[illegible]	[illegible]	[illegible]	[illegible]	[illegible]	[illegible]	[illegible]
		Wavelet_Coif3-HLH_glszm_SmallAreaEmphasis	FALSE	0.001918754	1	1479	0.006479757	1	1583	[illegible]	[illegible]	[illegible]	[illegible]	[illegible]	[illegible]	[illegible]	[illegible]	[illegible]	[illegible]	[illegible]	[illegible]
		Wavelet_Coif3-HLH_glszm_SmallAreaHighGrayLevelEmphasis	FALSE	2.27127E-11	3.62268E-08	498	0.026543471	1	1721	[illegible]	[illegible]	[illegible]	[illegible]	[illegible]	[illegible]	[illegible]	[illegible]	[illegible]	[illegible]	[illegible]	[illegible]
		Wavelet_Coif3-HLH_glszm_SmallAreaLowGrayLevelEmphasis	FALSE	0.003381182	1	1526	0.149924427	1	1934	[illegible]	[illegible]	[illegible]	[illegible]	[illegible]	[illegible]	[illegible]	[illegible]	[illegible]	[illegible]	[illegible]	[illegible]
		Wavelet_Coif3-HLH_glszm_ZoneEntropy	FALSE	0.436349298	1	2115	0.150217592	1	1810	[illegible]	[illegible]	[illegible]	[illegible]	[illegible]	[illegible]	[illegible]	[illegible]	[illegible]	[illegible]	[illegible]	[illegible]
		Wavelet_Coif3-HLH_glszm_ZonePercentage	FALSE	1.90652E-11	3.04091E-08	491	2.55378E-11	4.07328E-08	426	[illegible]	[illegible]	[illegible]	[illegible]	[illegible]	[illegible]	[illegible]	[illegible]	[illegible]	[illegible]	[illegible]	[illegible]
		Wavelet_Coif3-HLH_glszm_ZoneVariance	TRUE	1.56669E-10	2.49887E-07	577	8.63655E-09	1.37753E-05	753	[illegible]	[illegible]	[illegible]	[illegible]	[illegible]	[illegible]	[illegible]	[illegible]	[illegible]	[illegible]	[illegible]	[illegible]
	NGTDM	Wavelet_Coif3-HLH_ngtdm_Busyness	FALSE	1.61703E-05	0.025791552	1148	0.855021221	1	2278	[illegible]	[illegible]	[illegible]	[illegible]	[illegible]	[illegible]	[illegible]	[illegible]	[illegible]	[illegible]	[illegible]	[illegible]
		Wavelet_Coif3-HLH_ngtdm_Coarseness	FALSE	7.04028E-08	0.000112293	853	1.99198E-07	0.000317721	905	[illegible]	[illegible]	[illegible]	[illegible]	[illegible]	[illegible]	[illegible]	[illegible]	[illegible]	[illegible]	[illegible]	[illegible]
		Wavelet_Coif3-HLH_ngtdm_Complexity	FALSE	2.17962E-06	0.003476494	1032	0.451577303	1	2122	[illegible]	[illegible]	[illegible]	[illegible]	[illegible]	[illegible]	[illegible]	[illegible]	[illegible]	[illegible]	[illegible]	[illegible]
		Wavelet_Coif3-HLH_ngtdm_Contrast	FALSE	3.15022E-16	5.0246E-13	93	0.000269115	0.42923799	1331	[illegible]	[illegible]	[illegible]	[illegible]	[illegible]	[illegible]	[illegible]	[illegible]	[illegible]	[illegible]	[illegible]	[illegible]
		Wavelet_Coif3-HLH_ngtdm_Strength	TRUE	1.40366E-14	2.23884E-11	222	0.00988165	1	1622	[illegible]	[illegible]	[illegible]	[illegible]	[illegible]	[illegible]	[illegible]	[illegible]	[illegible]	[illegible]	[illegible]	[illegible]
Wavelet Coiflet 3 - HHL	First Order	Wavelet_Coif3-HHL_firstorder_10Percentile	FALSE	0.322015772	1	2057	0.076946218	1	1844	[illegible]	[illegible]	[illegible]	[illegible]	[illegible]	[illegible]	[illegible]	[illegible]	[illegible]	[illegible]	[illegible]	[illegible]
		Wavelet_Coif3-HHL_firstorder_90Percentile	FALSE	0.294273248	1	2041	0.144821246	1	1929	[illegible]	[illegible]	[illegible]	[illegible]	[illegible]	[illegible]	[illegible]	[illegible]	[illegible]	[illegible]	[illegible]	[illegible]
		Wavelet_Coif3-HHL_firstorder_Energy	TRUE	0.010749947	1	1630	0.357109779	1	2076	[illegible]	[illegible]	[illegible]	[illegible]	[illegible]	[illegible]	[illegible]	[illegible]	[illegible]	[illegible]	[illegible]	[illegible]
		Wavelet_Coif3-HHL_firstorder_Entropy	FALSE	2.4669E-15	3.93471E-12	162	8.88342E-10	1.41691E-06	629	[illegible]	[illegible]	[illegible]	[illegible]	[illegible]	[illegible]	[illegible]	[illegible]	[illegible]	[illegible]	[illegible]	[illegible]
		Wavelet_Coif3-HHL_firstorder_InterquartileRange	FALSE	0.619196686	1	2192	0.368673722	1	2082	[illegible]	[illegible]	[illegible]	[illegible]	[illegible]	[illegible]	[illegible]	[illegible]	[illegible]	[illegible]	[illegible]	[illegible]
		Wavelet_Coif3-HHL_firstorder_Kurtosis	TRUE	3.15022E-16	5.0246E-13	93	3.53998E-13	5.64626E-10	338	[illegible]	[illegible]	[illegible]	[illegible]	[illegible]	[illegible]	[illegible]	[illegible]	[illegible]	[illegible]	[illegible]	[illegible]
		Wavelet_Coif3-HHL_firstorder_Maximum	FALSE	1.89629E-17	3.02458E-14	2	1.53001E-10	2.44036E-07	576	[illegible]	[illegible]	[illegible]	[illegible]	[illegible]	[illegible]	[illegible]	[illegible]	[illegible]	[illegible]	[illegible]	[illegible]
		Wavelet_Coif3-HHL_firstorder_MeanAbsoluteDeviation	FALSE	0.153049112	1	1937	0.055978299	1	1805	[illegible]	[illegible]	[illegible]	[illegible]	[illegible]	[illegible]	[illegible]	[illegible]	[illegible]	[illegible]	[illegible]	[illegible]
		Wavelet_Coif3-HHL_firstorder_Mean	FALSE	0.34952603	1	2072	0.001918871	1	1479	[illegible]	[illegible]	[illegible]	[illegible]	[illegible]	[illegible]	[illegible]	[illegible]	[illegible]	[illegible]	[illegible]	[illegible]
		Wavelet_Coif3-HHL_firstorder_Median	FALSE	0.904013444	1	2295	0.132176039	1	1916	[illegible]	[illegible]	[illegible]	[illegible]	[illegible]	[illegible]	[illegible]	[illegible]	[illegible]	[illegible]	[illegible]	[illegible]
		Wavelet_Coif3-HHL_firstorder_Minimum	FALSE	3.54973E-17	5.66183E-14	22	1.7243E-10	2.75026E-07	581	[illegible]	[illegible]	[illegible]	[illegible]	[illegible]	[illegible]	[illegible]	[illegible]	[illegible]	[illegible]	[illegible]	[illegible]
		Wavelet_Coif3-HHL_firstorder_Range	FALSE	1.83751E-17	2.93083E-14	1	1.48343E-11	2.36607E-08	481	[illegible]	[illegible]	[illegible]	[illegible]	[illegible]	[illegible]	[illegible]	[illegible]	[illegible]	[illegible]	[illegible]	[illegible]
		Wavelet_Coif3-HHL_firstorder_RobustMeanAbsoluteDeviation	FALSE	0.556299084	1	2167	0.218134891	1	1991	[illegible]	[illegible]	[illegible]	[illegible]	[illegible]	[illegible]	[illegible]	[illegible]	[illegible]	[illegible]	[illegible]	[illegible]
		Wavelet_Coif3-HHL_firstorder_RootMeanSquared	FALSE	0.00201569	1	1483	0.011209738	1	1634	[illegible]	[illegible]	[illegible]	[illegible]	[illegible]	[illegible]	[illegible]	[illegible]	[illegible]	[illegible]	[illegible]	[illegible]
		Wavelet_Coif3-HHL_firstorder_Skewness	TRUE	0.24818505	1	2012	0.473840131	1	2132	[illegible]	[illegible]	[illegible]	[illegible]	[illegible]	[illegible]	[illegible]	[illegible]	[illegible]	[illegible]	[illegible]	[illegible]
		Wavelet_Coif3-HHL_firstorder_TotalEnergy	TRUE	0.010749947	1	1630	0.357109779	1	2076	[illegible]	[illegible]	[illegible]	[illegible]	[illegible]	[illegible]	[illegible]	[illegible]	[illegible]	[illegible]	[illegible]	[illegible]
		Wavelet_Coif3-HHL_firstorder_Uniformity	FALSE	9.95142E-15	1.58725E-11	210	3.1396E-09	5.00766E-06	684	[illegible]	[illegible]	[illegible]	[illegible]	[illegible]	[illegible]	[illegible]	[illegible]	[illegible]	[illegible]	[illegible]	[illegible]
		Wavelet_Coif3-HHL_firstorder_Variance	FALSE	0.00209132	1	1486	0.010638221	1	1629	[illegible]	[illegible]	[illegible]	[illegible]	[illegible]	[illegible]	[illegible]	[illegible]	[illegible]	[illegible]	[illegible]	[illegible]
	GLCM	Wavelet_Coif3-HHL_glcm_Autocorrelation	TRUE	3.64102E-11	5.80743E-08	517	0.010977937	1	1632	[illegible]	[illegible]	[illegible]	[illegible]	[illegible]	[illegible]	[illegible]	[illegible]	[illegible]	[illegible]	[illegible]	[illegible]
		Wavelet_Coif3-HHL_glcm_ClusterProminence	TRUE	5.9327E-16	9.46266E-13	114	1.71951E-09	2.74262E-06	680	[illegible]	[illegible]	[illegible]	[illegible]	[illegible]	[illegible]	[illegible]	[illegible]	[illegible]	[illegible]	[illegible]	[illegible]
		Wavelet_Coif3-HHL_glcm_ClusterShade	TRUE	0.323804597	1	2058	0.265036963	1	2023	[illegible]	[illegible]	[illegible]	[illegible]	[illegible]	[illegible]	[illegible]	[illegible]	[illegible]	[illegible]	[illegible]	[illegible]
		Wavelet_Coif3-HHL_glcm_ClusterTendency	TRUE	1.49678E-12	2.38737E-09	392	8.55082E-07	0.001363856	981	[illegible]	[illegible]	[illegible]	[illegible]	[illegible]	[illegible]	[illegible]	[illegible]	[illegible]	[illegible]	[illegible]	[illegible]
		Wavelet_Coif3-HHL_glcm_Contrast	TRUE	9.57229E-16	1.52678E-12	130	3.55305E-11	5.66711E-08	516	[illegible]	[illegible]	[illegible]	[illegible]	[illegible]	[illegible]	[illegible]	[illegible]	[illegible]	[illegible]	[illegible]	[illegible]
		Wavelet_Coif3-HHL_glcm_Correlation	FALSE	1.78053E-17	2.83995E-14	0	3.21285E-15	5.12449E-12	171	[illegible]	[illegible]	[illegible]	[illegible]	[illegible]	[illegible]	[illegible]	[illegible]	[illegible]	[illegible]	[illegible]	[illegible]
		Wavelet_Coif3-HHL_glcm_DifferenceAverage	FALSE	4.26153E-16	6.79714E-13	103	1.06178E-12	1.69354E-09	379	[illegible]	[illegible]	[illegible]	[illegible]	[illegible]	[illegible]	[illegible]	[illegible]	[illegible]	[illegible]	[illegible]	[illegible]
		Wavelet_Coif3-HHL_glcm_DifferenceEntropy	FALSE	2.59557E-12	4.13993E-09	413	1.03003E-06	0.001642892	991	[illegible]	[illegible]	[illegible]	[illegible]	[illegible]	[illegible]	[illegible]	[illegible]	[illegible]	[illegible]	[illegible]	[illegible]
		Wavelet_Coif3-HHL_glcm_DifferenceVariance	TRUE	1.61982E-12	2.58361E-09	395	1.99176E-06	0.00317686	1027	[illegible]	[illegible]	[illegible]	[illegible]	[illegible]	[illegible]	[illegible]	[illegible]	[illegible]	[illegible]	[illegible]	[illegible]
		Wavelet_Coif3-HHL_glcm_Id	FALSE	1.71465E-16	2.73487E-13	73	6.09826E-14	9.72673E-11	274	[illegible]	[illegible]	[illegible]	[illegible]	[illegible]	[illegible]	[illegible]	[illegible]	[illegible]	[illegible]	[illegible]	[illegible]
		Wavelet_Coif3-HHL_glcm_Idm	FALSE	3.05624E-16	4.8747E-13	92	2.55604E-13	4.07689E-10	326	[illegible]	[illegible]	[illegible]	[illegible]	[illegible]	[illegible]	[illegible]	[illegible]	[illegible]	[illegible]	[illegible]	[illegible]
		Wavelet_Coif3-HHL_glcm_Idmn	FALSE	6.09622E-14	9.72348E-11	274	0.009674479	1	1620	[illegible]	[illegible]	[illegible]	[illegible]	[illegible]	[illegible]	[illegible]	[illegible]	[illegible]	[illegible]	[illegible]	[illegible]
		Wavelet_Coif3-HHL_glcm_Idn	FALSE	2.77683E-14	4.42904E-11	246	0.010863642	1	1631	[illegible]	[illegible]	[illegible]	[illegible]	[illegible]	[illegible]	[illegible]	[illegible]	[illegible]	[illegible]	[illegible]	[illegible]
		Wavelet_Coif3-HHL_glcm_Imc1	FALSE	1.78053E-17	2.83995E-14	0	1.15191E-16	1.8373E-13	60	[illegible]	[illegible]	[illegible]	[illegible]	[illegible]	[illegible]	[illegible]	[illegible]	[illegible]	[illegible]	[illegible]	[illegible]
		Wavelet_Coif3-HHL_glcm_Imc2	FALSE	1.78053E-17	2.83995E-14	0	4.41711E-17	7.04529E-14	29	[illegible]	[illegible]	[illegible]	[illegible]	[illegible]	[illegible]	[illegible]	[illegible]	[illegible]	[illegible]	[illegible]	[illegible]
		Wavelet_Coif3-HHL_glcm_InverseVariance	FALSE	1.78053E-17	2.83995E-14	0	1.5372E-12	2.45183E-09	393	[illegible]	[illegible]	[illegible]	[illegible]	[illegible]	[illegible]	[illegible]	[illegible]	[illegible]	[illegible]	[illegible]	[illegible]
		Wavelet_Coif3-HHL_glcm_JointAverage	FALSE	4.59195E-12	7.32415E-09	435	0.005181184	1	1563	[illegible]	[illegible]	[illegible]	[illegible]	[illegible]	[illegible]	[illegible]	[illegible]	[illegible]	[illegible]	[illegible]	[illegible]
		Wavelet_Coif3-HHL_glcm_JointEnergy	FALSE	1.30931E-06	0.002088346	1004	0.001942697	1	1480	[illegible]	[illegible]	[illegible]	[illegible]	[illegible]	[illegible]	[illegible]	[illegible]	[illegible]	[illegible]	[illegible]	[illegible]
		Wavelet_Coif3-HHL_glcm_JointEntropy	FALSE	2.55523E-13	4.07559E-10	326	9.93082E-08	0.000158397	870	[illegible]	[illegible]	[illegible]	[illegible]	[illegible]	[illegible]	[illegible]	[illegible]	[illegible]	[illegible]	[illegible]	[illegible]
		Wavelet_Coif3-HHL_glcm_MCC	FALSE	2.28998E-17	3.65252E-14	8	6.49363E-16	1.03573E-12	117	[illegible]	[illegible]	[illegible]	[illegible]	[illegible]	[illegible]	[illegible]	[illegible]	[illegible]	[illegible]	[illegible]	[illegible]
		Wavelet_Coif3-HHL_glcm_MaximumProbability	FALSE	5.39003E-05	0.085971049	1223	0.006697982	1	1586	[illegible]	[illegible]	[illegible]	[illegible]	[illegible]	[illegible]	[illegible]	[illegible]	[illegible]	[illegible]	[illegible]	[illegible]
		Wavelet_Coif3-HHL_glcm_SumAverage	FALSE	4.59195E-12	7.32415E-09	435	0.005181184	1	1563	[illegible]	[illegible]	[illegible]	[illegible]	[illegible]	[illegible]	[illegible]	[illegible]	[illegible]	[illegible]	[illegible]	[illegible]
		Wavelet_Coif3-HHL_glcm_SumEntropy	FALSE	1.14658E-10	1.82879E-07	564	7.2066E-06	0.011494519	1100	[illegible]	[illegible]	[illegible]	[illegible]	[illegible]	[illegible]	[illegible]	[illegible]	[illegible]	[illegible]	[illegible]	[illegible]
		Wavelet_Coif3-HHL_glcm_SumSquares	TRUE	1.05399E-14	1.68112E-11	212	3.01193E-09	4.80404E-06	705	[illegible]	[illegible]	[illegible]	[illegible]	[illegible]	[illegible]	[illegible]	[illegible]	[illegible]	[illegible]	[illegible]	[illegible]
	GLDM	Wavelet_Coif3-HHL_gldm_DependenceEntropy	FALSE	1.95692E-17	3.12129E-14	3	1.40249E-09	2.23698E-06	671	[illegible]	[illegible]	[illegible]	[illegible]	[illegible]	[illegible]	[illegible]	[illegible]	[illegible]	[illegible]	[illegible]	[illegible]
		Wavelet_Coif3-HHL_gldm_DependenceNonUniformity	TRUE	4.95409E-16	7.90177E-13	108	4.75616E-10	7.58608E-07	624	[illegible]	[illegible]	[illegible]	[illegible]	[illegible]	[illegible]	[illegible]	[illegible]	[illegible]	[illegible]	[illegible]	[illegible]
		Wavelet_Coif3-HHL_gldm_DependenceNonUniformityNormalized	FALSE	2.43835E-17	3.88917E-14	10	1.11693E-09	1.7815E-06	661	[illegible]	[illegible]	[illegible]	[illegible]	[illegible]	[illegible]	[illegible]	[illegible]	[illegible]	[illegible]	[illegible]	[illegible]
		Wavelet_Coif3-HHL_gldm_DependenceVariance	FALSE	6.09341E-15	9.71898E-12	193	1.19299E-08	1.90282E-05	768	[illegible]	[illegible]	[illegible]	[illegible]	[illegible]	[illegible]	[illegible]	[illegible]	[illegible]	[illegible]	[illegible]	[illegible]
		Wavelet_Coif3-HHL_gldm_GrayLevelNonUniformity	TRUE	1.65034E-13	2.6323E-10	310	4.82155E-07	0.000769037	924	[illegible]	[illegible]	[illegible]	[illegible]	[illegible]	[illegible]	[illegible]	[illegible]	[illegible]	[illegible]	[illegible]	[illegible]
		Wavelet_Coif3-HHL_gldm_GrayLevelVariance	TRUE	3.71693E-15	5.9285E-12	176	1.20163E-09	1.9166E-06	642	[illegible]	[illegible]	[illegible]	[illegible]	[illegible]	[illegible]	[illegible]	[illegible]	[illegible]	[illegible]	[illegible]	[illegible]
		Wavelet_Coif3-HHL_gldm_HighGrayLevelEmphasis	TRUE	4.237E-14	6.75801E-11	261	1.75629E-05	0.028012774	1153	[illegible]	[illegible]	[illegible]	[illegible]	[illegible]	[illegible]	[illegible]	[illegible]	[illegible]	[illegible]	[illegible]	[illegible]
		Wavelet_Coif3-HHL_gldm_LargeDependenceEmphasis	FALSE	5.15788E-17	8.22682E-14	34	1.08508E-14	1.7307E-11	213	[illegible]	[illegible]	[illegible]	[illegible]	[illegible]	[illegible]	[illegible]	[illegible]	[illegible]	[illegible]	[illegible]	[illegible]
		Wavelet_Coif3-HHL_gldm_LargeDependenceHighGrayLevelEmphasis	TRUE	2.25599E-08	3.5983E-05	798	0.480634768	1	2135	[illegible]	[illegible]	[illegible]	[illegible]	[illegible]	[illegible]	[illegible]	[illegible]	[illegible]	[illegible]	[illegible]	[illegible]
		Wavelet_Coif3-HHL_gldm_LargeDependenceLowGrayLevelEmphasis	FALSE	1.26251E-16	2.01371E-13	63	4.30618E-08	6.86835E-05	829	[illegible]	[illegible]	[illegible]	[illegible]	[illegible]	[illegible]	[illegible]	[illegible]	[illegible]	[illegible]	[illegible]	[illegible]
		Wavelet_Coif3-HHL_gldm_LowGrayLevelEmphasis	FALSE	1.27192E-08	2.02871E-05	771	0.163833866	1	1947	[illegible]	[illegible]	[illegible]	[illegible]	[illegible]	[illegible]	[illegible]	[illegible]	[illegible]	[illegible]	[illegible]	[illegible]
		Wavelet_Coif3-HHL_gldm_SmallDependenceEmphasis	FALSE	5.34212E-10	8.52068E-07	629	5.4413E-07	0.000867888	957	[illegible]	[illegible]	[illegible]	[illegible]	[illegible]	[illegible]	[illegible]	[illegible]	[illegible]	[illegible]	[illegible]	[illegible]
		Wavelet_Coif3-HHL_gldm_SmallDependenceHighGrayLevelEmphasis	TRUE	2.5509E-14	4.06868E-11	243	2.55956E-08	4.0825E-05	804	[illegible]	[illegible]	[illegible]	[illegible]	[illegible]	[illegible]	[illegible]	[illegible]	[illegible]	[illegible]	[illegible]	[illegible]
		Wavelet_Coif3-HHL_gldm_SmallDependenceLowGrayLevelEmphasis	FALSE	1.39275E-05	0.022214373	1139	0.747771389	1	2240	[illegible]	[illegible]	[illegible]	[illegible]	[illegible]	[illegible]	[illegible]	[illegible]	[illegible]	[illegible]	[illegible]	[illegible]
	GLRLM	Wavelet_Coif3-HHL_glrlm_GrayLevelNonUniformity	TRUE	0.000124522	0.198612192	1278	0.131239923	1	1915	[illegible]	[illegible]	[illegible]	[illegible]	[illegible]	[illegible]	[illegible]	[illegible]	[illegible]	[illegible]	[illegible]	[illegible]
		Wavelet_Coif3-HHL_glrlm_GrayLevelNonUniformityNormalized	FALSE	1.97613E-14	3.15193E-11	234	2.94561E-09	4.69825E-06	704	[illegible]	[illegible]	[illegible]	[illegible]	[illegible]	[illegible]	[illegible]	[illegible]	[illegible]	[illegible]	[illegible]	[illegible]
		Wavelet_Coif3-HHL_glrlm_GrayLevelVariance	TRUE	7.46064E-15	1.18997E-11	200	1.4676E-09	2.34083E-06	673	[illegible]	[illegible]	[illegible]	[illegible]	[illegible]	[illegible]	[illegible]	[illegible]	[illegible]	[illegible]	[illegible]	[illegible]
		Wavelet_Coif3-HHL_glrlm_HighGrayLevelRunEmphasis	TRUE	3.21166E-15	5.1226E-12	171	6.82746E-07	0.00108898	969	[illegible]	[illegible]	[illegible]	[illegible]	[illegible]	[illegible]	[illegible]	[illegible]	[illegible]	[illegible]	[illegible]	[illegible]
		Wavelet_Coif3-HHL_glrlm_LongRunEmphasis	FALSE	3.93276E-12	6.27276E-09	429	1.21654E-06	0.001940379	1000	[illegible]	[illegible]	[illegible]	[illegible]	[illegible]	[illegible]	[illegible]	[illegible]	[illegible]	[illegible]	[illegible]	[illegible]
		Wavelet_Coif3-HHL_glrlm_LongRunHighGrayLevelEmphasis	TRUE	4.19877E-09	6.69704E-06	720	0.083900589	1	1855	[illegible]	[illegible]	[illegible]	[illegible]	[illegible]	[illegible]	[illegible]	[illegible]	[illegible]	[illegible]	[illegible]	[illegible]
		Wavelet_Coif3-HHL_glrlm_LongRunLowGrayLevelEmphasis	FALSE	3.02906E-15	4.83135E-12	169	5.66144E-06	0.009030002	1086	[illegible]	[illegible]	[illegible]	[illegible]	[illegible]	[illegible]	[illegible]	[illegible]	[illegible]	[illegible]	[illegible]	[illegible]
		Wavelet_Coif3-HHL_glrlm_LowGrayLevelRunEmphasis	FALSE	1.78829E-08	2.85233E-05	787	0.143816769	1	1928	[illegible]	[illegible]	[illegible]	[illegible]	[illegible]	[illegible]	[illegible]	[illegible]	[illegible]	[illegible]	[illegible]	[illegible]
		Wavelet_Coif3-HHL_glrlm_RunEntropy	FALSE	3.3348E-17	5.319E-14	20	3.19722E-12	5.09957E-09	421	[illegible]	[illegible]	[illegible]	[illegible]	[illegible]	[illegible]	[illegible]	[illegible]	[illegible]	[illegible]	[illegible]	[illegible]
		Wavelet_Coif3-HHL_glrlm_RunLengthNonUniformity	FALSE	4.13113E-08	6.58915E-05	827	0.001872023	1	1477	[illegible]	[illegible]	[illegible]	[illegible]	[illegible]	[illegible]	[illegible]	[illegible]	[illegible]	[illegible]	[illegible]	[illegible]
		Wavelet_Coif3-HHL_glrlm_RunLengthNonUniformityNormalized	FALSE	0.915601686	1	2299	0.566152692	1	2171	[illegible]	[illegible]	[illegible]	[illegible]	[illegible]	[illegible]	[illegible]	[illegible]	[illegible]	[illegible]	[illegible]	[illegible]
		Wavelet_Coif3-HHL_glrlm_RunPercentage	FALSE	7.25271E-17	1.15681E-13	45	2.94272E-15	4.69364E-12	168	[illegible]	[illegible]	[illegible]	[illegible]	[illegible]	[illegible]	[illegible]	[illegible]	[illegible]	[illegible]	[illegible]	[illegible]
		Wavelet_Coif3-HHL_glrlm_RunVariance	FALSE	2.84883E-13	4.54388E-10	330	3.06039E-07	0.000488133	927	[illegible]	[illegible]	[illegible]	[illegible]	[illegible]	[illegible]	[illegible]	[illegible]	[illegible]	[illegible]	[illegible]	[illegible]
		Wavelet_Coif3-HHL_glrlm_ShortRunEmphasis	FALSE	5.10536E-16	8.14304E-13	109	8.71614E-12	1.39022E-08	460	[illegible]	[illegible]	[illegible]	[illegible]	[illegible]	[illegible]	[illegible]	[illegible]	[illegible]	[illegible]	[illegible]	[illegible]
		Wavelet_Coif3-HHL_glrlm_ShortRunHighGrayLevelEmphasis	TRUE	1.47156E-16	2.34713E-13	68	6.28573E-10	1.00257E-06	636	[illegible]	[illegible]	[illegible]	[illegible]	[illegible]	[illegible]	[illegible]	[illegible]	[illegible]	[illegible]	[illegible]	[illegible]
		Wavelet_Coif3-HHL_glrlm_ShortRunLowGrayLevelEmphasis	FALSE	8.28059E-08	0.000132075	861	0.130308955	1	1914	[illegible]	[illegible]	[illegible]	[illegible]	[illegible]	[illegible]	[illegible]	[illegible]	[illegible]	[illegible]	[illegible]	[illegible]
	GLSZM	Wavelet_Coif3-HHL_glszm_GrayLevelNonUniformity	FALSE	1.07599E-07	0.000171621	874	0.000308323	0.491775546	921	[illegible]	[illegible]	[illegible]	[illegible]	[illegible]	[illegible]	[illegible]	[illegible]	[illegible]	[illegible]	[illegible]	[illegible]
		Wavelet_Coif3-HHL_glszm_GrayLevelNonUniformityNormalized	FALSE	0.074535188	1	1840	0.801172033	1	1854.5	[illegible]	[illegible]	[illegible]	[illegible]	[illegible]	[illegible]	[illegible]	[illegible]	[illegible]	[illegible]	[illegible]	[illegible]
		Wavelet_Coif3-HHL_glszm_GrayLevelVariance	FALSE	3.17549E-13	5.0649E-10	334	6.61957E-09	1.05582E-05	564	[illegible]	[illegible]	[illegible]	[illegible]	[illegible]	[illegible]	[illegible]	[illegible]	[illegible]	[illegible]	[illegible]	[illegible]
		Wavelet_Coif3-HHL_glszm_HighGrayLevelZoneEmphasis	TRUE	4.63552E-13	7.39365E-10	348	4.02054E-07	0.000641277	740	[illegible]	[illegible]	[illegible]	[illegible]	[illegible]	[illegible]	[illegible]	[illegible]	[illegible]	[illegible]	[illegible]	[illegible]
		Wavelet_Coif3-HHL_glszm_LargeAreaEmphasis	TRUE	1.89644E-10	3.02483E-07	585	1.87083E-05	0.029839783	1127	[illegible]	[illegible]	[illegible]	[illegible]	[illegible]	[illegible]	[illegible]	[illegible]	[illegible]	[illegible]	[illegible]	[illegible]
		Wavelet_Coif3-HHL_glszm_LargeAreaHighGrayLevelEmphasis	TRUE	0.033132364	1	1745	0.370623454	1	2083	[illegible]	[illegible]	[illegible]	[illegible]	[illegible]	[illegible]	[illegible]	[illegible]	[illegible]	[illegible]	[illegible]	[illegible]
		Wavelet_Coif3-HHL_glszm_LargeAreaLowGrayLevelEmphasis	TRUE	9.77743E-14	1.5595E-10	291	9.74215E-07	0.001553874	988	[illegible]	[illegible]	[illegible]	[illegible]	[illegible]	[illegible]	[illegible]	[illegible]	[illegible]	[illegible]	[illegible]	[illegible]
		Wavelet_Coif3-HHL_glszm_LowGrayLevelZoneEmphasis	FALSE	2.51905E-09	4.01789E-06	697	0.001565347	1	1198	[illegible]	[illegible]	[illegible]	[illegible]	[illegible]	[illegible]	[illegible]	[illegible]	[illegible]	[illegible]	[illegible]	[illegible]
		Wavelet_Coif3-HHL_glszm_SizeZoneNonUniformity	FALSE	0.000356596	0.568771279	1351	0.00718317	1	1214	[illegible]	[illegible]	[illegible]	[illegible]	[illegible]	[illegible]	[illegible]	[illegible]	[illegible]	[illegible]	[illegible]	[illegible]
		Wavelet_Coif3-HHL_glszm_SizeZoneNonUniformityNormalized	FALSE	3.15022E-16	5.0246E-13	93	2.13293E-06	0.003402021	794	[illegible]	[illegible]	[illegible]	[illegible]	[illegible]	[illegible]	[illegible]	[illegible]	[illegible]	[illegible]	[illegible]	[illegible]
		Wavelet_Coif3-HHL_glszm_SmallAreaEmphasis	FALSE	1.01598E-15	1.62048E-12	132	0.246823175	1	1968	[illegible]	[illegible]	[illegible]	[illegible]	[illegible]	[illegible]	[illegible]	[illegible]	[illegible]	[illegible]	[illegible]	[illegible]
		Wavelet_Coif3-HHL_glszm_SmallAreaHighGrayLevelEmphasis	TRUE	1.79853E-09	2.86865E-06	682	0.00012088	0.192803277	1276	[illegible]	[illegible]	[illegible]	[illegible]	[illegible]	[illegible]	[illegible]	[illegible]	[illegible]	[illegible]	[illegible]	[illegible]
		Wavelet_Coif3-HHL_glszm_SmallAreaLowGrayLevelEmphasis	FALSE	3.57939E-14	5.70912E-11	255	0.019729628	1	1690	[illegible]	[illegible]	[illegible]	[illegible]	[illegible]	[illegible]	[illegible]	[illegible]	[illegible]	[illegible]	[illegible]	[illegible]
		Wavelet_Coif3-HHL_glszm_ZoneEntropy	FALSE	1.04667E-15	1.66943E-12	133	1.25304E-11	1.99859E-08	330	[illegible]	[illegible]	[illegible]	[illegible]	[illegible]	[illegible]	[illegible]	[illegible]	[illegible]	[illegible]	[illegible]	[illegible]
		Wavelet_Coif3-HHL_glszm_ZonePercentage	FALSE	2.40506E-10	3.83606E-07	595	3.42933E-07	0.000546977	664	[illegible]	[illegible]	[illegible]	[illegible]	[illegible]	[illegible]	[illegible]	[illegible]	[illegible]	[illegible]	[illegible]	[illegible]
		Wavelet_Coif3-HHL_glszm_ZoneVariance	TRUE	2.29368E-10	3.65843E-07	593	0.00313034	1	1484	[illegible]	[illegible]	[illegible]	[illegible]	[illegible]	[illegible]	[illegible]	[illegible]	[illegible]	[illegible]	[illegible]	[illegible]
	NGTDM	Wavelet_Coif3-HHL_ngtdm_Busyness	TRUE	7.40866E-14	1.18168E-10	281	0.003004273	1	1516	[illegible]	[illegible]	[illegible]	[illegible]	[illegible]	[illegible]	[illegible]	[illegible]	[illegible]	[illegible]	[illegible]	[illegible]
		Wavelet_Coif3-HHL_ngtdm_Coarseness	FALSE	0.000546228	0.871234376	1382	0.30276801	1	2046	[illegible]	[illegible]	[illegible]	[illegible]	[illegible]	[illegible]	[illegible]	[illegible]	[illegible]	[illegible]	[illegible]	[illegible]
		Wavelet_Coif3-HHL_ngtdm_Complexity	TRUE	1.78053E-17	2.83995E-14	0	1.38335E-12	2.20644E-09	389	[illegible]	[illegible]	[illegible]	[illegible]	[illegible]	[illegible]	[illegible]	[illegible]	[illegible]	[illegible]	[illegible]	[illegible]
		Wavelet_Coif3-HHL_ngtdm_Contrast	FALSE	1.03336E-13	1.64822E-10	293	0.016192988	1	1670	[illegible]	[illegible]	[illegible]	[illegible]	[illegible]	[illegible]	[illegible]	[illegible]	[illegible]	[illegible]	[illegible]	[illegible]
		Wavelet_Coif3-HHL_ngtdm_Strength	TRUE	3.38334E-14	5.39643E-11	253	0.015713646	1	1667	[illegible]	[illegible]	[illegible]	[illegible]	[illegible]	[illegible]	[illegible]	[illegible]	[illegible]	[illegible]	[illegible]	[illegible]
Wavelet Coiflet 3 - HHH	First Order	Wavelet_Coif3-HHH_firstorder_10Percentile	FALSE	0.701197198	1	2223	0.513033444	1	2149	[illegible]	[illegible]	[illegible]	[illegible]	[illegible]	[illegible]	[illegible]	[illegible]	[illegible]	[illegible]	[illegible]	[illegible]
		Wavelet_Coif3-HHH_firstorder_90Percentile	FALSE	0.756090864	1	2243	0.388457896	1	2092	[illegible]	[illegible]	[illegible]	[illegible]	[illegible]	[illegible]	[illegible]	[illegible]	[illegible]	[illegible]	[illegible]	[illegible]
		Wavelet_Coif3-HHH_firstorder_Energy	FALSE	0.313168582	1	2052	0.959197745	1	2314	[illegible]	[illegible]	[illegible]	[illegible]	[illegible]	[illegible]	[illegible]	[illegible]	[illegible]	[illegible]	[illegible]	[illegible]
		Wavelet_Coif3-HHH_firstorder_Entropy	FALSE	4.961E-12	7.9128E-09	438	0.00022994	0.36675387	1193	[illegible]	[illegible]	[illegible]	[illegible]	[illegible]	[illegible]	[illegible]	[illegible]	[illegible]	[illegible]	[illegible]	[illegible]
		Wavelet_Coif3-HHH_firstorder_InterquartileRange	FALSE	0.956285864	1	2313	0.503662062	1	2145	[illegible]	[illegible]	[illegible]	[illegible]	[illegible]	[illegible]	[illegible]	[illegible]	[illegible]	[illegible]	[illegible]	[illegible]
		Wavelet_Coif3-HHH_firstorder_Kurtosis	TRUE	5.42621E-15	8.65481E-12	189	1.05437E-14	1.68172E-11	212	[illegible]	[illegible]	[illegible]	[illegible]	[illegible]	[illegible]	[illegible]	[illegible]	[illegible]	[illegible]	[illegible]	[illegible]
		Wavelet_Coif3-HHH_firstorder_Maximum	FALSE	1.4508E-15	2.31403E-12	144	5.4413E-07	0.000867888	957	[illegible]	[illegible]	[illegible]	[illegible]	[illegible]	[illegible]	[illegible]	[illegible]	[illegible]	[illegible]	[illegible]	[illegible]
		Wavelet_Coif3-HHH_firstorder_MeanAbsoluteDeviation	FALSE	0.606370763	1	2187	0.237864064	1	2005	[illegible]	[illegible]	[illegible]	[illegible]	[illegible]	[illegible]	[illegible]	[illegible]	[illegible]	[illegible]	[illegible]	[illegible]
		Wavelet_Coif3-HHH_firstorder_Mean	FALSE	0.601273871	1	2185	0.246694098	1	2011	[illegible]	[illegible]	[illegible]	[illegible]	[illegible]	[illegible]	[illegible]	[illegible]	[illegible]	[illegible]	[illegible]	[illegible]
		Wavelet_Coif3-HHH_firstorder_Median	FALSE	0.323804597	1	2058	0.161632822	1	1945	[illegible]	[illegible]	[illegible]	[illegible]	[illegible]	[illegible]	[illegible]	[illegible]	[illegible]	[illegible]	[illegible]	[illegible]
		Wavelet_Coif3-HHH_firstorder_Minimum	FALSE	8.46364E-17	1.34995E-13	50	9.74215E-07	0.001553874	988	[illegible]	[illegible]	[illegible]	[illegible]	[illegible]	[illegible]	[illegible]	[illegible]	[illegible]	[illegible]	[illegible]	[illegible]
		Wavelet_Coif3-HHH_firstorder_Range	FALSE	3.3348E-17	5.319E-14	20	2.15474E-07	0.000343681	909	[illegible]	[illegible]	[illegible]	[illegible]	[illegible]	[illegible]	[illegible]	[illegible]	[illegible]	[illegible]	[illegible]	[illegible]
		Wavelet_Coif3-HHH_firstorder_RobustMeanAbsoluteDeviation	FALSE	0.92430479	1	2302	0.494381839	1	2141	[illegible]	[illegible]	[illegible]	[illegible]	[illegible]	[illegible]	[illegible]	[illegible]	[illegible]	[illegible]	[illegible]	[illegible]
		Wavelet_Coif3-HHH_firstorder_RootMeanSquared	FALSE	0.189552107	1	1969	0.094915399	1	1871	[illegible]	[illegible]	[illegible]	[illegible]	[illegible]	[illegible]	[illegible]	[illegible]	[illegible]	[illegible]	[illegible]	[illegible]
		Wavelet_Coif3-HHH_firstorder_Skewness	TRUE	0.282655245	1	2034	0.714789644	1	2228	[illegible]	[illegible]	[illegible]	[illegible]	[illegible]	[illegible]	[illegible]	[illegible]	[illegible]	[illegible]	[illegible]	[illegible]
		Wavelet_Coif3-HHH_firstorder_TotalEnergy	FALSE	0.313168582	1	2052	0.959197745	1	2314	[illegible]	[illegible]	[illegible]	[illegible]	[illegible]	[illegible]	[illegible]	[illegible]	[illegible]	[illegible]	[illegible]	[illegible]
		Wavelet_Coif3-HHH_firstorder_Uniformity	FALSE	1.18163E-11	1.88469E-08	472	0.000521503	0.831796969	1248	[illegible]	[illegible]	[illegible]	[illegible]	[illegible]	[illegible]	[illegible]	[illegible]	[illegible]	[illegible]	[illegible]	[illegible]
		Wavelet_Coif3-HHH_firstorder_Variance	FALSE	0.185879198	1	1966	0.082599622	1	1853	[illegible]	[illegible]	[illegible]	[illegible]	[illegible]	[illegible]	[illegible]	[illegible]	[illegible]	[illegible]	[illegible]	[illegible]
	GLCM	Wavelet_Coif3-HHH_glcm_Autocorrelation	FALSE	4.8318E-05	0.077067226	1216	0.00699922	1	1590	[illegible]	[illegible]	[illegible]	[illegible]	[illegible]	[illegible]	[illegible]	[illegible]	[illegible]	[illegible]	[illegible]	[illegible]
		Wavelet_Coif3-HHH_glcm_ClusterProminence	TRUE	4.961E-12	7.9128E-09	438	0.00068665	1	1399	[illegible]	[illegible]	[illegible]	[illegible]	[illegible]	[illegible]	[illegible]	[illegible]	[illegible]	[illegible]	[illegible]	[illegible]
		Wavelet_Coif3-HHH_glcm_ClusterShade	FALSE	0.423537731	1	2109	0.953375076	1	2312	[illegible]	[illegible]	[illegible]	[illegible]	[illegible]	[illegible]	[illegible]	[illegible]	[illegible]	[illegible]	[illegible]	[illegible]
		Wavelet_Coif3-HHH_glcm_ClusterTendency	FALSE	1.30734E-11	2.08521E-08	476	0.006198744	1	1579	[illegible]	[illegible]	[illegible]	[illegible]	[illegible]	[illegible]	[illegible]	[illegible]	[illegible]	[illegible]	[illegible]	[illegible]
		Wavelet_Coif3-HHH_glcm_Contrast	FALSE	5.9997E-10	9.56952E-07	634	6.06712E-06	0.009677052	1090	[illegible]	[illegible]	[illegible]	[illegible]	[illegible]	[illegible]	[illegible]	[illegible]	[illegible]	[illegible]	[illegible]	[illegible]
		Wavelet_Coif3-HHH_glcm_Correlation	FALSE	0.254223133	1	2016	0.33467603	1	2064	[illegible]	[illegible]	[illegible]	[illegible]	[illegible]	[illegible]	[illegible]	[illegible]	[illegible]	[illegible]	[illegible]	[illegible]
		Wavelet_Coif3-HHH_glcm_DifferenceAverage	FALSE	1.24498E-08	1.98574E-05	770	2.03581E-05	0.032471212	1162	[illegible]	[illegible]	[illegible]	[illegible]	[illegible]	[illegible]	[illegible]	[illegible]	[illegible]	[illegible]	[illegible]	[illegible]
		Wavelet_Coif3-HHH_glcm_DifferenceEntropy	FALSE	0.345774158	1	2070	0.364793447	1	2080	[illegible]	[illegible]	[illegible]	[illegible]	[illegible]	[illegible]	[illegible]	[illegible]	[illegible]	[illegible]	[illegible]	[illegible]
		Wavelet_Coif3-HHH_glcm_DifferenceVariance	FALSE	0.000882784	1	1418	0.00731257	1	1594	[illegible]	[illegible]	[illegible]	[illegible]	[illegible]	[illegible]	[illegible]	[illegible]	[illegible]	[illegible]	[illegible]	[illegible]
		Wavelet_Coif3-HHH_glcm_Id	FALSE	2.83141E-07	0.00045161	923	0.000172216	0.27468418	1300	[illegible]	[illegible]	[illegible]	[illegible]	[illegible]	[illegible]	[illegible]	[illegible]	[illegible]	[illegible]	[illegible]	[illegible]
		Wavelet_Coif3-HHH_glcm_Idm	FALSE	7.7925E-08	0.00012429	858	6.59321E-05	0.105161684	1236	[illegible]	[illegible]	[illegible]	[illegible]	[illegible]	[illegible]	[illegible]	[illegible]	[illegible]	[illegible]	[illegible]	[illegible]
		Wavelet_Coif3-HHH_glcm_Idmn	FALSE	0.001317419	1	1449	0.048043648	1	1787	[illegible]	[illegible]	[illegible]	[illegible]	[illegible]	[illegible]	[illegible]	[illegible]	[illegible]	[illegible]	[illegible]	[illegible]
		Wavelet_Coif3-HHH_glcm_Idn	FALSE	0.000351654	0.560887765	1350	0.033435965	1	1746	[illegible]	[illegible]	[illegible]	[illegible]	[illegible]	[illegible]	[illegible]	[illegible]	[illegible]	[illegible]	[illegible]	[illegible]
		Wavelet_Coif3-HHH_glcm_Imc1	TRUE	1.78053E-17	2.83995E-14	0	6.89451E-16	1.09967E-12	119	[illegible]	[illegible]	[illegible]	[illegible]	[illegible]	[illegible]	[illegible]	[illegible]	[illegible]	[illegible]	[illegible]	[illegible]
		Wavelet_Coif3-HHH_glcm_Imc2	TRUE	1.78053E-17	2.83995E-14	0	1.66373E-16	2.65365E-13	72	[illegible]	[illegible]	[illegible]	[illegible]	[illegible]	[illegible]	[illegible]	[illegible]	[illegible]	[illegible]	[illegible]	[illegible]
		Wavelet_Coif3-HHH_glcm_InverseVariance	FALSE	0.464860741	1	2128	0.220879006	1	1993	[illegible]	[illegible]	[illegible]	[illegible]	[illegible]	[illegible]	[illegible]	[illegible]	[illegible]	[illegible]	[illegible]	[illegible]
		Wavelet_Coif3-HHH_glcm_JointAverage	FALSE	3.81459E-05	0.060842635	1201	0.006130228	1	1578	[illegible]	[illegible]	[illegible]	[illegible]	[illegible]	[illegible]	[illegible]	[illegible]	[illegible]	[illegible]	[illegible]	[illegible]
		Wavelet_Coif3-HHH_glcm_JointEnergy	TRUE	6.10425E-08	9.73627E-05	846	1.99176E-06	0.00317686	1027	[illegible]	[illegible]	[illegible]	[illegible]	[illegible]	[illegible]	[illegible]	[illegible]	[illegible]	[illegible]	[illegible]	[illegible]
		Wavelet_Coif3-HHH_glcm_JointEntropy	FALSE	0.007721569	1	1599	0.529649296	1	2156	[illegible]	[illegible]	[illegible]	[illegible]	[illegible]	[illegible]	[illegible]	[illegible]	[illegible]	[illegible]	[illegible]	[illegible]
		Wavelet_Coif3-HHH_glcm_MCC	FALSE	5.26117E-16	8.39157E-13	110	5.58796E-15	8.9128E-12	190	[illegible]	[illegible]	[illegible]	[illegible]	[illegible]	[illegible]	[illegible]	[illegible]	[illegible]	[illegible]	[illegible]	[illegible]
		Wavelet_Coif3-HHH_glcm_MaximumProbability	TRUE	1.95692E-17	3.12129E-14	3	1.24463E-12	1.98519E-09	385	[illegible]	[illegible]	[illegible]	[illegible]	[illegible]	[illegible]	[illegible]	[illegible]	[illegible]	[illegible]	[illegible]	[illegible]
		Wavelet_Coif3-HHH_glcm_SumAverage	FALSE	3.81459E-05	0.060842635	1201	0.006130228	1	1578	[illegible]	[illegible]	[illegible]	[illegible]	[illegible]	[illegible]	[illegible]	[illegible]	[illegible]	[illegible]	[illegible]	[illegible]
		Wavelet_Coif3-HHH_glcm_SumEntropy	FALSE	0.492073625	1	2140	0.218134891	1	1991	[illegible]	[illegible]	[illegible]	[illegible]	[illegible]	[illegible]	[illegible]	[illegible]	[illegible]	[illegible]	[illegible]	[illegible]
		Wavelet_Coif3-HHH_glcm_SumSquares	TRUE	2.70406E-11	4.31298E-08	505	0.000190682	0.304138361	1307	[illegible]	[illegible]	[illegible]	[illegible]	[illegible]	[illegible]	[illegible]	[illegible]	[illegible]	[illegible]	[illegible]	[illegible]
	GLDM	Wavelet_Coif3-HHH_gldm_DependenceEntropy	FALSE	3.13271E-17	4.99668E-14	18	8.29571E-05	0.132316501	1251	[illegible]	[illegible]	[illegible]	[illegible]	[illegible]	[illegible]	[illegible]	[illegible]	[illegible]	[illegible]	[illegible]	[illegible]
		Wavelet_Coif3-HHH_gldm_DependenceNonUniformity	TRUE	8.24607E-16	1.31525E-12	125	1.15102E-06	0.001835881	997	[illegible]	[illegible]	[illegible]	[illegible]	[illegible]	[illegible]	[illegible]	[illegible]	[illegible]	[illegible]	[illegible]	[illegible]
		Wavelet_Coif3-HHH_gldm_DependenceNonUniformityNormalized	FALSE	3.03625E-17	4.84282E-14	17	8.5491E-06	0.013635818	1110	[illegible]	[illegible]	[illegible]	[illegible]	[illegible]	[illegible]	[illegible]	[illegible]	[illegible]	[illegible]	[illegible]	[illegible]
		Wavelet_Coif3-HHH_gldm_DependenceVariance	FALSE	2.19321E-15	3.49817E-12	158	0.000871343	1	1417	[illegible]	[illegible]	[illegible]	[illegible]	[illegible]	[illegible]	[illegible]	[illegible]	[illegible]	[illegible]	[illegible]	[illegible]
		Wavelet_Coif3-HHH_gldm_GrayLevelNonUniformity	TRUE	2.40506E-10	3.83606E-07	595	0.002208922	1	1353	[illegible]	[illegible]	[illegible]	[illegible]	[illegible]	[illegible]	[illegible]	[illegible]	[illegible]	[illegible]	[illegible]	[illegible]
		Wavelet_Coif3-HHH_gldm_GrayLevelVariance	TRUE	8.49461E-12	1.35489E-08	459	0.000288719	0.460506047	1208	[illegible]	[illegible]	[illegible]	[illegible]	[illegible]	[illegible]	[illegible]	[illegible]	[illegible]	[illegible]	[illegible]	[illegible]
		Wavelet_Coif3-HHH_gldm_HighGrayLevelEmphasis	FALSE	1.28825E-07	0.000205476	883	0.001257064	1	1411	[illegible]	[illegible]	[illegible]	[illegible]	[illegible]	[illegible]	[illegible]	[illegible]	[illegible]	[illegible]	[illegible]	[illegible]
		Wavelet_Coif3-HHH_gldm_LargeDependenceEmphasis	FALSE	0.004840061	1	1557	0.029137327	1	1731	[illegible]	[illegible]	[illegible]	[illegible]	[illegible]	[illegible]	[illegible]	[illegible]	[illegible]	[illegible]	[illegible]	[illegible]
		Wavelet_Coif3-HHH_gldm_LargeDependenceHighGrayLevelEmphasis	FALSE	0.001236043	1	1444	0.128462402	1	1912	[illegible]	[illegible]	[illegible]	[illegible]	[illegible]	[illegible]	[illegible]	[illegible]	[illegible]	[illegible]	[illegible]	[illegible]
		Wavelet_Coif3-HHH_gldm_LargeDependenceLowGrayLevelEmphasis	FALSE	2.20222E-09	3.51255E-06	691	0.003935934	1	1539	[illegible]	[illegible]	[illegible]	[illegible]	[illegible]	[illegible]	[illegible]	[illegible]	[illegible]	[illegible]	[illegible]	[illegible]
		Wavelet_Coif3-HHH_gldm_LowGrayLevelEmphasis	FALSE	0.008788951	1	1611	0.057371204	1	1768	[illegible]	[illegible]	[illegible]	[illegible]	[illegible]	[illegible]	[illegible]	[illegible]	[illegible]	[illegible]	[illegible]	[illegible]
		Wavelet_Coif3-HHH_gldm_SmallDependenceEmphasis	FALSE	9.00446E-11	1.43621E-07	554	0.000254226	0.405489883	1327	[illegible]	[illegible]	[illegible]	[illegible]	[illegible]	[illegible]	[illegible]	[illegible]	[illegible]	[illegible]	[illegible]	[illegible]
		Wavelet_Coif3-HHH_gldm_SmallDependenceHighGrayLevelEmphasis	FALSE	5.92845E-14	9.45587E-11	273	6.19848E-05	0.098865735	1232	[illegible]	[illegible]	[illegible]	[illegible]	[illegible]	[illegible]	[illegible]	[illegible]	[illegible]	[illegible]	[illegible]	[illegible]
		Wavelet_Coif3-HHH_gldm_SmallDependenceLowGrayLevelEmphasis	FALSE	0.204778931	1	1981	0.445015666	1	2119	[illegible]	[illegible]	[illegible]	[illegible]	[illegible]	[illegible]	[illegible]	[illegible]	[illegible]	[illegible]	[illegible]	[illegible]
	GLRLM	Wavelet_Coif3-HHH_glrlm_GrayLevelNonUniformity	TRUE	4.10712E-09	6.55085E-06	719	0.013091258	1	1649	[illegible]	[illegible]	[illegible]	[illegible]	[illegible]	[illegible]	[illegible]	[illegible]	[illegible]	[illegible]	[illegible]	[illegible]
		Wavelet_Coif3-HHH_glrlm_GrayLevelNonUniformityNormalized	FALSE	6.58007E-12	1.04952E-08	449	0.000714662	1	1402	[illegible]	[illegible]	[illegible]	[illegible]	[illegible]	[illegible]	[illegible]	[illegible]	[illegible]	[illegible]	[illegible]	[illegible]
		Wavelet_Coif3-HHH_glrlm_GrayLevelVariance	TRUE	6.09321E-12	9.71866E-09	446	0.000305656	0.487520558	1340	[illegible]	[illegible]	[illegible]	[illegible]	[illegible]	[illegible]	[illegible]	[illegible]	[illegible]	[illegible]	[illegible]	[illegible]
		Wavelet_Coif3-HHH_glrlm_HighGrayLevelRunEmphasis	FALSE	1.34077E-11	2.13852E-08	477	0.001300844	1	1448	[illegible]	[illegible]	[illegible]	[illegible]	[illegible]	[illegible]	[illegible]	[illegible]	[illegible]	[illegible]	[illegible]	[illegible]
		Wavelet_Coif3-HHH_glrlm_LongRunEmphasis	FALSE	3.11433E-12	4.96736E-09	420	4.94766E-07	0.000789151	952	[illegible]	[illegible]	[illegible]	[illegible]	[illegible]	[illegible]	[illegible]	[illegible]	[illegible]	[illegible]	[illegible]	[illegible]
		Wavelet_Coif3-HHH_glrlm_LongRunHighGrayLevelEmphasis	FALSE	9.66941E-15	1.54227E-11	209	1.51632E-06	0.002418527	1012	[illegible]	[illegible]	[illegible]	[illegible]	[illegible]	[illegible]	[illegible]	[illegible]	[illegible]	[illegible]	[illegible]	[illegible]
		Wavelet_Coif3-HHH_glrlm_LongRunLowGrayLevelEmphasis	FALSE	0.237861323	1	2005	0.979592198	1	2321	[illegible]	[illegible]	[illegible]	[illegible]	[illegible]	[illegible]	[illegible]	[illegible]	[illegible]	[illegible]	[illegible]	[illegible]
		Wavelet_Coif3-HHH_glrlm_LowGrayLevelRunEmphasis	FALSE	0.040001379	1	1766	0.104693338	1	1884	[illegible]	[illegible]	[illegible]	[illegible]	[illegible]	[illegible]	[illegible]	[illegible]	[illegible]	[illegible]	[illegible]	[illegible]
		Wavelet_Coif3-HHH_glrlm_RunEntropy	FALSE	1.78053E-17	2.83995E-14	0	6.63027E-14	1.05753E-10	277	[illegible]	[illegible]	[illegible]	[illegible]	[illegible]	[illegible]	[illegible]	[illegible]	[illegible]	[illegible]	[illegible]	[illegible]
		Wavelet_Coif3-HHH_glrlm_RunLengthNonUniformity	TRUE	5.45189E-14	8.69577E-11	270	3.93043E-09	6.26904E-06	717	[illegible]	[illegible]	[illegible]	[illegible]	[illegible]	[illegible]	[illegible]	[illegible]	[illegible]	[illegible]	[illegible]	[illegible]
		Wavelet_Coif3-HHH_glrlm_RunLengthNonUniformityNormalized	FALSE	1.95692E-17	3.12129E-14	3	3.3534E-13	5.34867E-10	336	[illegible]	[illegible]	[illegible]	[illegible]	[illegible]	[illegible]	[illegible]	[illegible]	[illegible]	[illegible]	[illegible]	[illegible]
		Wavelet_Coif3-HHH_glrlm_RunPercentage	FALSE	7.78597E-07	0.001241863	976	0.000600479	0.957764371	1389	[illegible]	[illegible]	[illegible]	[illegible]	[illegible]	[illegible]	[illegible]	[illegible]	[illegible]	[illegible]	[illegible]	[illegible]
		Wavelet_Coif3-HHH_glrlm_RunVariance	FALSE	0.063918715	1	1821	0.233533745	1	2002	[illegible]	[illegible]	[illegible]	[illegible]	[illegible]	[illegible]	[illegible]	[illegible]	[illegible]	[illegible]	[illegible]	[illegible]
		Wavelet_Coif3-HHH_glrlm_ShortRunEmphasis	FALSE	1.70108E-07	0.000271322	897	0.094194617	1	1870	[illegible]	[illegible]	[illegible]	[illegible]	[illegible]	[illegible]	[illegible]	[illegible]	[illegible]	[illegible]	[illegible]	[illegible]
		Wavelet_Coif3-HHH_glrlm_ShortRunHighGrayLevelEmphasis	FALSE	0.001781354	1	1473	0.05836678	1	1810	[illegible]	[illegible]	[illegible]	[illegible]	[illegible]	[illegible]	[illegible]	[illegible]	[illegible]	[illegible]	[illegible]	[illegible]
		Wavelet_Coif3-HHH_glrlm_ShortRunLowGrayLevelEmphasis	FALSE	0.000162418	0.259057196	1296	0.090656288	1	1865	[illegible]	[illegible]	[illegible]	[illegible]	[illegible]	[illegible]	[illegible]	[illegible]	[illegible]	[illegible]	[illegible]	[illegible]
	GLSZM	Wavelet_Coif3-HHH_glszm_GrayLevelNonUniformity	TRUE	3.08739E-06	0.004924391	941	0.001594002	1	224.5	[illegible]	[illegible]	[illegible]	[illegible]	[illegible]	[illegible]	[illegible]	[illegible]	[illegible]	[illegible]	[illegible]	[illegible]
		Wavelet_Coif3-HHH_glszm_GrayLevelNonUniformityNormalized	FALSE	0.330314545	1	1889	0.222725673	1	601	[illegible]	[illegible]	[illegible]	[illegible]	[illegible]	[illegible]	[illegible]	[illegible]	[illegible]	[illegible]	[illegible]	[illegible]
		Wavelet_Coif3-HHH_glszm_GrayLevelVariance	FALSE	2.99607E-12	4.77873E-09	347	2.84178E-05	0.04532647	256.5	[illegible]	[illegible]	[illegible]	[illegible]	[illegible]	[illegible]	[illegible]	[illegible]	[illegible]	[illegible]	[illegible]	[illegible]
		Wavelet_Coif3-HHH_glszm_HighGrayLevelZoneEmphasis	FALSE	8.65062E-10	1.37977E-06	585	0.00017074	0.272331056	321.5	[illegible]	[illegible]	[illegible]	[illegible]	[illegible]	[illegible]	[illegible]	[illegible]	[illegible]	[illegible]	[illegible]	[illegible]
		Wavelet_Coif3-HHH_glszm_LargeAreaEmphasis	TRUE	1.7584E-09	2.80464E-06	681	0.00183872	1	1339	[illegible]	[illegible]	[illegible]	[illegible]	[illegible]	[illegible]	[illegible]	[illegible]	[illegible]	[illegible]	[illegible]	[illegible]
		Wavelet_Coif3-HHH_glszm_LargeAreaHighGrayLevelEmphasis	TRUE	0.047630759	1	1786	0.041941619	1	1732	[illegible]	[illegible]	[illegible]	[illegible]	[illegible]	[illegible]	[illegible]	[illegible]	[illegible]	[illegible]	[illegible]	[illegible]
		Wavelet_Coif3-HHH_glszm_LargeAreaLowGrayLevelEmphasis	TRUE	1.37503E-11	2.19317E-08	478	0.00029146	0.464878032	1304	[illegible]	[illegible]	[illegible]	[illegible]	[illegible]	[illegible]	[illegible]	[illegible]	[illegible]	[illegible]	[illegible]	[illegible]
		Wavelet_Coif3-HHH_glszm_LowGrayLevelZoneEmphasis	FALSE	0.15571995	1	1815	0.038841218	1	523.5	[illegible]	[illegible]	[illegible]	[illegible]	[illegible]	[illegible]	[illegible]	[illegible]	[illegible]	[illegible]	[illegible]	[illegible]
		Wavelet_Coif3-HHH_glszm_SizeZoneNonUniformity	TRUE	5.53386E-08	8.8265E-05	767.5	0.007592927	1	281	[illegible]	[illegible]	[illegible]	[illegible]	[illegible]	[illegible]	[illegible]	[illegible]	[illegible]	[illegible]	[illegible]	[illegible]
		Wavelet_Coif3-HHH_glszm_SizeZoneNonUniformityNormalized	FALSE	0.006867355	1	1480	0.584455255	1	629	[illegible]	[illegible]	[illegible]	[illegible]	[illegible]	[illegible]	[illegible]	[illegible]	[illegible]	[illegible]	[illegible]	[illegible]
		Wavelet_Coif3-HHH_glszm_SmallAreaEmphasis	FALSE	0.000633617	1	1393	0.002267059	1	1355	[illegible]	[illegible]	[illegible]	[illegible]	[illegible]	[illegible]	[illegible]	[illegible]	[illegible]	[illegible]	[illegible]	[illegible]
		Wavelet_Coif3-HHH_glszm_SmallAreaHighGrayLevelEmphasis	FALSE	3.02528E-08	4.82532E-05	812	0.001074912	1	1399	[illegible]	[illegible]	[illegible]	[illegible]	[illegible]	[illegible]	[illegible]	[illegible]	[illegible]	[illegible]	[illegible]	[illegible]
		Wavelet_Coif3-HHH_glszm_SmallAreaLowGrayLevelEmphasis	FALSE	0.722986518	1	2231	0.035648655	1	1714	[illegible]	[illegible]	[illegible]	[illegible]	[illegible]	[illegible]	[illegible]	[illegible]	[illegible]	[illegible]	[illegible]	[illegible]
		Wavelet_Coif3-HHH_glszm_ZoneEntropy	FALSE	0.002000218	1	1379	0.000291549	0.465020601	322	[illegible]	[illegible]	[illegible]	[illegible]	[illegible]	[illegible]	[illegible]	[illegible]	[illegible]	[illegible]	[illegible]	[illegible]
		Wavelet_Coif3-HHH_glszm_ZonePercentage	FALSE	2.01231E-10	3.20964E-07	486	7.14419E-05	0.113949891	296	[illegible]	[illegible]	[illegible]	[illegible]	[illegible]	[illegible]	[illegible]	[illegible]	[illegible]	[illegible]	[illegible]	[illegible]
		Wavelet_Coif3-HHH_glszm_ZoneVariance	TRUE	2.08584E-10	3.32692E-07	589	0.959627807	1	2126	[illegible]	[illegible]	[illegible]	[illegible]	[illegible]	[illegible]	[illegible]	[illegible]	[illegible]	[illegible]	[illegible]	[illegible]
	NGTDM	Wavelet_Coif3-HHH_ngtdm_Busyness	TRUE	1.08875E-06	0.001736558	994	0.002278152	1	1493	[illegible]	[illegible]	[illegible]	[illegible]	[illegible]	[illegible]	[illegible]	[illegible]	[illegible]	[illegible]	[illegible]	[illegible]
		Wavelet_Coif3-HHH_ngtdm_Coarseness	TRUE	7.7925E-08	0.00012429	858	0.750541798	1	2241	[illegible]	[illegible]	[illegible]	[illegible]	[illegible]	[illegible]	[illegible]	[illegible]	[illegible]	[illegible]	[illegible]	[illegible]
		Wavelet_Coif3-HHH_ngtdm_Complexity	FALSE	0.000174731	0.278696683	1301	7.12012E-05	0.113565868	1241	[illegible]	[illegible]	[illegible]	[illegible]	[illegible]	[illegible]	[illegible]	[illegible]	[illegible]	[illegible]	[illegible]	[illegible]
		Wavelet_Coif3-HHH_ngtdm_Contrast	FALSE	0.003187565	1	1521	0.069340852	1	1831	[illegible]	[illegible]	[illegible]	[illegible]	[illegible]	[illegible]	[illegible]	[illegible]	[illegible]	[illegible]	[illegible]	[illegible]
		Wavelet_Coif3-HHH_ngtdm_Strength	FALSE	6.00901E-05	0.09584371	1230	0.033435965	1	1746	[illegible]	[illegible]	[illegible]	[illegible]	[illegible]	[illegible]	[illegible]	[illegible]	[illegible]	[illegible]	[illegible]	[illegible]
	First Order	Wavelet_Coif3-LLL_firstorder_10Percentile	FALSE	4.98544E-05	0.079517703	1218	2.074E-08	3.30802E-05	794	[illegible]	[illegible]	[illegible]	[illegible]	[illegible]	[illegible]	[illegible]	[illegible]	[illegible]	[illegible]	[illegible]	[illegible]
		Wavelet_Coif3-LLL_firstorder_90Percentile	TRUE	7.03188E-17	1.12159E-13	44	1.6062E-13	2.5619E-10	309	[illegible]	[illegible]	[illegible]	[illegible]	[illegible]	[illegible]	[illegible]	[illegible]	[illegible]	[illegible]	[illegible]	[illegible]
		Wavelet_Coif3-LLL_firstorder_Energy	FALSE	1.45808E-10	2.32563E-07	574	6.43295E-10	1.02606E-06	637	[illegible]	[illegible]	[illegible]	[illegible]	[illegible]	[illegible]	[illegible]	[illegible]	[illegible]	[illegible]	[illegible]	[illegible]
		Wavelet_Coif3-LLL_firstorder_Entropy	FALSE	1.78053E-17	2.83995E-14	0	3.55689E-16	5.67324E-13	97	[illegible]	[illegible]	[illegible]	[illegible]	[illegible]	[illegible]	[illegible]	[illegible]	[illegible]	[illegible]	[illegible]	[illegible]
		Wavelet_Coif3-LLL_firstorder_InterquartileRange	FALSE	2.43835E-17	3.88917E-14	10	1.42033E-12	2.26543E-09	390	[illegible]	[illegible]	[illegible]	[illegible]	[illegible]	[illegible]	[illegible]	[illegible]	[illegible]	[illegible]	[illegible]	[illegible]
		Wavelet_Coif3-LLL_firstorder_Kurtosis	FALSE	3.66459E-16	5.84502E-13	98	2.06858E-15	3.29938E-12	156	[illegible]	[illegible]	[illegible]	[illegible]	[illegible]	[illegible]	[illegible]	[illegible]	[illegible]	[illegible]	[illegible]	[illegible]
		Wavelet_Coif3-LLL_firstorder_Maximum	FALSE	6.56418E-11	1.04699E-07	541	0.440671617	1	2117	[illegible]	[illegible]	[illegible]	[illegible]	[illegible]	[illegible]	[illegible]	[illegible]	[illegible]	[illegible]	[illegible]	[illegible]
		Wavelet_Coif3-LLL_firstorder_MeanAbsoluteDeviation	FALSE	5.42168E-16	8.64758E-13	111	2.77334E-13	4.42348E-10	329	[illegible]	[illegible]	[illegible]	[illegible]	[illegible]	[illegible]	[illegible]	[illegible]	[illegible]	[illegible]	[illegible]	[illegible]
		Wavelet_Coif3-LLL_firstorder_Mean	TRUE	4.39235E-13	7.0058E-10	346	3.00893E-06	0.004799245	1050	[illegible]	[illegible]	[illegible]	[illegible]	[illegible]	[illegible]	[illegible]	[illegible]	[illegible]	[illegible]	[illegible]	[illegible]
		Wavelet_Coif3-LLL_firstorder_Median	TRUE	1.3707E-09	2.18627E-06	670	0.469339873	1	2130	[illegible]	[illegible]	[illegible]	[illegible]	[illegible]	[illegible]	[illegible]	[illegible]	[illegible]	[illegible]	[illegible]	[illegible]
		Wavelet_Coif3-LLL_firstorder_Minimum	FALSE	0.661009477	1	2208	0.010527077	1	1628	[illegible]	[illegible]	[illegible]	[illegible]	[illegible]	[illegible]	[illegible]	[illegible]	[illegible]	[illegible]	[illegible]	[illegible]
		Wavelet_Coif3-LLL_firstorder_Range	FALSE	5.86019E-06	0.009346999	1088	0.246694098	1	2011	[illegible]	[illegible]	[illegible]	[illegible]	[illegible]	[illegible]	[illegible]	[illegible]	[illegible]	[illegible]	[illegible]	[illegible]
		Wavelet_Coif3-LLL_firstorder_RobustMeanAbsoluteDeviation	FALSE	2.36302E-17	3.76901E-14	9	6.57331E-13	1.04844E-09	361	[illegible]	[illegible]	[illegible]	[illegible]	[illegible]	[illegible]	[illegible]	[illegible]	[illegible]	[illegible]	[illegible]	[illegible]
		Wavelet_Coif3-LLL_firstorder_RootMeanSquared	TRUE	1.15209E-11	1.83759E-08	471	1.90704E-11	3.04172E-08	491	[illegible]	[illegible]	[illegible]	[illegible]	[illegible]	[illegible]	[illegible]	[illegible]	[illegible]	[illegible]	[illegible]	[illegible]
		Wavelet_Coif3-LLL_firstorder_Skewness	FALSE	0.005064965	1	1561	3.30668E-07	0.000527416	931	[illegible]	[illegible]	[illegible]	[illegible]	[illegible]	[illegible]	[illegible]	[illegible]	[illegible]	[illegible]	[illegible]	[illegible]
		Wavelet_Coif3-LLL_firstorder_TotalEnergy	FALSE	1.45808E-10	2.32563E-07	574	6.43295E-10	1.02606E-06	637	[illegible]	[illegible]	[illegible]	[illegible]	[illegible]	[illegible]	[illegible]	[illegible]	[illegible]	[illegible]	[illegible]	[illegible]
		Wavelet_Coif3-LLL_firstorder_Uniformity	FALSE	1.78053E-17	2.83995E-14	0	1.93806E-16	3.0912E-13	77	[illegible]	[illegible]	[illegible]	[illegible]	[illegible]	[illegible]	[illegible]	[illegible]	[illegible]	[illegible]	[illegible]	[illegible]
		Wavelet_Coif3-LLL_firstorder_Variance	FALSE	3.96371E-08	6.32211E-05	825	6.58354E-10	1.05007E-06	638	[illegible]	[illegible]	[illegible]	[illegible]	[illegible]	[illegible]	[illegible]	[illegible]	[illegible]	[illegible]	[illegible]	[illegible]
		Wavelet_Coif3-LLL_glcm_Autocorrelation	FALSE	0.467096035	1	2129	0.011445874	1	1636	[illegible]	[illegible]	[illegible]	[illegible]	[illegible]	[illegible]	[illegible]	[illegible]	[illegible]	[illegible]	[illegible]	[illegible]
		Wavelet_Coif3-LLL_glcm_ClusterProminence	TRUE	0.000967324	1	1425	0.008064072	1	1603	[illegible]	[illegible]	[illegible]	[illegible]	[illegible]	[illegible]	[illegible]	[illegible]	[illegible]	[illegible]	[illegible]	[illegible]
		Wavelet_Coif3-LLL_glcm_ClusterShade	TRUE	0.778416214	1	2251	0.00159224	1	1464	[illegible]	[illegible]	[illegible]	[illegible]	[illegible]	[illegible]	[illegible]	[illegible]	[illegible]	[illegible]	[illegible]	[illegible]
		Wavelet_Coif3-LLL_glcm_ClusterTendency	FALSE	3.36526E-09	5.36758E-06	710	4.54677E-11	7.2521E-08	526	[illegible]	[illegible]	[illegible]	[illegible]	[illegible]	[illegible]	[illegible]	[illegible]	[illegible]	[illegible]	[illegible]	[illegible]
		Wavelet_Coif3-LLL_glcm_Contrast	FALSE	7.18487E-08	0.000114599	854	1.3402E-09	2.13763E-06	669	[illegible]	[illegible]	[illegible]	[illegible]	[illegible]	[illegible]	[illegible]	[illegible]	[illegible]	[illegible]	[illegible]	[illegible]
		Wavelet_Coif3-LLL_glcm_Correlation	FALSE	4.00839E-07	0.000639339	941	2.40335E-08	3.83335E-05	801	[illegible]	[illegible]	[illegible]	[illegible]	[illegible]	[illegible]	[illegible]	[illegible]	[illegible]	[illegible]	[illegible]	[illegible]
		Wavelet_Coif3-LLL_glcm_DifferenceAverage	FALSE	3.03625E-17	4.84282E-14	17	6.64858E-15	1.06045E-11	196	[illegible]	[illegible]	[illegible]	[illegible]	[illegible]	[illegible]	[illegible]	[illegible]	[illegible]	[illegible]	[illegible]	[illegible]
		Wavelet_Coif3-LLL_glcm_DifferenceEntropy	FALSE	4.69872E-17	7.49446E-14	31	7.04437E-15	1.12358E-11	198	[illegible]	[illegible]	[illegible]	[illegible]	[illegible]	[illegible]	[illegible]	[illegible]	[illegible]	[illegible]	[illegible]	[illegible]
		Wavelet_Coif3-LLL_glcm_DifferenceVariance	FALSE	0.015093371	1	1663	0.094915399	1	1871	[illegible]	[illegible]	[illegible]	[illegible]	[illegible]	[illegible]	[illegible]	[illegible]	[illegible]	[illegible]	[illegible]	[illegible]
		Wavelet_Coif3-LLL_glcm_Id	FALSE	1.78053E-17	2.83995E-14	0	3.45096E-16	5.50428E-13	96	[illegible]	[illegible]	[illegible]	[illegible]	[illegible]	[illegible]	[illegible]	[illegible]	[illegible]	[illegible]	[illegible]	[illegible]

Wavelet Coiflet 3 - LLL	GLCM	Wavelet_Coif3-LLL_glcm_Idm	FALSE	1.78053E-17	2.83995E-14	0	3.45096E-16	5.50428E-13	96	0.040714475	1	1768	0.000546269	0.871299676	1382	0.055976865	1	1805	0.000524463	0.83651769	1379
		Wavelet_Coif3-LLL_glcm_Idmn	FALSE	8.86972E-15	1.41472E-11	206	5.96761E-09	9.51834E-06	736	0.775615099	1	2250	0.653085267	1	2205	0.271352173	1	2027	0.72298807	1	2231
		Wavelet_Coif3-LLL_glcm_Idn	FALSE	1.89629E-17	3.02458E-14	2	3.3082E-15	5.27658E-12	172	0.284295652	1	2035	0.329212663	1	2061	0.08389878	1	1855	0.081955298	1	1852
		Wavelet_Coif3-LLL_glcm_Imc1	FALSE	3.44061E-17	5.48777E-14	21	1.05437E-14	1.68172E-11	212	0.232100073	1	2001	0.122159286	1	1905	0.372576783	1	2084	0.029681492	1	1733
		Wavelet_Coif3-LLL_glcm_Imc2	FALSE	1.14433E-15	1.8252E-12	136	3.78787E-14	6.04166E-11	257	0.347646875	1	2071	0.546534445	1	2163	0.157295396	1	1941	0.105477456	1	1885
		Wavelet_Coif3-LLL_glcm_InverseVariance	FALSE	4.55482E-17	7.26494E-14	30	3.94171E-15	6.28703E-12	178	0.345774158	1	2070	0.068227531	1	1829	0.150958941	1	1935	0.031366724	1	1739
		Wavelet_Coif3-LLL_glcm_JointAverage	FALSE	0.099331372	1	1877	0.018062272	1	1681	0.979592079	1	2321	0.208723612	1	1984	0.869380882	1	2283	0.279396521	1	2032
		Wavelet_Coif3-LLL_glcm_JointEnergy	FALSE	1.78053E-17	2.83995E-14	0	8.20986E-17	1.30947E-13	49	0.00417014	1	1544	0.000108873	0.173652817	1269	0.060336482	1	1814	0.00937098	1	1617
		Wavelet_Coif3-LLL_glcm_JointEntropy	FALSE	1.78053E-17	2.83995E-14	0	3.8943E-16	6.2114E-13	100	0.085216271	1	1857	0.000250626	0.39974843	1326	0.174016209	1	1956	0.000223502	0.356486446	1318
		Wavelet_Coif3-LLL_glcm_MCC	FALSE	1.28545E-06	0.002050295	1003	5.11943E-09	8.16548E-06	729	0.653083373	1	2205	0.368673722	1	2082	0.758870849	1	2244	0.107059666	1	1887
		Wavelet_Coif3-LLL_glcm_MaximumProbability	FALSE	1.78053E-17	2.83995E-14	0	1.11706E-16	1.78171E-13	59	0.0023059	1	1494	9.50847E-05	0.151660129	1260	0.071610604	1	1835	0.010092764	1	1624
		Wavelet_Coif3-LLL_glcm_SumAverage	FALSE	0.099331372	1	1877	0.018062272	1	1681	0.979592079	1	2321	0.208723612	1	1984	0.869380882	1	2283	0.279396521	1	2032
		Wavelet_Coif3-LLL_glcm_SumEntropy	FALSE	1.78053E-17	2.83995E-14	0	5.26322E-16	8.39484E-13	110	0.144818898	1	1929	0.000107253	0.171068248	1268	0.362860109	1	2079	6.59321E-05	0.105161684	1236
		Wavelet_Coif3-LLL_glcm_SumSquares	FALSE	5.00724E-09	7.98654E-06	728	6.25297E-11	9.97349E-08	539	0.601273871	1	2185	0.125730786	1	1909	0.489773714	1	2139	0.067676407	1	1828
	GLDM	Wavelet_Coif3-LLL_gldm_DependenceEntropy	FALSE	3.2322E-17	5.15536E-14	19	1.69677E-13	2.70635E-10	311	0.063918715	1	1821	0.436351979	1	2115	0.054583274	1	1802	0.204781577	1	1981
		Wavelet_Coif3-LLL_gldm_DependenceNonUniformity	TRUE	1.78053E-17	2.83995E-14	0	1.5179E-16	2.42105E-13	69	0.208720952	1	1984	0.000805194	1	1411	0.018421052	1	1683	1.53001E-10	2.44036E-07	576
		Wavelet_Coif3-LLL_gldm_DependenceNonUniformityNormalized	TRUE	1.78053E-17	2.83995E-14	0	3.55689E-16	5.67324E-13	97	0.037254019	1	1758	0.000288876	0.460757417	1336	0.013498901	1	1652	2.39596E-05	0.038215557	1172
		Wavelet_Coif3-LLL_gldm_DependenceVariance	TRUE	1.78053E-17	2.83995E-14	0	9.0057E-17	1.43641E-13	52	0.002223174	1	1491	5.76036E-06	0.009187768	1087	0.08389878	1	1855	0.005928752	1	1575
		Wavelet_Coif3-LLL_gldm_GrayLevelNonUniformity	TRUE	1.78053E-17	2.83995E-14	0	6.41108E-17	1.02257E-13	41	0.000155427	0.247906512	1293	9.04313E-07	0.001442379	984	0.000503431	0.802972335	1376	7.08935E-07	0.001130752	971
		Wavelet_Coif3-LLL_gldm_GrayLevelVariance	FALSE	4.39518E-08	7.01032E-05	830	5.59754E-10	8.92807E-07	631	0.687701293	1	2218	0.138874347	1	1923	0.494379317	1	2141	0.071612261	1	1835
		Wavelet_Coif3-LLL_gldm_HighGrayLevelEmphasis	FALSE	0.501330917	1	2144	0.007233074	1	1593	0.260362689	1	2020	0.000356625	0.568816574	1351	0.271352173	1	2027	0.000773897	1	1408
		Wavelet_Coif3-LLL_gldm_LargeDependenceEmphasis	TRUE	1.78053E-17	2.83995E-14	0	8.20986E-17	1.30947E-13	49	0.003585451	1	1531	2.20891E-05	0.035232118	1167	0.065506838	1	1824	0.00407511	1	1542
		Wavelet_Coif3-LLL_gldm_LargeDependenceHighGrayLevelEmphasis	TRUE	7.71507E-17	1.23055E-13	47	1.76853E-16	2.82081E-13	74	1.95578E-06	0.003119472	1026	1.45193E-07	0.000231583	889	0.451574659	1	2122	3.11783E-06	0.004972945	1052
		Wavelet_Coif3-LLL_gldm_LargeDependenceLowGrayLevelEmphasis	FALSE	1.63305E-15	2.60472E-12	148	1.89322E-13	3.01969E-10	315	0.09636827	1	1873	3.20196E-05	0.051071271	1190	0.48290883	1	2136	0.003113163	1	1519
		Wavelet_Coif3-LLL_gldm_LowGrayLevelEmphasis	FALSE	0.290921619	1	2039	0.390471262	1	2093	0.976677441	1	2320	0.080678879	1	1850	0.935922859	1	2306	0.100841938	1	1879
		Wavelet_Coif3-LLL_gldm_SmallDependenceEmphasis	FALSE	2.7641E-17	4.40874E-14	14	1.14476E-15	1.82589E-12	136	0.306207145	1	2048	0.017708966	1	1679	0.26818039	1	2025	0.004840309	1	1557
		Wavelet_Coif3-LLL_gldm_SmallDependenceHighGrayLevelEmphasis	FALSE	1.02621E-08	1.63681E-05	761	4.75616E-10	7.58608E-07	624	0.066043388	1	1825	0.013918839	1	1655	0.048042342	1	1787	0.004122485	1	1543
		Wavelet_Coif3-LLL_gldm_SmallDependenceLowGrayLevelEmphasis	FALSE	0.331024503	1	2062	0.001268103	1	1446	0.706622361	1	2225	0.004417384	1	1549	0.653083373	1	2205	0.00242073	1	1498
	GLRLM	Wavelet_Coif3-LLL_glrlm_GrayLevelNonUniformity	TRUE	1.67804E-08	2.67648E-05	784	2.16246E-12	3.44912E-09	406	0.311418531	1	2051	1.78544E-05	0.028477724	1154	0.761653954	1	2245	2.17321E-05	0.034662654	1166
		Wavelet_Coif3-LLL_glrlm_GrayLevelNonUniformityNormalized	FALSE	1.78053E-17	2.83995E-14	0	2.47242E-16	3.94351E-13	85	0.460408054	1	2126	0.010638221	1	1629	0.476096548	1	2133	0.004267625	1	1546
		Wavelet_Coif3-LLL_glrlm_GrayLevelVariance	FALSE	0.374539291	1	2085	4.20917E-06	0.00671363	1069	0.432054154	1	2113	0.301056715	1	2045	0.299349052	1	2044	0.234970931	1	2003
		Wavelet_Coif3-LLL_glrlm_HighGrayLevelRunEmphasis	FALSE	0.536850824	1	2159	0.015713646	1	1667	0.222257606	1	1994	0.000387674	0.618339447	1357	0.197057862	1	1975	0.00100587	1	1428
		Wavelet_Coif3-LLL_glrlm_LongRunEmphasis	FALSE	1.78053E-17	2.83995E-14	0	9.0057E-17	1.43641E-13	52	0.003670255	1	1533	6.29501E-05	0.10040539	1233	0.085216271	1	1857	0.011686416	1	1638
		Wavelet_Coif3-LLL_glrlm_LongRunHighGrayLevelEmphasis	TRUE	9.51047E-14	1.51692E-10	290	3.63705E-13	5.8011E-10	339	1.39275E-05	0.022214373	1139	0.006479757	1	1583	0.011445393	1	1636	0.000894489	1	1419
		Wavelet_Coif3-LLL_glrlm_LongRunLowGrayLevelEmphasis	FALSE	1.22334E-09	1.95123E-06	665	9.08674E-05	0.14493345	1257	0.541680668	1	2161	0.02532308	1	1716	0.706622361	1	2225	0.06604497	1	1825
		Wavelet_Coif3-LLL_glrlm_LowGrayLevelRunEmphasis	FALSE	0.059344104	1	1812	0.87801893	1	2286	0.947554589	1	2310	0.093478231	1	1869	0.99125297	1	2325	0.123934996	1	1907
		Wavelet_Coif3-LLL_glrlm_RunEntropy	FALSE	9.86171E-16	1.57294E-12	131	0.00016007	0.255311481	1295	0.58861707	1	2180	0.442840596	1	2118	0.803751758	1	2260	0.745004234	1	2239
		Wavelet_Coif3-LLL_glrlm_RunLengthNonUniformity	TRUE	1.78053E-17	2.83995E-14	0	1.15191E-16	1.8373E-13	60	0.857888803	1	2279	1.90671E-05	0.030412094	1158	0.632139605	1	2197	3.96443E-08	6.32326E-05	825
		Wavelet_Coif3-LLL_glrlm_RunLengthNonUniformityNormalized	FALSE	1.78053E-17	2.83995E-14	0	2.62727E-16	4.19049E-13	87	0.015871408	1	1668	6.90474E-05	0.110130672	1239	0.059344104	1	1812	0.000351682	0.560932518	1350
		Wavelet_Coif3-LLL_glrlm_RunPercentage	FALSE	1.78053E-17	2.83995E-14	0	1.93806E-16	3.0912E-13	77	0.009777148	1	1621	4.39783E-05	0.070145403	1210	0.05644793	1	1806	0.000859979	1	1416
		Wavelet_Coif3-LLL_glrlm_RunVariance	FALSE	1.78053E-17	2.83995E-14	0	8.46711E-17	1.3505E-13	50	0.002065826	1	1485	3.15113E-05	0.050260563	1189	0.083246246	1	1854	0.017190365	1	1676
		Wavelet_Coif3-LLL_glrlm_ShortRunEmphasis	FALSE	1.78053E-17	2.83995E-14	0	1.87984E-16	2.99835E-13	76	0.012693504	1	1646	5.82619E-05	0.092927787	1228	0.077556634	1	1845	0.000980063	1	1426
		Wavelet_Coif3-LLL_glrlm_ShortRunHighGrayLevelEmphasis	FALSE	0.011685927	1	1638	0.000445095	0.709926607	1367	0.085216271	1	1857	0.000187935	0.299756115	1306	0.062362524	1	1818	0.000457493	0.729701243	1369
		Wavelet_Coif3-LLL_glrlm_ShortRunLowGrayLevelEmphasis	FALSE	0.406802645	1	2101	0.329212663	1	2061	0.956285864	1	2313	0.127546784	1	1911	0.947554589	1	2310	0.144821246	1	1929
	GLSZM	Wavelet_Coif3-LLL_glszm_GrayLevelNonUniformity	TRUE	3.2322E-17	5.15536E-14	19	1.17928E-15	1.88095E-12	137	0.976677441	1	2320	0.081955298	1	1852	0.621776014	1	2193	1.84513E-05	0.029429773	1156
		Wavelet_Coif3-LLL_glszm_GrayLevelNonUniformityNormalized	FALSE	0.233531013	1	2002	0.478364009	1	2134	0.970849136	1	2318	0.586102738	1	2179	0.997084271	1	2327	0.529649296	1	2156
		Wavelet_Coif3-LLL_glszm_GrayLevelVariance	FALSE	2.30326E-09	3.6737E-06	693	0.160540707	1	1944	0.230675369	1	2000	0.281024077	1	2033	0.243722834	1	2009	0.141823853	1	1926
		Wavelet_Coif3-LLL_glszm_HighGrayLevelZoneEmphasis	FALSE	0.938829607	1	2307	0.003150251	1	1520	0.127544559	1	1911	0.000204995	0.326967418	1312	0.083246246	1	1854	0.001101231	1	1435
		Wavelet_Coif3-LLL_glszm_LargeAreaEmphasis	TRUE	1.78053E-17	2.83995E-14	0	5.16003E-17	8.23025E-14	34	0.026048477	1	1719	8.49698E-12	1.35527E-08	459	0.578581096	1	2176	2.27188E-11	3.62364E-08	498
		Wavelet_Coif3-LLL_glszm_LargeAreaHighGrayLevelEmphasis	TRUE	1.78053E-17	2.83995E-14	0	9.57805E-17	1.5277E-13	54	1.88148E-09	3.00096E-06	684	2.24517E-05	0.03581048	1168	1.8862E-06	0.003008496	1024	2.62727E-16	4.19049E-13	87
		Wavelet_Coif3-LLL_glszm_LargeAreaLowGrayLevelEmphasis	TRUE	1.66306E-16	2.65258E-13	72	5.12199E-15	8.16957E-12	187	0.000763663	1	1407	3.51864E-09	5.61223E-06	712	0.332845627	1	2063	1.76991E-07	0.000282301	899
		Wavelet_Coif3-LLL_glszm_LowGrayLevelZoneEmphasis	FALSE	0.001204813	1	1442	3.81498E-05	0.060848986	1201	0.809411021	1	2262	0.12663625	1	1910	0.826447574	1	2268	0.124830373	1	1908
		Wavelet_Coif3-LLL_glszm_SizeZoneNonUniformity	FALSE	2.70406E-11	4.31298E-08	505	9.22756E-11	1.4718E-07	555	0.745002796	1	2239	0.005240061	1	1564	0.883784593	1	2288	0.078174478	1	1846
		Wavelet_Coif3-LLL_glszm_SizeZoneNonUniformityNormalized	FALSE	2.55523E-13	4.07559E-10	326	0.698491973	1	2222	0.566150423	1	2171	0.62177805	1	2193	0.566150423	1	2171	0.578583315	1	2176
		Wavelet_Coif3-LLL_glszm_SmallAreaEmphasis	FALSE	1.56221E-13	2.49173E-10	308	0.59366718	1	2182	0.634741941	1	2198	0.639962105	1	2200	0.606370763	1	2187	0.551407288	1	2165
		Wavelet_Coif3-LLL_glszm_SmallAreaHighGrayLevelEmphasis	FALSE	0.043675548	1	1776	0.007154355	1	1592	0.164940355	1	1948	0.019729628	1	1690	0.068780721	1	1830	0.080678879	1	1850
		Wavelet_Coif3-LLL_glszm_SmallAreaLowGrayLevelEmphasis	FALSE	0.000199136	0.317621788	1310	0.003040177	1	1517	0.471584453	1	2131	0.770022959	1	2248	0.541680668	1	2161	0.795283375	1	2257
		Wavelet_Coif3-LLL_glszm_ZoneEntropy	FALSE	1.18934E-07	0.000189699	879	0.000553724	0.883189912	1383	0.432054154	1	2113	0.281024077	1	2033	0.480632205	1	2135	0.417218098	1	2106
		Wavelet_Coif3-LLL_glszm_ZonePercentage	FALSE	5.32056E-17	8.48629E-14	35	3.03018E-15	4.83314E-12	169	0.492073625	1	2140	0.037589523	1	1759	0.384447397	1	2090	0.006062394	1	1577
		Wavelet_Coif3-LLL_glszm_ZoneVariance	TRUE	1.78053E-17	2.83995E-14	0	5.16003E-17	8.23025E-14	34	0.026294529	1	1720	8.49698E-12	1.35527E-08	459	0.576084726	1	2175	1.95541E-11	3.11887E-08	492
	NGTDM	Wavelet_Coif3-LLL_ngtdm_Busyness	FALSE	3.08905E-08	4.92703E-05	813	9.22531E-05	0.147143725	1258	0.345774158	1	2070	0.010977937	1	1632	0.345774158	1	2070	0.012055639	1	1641
		Wavelet_Coif3-LLL_ngtdm_Coarseness	FALSE	6.25137E-11	9.97094E-08	539	6.96346E-06	0.011106721	1098	0.434198658	1	2114	0.020314528	1	1693	0.177514152	1	1959	0.018972669	1	1686
		Wavelet_Coif3-LLL_ngtdm_Complexity	TRUE	6.59256E-05	0.105151347	1236	4.19964E-09	6.69843E-06	720	0.038951615	1	1763	0.000327914	0.523022408	1345	0.033132364	1	1745	3.2883E-06	0.005244846	1055
		Wavelet_Coif3-LLL_ngtdm_Contrast	FALSE	4.95409E-16	7.90177E-13	108	3.50061E-08	5.58348E-05	819	0.720250106	1	2230	0.563682033	1	2170	0.629541818	1	2196	0.55630139	1	2167
		Wavelet_Coif3-LLL_ngtdm_Strength	FALSE	4.39235E-13	7.0058E-10	346	0.000146559	0.23376137	1289	0.000356596	0.568771279	1351	0.118667577	1	1901	0.000906211	1	1420	0.065508412	1	1824
Gabor - kernel size 8 - frequency = 0.5 - orientation 0	First Order	Gabor_S8Fp5A0_firstorder_10Percentile	FALSE	0.080677108	1	1850	0.010977937	1	1632	0.915601686	1	2299	0.685015585	1	2217	0.909805166	1	2297	0.728471475	1	2233
		Gabor_S8Fp5A0_firstorder_90Percentile	FALSE	0.12846017	1	1912	0.016519646	1	1672	0.921402696	1	2301	0.792465505	1	2256	0.994168581	1	2326	0.866505949	1	2282
		Gabor_S8Fp5A0_firstorder_Energy	FALSE	0.685013843	1	2217	0.160540707	1	1944	0.886670109	1	2289	0.434201343	1	2114	0.880900619	1	2287	0.282658057	1	2034
		Gabor_S8Fp5A0_firstorder_Entropy	FALSE	0.171713015	1	1954	0.029956836	1	1734	0.860759099	1	2280	0.601275996	1	2185	0.970849136	1	2318	0.731218417	1	2234
		Gabor_S8Fp5A0_firstorder_InterquartileRange	FALSE	0.011685927	1	1638	0.001759463	1	1472	0.840707747	1	2273	0.761655305	1	2245	0.895335523	1	2292	0.895336131	1	2292
		Gabor_S8Fp5A0_firstorder_Kurtosis	TRUE	3.05624E-16	4.8747E-13	92	8.87289E-15	1.41523E-11	206	5.19012E-06	0.008278246	1081	1.61721E-05	0.025794491	1148	3.49997E-08	5.58246E-05	819	3.15113E-05	0.050260563	1189
		Gabor_S8Fp5A0_firstorder_Maximum	FALSE	5.00011E-17	7.97518E-14	33	1.12021E-07	0.000178673	876	0.647820901	1	2203	0.935923232	1	2306	0.997084271	1	2327	0.770022959	1	2248
		Gabor_S8Fp5A0_firstorder_MeanAbsoluteDeviation	FALSE	0.272947663	1	2028	0.055045973	1	1803	0.970849136	1	2318	0.770022959	1	2248	0.909805166	1	2297	0.889557769	1	2290
		Gabor_S8Fp5A0_firstorder_Mean	FALSE	7.67914E-15	1.22482E-11	201	2.95586E-06	0.004714593	1049	0.653083373	1	2205	0.19079081	1	1970	6.59256E-05	0.105151347	1236	0.008151701	1	1604
		Gabor_S8Fp5A0_firstorder_Median	FALSE	2.2848E-07	0.000364426	912	0.826448572	1	2268	0.534444049	1	2158	0.156227965	1	1940	0.898226825	1	2293	0.132176039	1	1916
		Gabor_S8Fp5A0_firstorder_Minimum	FALSE	3.66459E-16	5.84502E-13	98	2.70369E-06	0.004312378	1044	0.028336262	1	1728	0.268183186	1	2025	0.626948606	1	2195	0.921403154	1	2301
		Gabor_S8Fp5A0_firstorder_Range	FALSE	2.28998E-17	3.65252E-14	8	5.98185E-08	9.54105E-05	845	0.171713015	1	1954	0.817919593	1	2265	0.817918548	1	2265	0.849290197	1	2276
		Gabor_S8Fp5A0_firstorder_RobustMeanAbsoluteDeviation	FALSE	0.019922217	1	1691	0.003263947	1	1523	0.857888803	1	2279	0.745004234	1	2239	0.886670109	1	2289	0.857889624	1	2279
		Gabor_S8Fp5A0_firstorder_RootMeanSquared	FALSE	0.834997126	1	2271	0.34205087	1	2068	0.967935548	1	2317	0.626950619	1	2195	0.959197507	1	2314	0.676977277	1	2214
		Gabor_S8Fp5A0_firstorder_Skewness	FALSE	0.00023668	0.377504633	1322	8.29571E-05	0.132316501	1251	0.959197507	1	2314	0.052326356	1	1797	0.122157104	1	1905	0.034670015	1	1750
		Gabor_S8Fp5A0_firstorder_TotalEnergy	FALSE	0.685013843	1	2217	0.160540707	1	1944	0.886670109	1	2289	0.432056844	1	2113	0.880900619	1	2287	0.284298465	1	2035
		Gabor_S8Fp5A0_firstorder_Uniformity	FALSE	0.00105931	1	1432	0.000398573	0.635723176	1359	0.447194162	1	2120	0.384450175	1	2090	0.800925959	1	2259	0.58359123	1	2178
		Gabor_S8Fp5A0_firstorder_Variance	FALSE	0.979592079	1	2321	0.578583315	1	2176	0.99125297	1	2325	0.852154733	1	2277	0.997084271	1	2327	0.875138167	1	2285
	GLCM	Gabor_S8Fp5A0_glcm_Autocorrelation	FALSE	3.89277E-16	6.20896E-13	100	6.89973E-08	0.000110051	852	9.365E-05	0.149371706	1259	0.109468172	1	1890	4.68266E-05	0.074688457	1214	0.008151701	1	1604
		Gabor_S8Fp5A0_glcm_ClusterProminence	TRUE	0.204778931	1	1981	0.323807424	1	2058	0.000204978	0.326939385	1312	0.013362151	1	1651	1.89639E-12	3.02473E-09	401	1.24327E-11	1.98301E-08	474
		Gabor_S8Fp5A0_glcm_ClusterShade	TRUE	4.90805E-05	0.078283376	1217	0.003502625	1	1529	5.74745E-13	9.16718E-10	356	5.86351E-10	9.35229E-07	633	1.66777E-07	0.00026601	896	1.45843E-10	2.3262E-07	574
		Gabor_S8Fp5A0_glcm_ClusterTendency	FALSE	0.666314815	1	2210	0.304485762	1	2047	0.994168581	1	2326	0.792465505	1	2256	0.997084271	1	2327	0.826448572	1	2268
		Gabor_S8Fp5A0_glcm_Contrast	FALSE	0.898226825	1	2293	0.517752969	1	2151	0.997084271	1	2327	0.843567126	1	2274	0.99125297	1	2325	0.880901309	1	2287
		Gabor_S8Fp5A0_glcm_Correlation	FALSE	0.866505176	1	2282	0.515390402	1	2150	0.756090864	1	2243	0.772818192	1	2249	0.742238913	1	2238	0.798103917	1	2258
		Gabor_S8Fp5A0_glcm_DifferenceAverage	FALSE	0.254223133	1	2016	0.04035776	1	1767	0.99125297	1	2325	0.714789644	1	2228	0.927207892	1	2303	0.820760245	1	2266
		Gabor_S8Fp5A0_glcm_DifferenceEntropy	FALSE	0.292594211	1	2040	0.063398004	1	1820	0.904013444	1	2295	0.661011334	1	2208	0.979592079	1	2321	0.784028284	1	2253
		Gabor_S8Fp5A0_glcm_DifferenceVariance	FALSE	0.578581096	1	2176	0.857889624	1	2279	0.988337474	1	2324	0.944645833	1	2309	0.806580124	1	2261	0.875138167	1	2285
		Gabor_S8Fp5A0_glcm_Id	FALSE	0.003381182	1	1526	0.00060862	0.970749095	1390	0.685013843	1	2217	0.515390402	1	2150	0.927207892	1	2303	0.642577912	1	2201
		Gabor_S8Fp5A0_glcm_Idm	FALSE	0.002763421	1	1509	0.000561273	0.895231146	1384	0.698490298	1	2222	0.508336427	1	2147	0.938829607	1	2307	0.650451865	1	2204
		Gabor_S8Fp5A0_glcm_Idmn	FALSE	2.15053E-17	3.4301E-14	6	3.19722E-12	5.09957E-09	421	0.578581096	1	2176	0.183462551	1	1964	0.1239328	1	1907	0.10160305	1	1880
		Gabor_S8Fp5A0_glcm_Idn	FALSE	1.89629E-17	3.02458E-14	2	1.40005E-13	2.23308E-10	304	0.469337277	1	2130	0.084557266	1	1856	0.282655245	1	2034	0.103138991	1	1882
		Gabor_S8Fp5A0_glcm_Imc1	FALSE	5.7573E-16	9.18289E-13	113	5.64329E-12	9.00105E-09	443	0.276157862	1	2030	0.162730537	1	1946	0.471584453	1	2131	0.34205087	1	2068
		Gabor_S8Fp5A0_glcm_Imc2	FALSE	6.80081E-09	1.08473E-05	742	0.000503469	0.803033238	1376	0.71206247	1	2227	0.608928526	1	2188	0.826447574	1	2268	0.674305941	1	2213
		Gabor_S8Fp5A0_glcm_InverseVariance	FALSE	0.556299084	1	2167	0.576086955	1	2175	0.742238913	1	2238	0.933017355	1	2305	0.747769965	1	2240	0.895336131	1	2292
		Gabor_S8Fp5A0_glcm_JointAverage	FALSE	4.80724E-16	7.66754E-13	107	2.24087E-07	0.000357419	911	0.049718682	1	1791	0.619198733	1	2192	0.515387948	1	2150	0.837852303	1	2272
		Gabor_S8Fp5A0_glcm_JointEnergy	FALSE	1.61703E-05	0.025791552	1148	1.93824E-05	0.03091489	1159	0.222257606	1	1994	0.130308955	1	1914	0.78122021	1	2252	0.425660281	1	2110
		Gabor_S8Fp5A0_glcm_JointEntropy	FALSE	0.048042342	1	1787	0.003757081	1	1535	0.817918548	1	2265	0.553851692	1	2166	0.988337474	1	2324	0.706623996	1	2225
		Gabor_S8Fp5A0_glcm_MCC	FALSE	2.70406E-11	4.31298E-08	505	6.89973E-08	0.000110051	852	0.973763113	1	2319	0.695788915	1	2221	0.98250699	1	2322	0.249687846	1	2013
		Gabor_S8Fp5A0_glcm_MaximumProbability	FALSE	1.48863E-06	0.002374371	1011	5.46847E-06	0.008722215	1084	0.233531013	1	2002	0.130308955	1	1914	0.720250106	1	2230	0.419319338	1	2107
		Gabor_S8Fp5A0_glcm_SumAverage	FALSE	4.80724E-16	7.66754E-13	107	2.24087E-07	0.000357419	911	0.049718682	1	1791	0.619198733	1	2192	0.515387948	1	2150	0.837852303	1	2272
		Gabor_S8Fp5A0_glcm_SumEntropy	FALSE	0.016851436	1	1674	0.001284377	1	1447	0.803751758	1	2260	0.563682033	1	2170	0.956285864	1	2313	0.725728015	1	2232
		Gabor_S8Fp5A0_glcm_SumSquares	FALSE	0.803751758	1	2260	0.423540439	1	2109	1	1	2328	0.840708665	1	2273	0.99125297	1	2325	0.886670766	1	2289
	GLDM	Gabor_S8Fp5A0_gldm_DependenceEntropy	FALSE	0.449381383	1	2121	0.510682113	1	2148	0.947554589	1	2310	0.789650343	1	2255	0.967935548	1	2317	0.857889624	1	2279
		Gabor_S8Fp5A0_gldm_DependenceNonUniformity	FALSE	2.3957E-05	0.03821137	1172	0.001826228	1	1475	0.728469951	1	2233	0.025563198	1	1717	0.720250106	1	2230	0.524874142	1	2154
		Gabor_S8Fp5A0_gldm_DependenceNonUniformityNormalized	FALSE	1.01275E-05	0.016153356	1120	0.000346803	0.55315076	1349	0.731216908	1	2234	0.573595689	1	2174	0.994168581	1	2326	0.720251672	1	2230
		Gabor_S8Fp5A0_gldm_DependenceVariance	FALSE	1.16882E-09	1.86427E-06	663	3.71233E-07	0.000592117	937	0.099331372	1	1877	0.079418652	1	1848	0.901119478	1	2294	0.402684098	1	2099
		Gabor_S8Fp5A0_gldm_GrayLevelNonUniformity	FALSE	0.001966674	1	1481	0.003381369	1	1526	0.394514211	1	2095	0.090656288	1	1865	0.425657577	1	2110	0.146846314	1	1931
		Gabor_S8Fp5A0_gldm_GrayLevelVariance	FALSE	0.985422135	1	2323	0.571109542	1	2173	0.99125297	1	2325	0.872259057	1	2284	0.99125297	1	2325	0.886670766	1	2289
		Gabor_S8Fp5A0_gldm_HighGrayLevelEmphasis	FALSE	7.67914E-15	1.22482E-11	201	2.8619E-05	0.045647285	1183	8.94928E-05	0.142741013	1256	0.138874347	1	1923	4.98544E-05	0.079517703	1218	0.008979537	1	1613
		Gabor_S8Fp5A0_gldm_LargeDependenceEmphasis	FALSE	3.28787E-06	0.005244155	1055	1.64416E-05	0.026224301	1149	0.185879198	1	1966	0.099333347	1	1877	0.869380882	1	2283	0.347649692	1	2071
		Gabor_S8Fp5A0_gldm_LargeDependenceHighGrayLevelEmphasis	FALSE	3.77832E-17	6.02642E-14	24	4.7759E-11	7.61756E-08	528	0.004316799	1	1547	0.482911388	1	2136	0.001572299	1	1463	0.001421678	1	1455
		Gabor_S8Fp5A0_gldm_LargeDependenceLowGrayLevelEmphasis	FALSE	8.94928E-05	0.142741013	1256	0.728471475	1	2233	0.596196407	1	2183	0.561216583	1	2169	0.532042731	1	2157	0.637350765	1	2199
		Gabor_S8Fp5A0_gldm_LowGrayLevelEmphasis	FALSE	5.12013E-15	8.1666E-12	187	0.000187935	0.299756115	1306	0.73947835	1	2237	0.527258961	1	2155	0.243722834	1	2009	0.494381839	1	2141
		Gabor_S8Fp5A0_gldm_SmallDependenceEmphasis	FALSE	0.812244415	1	2263	0.655722998	1	2206	0.927207892	1	2303	0.843567126	1	2274	0.895335523	1	2292	0.855021221	1	2278
		Gabor_S8Fp5A0_gldm_SmallDependenceHighGrayLevelEmphasis	TRUE	1.48303E-11	2.36543E-08	481	0.003341808	1	1525	2.66863E-08	4.25647E-05	806	3.15113E-05	0.050260563	1189	2.64004E-05	0.04210868	1178	0.451577303	1	2122
		Gabor_S8Fp5A0_gldm_SmallDependenceLowGrayLevelEmphasis	FALSE	9.29124E-16	1.48195E-12	129	1.48867E-05	0.023744272	1143	0.453773977	1	2123	0.18832449	1	1968	0.10469131	1	1884	0.121278919	1	1904
	GLRLM	Gabor_S8Fp5A0_glrlm_GrayLevelNonUniformity	FALSE	0.400630121	1	2098	0.075133362	1	1841	0.532042731	1	2157	0.046817764	1	1784	0.501330917	1	2144	0.997084288	1	2327
		Gabor_S8Fp5A0_glrlm_GrayLevelNonUniformityNormalized	FALSE	0.028867544	1	1730	0.003845563	1	1537	0.817918548	1	2265	0.541683029	1	2161	0.94173717	1	2308	0.685015585	1	2217
		Gabor_S8Fp5A0_glrlm_GrayLevelVariance	FALSE	0.709340563	1	2226	0.901120052	1	2294	0.959197507	1	2314	0.962109916	1	2315	0.94173717	1	2308	0.94173751	1	2308
		Gabor_S8Fp5A0_glrlm_HighGrayLevelRunEmphasis	FALSE	6.64619E-15	1.06007E-11	196	1.97026E-05	0.031425596	1160	9.22444E-05	0.147129809	1258	0.140835384	1	1925	5.06398E-05	0.080770464	1219	0.009173395	1	1615
		Gabor_S8Fp5A0_glrlm_LongRunEmphasis	FALSE	6.69489E-05	0.106783469	1237	0.000177313	0.282813705	1302	0.153049112	1	1937	0.064446078	1	1822	0.846426763	1	2275	0.30276801	1	2046
		Gabor_S8Fp5A0_glrlm_LongRunHighGrayLevelEmphasis	FALSE	8.46364E-17	1.34995E-13	50	1.16908E-09	1.86469E-06	663	0.000445061	0.709871805	1367	0.65836503	1	2207	0.00072418	1	1403	0.000553724	0.883189912	1383
		Gabor_S8Fp5A0_glrlm_LongRunLowGrayLevelEmphasis	FALSE	6.89852E-08	0.000110031	852	0.297653489	1	2043	0.764440146	1	2246	0.778417474	1	2251	0.674304148	1	2213	0.750541798	1	2241
		Gabor_S8Fp5A0_glrlm_LowGrayLevelRunEmphasis	FALSE	5.9194E-15	9.44144E-12	192	0.000230012	0.366869758	1320	0.745002796	1	2239	0.503662062	1	2145	0.229256891	1	1999	0.494381839	1	2141
		Gabor_S8Fp5A0_glrlm_RunEntropy	FALSE	0.008979136	1	1613	0.318460336	1	2055	0.695787227	1	2221	0.880901309	1	2287	0.671636899	1	2212	0.924305231	1	2302
		Gabor_S8Fp5A0_glrlm_RunLengthNonUniformity	FALSE	0.002633928	1	1505	0.003381369	1	1526	0.676975497	1	2214	0.006130228	1	1578	0.717517273	1	2229	0.513033444	1	2149
		Gabor_S8Fp5A0_glrlm_RunLengthNonUniformityNormalized	FALSE	0.008151329	1	1604	0.000930278	1	1422	0.645196229	1	2202	0.357109779	1	2076	0.889557128	1	2290	0.503662062	1	2145
		Gabor_S8Fp5A0_glrlm_RunPercentage	FALSE	0.000155427	0.247906512	1293	7.92504E-05	0.126404388	1248	0.368670925	1	2082	0.19833231	1	1976	0.76722939	1	2247	0.396549303	1	2096
		Gabor_S8Fp5A0_glrlm_RunVariance	FALSE	5.01257E-06	0.007995052	1079	4.98594E-05	0.079525768	1218	0.088583268	1	1862	0.037589523	1	1759	0.979592079	1	2321	0.284298465	1	2035
		Gabor_S8Fp5A0_glrlm_ShortRunEmphasis	FALSE	0.007232734	1	1593	0.001458056	1	1457	0.601273871	1	2185	0.266606881	1	2024	0.878018223	1	2286	0.423540439	1	2109
		Gabor_S8Fp5A0_glrlm_ShortRunHighGrayLevelEmphasis	TRUE	2.34307E-14	3.7372E-11	240	0.000108873	0.173652817	1269	2.3957E-05	0.03821137	1172	0.092058573	1	1867	7.92428E-05	0.126392221	1248	0.017361732	1	1677
		Gabor_S8Fp5A0_glrlm_ShortRunLowGrayLevelEmphasis	FALSE	2.6161E-15	4.17268E-12	164	0.000148733	0.237229908	1290	0.661009477	1	2208	0.390471262	1	2093	0.156225546	1	1940	0.343910701	1	2069
	GLSZM	Gabor_S8Fp5A0_glszm_GrayLevelNonUniformity	FALSE	0.002763421	1	1509	0.022160909	1	1702	0.761653954	1	2245	0.008064072	1	1603	0.706622361	1	2225	0.01180836	1	1639
		Gabor_S8Fp5A0_glszm_GrayLevelNonUniformityNormalized	FALSE	0.001386049	1	1453	0.386450864	1	2091	0.634741941	1	2198	0.901120052	1	2294	0.629541818	1	2196	0.892446236	1	2291
		Gabor_S8Fp5A0_glszm_GrayLevelVariance	FALSE	0.053670385	1	1800	0.131239923	1	1915	0.52487172	1	2154	0.571109542	1	2173	0.99125297	1	2325	0.944645833	1	2309
		Gabor_S8Fp5A0_glszm_HighGrayLevelZoneEmphasis	FALSE	7.10126E-16	1.13265E-12	120	2.21954E-06	0.003540173	1033	0.00011908	0.189933136	1275	0.148892968	1	1933	5.56043E-05	0.088688815	1225	0.01156559	1	1637
		Gabor_S8Fp5A0_glszm_LargeAreaEmphasis	FALSE	0.019158786	1	1687	0.021529852	1	1699	0.181064189	1	1962	0.000124533	0.198630316	1278	0.281021266	1	2033	0.000169719	0.270702329	1299
		Gabor_S8Fp5A0_glszm_LargeAreaHighGrayLevelEmphasis	TRUE	1.4862E-14	2.37049E-11	224	6.49076E-08	0.000103528	849	1.0926E-10	1.74269E-07	562	5.39929E-11	8.61186E-08	533	0.010199378	1	1625	0.214064797	1	1988
		Gabor_S8Fp5A0_glszm_LargeAreaLowGrayLevelEmphasis	FALSE	0.00105931	1	1432	0.924305231	1	2302	0.170570009	1	1953	0.014942445	1	1662	0.343907882	1	2069	0.002196341	1	1490
		Gabor_S8Fp5A0_glszm_LowGrayLevelZoneEmphasis	FALSE	3.10892E-14	4.95873E-11	250	0.000826708	1	1413	0.73672114	1	2236	0.706623996	1	2225	0.139849906	1	1924	0.742240365	1	2238
		Gabor_S8Fp5A0_glszm_SizeZoneNonUniformity	FALSE	0.64257597	1	2201	0.935923232	1	2306	0.840707747	1	2273	0.655722998	1	2206	0.889557128	1	2290	0.340197485	1	2067
		Gabor_S8Fp5A0_glszm_SizeZoneNonUniformityNormalized	FALSE	1.02621E-08	1.63681E-05	761	0.000107253	0.171068248	1268	0.703907896	1	2224	0.402684098	1	2099	0.772816901	1	2249	0.478364009	1	2134
		Gabor_S8Fp5A0_glszm_SmallAreaEmphasis	FALSE	1.45169E-07	0.000231544	889	0.001918871	1	1479	0.687701293	1	2218	0.58359123	1	2178	0.71206247	1	2227	0.576086955	1	2175
		Gabor_S8Fp5A0_glszm_SmallAreaHighGrayLevelEmphasis	TRUE	1.07827E-15	1.71984E-12	134	1.0493E-06	0.001673641	992	3.52909E-06	0.005628895	1059	0.001918871	1	1479	8.12211E-06	0.012954771	1107	0.013637986	1	1653
		Gabor_S8Fp5A0_glszm_SmallAreaLowGrayLevelEmphasis	FALSE	7.61753E-14	1.215E-10	282	0.01180836	1	1639	0.655721116	1	2206	0.800927098	1	2259	0.190788218	1	1970	0.872259057	1	2284
		Gabor_S8Fp5A0_glszm_ZoneEntropy	FALSE	0.080677108	1	1850	0.573595689	1	2174	0.889557128	1	2290	0.682332129	1	2216	0.94464551	1	2309	0.742240365	1	2238
		Gabor_S8Fp5A0_glszm_ZonePercentage	FALSE	0.731216908	1	2234	0.846945898	1	2228	0.840707747	1	2273	0.94173751	1	2308	0.837851369	1	2272	0.962109916	1	2315
		Gabor_S8Fp5A0_glszm_ZoneVariance	FALSE	0.019158786	1	1687	0.020314528	1	1693	0.181064189	1	1962	0.000105655	0.168520006	1267	0.279393712	1	2032	0.000150938	0.240746861	1291
	NGTDM	Gabor_S8Fp5A0_ngtdm_Busyness	TRUE	1.93728E-16	3.08996E-13	77	3.36596E-09	5.36871E-06	710	0.621776014	1	2193	0.018421735	1	1683	0.008788951	1	1611	2.11289E-07	0.000337005	908
		Gabor_S8Fp5A0_ngtdm_Coarseness	FALSE	3.41281E-05	0.0544343	1194	0.002510151	1	1501	0.010526625	1	1628	0.004731276	1	1555	0.013918282	1	1655	0.005928752	1	1575
		Gabor_S8Fp5A0_ngtdm_Complexity	FALSE	2.19321E-15	3.49817E-12	158	9.94864E-05	0.158680859	1263	0.536850824	1	2159	0.458193314	1	2125	0.006551463	1	1584	0.064975454	1	1823
		Gabor_S8Fp5A0_ngtdm_Contrast	FALSE	6.60994E-17	1.05429E-13	42	9.73704E-10	1.55306E-06	655	0.52487172	1	2154	0.314927922	1	2053	0.332845627	1	2063	0.184669228	1	1965
		Gabor_S8Fp5A0_ngtdm_Strength	TRUE	2.15053E-17	3.4301E-14	6	3.12025E-07	0.00049768	928	0.009172988	1	1615	0.566152692	1	2171	1.09462E-08	1.74592E-05	764	0.166057422	1	1949
Gabor - kernel size 8 - frequency = 0.5 - orientation π/4	First Order	Gabor_S8Fp5A45_firstorder_10Percentile	FALSE	0.895335523	1	2292	0.883785268	1	2288	0.98250699	1	2322	0.860759904	1	2280	0.976677441	1	2320	0.812245491	1	2263
		Gabor_S8Fp5A45_firstorder_90Percentile	FALSE	0.935922859	1	2306	0.965022592	1	2316	0.956285864	1	2313	0.767230711	1	2247	0.99125297	1	2325	0.778417474	1	2251
		Gabor_S8Fp5A45_firstorder_Energy	FALSE	0.464860741	1	2128	0.544106049	1	2162	0.973763113	1	2319	0.015246831	1	1664	0.994168581	1	2326	0.015093962	1	1663
		Gabor_S8Fp5A45_firstorder_Entropy	FALSE	0.442837931	1	2118	0.58359123	1	2178	0.92430479	1	2302	0.843567126	1	2274	0.962109695	1	2315	0.840708665	1	2273
		Gabor_S8Fp5A45_firstorder_InterquartileRange	FALSE	0.717517273	1	2229	0.568628535	1	2172	0.985422135	1	2323	0.725728015	1	2232	0.976677441	1	2320	0.81508133	1	2264
		Gabor_S8Fp5A45_firstorder_Kurtosis	TRUE	2.05904E-16	3.28418E-13	79	6.27076E-14	1.00019E-10	275	7.67914E-15	1.22482E-11	201	0.742240365	1	2238	0.023695451	1	1709	0.263473425	1	2022
		Gabor_S8Fp5A45_firstorder_Maximum	FALSE	6.81769E-17	1.08742E-13	43	8.88346E-10	1.41691E-06	651	0.314925095	1	2053	0.58359123	1	2178	0.701197198	1	2223	0.840708665	1	2273
		Gabor_S8Fp5A45_firstorder_MeanAbsoluteDeviation	FALSE	0.921402696	1	2301	0.935923232	1	2306	0.950464366	1	2311	0.800927098	1	2259	0.962109695	1	2315	0.829296174	1	2269
		Gabor_S8Fp5A45_firstorder_Mean	FALSE	1.60462E-10	2.55937E-07	578	0.00279685	1	1510	0.398585183	1	2097	0.829296174	1	2269	0.251191433	1	2014	0.800927098	1	2259
		Gabor_S8Fp5A45_firstorder_Median	FALSE	0.007473568	1	1596	0.795283375	1	2257	0.886670109	1	2289	0.901120052	1	2294	0.933016965	1	2305	0.947554895	1	2310
		Gabor_S8Fp5A45_firstorder_Minimum	FALSE	1.15144E-16	1.83655E-13	60	3.18124E-07	0.000507407	929	0.011930934	1	1640	0.048875799	1	1789	0.558754064	1	2168	0.508336427	1	2147
		Gabor_S8Fp5A45_firstorder_Range	FALSE	5.00011E-17	7.97518E-14	33	5.83857E-09	9.31251E-06	735	0.05644793	1	1806	0.306209971	1	2048	0.50599392	1	2146	0.695788915	1	2221
		Gabor_S8Fp5A45_firstorder_RobustMeanAbsoluteDeviation	FALSE	0.772816901	1	2249	0.676977277	1	2214	0.985422135	1	2323	0.758872214	1	2244	0.994168581	1	2326	0.829296174	1	2269
		Gabor_S8Fp5A45_firstorder_RootMeanSquared	FALSE	0.402681349	1	2099	0.449384032	1	2121	0.85215388	1	2277	0.927208316	1	2303	0.901119478	1	2294	0.898227416	1	2293
		Gabor_S8Fp5A45_firstorder_Skewness	FALSE	4.67179E-07	0.00074515	949	0.002278152	1	1493	0.070467408	1	1833	0.563682033	1	2170	0.453773977	1	2123	0.442840596	1	2118
		Gabor_S8Fp5A45_firstorder_TotalEnergy	FALSE	0.464860741	1	2128	0.546534445	1	2163	0.976677441	1	2320	0.015093962	1	1663	0.994168581	1	2326	0.015093962	1	1663
		Gabor_S8Fp5A45_firstorder_Uniformity	FALSE	0.621776014	1	2193	0.81508133	1	2264	0.938829607	1	2307	0.912703353	1	2298	0.997084271	1	2327	0.860759904	1	2280
		Gabor_S8Fp5A45_firstorder_Variance	FALSE	0.320233409	1	2056	0.345776976	1	2070	0.872258317	1	2284	0.967935735	1	2317	0.997084271	1	2327	0.950464655	1	2311
	GLCM	Gabor_S8Fp5A45_glcm_Autocorrelation	TRUE	2.62412E-14	4.18547E-11	244	1.42336E-07	0.000227026	888	5.44048E-07	0.000867756	957	2.56243E-06	0.004087081	1041	0.020712071	1	1695	0.731218417	1	2234
		Gabor_S8Fp5A45_glcm_ClusterProminence	TRUE	0.014791687	1	1661	0.031080278	1	1738	4.32784E-07	0.000690291	945	1.32776E-08	2.11778E-05	773	0.001073092	1	1433	0.01156559	1	1637
		Gabor_S8Fp5A45_glcm_ClusterShade	TRUE	1.85231E-06	0.002954441	1023	0.001268103	1	1446	0.103911835	1	1883	5.28181E-06	0.008424486	1082	2.44775E-11	3.90416E-08	501	0.00988165	1	1622
		Gabor_S8Fp5A45_glcm_ClusterTendency	FALSE	0.343907882	1	2069	0.294276069	1	2041	0.92430479	1	2302	0.93011237	1	2304	0.953374804	1	2312	0.982507093	1	2322
		Gabor_S8Fp5A45_glcm_Contrast	FALSE	0.351411621	1	2073	0.265036963	1	2023	0.886670109	1	2289	0.962109916	1	2315	0.950464366	1	2311	0.979592198	1	2321
		Gabor_S8Fp5A45_glcm_Correlation	FALSE	6.59256E-05	0.105151347	1236	0.00273067	1	1508	0.442837931	1	2118	0.329212663	1	2061	0.591138569	1	2181	0.357109779	1	2076
		Gabor_S8Fp5A45_glcm_DifferenceAverage	FALSE	0.561214295	1	2169	0.596198554	1	2183	0.927207892	1	2303	0.875138167	1	2285	0.94464551	1	2309	0.869381639	1	2283
		Gabor_S8Fp5A45_glcm_DifferenceEntropy	FALSE	0.515387948	1	2150	0.489776251	1	2139	0.904013444	1	2295	0.886670766	1	2289	0.930111963	1	2304	0.889557769	1	2290
		Gabor_S8Fp5A45_glcm_DifferenceVariance	FALSE	0.119530921	1	1902	0.1110975	1	1892	0.803751758	1	2260	0.642577912	1	2201	0.933016965	1	2305	0.82360325	1	2267
		Gabor_S8Fp5A45_glcm_Id	FALSE	0.698490298	1	2222	0.875138167	1	2285	0.938829607	1	2307	0.912703353	1	2298	0.985422135	1	2323	0.886670766	1	2289
		Gabor_S8Fp5A45_glcm_Idm	FALSE	0.687701293	1	2218	0.840708665	1	2273	0.92430479	1	2302	0.918502123	1	2300	0.979592079	1	2321	0.860759904	1	2280
		Gabor_S8Fp5A45_glcm_Idmn	FALSE	2.05904E-16	3.28418E-13	79	1.63117E-06	0.002601717	1016	0.690392694	1	2219	0.515390402	1	2150	0.297650666	1	2043	0.413034288	1	2104
		Gabor_S8Fp5A45_glcm_Idn	FALSE	1.18735E-16	1.89382E-13	61	2.47099E-07	0.000394123	916	0.614052096	1	2190	0.421426789	1	2108	0.410949003	1	2103	0.434201343	1	2114
		Gabor_S8Fp5A45_glcm_Imc1	FALSE	1.32464E-13	2.11281E-10	302	1.11861E-08	1.78419E-05	765	0.08389878	1	1855	0.099333347	1	1877	0.067674802	1	1828	0.033435965	1	1746
		Gabor_S8Fp5A45_glcm_Imc2	FALSE	3.02906E-15	4.83135E-12	169	0.000387674	0.618339447	1357	0.220876309	1	1993	0.947554895	1	2310	0.485191301	1	2137	0.898227416	1	2293
		Gabor_S8Fp5A45_glcm_InverseVariance	FALSE	2.21526E-11	3.53334E-08	497	9.46602E-06	0.015098299	1116	0.596196407	1	2183	0.46486335	1	2128	0.544103696	1	2162	0.682332129	1	2216
		Gabor_S8Fp5A45_glcm_JointAverage	FALSE	1.02415E-14	1.63352E-11	211	3.12025E-07	0.00049768	928	0.166054948	1	1949	0.566152692	1	2171	0.320233409	1	2056	0.586102738	1	2179
		Gabor_S8Fp5A45_glcm_JointEnergy	FALSE	0.988337474	1	2324	0.956286119	1	2313	0.962109695	1	2315	0.857889624	1	2279	0.918501649	1	2300	0.829296174	1	2269
		Gabor_S8Fp5A45_glcm_JointEntropy	FALSE	0.598732698	1	2184	0.653085267	1	2205	0.92430479	1	2302	0.846427649	1	2275	0.953374804	1	2312	0.863632025	1	2281
		Gabor_S8Fp5A45_glcm_MCC	FALSE	7.00768E-14	1.11773E-10	279	3.87593E-05	0.061821019	1202	0.175176423	1	1957	0.214064797	1	1988	0.136932113	1	1921	0.240782264	1	2007
		Gabor_S8Fp5A45_glcm_MaximumProbability	FALSE	0.786836709	1	2254	0.770022959	1	2248	0.976677441	1	2320	0.909805691	1	2297	0.997084271	1	2327	0.87801893	1	2286
		Gabor_S8Fp5A45_glcm_SumAverage	FALSE	1.02415E-14	1.63352E-11	211	3.12025E-07	0.00049768	928	0.166054948	1	1949	0.566152692	1	2171	0.320233409	1	2056	0.586102738	1	2179
		Gabor_S8Fp5A45_glcm_SumEntropy	FALSE	0.578581096	1	2176	0.591140737	1	2181	0.918501649	1	2300	0.860759904	1	2280	0.947554589	1	2310	0.886670766	1	2289
		Gabor_S8Fp5A45_glcm_SumSquares	FALSE	0.343907882	1	2069	0.281024077	1	2033	0.895335523	1	2292	0.953375076	1	2312	0.962109695	1	2315	0.979592198	1	2321
	GLDM	Gabor_S8Fp5A45_gldm_DependenceEntropy	FALSE	0.125728575	1	1909	0.394516973	1	2095	0.965022388	1	2316	0.798103917	1	2258	0.889557128	1	2290	0.798103917	1	2258
		Gabor_S8Fp5A45_gldm_DependenceNonUniformity	FALSE	0.909805166	1	2297	0.415123078	1	2105	0.329209838	1	2061	0.000784201	1	1409	0.266604087	1	2024	0.000210998	0.336542028	1314
		Gabor_S8Fp5A45_gldm_DependenceNonUniformityNormalized	FALSE	0.593665023	1	2182	0.809412113	1	2262	0.515387948	1	2150	0.611488968	1	2189	0.464860741	1	2128	0.603822015	1	2186
		Gabor_S8Fp5A45_gldm_DependenceVariance	FALSE	0.070467408	1	1833	0.988337543	1	2324	0.655721116	1	2206	0.693089717	1	2220	0.770021653	1	2248	0.784028284	1	2253
		Gabor_S8Fp5A45_gldm_GrayLevelNonUniformity	FALSE	0.930111963	1	2304	0.194534747	1	1973	0.532042731	1	2157	0.230678093	1	2000	0.489773714	1	2139	0.05934559	1	1812
		Gabor_S8Fp5A45_gldm_GrayLevelVariance	FALSE	0.222257606	1	1994	0.210049766	1	1985	0.889557128	1	2290	0.933017355	1	2305	0.970849136	1	2318	0.988337543	1	2324
		Gabor_S8Fp5A45_gldm_HighGrayLevelEmphasis	TRUE	1.57774E-12	2.5165E-09	394	8.1231E-06	0.012956347	1107	5.44048E-07	0.000867756	957	8.9964E-06	0.014349265	1113	0.020712071	1	1695	0.756092244	1	2243
		Gabor_S8Fp5A45_gldm_LargeDependenceEmphasis	FALSE	0.892445612	1	2291	0.852154733	1	2277	0.965022388	1	2316	0.725728015	1	2232	0.94173717	1	2308	0.706623996	1	2225
		Gabor_S8Fp5A45_gldm_LargeDependenceHighGrayLevelEmphasis	TRUE	7.76782E-16	1.23897E-12	123	1.38571E-08	2.21022E-05	775	1.34711E-05	0.02148638	1137	1.18954E-07	0.000189731	879	0.114411427	1	1896	0.097842819	1	1875
		Gabor_S8Fp5A45_gldm_LargeDependenceLowGrayLevelEmphasis	FALSE	2.77214E-11	4.42157E-08	506	0.006198744	1	1579	0.655721116	1	2206	0.950464655	1	2311	0.368670925	1	2082	0.962109916	1	2315
		Gabor_S8Fp5A45_gldm_LowGrayLevelEmphasis	FALSE	8.13521E-15	1.29757E-11	203	1.3585E-06	0.002166804	1006	0.676975497	1	2214	0.619198733	1	2192	0.218132202	1	1991	0.608928526	1	2188
		Gabor_S8Fp5A45_gldm_SmallDependenceEmphasis	FALSE	0.171713015	1	1954	0.145831089	1	1930	0.815080269	1	2264	0.869381639	1	2283	0.935922859	1	2306	0.959197745	1	2314
		Gabor_S8Fp5A45_gldm_SmallDependenceHighGrayLevelEmphasis	TRUE	8.28091E-12	1.3208E-08	458	1.56457E-05	0.024954933	1146	9.41436E-09	1.50159E-05	757	1.87568E-05	0.029917094	1157	0.013637437	1	1653	0.423540439	1	2109
		Gabor_S8Fp5A45_gldm_SmallDependenceLowGrayLevelEmphasis	FALSE	5.02525E-13	8.01527E-10	351	0.000633663	1	1393	0.510679643	1	2148	0.360938787	1	2078	0.139849906	1	1924	0.33467603	1	2064
	GLRLM	Gabor_S8Fp5A45_glrlm_GrayLevelNonUniformity	FALSE	0.875137443	1	2285	0.289258288	1	2038	0.57359345	1	2174	0.510682113	1	2148	0.510679643	1	2148	0.008510804	1	1608
		Gabor_S8Fp5A45_glrlm_GrayLevelNonUniformityNormalized	FALSE	0.820759216	1	2266	0.953375076	1	2312	0.976677441	1	2320	0.770022959	1	2248	0.92430479	1	2302	0.745004234	1	2239
		Gabor_S8Fp5A45_glrlm_GrayLevelVariance	FALSE	0.150958941	1	1935	0.176344927	1	1958	0.770021653	1	2248	0.795283375	1	2257	0.898226825	1	2293	0.991253021	1	2325
		Gabor_S8Fp5A45_glrlm_HighGrayLevelRunEmphasis	TRUE	1.18013E-12	1.88231E-09	383	6.72819E-06	0.010731455	1096	5.44048E-07	0.000867756	957	1.78544E-05	0.028477724	1154	0.020712071	1	1695	0.695788915	1	2221
		Gabor_S8Fp5A45_glrlm_LongRunEmphasis	FALSE	0.857888803	1	2279	0.761655305	1	2245	0.875137443	1	2285	0.598734834	1	2184	0.837851369	1	2272	0.58359123	1	2178
		Gabor_S8Fp5A45_glrlm_LongRunHighGrayLevelEmphasis	TRUE	1.17883E-15	1.88024E-12	137	1.07155E-08	1.70912E-05	763	6.38868E-06	0.010189947	1093	1.16598E-07	0.000185973	878	0.13030671	1	1914	0.083248047	1	1854
		Gabor_S8Fp5A45_glrlm_LongRunLowGrayLevelEmphasis	FALSE	2.8419E-11	4.53282E-08	507	0.005481657	1	1568	0.661009477	1	2208	0.855021221	1	2278	0.372576783	1	2084	0.852154733	1	2277
		Gabor_S8Fp5A45_glrlm_LowGrayLevelRunEmphasis	FALSE	1.21653E-14	1.94036E-11	217	1.38366E-06	0.002206931	1007	0.674304148	1	2213	0.558756361	1	2168	0.163831404	1	1947	0.578583315	1	2176
		Gabor_S8Fp5A45_glrlm_RunEntropy	FALSE	1.63094E-06	0.002601353	1016	0.012957642	1	1648	0.786836709	1	2254	0.883785268	1	2288	0.883784593	1	2288	0.956286119	1	2313
		Gabor_S8Fp5A45_glrlm_RunLengthNonUniformity	FALSE	0.915601686	1	2299	0.167177676	1	1950	0.79246432	1	2256	0.079418652	1	1848	0.840707747	1	2273	0.413034288	1	2104
		Gabor_S8Fp5A45_glrlm_RunLengthNonUniformityNormalized	FALSE	0.750540389	1	2241	0.967935735	1	2317	0.997084271	1	2327	0.725728015	1	2232	0.988337474	1	2324	0.742240365	1	2238
		Gabor_S8Fp5A45_glrlm_RunPercentage	FALSE	0.795282205	1	2257	0.979270885	1	2273	0.965022388	1	2316	0.739479817	1	2237	0.997084271	1	2327	0.770022959	1	2248
		Gabor_S8Fp5A45_glrlm_RunVariance	FALSE	0.661009477	1	2208	0.510682113	1	2148	0.817918548	1	2265	0.534446436	1	2158	0.809411021	1	2262	0.515390402	1	2150
		Gabor_S8Fp5A45_glrlm_ShortRunEmphasis	FALSE	0.679650915	1	2215	0.81508133	1	2264	0.994168581	1	2326	0.756092244	1	2243	0.973763113	1	2319	0.764441481	1	2246
		Gabor_S8Fp5A45_glrlm_ShortRunHighGrayLevelEmphasis	TRUE	2.40012E-12	3.82819E-09	410	3.52367E-05	0.05620257	1196	1.04915E-06	0.001673399	992	0.000167257	0.26677482	1298	0.052324979	1	1797	0.505996405	1	2146
		Gabor_S8Fp5A45_glrlm_ShortRunLowGrayLevelEmphasis	FALSE	6.64619E-15	1.06007E-11	196	1.01109E-06	0.001612687	990	0.52487172	1	2154	0.503662062	1	2145	0.111917218	1	1893	0.460410674	1	2126
		Gabor_S8Fp5A45_glszm_GrayLevelNonUniformity	FALSE	9.34713E-08	0.000149087	867	0.000117328	0.18713768	1274	0.815080269	1	2264	0.104693338	1	1884	0.94464551	1	2309	0.000265318	0.423181675	1330
		Gabor_S8Fp5A45_glszm_GrayLevelNonUniformityNormalized	FALSE	1.56669E-10	2.49887E-07	577	1.15894E-05	0.018485037	1128	0.953374804	1	2312	0.857889624	1	2279	0.94464551	1	2309	0.826448572	1	2268

	GLSZM	Gabor_S8Fp5A45_glszm_GrayLevelVariance	FALSE	[illegible]	[illegible]	[illegible]	[illegible]	[illegible]	[illegible]	[illegible]	[illegible]	[illegible]	[illegible]	[illegible]	[illegible]	[illegible]	[illegible]	[illegible]	[illegible]	[illegible]	[illegible]
		Gabor_S8Fp5A45_glszm_HighGrayLevelZoneEmphasis	TRUE	[illegible]	[illegible]	[illegible]	[illegible]	[illegible]	[illegible]	[illegible]	[illegible]	[illegible]	[illegible]	[illegible]	[illegible]	[illegible]	[illegible]	[illegible]	[illegible]	[illegible]	[illegible]
		Gabor_S8Fp5A45_glszm_LargeAreaEmphasis	FALSE	[illegible]	[illegible]	[illegible]	[illegible]	[illegible]	[illegible]	[illegible]	[illegible]	[illegible]	[illegible]	[illegible]	[illegible]	[illegible]	[illegible]	[illegible]	[illegible]	[illegible]	[illegible]
		Gabor_S8Fp5A45_glszm_LargeAreaHighGrayLevelEmphasis	TRUE	[illegible]	[illegible]	[illegible]	[illegible]	[illegible]	[illegible]	[illegible]	[illegible]	[illegible]	[illegible]	[illegible]	[illegible]	[illegible]	[illegible]	[illegible]	[illegible]	[illegible]	[illegible]
		Gabor_S8Fp5A45_glszm_LargeAreaLowGrayLevelEmphasis	FALSE	[illegible]	[illegible]	[illegible]	[illegible]	[illegible]	[illegible]	[illegible]	[illegible]	[illegible]	[illegible]	[illegible]	[illegible]	[illegible]	[illegible]	[illegible]	[illegible]	[illegible]	[illegible]
		Gabor_S8Fp5A45_glszm_LowGrayLevelZoneEmphasis	FALSE	[illegible]	[illegible]	[illegible]	[illegible]	[illegible]	[illegible]	[illegible]	[illegible]	[illegible]	[illegible]	[illegible]	[illegible]	[illegible]	[illegible]	[illegible]	[illegible]	[illegible]	[illegible]
		Gabor_S8Fp5A45_glszm_SizeZoneNonUniformity	FALSE	[illegible]	[illegible]	[illegible]	[illegible]	[illegible]	[illegible]	[illegible]	[illegible]	[illegible]	[illegible]	[illegible]	[illegible]	[illegible]	[illegible]	[illegible]	[illegible]	[illegible]	[illegible]
		Gabor_S8Fp5A45_glszm_SizeZoneNonUniformityNormalized	FALSE	[illegible]	[illegible]	[illegible]	[illegible]	[illegible]	[illegible]	[illegible]	[illegible]	[illegible]	[illegible]	[illegible]	[illegible]	[illegible]	[illegible]	[illegible]	[illegible]	[illegible]	[illegible]
		Gabor_S8Fp5A45_glszm_SmallAreaEmphasis	FALSE	[illegible]	[illegible]	[illegible]	[illegible]	[illegible]	[illegible]	[illegible]	[illegible]	[illegible]	[illegible]	[illegible]	[illegible]	[illegible]	[illegible]	[illegible]	[illegible]	[illegible]	[illegible]
		Gabor_S8Fp5A45_glszm_SmallAreaHighGrayLevelEmphasis	TRUE	[illegible]	[illegible]	[illegible]	[illegible]	[illegible]	[illegible]	[illegible]	[illegible]	[illegible]	[illegible]	[illegible]	[illegible]	[illegible]	[illegible]	[illegible]	[illegible]	[illegible]	[illegible]
		Gabor_S8Fp5A45_glszm_SmallAreaLowGrayLevelEmphasis	FALSE	[illegible]	[illegible]	[illegible]	[illegible]	[illegible]	[illegible]	[illegible]	[illegible]	[illegible]	[illegible]	[illegible]	[illegible]	[illegible]	[illegible]	[illegible]	[illegible]	[illegible]	[illegible]
		Gabor_S8Fp5A45_glszm_ZoneEntropy	FALSE	[illegible]	[illegible]	[illegible]	[illegible]	[illegible]	[illegible]	[illegible]	[illegible]	[illegible]	[illegible]	[illegible]	[illegible]	[illegible]	[illegible]	[illegible]	[illegible]	[illegible]	[illegible]
		Gabor_S8Fp5A45_glszm_ZonePercentage	FALSE	[illegible]	[illegible]	[illegible]	[illegible]	[illegible]	[illegible]	[illegible]	[illegible]	[illegible]	[illegible]	[illegible]	[illegible]	[illegible]	[illegible]	[illegible]	[illegible]	[illegible]	[illegible]
		Gabor_S8Fp5A45_glszm_ZoneVariance	FALSE	[illegible]	[illegible]	[illegible]	[illegible]	[illegible]	[illegible]	[illegible]	[illegible]	[illegible]	[illegible]	[illegible]	[illegible]	[illegible]	[illegible]	[illegible]	[illegible]	[illegible]	[illegible]
	NGTDM	Gabor_S8Fp5A45_ngtdm_Busyness	FALSE	[illegible]	[illegible]	[illegible]	[illegible]	[illegible]	[illegible]	[illegible]	[illegible]	[illegible]	[illegible]	[illegible]	[illegible]	[illegible]	[illegible]	[illegible]	[illegible]	[illegible]	[illegible]
		Gabor_S8Fp5A45_ngtdm_Coarseness	FALSE	[illegible]	[illegible]	[illegible]	[illegible]	[illegible]	[illegible]	[illegible]	[illegible]	[illegible]	[illegible]	[illegible]	[illegible]	[illegible]	[illegible]	[illegible]	[illegible]	[illegible]	[illegible]
		Gabor_S8Fp5A45_ngtdm_Complexity	FALSE	[illegible]	[illegible]	[illegible]	[illegible]	[illegible]	[illegible]	[illegible]	[illegible]	[illegible]	[illegible]	[illegible]	[illegible]	[illegible]	[illegible]	[illegible]	[illegible]	[illegible]	[illegible]
		Gabor_S8Fp5A45_ngtdm_Contrast	FALSE	[illegible]	[illegible]	[illegible]	[illegible]	[illegible]	[illegible]	[illegible]	[illegible]	[illegible]	[illegible]	[illegible]	[illegible]	[illegible]	[illegible]	[illegible]	[illegible]	[illegible]	[illegible]
		Gabor_S8Fp5A45_ngtdm_Strength	TRUE	[illegible]	[illegible]	[illegible]	[illegible]	[illegible]	[illegible]	[illegible]	[illegible]	[illegible]	[illegible]	[illegible]	[illegible]	[illegible]	[illegible]	[illegible]	[illegible]	[illegible]	[illegible]
Gabor - kernel size 8 - frequency = 0.5 - orientation π/2	First Order	Gabor_S8Fp5A90_firstorder_10Percentile	FALSE	[illegible]	[illegible]	[illegible]	[illegible]	[illegible]	[illegible]	[illegible]	[illegible]	[illegible]	[illegible]	[illegible]	[illegible]	[illegible]	[illegible]	[illegible]	[illegible]	[illegible]	[illegible]
		Gabor_S8Fp5A90_firstorder_90Percentile	FALSE	[illegible]	[illegible]	[illegible]	[illegible]	[illegible]	[illegible]	[illegible]	[illegible]	[illegible]	[illegible]	[illegible]	[illegible]	[illegible]	[illegible]	[illegible]	[illegible]	[illegible]	[illegible]
		Gabor_S8Fp5A90_firstorder_Energy	FALSE	[illegible]	[illegible]	[illegible]	[illegible]	[illegible]	[illegible]	[illegible]	[illegible]	[illegible]	[illegible]	[illegible]	[illegible]	[illegible]	[illegible]	[illegible]	[illegible]	[illegible]	[illegible]
		Gabor_S8Fp5A90_firstorder_Entropy	FALSE	[illegible]	[illegible]	[illegible]	[illegible]	[illegible]	[illegible]	[illegible]	[illegible]	[illegible]	[illegible]	[illegible]	[illegible]	[illegible]	[illegible]	[illegible]	[illegible]	[illegible]	[illegible]
		Gabor_S8Fp5A90_firstorder_InterquartileRange	FALSE	[illegible]	[illegible]	[illegible]	[illegible]	[illegible]	[illegible]	[illegible]	[illegible]	[illegible]	[illegible]	[illegible]	[illegible]	[illegible]	[illegible]	[illegible]	[illegible]	[illegible]	[illegible]
		Gabor_S8Fp5A90_firstorder_Kurtosis	TRUE	[illegible]	[illegible]	[illegible]	[illegible]	[illegible]	[illegible]	[illegible]	[illegible]	[illegible]	[illegible]	[illegible]	[illegible]	[illegible]	[illegible]	[illegible]	[illegible]	[illegible]	[illegible]
		Gabor_S8Fp5A90_firstorder_Maximum	FALSE	[illegible]	[illegible]	[illegible]	[illegible]	[illegible]	[illegible]	[illegible]	[illegible]	[illegible]	[illegible]	[illegible]	[illegible]	[illegible]	[illegible]	[illegible]	[illegible]	[illegible]	[illegible]
		Gabor_S8Fp5A90_firstorder_MeanAbsoluteDeviation	FALSE	[illegible]	[illegible]	[illegible]	[illegible]	[illegible]	[illegible]	[illegible]	[illegible]	[illegible]	[illegible]	[illegible]	[illegible]	[illegible]	[illegible]	[illegible]	[illegible]	[illegible]	[illegible]
		Gabor_S8Fp5A90_firstorder_Mean	FALSE	[illegible]	[illegible]	[illegible]	[illegible]	[illegible]	[illegible]	[illegible]	[illegible]	[illegible]	[illegible]	[illegible]	[illegible]	[illegible]	[illegible]	[illegible]	[illegible]	[illegible]	[illegible]
		Gabor_S8Fp5A90_firstorder_Median	FALSE	[illegible]	[illegible]	[illegible]	[illegible]	[illegible]	[illegible]	[illegible]	[illegible]	[illegible]	[illegible]	[illegible]	[illegible]	[illegible]	[illegible]	[illegible]	[illegible]	[illegible]	[illegible]
		Gabor_S8Fp5A90_firstorder_Minimum	FALSE	[illegible]	[illegible]	[illegible]	[illegible]	[illegible]	[illegible]	[illegible]	[illegible]	[illegible]	[illegible]	[illegible]	[illegible]	[illegible]	[illegible]	[illegible]	[illegible]	[illegible]	[illegible]
		Gabor_S8Fp5A90_firstorder_Range	FALSE	[illegible]	[illegible]	[illegible]	[illegible]	[illegible]	[illegible]	[illegible]	[illegible]	[illegible]	[illegible]	[illegible]	[illegible]	[illegible]	[illegible]	[illegible]	[illegible]	[illegible]	[illegible]
		Gabor_S8Fp5A90_firstorder_RobustMeanAbsoluteDeviation	FALSE	[illegible]	[illegible]	[illegible]	[illegible]	[illegible]	[illegible]	[illegible]	[illegible]	[illegible]	[illegible]	[illegible]	[illegible]	[illegible]	[illegible]	[illegible]	[illegible]	[illegible]	[illegible]
		Gabor_S8Fp5A90_firstorder_RootMeanSquared	FALSE	[illegible]	[illegible]	[illegible]	[illegible]	[illegible]	[illegible]	[illegible]	[illegible]	[illegible]	[illegible]	[illegible]	[illegible]	[illegible]	[illegible]	[illegible]	[illegible]	[illegible]	[illegible]
		Gabor_S8Fp5A90_firstorder_Skewness	FALSE	[illegible]	[illegible]	[illegible]	[illegible]	[illegible]	[illegible]	[illegible]	[illegible]	[illegible]	[illegible]	[illegible]	[illegible]	[illegible]	[illegible]	[illegible]	[illegible]	[illegible]	[illegible]
		Gabor_S8Fp5A90_firstorder_TotalEnergy	FALSE	[illegible]	[illegible]	[illegible]	[illegible]	[illegible]	[illegible]	[illegible]	[illegible]	[illegible]	[illegible]	[illegible]	[illegible]	[illegible]	[illegible]	[illegible]	[illegible]	[illegible]	[illegible]
		Gabor_S8Fp5A90_firstorder_Uniformity	FALSE	[illegible]	[illegible]	[illegible]	[illegible]	[illegible]	[illegible]	[illegible]	[illegible]	[illegible]	[illegible]	[illegible]	[illegible]	[illegible]	[illegible]	[illegible]	[illegible]	[illegible]	[illegible]
		Gabor_S8Fp5A90_firstorder_Variance	FALSE	[illegible]	[illegible]	[illegible]	[illegible]	[illegible]	[illegible]	[illegible]	[illegible]	[illegible]	[illegible]	[illegible]	[illegible]	[illegible]	[illegible]	[illegible]	[illegible]	[illegible]	[illegible]
	GLCM	Gabor_S8Fp5A90_glcm_Autocorrelation	TRUE	[illegible]	[illegible]	[illegible]	[illegible]	[illegible]	[illegible]	[illegible]	[illegible]	[illegible]	[illegible]	[illegible]	[illegible]	[illegible]	[illegible]	[illegible]	[illegible]	[illegible]	[illegible]
		Gabor_S8Fp5A90_glcm_ClusterProminence	TRUE	[illegible]	[illegible]	[illegible]	[illegible]	[illegible]	[illegible]	[illegible]	[illegible]	[illegible]	[illegible]	[illegible]	[illegible]	[illegible]	[illegible]	[illegible]	[illegible]	[illegible]	[illegible]
		Gabor_S8Fp5A90_glcm_ClusterShade	TRUE	[illegible]	[illegible]	[illegible]	[illegible]	[illegible]	[illegible]	[illegible]	[illegible]	[illegible]	[illegible]	[illegible]	[illegible]	[illegible]	[illegible]	[illegible]	[illegible]	[illegible]	[illegible]
		Gabor_S8Fp5A90_glcm_ClusterTendency	FALSE	[illegible]	[illegible]	[illegible]	[illegible]	[illegible]	[illegible]	[illegible]	[illegible]	[illegible]	[illegible]	[illegible]	[illegible]	[illegible]	[illegible]	[illegible]	[illegible]	[illegible]	[illegible]
		Gabor_S8Fp5A90_glcm_Contrast	FALSE	[illegible]	[illegible]	[illegible]	[illegible]	[illegible]	[illegible]	[illegible]	[illegible]	[illegible]	[illegible]	[illegible]	[illegible]	[illegible]	[illegible]	[illegible]	[illegible]	[illegible]	[illegible]
		Gabor_S8Fp5A90_glcm_Correlation	FALSE	[illegible]	[illegible]	[illegible]	[illegible]	[illegible]	[illegible]	[illegible]	[illegible]	[illegible]	[illegible]	[illegible]	[illegible]	[illegible]	[illegible]	[illegible]	[illegible]	[illegible]	[illegible]
		Gabor_S8Fp5A90_glcm_DifferenceAverage	FALSE	[illegible]	[illegible]	[illegible]	[illegible]	[illegible]	[illegible]	[illegible]	[illegible]	[illegible]	[illegible]	[illegible]	[illegible]	[illegible]	[illegible]	[illegible]	[illegible]	[illegible]	[illegible]
		Gabor_S8Fp5A90_glcm_DifferenceEntropy	FALSE	[illegible]	[illegible]	[illegible]	[illegible]	[illegible]	[illegible]	[illegible]	[illegible]	[illegible]	[illegible]	[illegible]	[illegible]	[illegible]	[illegible]	[illegible]	[illegible]	[illegible]	[illegible]
		Gabor_S8Fp5A90_glcm_DifferenceVariance	FALSE	[illegible]	[illegible]	[illegible]	[illegible]	[illegible]	[illegible]	[illegible]	[illegible]	[illegible]	[illegible]	[illegible]	[illegible]	[illegible]	[illegible]	[illegible]	[illegible]	[illegible]	[illegible]
		Gabor_S8Fp5A90_glcm_Id	FALSE	[illegible]	[illegible]	[illegible]	[illegible]	[illegible]	[illegible]	[illegible]	[illegible]	[illegible]	[illegible]	[illegible]	[illegible]	[illegible]	[illegible]	[illegible]	[illegible]	[illegible]	[illegible]
		Gabor_S8Fp5A90_glcm_Idm	FALSE	[illegible]	[illegible]	[illegible]	[illegible]	[illegible]	[illegible]	[illegible]	[illegible]	[illegible]	[illegible]	[illegible]	[illegible]	[illegible]	[illegible]	[illegible]	[illegible]	[illegible]	[illegible]
		Gabor_S8Fp5A90_glcm_Idmn	FALSE	[illegible]	[illegible]	[illegible]	[illegible]	[illegible]	[illegible]	[illegible]	[illegible]	[illegible]	[illegible]	[illegible]	[illegible]	[illegible]	[illegible]	[illegible]	[illegible]	[illegible]	[illegible]
		Gabor_S8Fp5A90_glcm_Idn	FALSE	[illegible]	[illegible]	[illegible]	[illegible]	[illegible]	[illegible]	[illegible]	[illegible]	[illegible]	[illegible]	[illegible]	[illegible]	[illegible]	[illegible]	[illegible]	[illegible]	[illegible]	[illegible]
		Gabor_S8Fp5A90_glcm_Imc1	FALSE	[illegible]	[illegible]	[illegible]	[illegible]	[illegible]	[illegible]	[illegible]	[illegible]	[illegible]	[illegible]	[illegible]	[illegible]	[illegible]	[illegible]	[illegible]	[illegible]	[illegible]	[illegible]
		Gabor_S8Fp5A90_glcm_Imc2	FALSE	[illegible]	[illegible]	[illegible]	[illegible]	[illegible]	[illegible]	[illegible]	[illegible]	[illegible]	[illegible]	[illegible]	[illegible]	[illegible]	[illegible]	[illegible]	[illegible]	[illegible]	[illegible]
		Gabor_S8Fp5A90_glcm_InverseVariance	FALSE	[illegible]	[illegible]	[illegible]	[illegible]	[illegible]	[illegible]	[illegible]	[illegible]	[illegible]	[illegible]	[illegible]	[illegible]	[illegible]	[illegible]	[illegible]	[illegible]	[illegible]	[illegible]
		Gabor_S8Fp5A90_glcm_JointAverage	FALSE	[illegible]	[illegible]	[illegible]	[illegible]	[illegible]	[illegible]	[illegible]	[illegible]	[illegible]	[illegible]	[illegible]	[illegible]	[illegible]	[illegible]	[illegible]	[illegible]	[illegible]	[illegible]
		Gabor_S8Fp5A90_glcm_JointEnergy	FALSE	[illegible]	[illegible]	[illegible]	[illegible]	[illegible]	[illegible]	[illegible]	[illegible]	[illegible]	[illegible]	[illegible]	[illegible]	[illegible]	[illegible]	[illegible]	[illegible]	[illegible]	[illegible]
		Gabor_S8Fp5A90_glcm_JointEntropy	FALSE	[illegible]	[illegible]	[illegible]	[illegible]	[illegible]	[illegible]	[illegible]	[illegible]	[illegible]	[illegible]	[illegible]	[illegible]	[illegible]	[illegible]	[illegible]	[illegible]	[illegible]	[illegible]
		Gabor_S8Fp5A90_glcm_MCC	FALSE	[illegible]	[illegible]	[illegible]	[illegible]	[illegible]	[illegible]	[illegible]	[illegible]	[illegible]	[illegible]	[illegible]	[illegible]	[illegible]	[illegible]	[illegible]	[illegible]	[illegible]	[illegible]
		Gabor_S8Fp5A90_glcm_MaximumProbability	FALSE	[illegible]	[illegible]	[illegible]	[illegible]	[illegible]	[illegible]	[illegible]	[illegible]	[illegible]	[illegible]	[illegible]	[illegible]	[illegible]	[illegible]	[illegible]	[illegible]	[illegible]	[illegible]
		Gabor_S8Fp5A90_glcm_SumAverage	FALSE	[illegible]	[illegible]	[illegible]	[illegible]	[illegible]	[illegible]	[illegible]	[illegible]	[illegible]	[illegible]	[illegible]	[illegible]	[illegible]	[illegible]	[illegible]	[illegible]	[illegible]	[illegible]
		Gabor_S8Fp5A90_glcm_SumEntropy	FALSE	[illegible]	[illegible]	[illegible]	[illegible]	[illegible]	[illegible]	[illegible]	[illegible]	[illegible]	[illegible]	[illegible]	[illegible]	[illegible]	[illegible]	[illegible]	[illegible]	[illegible]	[illegible]
		Gabor_S8Fp5A90_glcm_SumSquares	FALSE	[illegible]	[illegible]	[illegible]	[illegible]	[illegible]	[illegible]	[illegible]	[illegible]	[illegible]	[illegible]	[illegible]	[illegible]	[illegible]	[illegible]	[illegible]	[illegible]	[illegible]	[illegible]
	GLDM	Gabor_S8Fp5A90_gldm_DependenceEntropy	FALSE	[illegible]	[illegible]	[illegible]	[illegible]	[illegible]	[illegible]	[illegible]	[illegible]	[illegible]	[illegible]	[illegible]	[illegible]	[illegible]	[illegible]	[illegible]	[illegible]	[illegible]	[illegible]
		Gabor_S8Fp5A90_gldm_DependenceNonUniformity	FALSE	[illegible]	[illegible]	[illegible]	[illegible]	[illegible]	[illegible]	[illegible]	[illegible]	[illegible]	[illegible]	[illegible]	[illegible]	[illegible]	[illegible]	[illegible]	[illegible]	[illegible]	[illegible]
		Gabor_S8Fp5A90_gldm_DependenceNonUniformityNormalized	FALSE	[illegible]	[illegible]	[illegible]	[illegible]	[illegible]	[illegible]	[illegible]	[illegible]	[illegible]	[illegible]	[illegible]	[illegible]	[illegible]	[illegible]	[illegible]	[illegible]	[illegible]	[illegible]
		Gabor_S8Fp5A90_gldm_DependenceVariance	FALSE	[illegible]	[illegible]	[illegible]	[illegible]	[illegible]	[illegible]	[illegible]	[illegible]	[illegible]	[illegible]	[illegible]	[illegible]	[illegible]	[illegible]	[illegible]	[illegible]	[illegible]	[illegible]
		Gabor_S8Fp5A90_gldm_GrayLevelNonUniformity	FALSE	[illegible]	[illegible]	[illegible]	[illegible]	[illegible]	[illegible]	[illegible]	[illegible]	[illegible]	[illegible]	[illegible]	[illegible]	[illegible]	[illegible]	[illegible]	[illegible]	[illegible]	[illegible]
		Gabor_S8Fp5A90_gldm_GrayLevelVariance	FALSE	[illegible]	[illegible]	[illegible]	[illegible]	[illegible]	[illegible]	[illegible]	[illegible]	[illegible]	[illegible]	[illegible]	[illegible]	[illegible]	[illegible]	[illegible]	[illegible]	[illegible]	[illegible]
		Gabor_S8Fp5A90_gldm_HighGrayLevelEmphasis	TRUE	[illegible]	[illegible]	[illegible]	[illegible]	[illegible]	[illegible]	[illegible]	[illegible]	[illegible]	[illegible]	[illegible]	[illegible]	[illegible]	[illegible]	[illegible]	[illegible]	[illegible]	[illegible]
		Gabor_S8Fp5A90_gldm_LargeDependenceEmphasis	FALSE	[illegible]	[illegible]	[illegible]	[illegible]	[illegible]	[illegible]	[illegible]	[illegible]	[illegible]	[illegible]	[illegible]	[illegible]	[illegible]	[illegible]	[illegible]	[illegible]	[illegible]	[illegible]
		Gabor_S8Fp5A90_gldm_LargeDependenceHighGrayLevelEmphasis	TRUE	[illegible]	[illegible]	[illegible]	[illegible]	[illegible]	[illegible]	[illegible]	[illegible]	[illegible]	[illegible]	[illegible]	[illegible]	[illegible]	[illegible]	[illegible]	[illegible]	[illegible]	[illegible]
		Gabor_S8Fp5A90_gldm_LargeDependenceLowGrayLevelEmphasis	FALSE	[illegible]	[illegible]	[illegible]	[illegible]	[illegible]	[illegible]	[illegible]	[illegible]	[illegible]	[illegible]	[illegible]	[illegible]	[illegible]	[illegible]	[illegible]	[illegible]	[illegible]	[illegible]
		Gabor_S8Fp5A90_gldm_LowGrayLevelEmphasis	FALSE	[illegible]	[illegible]	[illegible]	[illegible]	[illegible]	[illegible]	[illegible]	[illegible]	[illegible]	[illegible]	[illegible]	[illegible]	[illegible]	[illegible]	[illegible]	[illegible]	[illegible]	[illegible]
		Gabor_S8Fp5A90_gldm_SmallDependenceEmphasis	FALSE	[illegible]	[illegible]	[illegible]	[illegible]	[illegible]	[illegible]	[illegible]	[illegible]	[illegible]	[illegible]	[illegible]	[illegible]	[illegible]	[illegible]	[illegible]	[illegible]	[illegible]	[illegible]
		Gabor_S8Fp5A90_gldm_SmallDependenceHighGrayLevelEmphasis	TRUE	[illegible]	[illegible]	[illegible]	[illegible]	[illegible]	[illegible]	[illegible]	[illegible]	[illegible]	[illegible]	[illegible]	[illegible]	[illegible]	[illegible]	[illegible]	[illegible]	[illegible]	[illegible]
		Gabor_S8Fp5A90_gldm_SmallDependenceLowGrayLevelEmphasis	FALSE	[illegible]	[illegible]	[illegible]	[illegible]	[illegible]	[illegible]	[illegible]	[illegible]	[illegible]	[illegible]	[illegible]	[illegible]	[illegible]	[illegible]	[illegible]	[illegible]	[illegible]	[illegible]
	GLRLM	Gabor_S8Fp5A90_glrlm_GrayLevelNonUniformity	FALSE	[illegible]	[illegible]	[illegible]	[illegible]	[illegible]	[illegible]	[illegible]	[illegible]	[illegible]	[illegible]	[illegible]	[illegible]	[illegible]	[illegible]	[illegible]	[illegible]	[illegible]	[illegible]
		Gabor_S8Fp5A90_glrlm_GrayLevelNonUniformityNormalized	FALSE	[illegible]	[illegible]	[illegible]	[illegible]	[illegible]	[illegible]	[illegible]	[illegible]	[illegible]	[illegible]	[illegible]	[illegible]	[illegible]	[illegible]	[illegible]	[illegible]	[illegible]	[illegible]
		Gabor_S8Fp5A90_glrlm_GrayLevelVariance	FALSE	[illegible]	[illegible]	[illegible]	[illegible]	[illegible]	[illegible]	[illegible]	[illegible]	[illegible]	[illegible]	[illegible]	[illegible]	[illegible]	[illegible]	[illegible]	[illegible]	[illegible]	[illegible]
		Gabor_S8Fp5A90_glrlm_HighGrayLevelRunEmphasis	TRUE	[illegible]	[illegible]	[illegible]	[illegible]	[illegible]	[illegible]	[illegible]	[illegible]	[illegible]	[illegible]	[illegible]	[illegible]	[illegible]	[illegible]	[illegible]	[illegible]	[illegible]	[illegible]
		Gabor_S8Fp5A90_glrlm_LongRunEmphasis	FALSE	[illegible]	[illegible]	[illegible]	[illegible]	[illegible]	[illegible]	[illegible]	[illegible]	[illegible]	[illegible]	[illegible]	[illegible]	[illegible]	[illegible]	[illegible]	[illegible]	[illegible]	[illegible]
		Gabor_S8Fp5A90_glrlm_LongRunHighGrayLevelEmphasis	TRUE	[illegible]	[illegible]	[illegible]	[illegible]	[illegible]	[illegible]	[illegible]	[illegible]	[illegible]	[illegible]	[illegible]	[illegible]	[illegible]	[illegible]	[illegible]	[illegible]	[illegible]	[illegible]
		Gabor_S8Fp5A90_glrlm_LongRunLowGrayLevelEmphasis	FALSE	[illegible]	[illegible]	[illegible]	[illegible]	[illegible]	[illegible]	[illegible]	[illegible]	[illegible]	[illegible]	[illegible]	[illegible]	[illegible]	[illegible]	[illegible]	[illegible]	[illegible]	[illegible]
		Gabor_S8Fp5A90_glrlm_LowGrayLevelRunEmphasis	FALSE	[illegible]	[illegible]	[illegible]	[illegible]	[illegible]	[illegible]	[illegible]	[illegible]	[illegible]	[illegible]	[illegible]	[illegible]	[illegible]	[illegible]	[illegible]	[illegible]	[illegible]	[illegible]
		Gabor_S8Fp5A90_glrlm_RunEntropy	FALSE	[illegible]	[illegible]	[illegible]	[illegible]	[illegible]	[illegible]	[illegible]	[illegible]	[illegible]	[illegible]	[illegible]	[illegible]	[illegible]	[illegible]	[illegible]	[illegible]	[illegible]	[illegible]
		Gabor_S8Fp5A90_glrlm_RunLengthNonUniformity	FALSE	[illegible]	[illegible]	[illegible]	[illegible]	[illegible]	[illegible]	[illegible]	[illegible]	[illegible]	[illegible]	[illegible]	[illegible]	[illegible]	[illegible]	[illegible]	[illegible]	[illegible]	[illegible]
		Gabor_S8Fp5A90_glrlm_RunLengthNonUniformityNormalized	FALSE	[illegible]	[illegible]	[illegible]	[illegible]	[illegible]	[illegible]	[illegible]	[illegible]	[illegible]	[illegible]	[illegible]	[illegible]	[illegible]	[illegible]	[illegible]	[illegible]	[illegible]	[illegible]
		Gabor_S8Fp5A90_glrlm_RunPercentage	FALSE	[illegible]	[illegible]	[illegible]	[illegible]	[illegible]	[illegible]	[illegible]	[illegible]	[illegible]	[illegible]	[illegible]	[illegible]	[illegible]	[illegible]	[illegible]	[illegible]	[illegible]	[illegible]
		Gabor_S8Fp5A90_glrlm_RunVariance	FALSE	[illegible]	[illegible]	[illegible]	[illegible]	[illegible]	[illegible]	[illegible]	[illegible]	[illegible]	[illegible]	[illegible]	[illegible]	[illegible]	[illegible]	[illegible]	[illegible]	[illegible]	[illegible]
		Gabor_S8Fp5A90_glrlm_ShortRunEmphasis	FALSE	[illegible]	[illegible]	[illegible]	[illegible]	[illegible]	[illegible]	[illegible]	[illegible]	[illegible]	[illegible]	[illegible]	[illegible]	[illegible]	[illegible]	[illegible]	[illegible]	[illegible]	[illegible]
		Gabor_S8Fp5A90_glrlm_ShortRunHighGrayLevelEmphasis	TRUE	[illegible]	[illegible]	[illegible]	[illegible]	[illegible]	[illegible]	[illegible]	[illegible]	[illegible]	[illegible]	[illegible]	[illegible]	[illegible]	[illegible]	[illegible]	[illegible]	[illegible]	[illegible]
		Gabor_S8Fp5A90_glrlm_ShortRunLowGrayLevelEmphasis	FALSE	[illegible]	[illegible]	[illegible]	[illegible]	[illegible]	[illegible]	[illegible]	[illegible]	[illegible]	[illegible]	[illegible]	[illegible]	[illegible]	[illegible]	[illegible]	[illegible]	[illegible]	[illegible]
	GLSZM	Gabor_S8Fp5A90_glszm_GrayLevelNonUniformity	FALSE	[illegible]	[illegible]	[illegible]	[illegible]	[illegible]	[illegible]	[illegible]	[illegible]	[illegible]	[illegible]	[illegible]	[illegible]	[illegible]	[illegible]	[illegible]	[illegible]	[illegible]	[illegible]
		Gabor_S8Fp5A90_glszm_GrayLevelNonUniformityNormalized	FALSE	[illegible]	[illegible]	[illegible]	[illegible]	[illegible]	[illegible]	[illegible]	[illegible]	[illegible]	[illegible]	[illegible]	[illegible]	[illegible]	[illegible]	[illegible]	[illegible]	[illegible]	[illegible]
		Gabor_S8Fp5A90_glszm_GrayLevelVariance	FALSE	[illegible]	[illegible]	[illegible]	[illegible]	[illegible]	[illegible]	[illegible]	[illegible]	[illegible]	[illegible]	[illegible]	[illegible]	[illegible]	[illegible]	[illegible]	[illegible]	[illegible]	[illegible]
		Gabor_S8Fp5A90_glszm_HighGrayLevelZoneEmphasis	TRUE	[illegible]	[illegible]	[illegible]	[illegible]	[illegible]	[illegible]	[illegible]	[illegible]	[illegible]	[illegible]	[illegible]	[illegible]	[illegible]	[illegible]	[illegible]	[illegible]	[illegible]	[illegible]
		Gabor_S8Fp5A90_glszm_LargeAreaEmphasis	FALSE	[illegible]	[illegible]	[illegible]	[illegible]	[illegible]	[illegible]	[illegible]	[illegible]	[illegible]	[illegible]	[illegible]	[illegible]	[illegible]	[illegible]	[illegible]	[illegible]	[illegible]	[illegible]
		Gabor_S8Fp5A90_glszm_LargeAreaHighGrayLevelEmphasis	TRUE	[illegible]	[illegible]	[illegible]	[illegible]	[illegible]	[illegible]	[illegible]	[illegible]	[illegible]	[illegible]	[illegible]	[illegible]	[illegible]	[illegible]	[illegible]	[illegible]	[illegible]	[illegible]
		Gabor_S8Fp5A90_glszm_LargeAreaLowGrayLevelEmphasis	FALSE	[illegible]	[illegible]	[illegible]	[illegible]	[illegible]	[illegible]	[illegible]	[illegible]	[illegible]	[illegible]	[illegible]	[illegible]	[illegible]	[illegible]	[illegible]	[illegible]	[illegible]	[illegible]
		Gabor_S8Fp5A90_glszm_LowGrayLevelZoneEmphasis	FALSE	[illegible]	[illegible]	[illegible]	[illegible]	[illegible]	[illegible]	[illegible]	[illegible]	[illegible]	[illegible]	[illegible]	[illegible]	[illegible]	[illegible]	[illegible]	[illegible]	[illegible]	[illegible]
		Gabor_S8Fp5A90_glszm_SizeZoneNonUniformity	FALSE	[illegible]	[illegible]	[illegible]	[illegible]	[illegible]	[illegible]	[illegible]	[illegible]	[illegible]	[illegible]	[illegible]	[illegible]	[illegible]	[illegible]	[illegible]	[illegible]	[illegible]	[illegible]
		Gabor_S8Fp5A90_glszm_SizeZoneNonUniformityNormalized	FALSE	[illegible]	[illegible]	[illegible]	[illegible]	[illegible]	[illegible]	[illegible]	[illegible]	[illegible]	[illegible]	[illegible]	[illegible]	[illegible]	[illegible]	[illegible]	[illegible]	[illegible]	[illegible]
		Gabor_S8Fp5A90_glszm_SmallAreaEmphasis	FALSE	[illegible]	[illegible]	[illegible]	[illegible]	[illegible]	[illegible]	[illegible]	[illegible]	[illegible]	[illegible]	[illegible]	[illegible]	[illegible]	[illegible]	[illegible]	[illegible]	[illegible]	[illegible]
		Gabor_S8Fp5A90_glszm_SmallAreaHighGrayLevelEmphasis	TRUE	[illegible]	[illegible]	[illegible]	[illegible]	[illegible]	[illegible]	[illegible]	[illegible]	[illegible]	[illegible]	[illegible]	[illegible]	[illegible]	[illegible]	[illegible]	[illegible]	[illegible]	[illegible]
		Gabor_S8Fp5A90_glszm_SmallAreaLowGrayLevelEmphasis	FALSE	[illegible]	[illegible]	[illegible]	[illegible]	[illegible]	[illegible]	[illegible]	[illegible]	[illegible]	[illegible]	[illegible]	[illegible]	[illegible]	[illegible]	[illegible]	[illegible]	[illegible]	[illegible]
		Gabor_S8Fp5A90_glszm_ZoneEntropy	FALSE	[illegible]	[illegible]	[illegible]	[illegible]	[illegible]	[illegible]	[illegible]	[illegible]	[illegible]	[illegible]	[illegible]	[illegible]	[illegible]	[illegible]	[illegible]	[illegible]	[illegible]	[illegible]
		Gabor_S8Fp5A90_glszm_ZonePercentage	FALSE	[illegible]	[illegible]	[illegible]	[illegible]	[illegible]	[illegible]	[illegible]	[illegible]	[illegible]	[illegible]	[illegible]	[illegible]	[illegible]	[illegible]	[illegible]	[illegible]	[illegible]	[illegible]
		Gabor_S8Fp5A90_glszm_ZoneVariance	FALSE	[illegible]	[illegible]	[illegible]	[illegible]	[illegible]	[illegible]	[illegible]	[illegible]	[illegible]	[illegible]	[illegible]	[illegible]	[illegible]	[illegible]	[illegible]	[illegible]	[illegible]	[illegible]
	NGTDM	Gabor_S8Fp5A90_ngtdm_Busyness	TRUE	[illegible]	[illegible]	[illegible]	[illegible]	[illegible]	[illegible]	[illegible]	[illegible]	[illegible]	[illegible]	[illegible]	[illegible]	[illegible]	[illegible]	[illegible]	[illegible]	[illegible]	[illegible]
		Gabor_S8Fp5A90_ngtdm_Coarseness	FALSE	[illegible]	[illegible]	[illegible]	[illegible]	[illegible]	[illegible]	[illegible]	[illegible]	[illegible]	[illegible]	[illegible]	[illegible]	[illegible]	[illegible]	[illegible]	[illegible]	[illegible]	[illegible]
		Gabor_S8Fp5A90_ngtdm_Complexity	FALSE	[illegible]	[illegible]	[illegible]	[illegible]	[illegible]	[illegible]	[illegible]	[illegible]	[illegible]	[illegible]	[illegible]	[illegible]	[illegible]	[illegible]	[illegible]	[illegible]	[illegible]	[illegible]
		Gabor_S8Fp5A90_ngtdm_Contrast	FALSE	[illegible]	[illegible]	[illegible]	[illegible]	[illegible]	[illegible]	[illegible]	[illegible]	[illegible]	[illegible]	[illegible]	[illegible]	[illegible]	[illegible]	[illegible]	[illegible]	[illegible]	[illegible]
		Gabor_S8Fp5A90_ngtdm_Strength	TRUE	[illegible]	[illegible]	[illegible]	[illegible]	[illegible]	[illegible]	[illegible]	[illegible]	[illegible]	[illegible]	[illegible]	[illegible]	[illegible]	[illegible]	[illegible]	[illegible]	[illegible]	[illegible]
Gabor - kernel size 8 - frequency = 0.5 - orientation 3π/4	First Order	Gabor_S8Fp5A135_firstorder_10Percentile	FALSE	[illegible]	[illegible]	[illegible]	[illegible]	[illegible]	[illegible]	[illegible]	[illegible]	[illegible]	[illegible]	[illegible]	[illegible]	[illegible]	[illegible]	[illegible]	[illegible]	[illegible]	[illegible]
		Gabor_S8Fp5A135_firstorder_90Percentile	FALSE	[illegible]	[illegible]	[illegible]	[illegible]	[illegible]	[illegible]	[illegible]	[illegible]	[illegible]	[illegible]	[illegible]	[illegible]	[illegible]	[illegible]	[illegible]	[illegible]	[illegible]	[illegible]
		Gabor_S8Fp5A135_firstorder_Energy	FALSE	[illegible]	[illegible]	[illegible]	[illegible]	[illegible]	[illegible]	[illegible]	[illegible]	[illegible]	[illegible]	[illegible]	[illegible]	[illegible]	[illegible]	[illegible]	[illegible]	[illegible]	[illegible]
		Gabor_S8Fp5A135_firstorder_Entropy	FALSE	[illegible]	[illegible]	[illegible]	[illegible]	[illegible]	[illegible]	[illegible]	[illegible]	[illegible]	[illegible]	[illegible]	[illegible]	[illegible]	[illegible]	[illegible]	[illegible]	[illegible]	[illegible]
		Gabor_S8Fp5A135_firstorder_InterquartileRange	FALSE	[illegible]	[illegible]	[illegible]	[illegible]	[illegible]	[illegible]	[illegible]	[illegible]	[illegible]	[illegible]	[illegible]	[illegible]	[illegible]	[illegible]	[illegible]	[illegible]	[illegible]	[illegible]
		Gabor_S8Fp5A135_firstorder_Kurtosis	TRUE	[illegible]	[illegible]	[illegible]	[illegible]	[illegible]	[illegible]	[illegible]	[illegible]	[illegible]	[illegible]	[illegible]	[illegible]	[illegible]	[illegible]	[illegible]	[illegible]	[illegible]	[illegible]
		Gabor_S8Fp5A135_firstorder_Maximum	FALSE	[illegible]	[illegible]	[illegible]	[illegible]	[illegible]	[illegible]	[illegible]	[illegible]	[illegible]	[illegible]	[illegible]	[illegible]	[illegible]	[illegible]	[illegible]	[illegible]	[illegible]	[illegible]
		Gabor_S8Fp5A135_firstorder_MeanAbsoluteDeviation	FALSE	[illegible]	[illegible]	[illegible]	[illegible]	[illegible]	[illegible]	[illegible]	[illegible]	[illegible]	[illegible]	[illegible]	[illegible]	[illegible]	[illegible]	[illegible]	[illegible]	[illegible]	[illegible]
		Gabor_S8Fp5A135_firstorder_Mean	FALSE	[illegible]	[illegible]	[illegible]	[illegible]	[illegible]	[illegible]	[illegible]	[illegible]	[illegible]	[illegible]	[illegible]	[illegible]	[illegible]	[illegible]	[illegible]	[illegible]	[illegible]	[illegible]
		Gabor_S8Fp5A135_firstorder_Median	FALSE	[illegible]	[illegible]	[illegible]	[illegible]	[illegible]	[illegible]	[illegible]	[illegible]	[illegible]	[illegible]	[illegible]	[illegible]	[illegible]	[illegible]	[illegible]	[illegible]	[illegible]	[illegible]
		Gabor_S8Fp5A135_firstorder_Minimum	FALSE	[illegible]	[illegible]	[illegible]	[illegible]	[illegible]	[illegible]	[illegible]	[illegible]	[illegible]	[illegible]	[illegible]	[illegible]	[illegible]	[illegible]	[illegible]	[illegible]	[illegible]	[illegible]
		Gabor_S8Fp5A135_firstorder_Range	FALSE	[illegible]	[illegible]	[illegible]	[illegible]	[illegible]	[illegible]	[illegible]	[illegible]	[illegible]	[illegible]	[illegible]	[illegible]	[illegible]	[illegible]	[illegible]	[illegible]	[illegible]	[illegible]
		Gabor_S8Fp5A135_firstorder_RobustMeanAbsoluteDeviation	FALSE	[illegible]	[illegible]	[illegible]	[illegible]	[illegible]	[illegible]	[illegible]	[illegible]	[illegible]	[illegible]	[illegible]	[illegible]	[illegible]	[illegible]	[illegible]	[illegible]	[illegible]	[illegible]
		Gabor_S8Fp5A135_firstorder_RootMeanSquared	FALSE	[illegible]	[illegible]	[illegible]	[illegible]	[illegible]	[illegible]	[illegible]	[illegible]	[illegible]	[illegible]	[illegible]	[illegible]	[illegible]	[illegible]	[illegible]	[illegible]	[illegible]	[illegible]
		Gabor_S8Fp5A135_firstorder_Skewness	FALSE	[illegible]	[illegible]	[illegible]	[illegible]	[illegible]	[illegible]	[illegible]	[illegible]	[illegible]	[illegible]	[illegible]	[illegible]	[illegible]	[illegible]	[illegible]	[illegible]	[illegible]	[illegible]
		Gabor_S8Fp5A135_firstorder_TotalEnergy	FALSE	[illegible]	[illegible]	[illegible]	[illegible]	[illegible]	[illegible]	[illegible]	[illegible]	[illegible]	[illegible]	[illegible]	[illegible]	[illegible]	[illegible]	[illegible]	[illegible]	[illegible]	[illegible]
		Gabor_S8Fp5A135_firstorder_Uniformity	FALSE	[illegible]	[illegible]	[illegible]	[illegible]	[illegible]	[illegible]	[illegible]	[illegible]	[illegible]	[illegible]	[illegible]	[illegible]	[illegible]	[illegible]	[illegible]	[illegible]	[illegible]	[illegible]
		Gabor_S8Fp5A135_firstorder_Variance	FALSE	[illegible]	[illegible]	[illegible]	[illegible]	[illegible]	[illegible]	[illegible]	[illegible]	[illegible]	[illegible]	[illegible]	[illegible]	[illegible]	[illegible]	[illegible]	[illegible]	[illegible]	[illegible]
	GLCM	Gabor_S8Fp5A135_glcm_Autocorrelation	FALSE	[illegible]	[illegible]	[illegible]	[illegible]	[illegible]	[illegible]	[illegible]	[illegible]	[illegible]	[illegible]	[illegible]	[illegible]	[illegible]	[illegible]	[illegible]	[illegible]	[illegible]	[illegible]
		Gabor_S8Fp5A135_glcm_ClusterProminence	TRUE	[illegible]	[illegible]	[illegible]	[illegible]	[illegible]	[illegible]	[illegible]	[illegible]	[illegible]	[illegible]	[illegible]	[illegible]	[illegible]	[illegible]	[illegible]	[illegible]	[illegible]	[illegible]
		Gabor_S8Fp5A135_glcm_ClusterShade	TRUE	[illegible]	[illegible]	[illegible]	[illegible]	[illegible]	[illegible]	[illegible]	[illegible]	[illegible]	[illegible]	[illegible]	[illegible]	[illegible]	[illegible]	[illegible]	[illegible]	[illegible]	[illegible]
		Gabor_S8Fp5A135_glcm_ClusterTendency	FALSE	[illegible]	[illegible]	[illegible]	[illegible]	[illegible]	[illegible]	[illegible]	[illegible]	[illegible]	[illegible]	[illegible]	[illegible]	[illegible]	[illegible]	[illegible]	[illegible]	[illegible]	[illegible]
		Gabor_S8Fp5A135_glcm_Contrast	FALSE	[illegible]	[illegible]	[illegible]	[illegible]	[illegible]	[illegible]	[illegible]	[illegible]	[illegible]	[illegible]	[illegible]	[illegible]	[illegible]	[illegible]	[illegible]	[illegible]	[illegible]	[illegible]
		Gabor_S8Fp5A135_glcm_Correlation	FALSE	[illegible]	[illegible]	[illegible]	[illegible]	[illegible]	[illegible]	[illegible]	[illegible]	[illegible]	[illegible]	[illegible]	[illegible]	[illegible]	[illegible]	[illegible]	[illegible]	[illegible]	[illegible]
		Gabor_S8Fp5A135_glcm_DifferenceAverage	FALSE	[illegible]	[illegible]	[illegible]	[illegible]	[illegible]	[illegible]	[illegible]	[illegible]	[illegible]	[illegible]	[illegible]	[illegible]	[illegible]	[illegible]	[illegible]	[illegible]	[illegible]	[illegible]
		Gabor_S8Fp5A135_glcm_DifferenceEntropy	FALSE	[illegible]	[illegible]	[illegible]	[illegible]	[illegible]	[illegible]	[illegible]	[illegible]	[illegible]	[illegible]	[illegible]	[illegible]	[illegible]	[illegible]	[illegible]	[illegible]	[illegible]	[illegible]
		Gabor_S8Fp5A135_glcm_DifferenceVariance	FALSE	[illegible]	[illegible]	[illegible]	[illegible]	[illegible]	[illegible]	[illegible]	[illegible]	[illegible]	[illegible]	[illegible]	[illegible]	[illegible]	[illegible]	[illegible]	[illegible]	[illegible]	[illegible]
		Gabor_S8Fp5A135_glcm_Id	FALSE	[illegible]	[illegible]	[illegible]	[illegible]	[illegible]	[illegible]	[illegible]	[illegible]	[illegible]	[illegible]	[illegible]	[illegible]	[illegible]	[illegible]	[illegible]	[illegible]	[illegible]	[illegible]
		Gabor_S8Fp5A135_glcm_Idm	FALSE	[illegible]	[illegible]	[illegible]	[illegible]	[illegible]	[illegible]	[illegible]	[illegible]	[illegible]	[illegible]	[illegible]	[illegible]	[illegible]	[illegible]	[illegible]	[illegible]	[illegible]	[illegible]
		Gabor_S8Fp5A135_glcm_Idmn	FALSE	[illegible]	[illegible]	[illegible]	[illegible]	[illegible]	[illegible]	[illegible]	[illegible]	[illegible]	[illegible]	[illegible]	[illegible]	[illegible]	[illegible]	[illegible]	[illegible]	[illegible]	[illegible]
		Gabor_S8Fp5A135_glcm_Idn	FALSE	[illegible]	[illegible]	[illegible]	[illegible]	[illegible]	[illegible]	[illegible]	[illegible]	[illegible]	[illegible]	[illegible]	[illegible]	[illegible]	[illegible]	[illegible]	[illegible]	[illegible]	[illegible]
		Gabor_S8Fp5A135_glcm_Imc1	FALSE	[illegible]	[illegible]	[illegible]	[illegible]	[illegible]	[illegible]	[illegible]	[illegible]	[illegible]	[illegible]	[illegible]	[illegible]	[illegible]	[illegible]	[illegible]	[illegible]	[illegible]	[illegible]
		Gabor_S8Fp5A135_glcm_Imc2	FALSE	[illegible]	[illegible]	[illegible]	[illegible]	[illegible]	[illegible]	[illegible]	[illegible]	[illegible]	[illegible]	[illegible]	[illegible]	[illegible]	[illegible]	[illegible]	[illegible]	[illegible]	[illegible]
		Gabor_S8Fp5A135_glcm_InverseVariance	FALSE	[illegible]	[illegible]	[illegible]	[illegible]	[illegible]	[illegible]	[illegible]	[illegible]	[illegible]	[illegible]	[illegible]	[illegible]	[illegible]	[illegible]	[illegible]	[illegible]	[illegible]	[illegible]
		Gabor_S8Fp5A135_glcm_JointAverage	FALSE	[illegible]	[illegible]	[illegible]	[illegible]	[illegible]	[illegible]	[illegible]	[illegible]	[illegible]	[illegible]	[illegible]	[illegible]	[illegible]	[illegible]	[illegible]	[illegible]	[illegible]	[illegible]
		Gabor_S8Fp5A135_glcm_JointEnergy	FALSE	[illegible]	[illegible]	[illegible]	[illegible]	[illegible]	[illegible]	[illegible]	[illegible]	[illegible]	[illegible]	[illegible]	[illegible]	[illegible]	[illegible]	[illegible]	[illegible]	[illegible]	[illegible]
		Gabor_S8Fp5A135_glcm_JointEntropy	FALSE	[illegible]	[illegible]	[illegible]	[illegible]	[illegible]	[illegible]	[illegible]	[illegible]	[illegible]	[illegible]	[illegible]	[illegible]	[illegible]	[illegible]	[illegible]	[illegible]	[illegible]	[illegible]
		Gabor_S8Fp5A135_glcm_MCC	FALSE	[illegible]	[illegible]	[illegible]	[illegible]	[illegible]	[illegible]	[illegible]	[illegible]	[illegible]	[illegible]	[illegible]	[illegible]	[illegible]	[illegible]	[illegible]	[illegible]	[illegible]	[illegible]
		Gabor_S8Fp5A135_glcm_MaximumProbability	FALSE	[illegible]	[illegible]	[illegible]	[illegible]	[illegible]	[illegible]	[illegible]	[illegible]	[illegible]	[illegible]	[illegible]	[illegible]	[illegible]	[illegible]	[illegible]	[illegible]	[illegible]	[illegible]
		Gabor_S8Fp5A135_glcm_SumAverage	FALSE	[illegible]	[illegible]	[illegible]	[illegible]	[illegible]	[illegible]	[illegible]	[illegible]	[illegible]	[illegible]	[illegible]	[illegible]	[illegible]	[illegible]	[illegible]	[illegible]	[illegible]	[illegible]
		Gabor_S8Fp5A135_glcm_SumEntropy	FALSE	[illegible]	[illegible]	[illegible]	[illegible]	[illegible]	[illegible]	[illegible]	[illegible]	[illegible]	[illegible]	[illegible]	[illegible]	[illegible]	[illegible]	[illegible]	[illegible]	[illegible]	[illegible]
		Gabor_S8Fp5A135_glcm_SumSquares	FALSE	[illegible]	[illegible]	[illegible]	[illegible]	[illegible]	[illegible]	[illegible]	[illegible]	[illegible]	[illegible]	[illegible]	[illegible]	[illegible]	[illegible]	[illegible]	[illegible]	[illegible]	[illegible]
	GLDM	Gabor_S8Fp5A135_gldm_DependenceEntropy	FALSE	[illegible]	[illegible]	[illegible]	[illegible]	[illegible]	[illegible]	[illegible]	[illegible]	[illegible]	[illegible]	[illegible]	[illegible]	[illegible]	[illegible]	[illegible]	[illegible]	[illegible]	[illegible]
		Gabor_S8Fp5A135_gldm_DependenceNonUniformity	TRUE	[illegible]	[illegible]	[illegible]	[illegible]	[illegible]	[illegible]	[illegible]	[illegible]	[illegible]	[illegible]	[illegible]	[illegible]	[illegible]	[illegible]	[illegible]	[illegible]	[illegible]	[illegible]
		Gabor_S8Fp5A135_gldm_DependenceNonUniformityNormalized	FALSE	[illegible]	[illegible]	[illegible]	[illegible]	[illegible]	[illegible]	[illegible]	[illegible]	[illegible]	[illegible]	[illegible]	[illegible]	[illegible]	[illegible]	[illegible]	[illegible]	[illegible]	[illegible]
		Gabor_S8Fp5A135_gldm_DependenceVariance	FALSE	[illegible]	[illegible]	[illegible]	[illegible]	[illegible]	[illegible]	[illegible]	[illegible]	[illegible]	[illegible]	[illegible]	[illegible]	[illegible]	[illegible]	[illegible]	[illegible]	[illegible]	[illegible]
		Gabor_S8Fp5A135_gldm_GrayLevelNonUniformity	FALSE	[illegible]	[illegible]	[illegible]	[illegible]	[illegible]	[illegible]	[illegible]	[illegible]	[illegible]	[illegible]	[illegible]	[illegible]	[illegible]	[illegible]	[illegible]	[illegible]	[illegible]	[illegible]
		Gabor_S8Fp5A135_gldm_GrayLevelVariance	FALSE	[illegible]	[illegible]	[illegible]	[illegible]	[illegible]	[illegible]	[illegible]	[illegible]	[illegible]	[illegible]	[illegible]	[illegible]	[illegible]	[illegible]	[illegible]	[illegible]	[illegible]	[illegible]
		Gabor_S8Fp5A135_gldm_HighGrayLevelEmphasis	FALSE	[illegible]	[illegible]	[illegible]	[illegible]	[illegible]	[illegible]	[illegible]	[illegible]	[illegible]	[illegible]	[illegible]	[illegible]	[illegible]	[illegible]	[illegible]	[illegible]	[illegible]	[illegible]
		Gabor_S8Fp5A135_gldm_LargeDependenceEmphasis	FALSE	[illegible]	[illegible]	[illegible]	[illegible]	[illegible]	[illegible]	[illegible]	[illegible]	[illegible]	[illegible]	[illegible]	[illegible]	[illegible]	[illegible]	[illegible]	[illegible]	[illegible]	[illegible]
		Gabor_S8Fp5A135_gldm_LargeDependenceHighGrayLevelEmphasis	TRUE	[illegible]	[illegible]	[illegible]	[illegible]	[illegible]	[illegible]	[illegible]	[illegible]	[illegible]	[illegible]	[illegible]	[illegible]	[illegible]	[illegible]	[illegible]	[illegible]	[illegible]	[illegible]
		Gabor_S8Fp5A135_gldm_LargeDependenceLowGrayLevelEmphasis	FALSE	[illegible]	[illegible]	[illegible]	[illegible]	[illegible]	[illegible]	[illegible]	[illegible]	[illegible]	[illegible]	[illegible]	[illegible]	[illegible]	[illegible]	[illegible]	[illegible]	[illegible]	[illegible]
		Gabor_S8Fp5A135_gldm_LowGrayLevelEmphasis	FALSE	[illegible]	[illegible]	[illegible]	[illegible]	[illegible]	[illegible]	[illegible]	[illegible]	[illegible]	[illegible]	[illegible]	[illegible]	[illegible]	[illegible]	[illegible]	[illegible]	[illegible]	[illegible]
		Gabor_S8Fp5A135_gldm_SmallDependenceEmphasis	FALSE	[illegible]	[illegible]	[illegible]	[illegible]	[illegible]	[illegible]	[illegible]	[illegible]	[illegible]	[illegible]	[illegible]	[illegible]	[illegible]	[illegible]	[illegible]	[illegible]	[illegible]	[illegible]
		Gabor_S8Fp5A135_gldm_SmallDependenceHighGrayLevelEmphasis	FALSE	[illegible]	[illegible]	[illegible]	[illegible]	[illegible]	[illegible]	[illegible]	[illegible]	[illegible]	[illegible]	[illegible]	[illegible]	[illegible]	[illegible]	[illegible]	[illegible]	[illegible]	[illegible]
		Gabor_S8Fp5A135_gldm_SmallDependenceLowGrayLevelEmphasis	FALSE	[illegible]	[illegible]	[illegible]	[illegible]	[illegible]	[illegible]	[illegible]	[illegible]	[illegible]	[illegible]	[illegible]	[illegible]	[illegible]	[illegible]	[illegible]	[illegible]	[illegible]	[illegible]
	GLRLM	Gabor_S8Fp5A135_glrlm_GrayLevelNonUniformity	FALSE	[illegible]	[illegible]	[illegible]	[illegible]	[illegible]	[illegible]	[illegible]	[illegible]	[illegible]	[illegible]	[illegible]	[illegible]	[illegible]	[illegible]	[illegible]	[illegible]	[illegible]	[illegible]
		Gabor_S8Fp5A135_glrlm_GrayLevelNonUniformityNormalized	FALSE	[illegible]	[illegible]	[illegible]	[illegible]	[illegible]	[illegible]	[illegible]	[illegible]	[illegible]	[illegible]	[illegible]	[illegible]	[illegible]	[illegible]	[illegible]	[illegible]	[illegible]	[illegible]
		Gabor_S8Fp5A135_glrlm_GrayLevelVariance	FALSE	[illegible]	[illegible]	[illegible]	[illegible]	[illegible]	[illegible]	[illegible]	[illegible]	[illegible]	[illegible]	[illegible]	[illegible]	[illegible]	[illegible]	[illegible]	[illegible]	[illegible]	[illegible]
		Gabor_S8Fp5A135_glrlm_HighGrayLevelRunEmphasis	FALSE	[illegible]	[illegible]	[illegible]	[illegible]	[illegible]	[illegible]	[illegible]	[illegible]	[illegible]	[illegible]	[illegible]	[illegible]	[illegible]	[illegible]	[illegible]	[illegible]	[illegible]	[illegible]
		Gabor_S8Fp5A135_glrlm_LongRunEmphasis	FALSE	[illegible]	[illegible]	[illegible]	[illegible]	[illegible]	[illegible]	[illegible]	[illegible]	[illegible]	[illegible]	[illegible]	[illegible]	[illegible]	[illegible]	[illegible]	[illegible]	[illegible]	[illegible]
		Gabor_S8Fp5A135_glrlm_LongRunHighGrayLevelEmphasis	FALSE	[illegible]	[illegible]	[illegible]	[illegible]	[illegible]	[illegible]	[illegible]	[illegible]	[illegible]	[illegible]	[illegible]	[illegible]	[illegible]	[illegible]	[illegible]	[illegible]	[illegible]	[illegible]
		Gabor_S8Fp5A135_glrlm_LongRunLowGrayLevelEmphasis	FALSE	[illegible]	[illegible]	[illegible]	[illegible]	[illegible]	[illegible]	[illegible]	[illegible]	[illegible]	[illegible]	[illegible]	[illegible]	[illegible]	[illegible]	[illegible]	[illegible]	[illegible]	[illegible]
		Gabor_S8Fp5A135_glrlm_LowGrayLevelRunEmphasis	FALSE	[illegible]	[illegible]	[illegible]	[illegible]	[illegible]	[illegible]	[illegible]	[illegible]	[illegible]	[illegible]	[illegible]	[illegible]	[illegible]	[illegible]	[illegible]	[illegible]	[illegible]	[illegible]
		Gabor_S8Fp5A135_glrlm_RunEntropy	FALSE	[illegible]	[illegible]	[illegible]	[illegible]	[illegible]	[illegible]	[illegible]	[illegible]	[illegible]	[illegible]	[illegible]	[illegible]	[illegible]	[illegible]	[illegible]	[illegible]	[illegible]	[illegible]
		Gabor_S8Fp5A135_glrlm_RunLengthNonUniformity	TRUE	[illegible]	[illegible]	[illegible]	[illegible]	[illegible]	[illegible]	[illegible]	[illegible]	[illegible]	[illegible]	[illegible]	[illegible]	[illegible]	[illegible]	[illegible]	[illegible]	[illegible]	[illegible]
		Gabor_S8Fp5A135_glrlm_RunLengthNonUniformityNormalized	FALSE	[illegible]	[illegible]	[illegible]	[illegible]	[illegible]	[illegible]	[illegible]	[illegible]	[illegible]	[illegible]	[illegible]	[illegible]	[illegible]	[illegible]	[illegible]	[illegible]	[illegible]	[illegible]
		Gabor_S8Fp5A135_glrlm_RunPercentage	FALSE	[illegible]	[illegible]	[illegible]	[illegible]	[illegible]	[illegible]	[illegible]	[illegible]	[illegible]	[illegible]	[illegible]	[illegible]	[illegible]	[illegible]	[illegible]	[illegible]	[illegible]	[illegible]
		Gabor_S8Fp5A135_glrlm_RunVariance	FALSE	[illegible]	[illegible]	[illegible]	[illegible]	[illegible]	[illegible]	[illegible]	[illegible]	[illegible]	[illegible]	[illegible]	[illegible]	[illegible]	[illegible]	[illegible]	[illegible]	[illegible]	[illegible]
		Gabor_S8Fp5A135_glrlm_ShortRunEmphasis	FALSE	[illegible]	[illegible]	[illegible]	[illegible]	[illegible]	[illegible]	[illegible]	[illegible]	[illegible]	[illegible]	[illegible]	[illegible]	[illegible]	[illegible]	[illegible]	[illegible]	[illegible]	[illegible]
		Gabor_S8Fp5A135_glrlm_ShortRunHighGrayLevelEmphasis	FALSE	[illegible]	[illegible]	[illegible]	[illegible]	[illegible]	[illegible]	[illegible]	[illegible]	[illegible]	[illegible]	[illegible]	[illegible]	[illegible]	[illegible]	[illegible]	[illegible]	[illegible]	[illegible]
		Gabor_S8Fp5A135_glrlm_ShortRunLowGrayLevelEmphasis	FALSE	[illegible]	[illegible]	[illegible]	[illegible]	[illegible]	[illegible]	[illegible]	[illegible]	[illegible]	[illegible]	[illegible]	[illegible]	[illegible]	[illegible]	[illegible]	[illegible]	[illegible]	[illegible]
	GLSZM	Gabor_S8Fp5A135_glszm_GrayLevelNonUniformity	TRUE	[illegible]	[illegible]	[illegible]	[illegible]	[illegible]	[illegible]	[illegible]	[illegible]	[illegible]	[illegible]	[illegible]	[illegible]	[illegible]	[illegible]	[illegible]	[illegible]	[illegible]	[illegible]
		Gabor_S8Fp5A135_glszm_GrayLevelNonUniformityNormalized	FALSE	[illegible]	[illegible]	[illegible]	[illegible]	[illegible]	[illegible]	[illegible]	[illegible]	[illegible]	[illegible]	[illegible]	[illegible]	[illegible]	[illegible]	[illegible]	[illegible]	[illegible]	[illegible]
		Gabor_S8Fp5A135_glszm_GrayLevelVariance	FALSE	[illegible]	[illegible]	[illegible]	[illegible]	[illegible]	[illegible]	[illegible]	[illegible]	[illegible]	[illegible]	[illegible]	[illegible]	[illegible]	[illegible]	[illegible]	[illegible]	[illegible]	[illegible]
		Gabor_S8Fp5A135_glszm_HighGrayLevelZoneEmphasis	FALSE	[illegible]	[illegible]	[illegible]	[illegible]	[illegible]	[illegible]	[illegible]	[illegible]	[illegible]	[illegible]	[illegible]	[illegible]	[illegible]	[illegible]	[illegible]	[illegible]	[illegible]	[illegible]
		Gabor_S8Fp5A135_glszm_LargeAreaEmphasis	TRUE	[illegible]	[illegible]	[illegible]	[illegible]	[illegible]	[illegible]	[illegible]	[illegible]	[illegible]	[illegible]	[illegible]	[illegible]	[illegible]	[illegible]	[illegible]	[illegible]	[illegible]	[illegible]
		Gabor_S8Fp5A135_glszm_LargeAreaHighGrayLevelEmphasis	TRUE	[illegible]	[illegible]	[illegible]	[illegible]	[illegible]	[illegible]	[illegible]	[illegible]	[illegible]	[illegible]	[illegible]	[illegible]	[illegible]	[illegible]	[illegible]	[illegible]	[illegible]	[illegible]
		Gabor_S8Fp5A135_glszm_LargeAreaLowGrayLevelEmphasis	FALSE	[illegible]	[illegible]	[illegible]	[illegible]	[illegible]	[illegible]	[illegible]	[illegible]	[illegible]	[illegible]	[illegible]	[illegible]	[illegible]	[illegible]	[illegible]	[illegible]	[illegible]	[illegible]
		Gabor_S8Fp5A135_glszm_LowGrayLevelZoneEmphasis	FALSE	[illegible]	[illegible]	[illegible]	[illegible]	[illegible]	[illegible]	[illegible]	[illegible]	[illegible]	[illegible]	[illegible]	[illegible]	[illegible]	[illegible]	[illegible]	[illegible]	[illegible]	[illegible]
		Gabor_S8Fp5A135_glszm_SizeZoneNonUniformity	FALSE	[illegible]	[illegible]	[illegible]	[illegible]	[illegible]	[illegible]	[illegible]	[illegible]	[illegible]	[illegible]	[illegible]	[illegible]	[illegible]	[illegible]	[illegible]	[illegible]	[illegible]	[illegible]
		Gabor_S8Fp5A135_glszm_SizeZoneNonUniformityNormalized	FALSE	[illegible]	[illegible]	[illegible]	[illegible]	[illegible]	[illegible]	[illegible]	[illegible]	[illegible]	[illegible]	[illegible]	[illegible]	[illegible]	[illegible]	[illegible]	[illegible]	[illegible]	[illegible]
		Gabor_S8Fp5A135_glszm_SmallAreaEmphasis	FALSE	[illegible]	[illegible]	[illegible]	[illegible]	[illegible]	[illegible]	[illegible]	[illegible]	[illegible]	[illegible]	[illegible]	[illegible]	[illegible]	[illegible]	[illegible]	[illegible]	[illegible]	[illegible]
		Gabor_S8Fp5A135_glszm_SmallAreaHighGrayLevelEmphasis	FALSE	[illegible]	[illegible]	[illegible]	[illegible]	[illegible]	[illegible]	[illegible]	[illegible]	[illegible]	[illegible]	[illegible]	[illegible]	[illegible]	[illegible]	[illegible]	[illegible]	[illegible]	[illegible]
		Gabor_S8Fp5A135_glszm_SmallAreaLowGrayLevelEmphasis	FALSE	[illegible]	[illegible]	[illegible]	[illegible]	[illegible]	[illegible]	[illegible]	[illegible]	[illegible]	[illegible]	[illegible]	[illegible]	[illegible]	[illegible]	[illegible]	[illegible]	[illegible]	[illegible]
		Gabor_S8Fp5A135_glszm_ZoneEntropy	FALSE	[illegible]	[illegible]	[illegible]	[illegible]	[illegible]	[illegible]	[illegible]	[illegible]	[illegible]	[illegible]	[illegible]	[illegible]	[illegible]	[illegible]	[illegible]	[illegible]	[illegible]	[illegible]
		Gabor_S8Fp5A135_glszm_ZonePercentage	FALSE	[illegible]	[illegible]	[illegible]	[illegible]	[illegible]	[illegible]	[illegible]	[illegible]	[illegible]	[illegible]	[illegible]	[illegible]	[illegible]	[illegible]	[illegible]	[illegible]	[illegible]	[illegible]
		Gabor_S8Fp5A135_glszm_ZoneVariance	TRUE	[illegible]	[illegible]	[illegible]	[illegible]	[illegible]	[illegible]	[illegible]	[illegible]	[illegible]	[illegible]	[illegible]	[illegible]	[illegible]	[illegible]	[illegible]	[illegible]	[illegible]	[illegible]
	NGTDM	Gabor_S8Fp5A135_ngtdm_Busyness	FALSE	[illegible]	[illegible]	[illegible]	[illegible]	[illegible]	[illegible]	[illegible]	[illegible]	[illegible]	[illegible]	[illegible]	[illegible]	[illegible]	[illegible]	[illegible]	[illegible]	[illegible]	[illegible]
		Gabor_S8Fp5A135_ngtdm_Coarseness	FALSE	[illegible]	[illegible]	[illegible]	[illegible]	[illegible]	[illegible]	[illegible]	[illegible]	[illegible]	[illegible]	[illegible]	[illegible]	[illegible]	[illegible]	[illegible]	[illegible]	[illegible]	[illegible]
		Gabor_S8Fp5A135_ngtdm_Complexity	FALSE	[illegible]	[illegible]	[illegible]	[illegible]	[illegible]	[illegible]	[illegible]	[illegible]	[illegible]	[illegible]	[illegible]	[illegible]	[illegible]	[illegible]	[illegible]	[illegible]	[illegible]	[illegible]
		Gabor_S8Fp5A135_ngtdm_Contrast	FALSE	[illegible]	[illegible]	[illegible]	[illegible]	[illegible]	[illegible]	[illegible]	[illegible]	[illegible]	[illegible]	[illegible]	[illegible]	[illegible]	[illegible]	[illegible]	[illegible]	[illegible]	[illegible]
		Gabor_S8Fp5A135_ngtdm_Strength	TRUE	[illegible]	[illegible]	[illegible]	[illegible]	[illegible]	[illegible]	[illegible]	[illegible]	[illegible]	[illegible]	[illegible]	[illegible]	[illegible]	[illegible]	[illegible]	[illegible]	[illegible]	[illegible]

Supplementary Table S1: Wilcoxon results for each individual radiomics feature. In column D, list of features found to be impacted by the reconstruction kernel after harmonization in at least one of the configurations examined (Tumor - Combat Harmonization - Reference I26f, Pancreas - Combat Harmonization - Reference I26f, Pancreas - Combat Harmonization - Reference B20f, and Tumor - Combat Harmonization - Reference B20f)
