# Supplementary material for: Can CT Image Reconstruction Parameters Impact the Predictive Value of Radiomics Features in Grading Pancreatic Neuroendocrine Neoplasms?
Source: Bioengineering (Basel). 2025 Jan 16;12(1):80. doi: 10.3390/bioengineering12010080 (PMC11763079; doi:10.3390/bioengineering12010080)
Supplement: Supplementary file 1 [file bioengineering-12-00080-s001.zip › Supplementary_Figures_and_Tables/Supplementary_TableS2.pdf]

LASSO	SVM	5-fold CV Accuracies (means ± SD)	Accuracy [95% CI]	Sensitivity [95% CI]	Specificity [95% CI]	Precision [95% CI]	F1 Score [95% CI]
selection using all features	B20f features selected on B20f	0.81 ± 0.03	0.81 [0.75 - 0.89]	0.86 [0.77 - 0.95]	0.78 [0.67 - 0.88]	0.79 [0.69 - 0.89]	0.83 [0.75 - 0.89]
	I26f features selected on B20f	0.72 ± 0.05	0.79 [0.72 - 0.86]	0.84 [0.75 - 0.93]	0.74 [0.63 - 0.85]	0.77 [0.66 - 0.87]	0.80 [0.72 - 087]
	B20f features selected on I26f	0.72 ± 0.04	0.76 [0.68 - 0.84]	0.79 [0.68 - 0.89]	0.72 [0.60 - 0.84]	0.74 [0.63 - 0.85]	0.77 [0.68 - 0.84]
	I26f features selected on I26f	0.77 ± 0.07	0.81 [0.73 - 0.88]	0.91 [0.83 - 0.98]	0.71 [0.59 - 0.82]	0.76 [0.65 - 0.86]	0.83 [0.75 - 0.89]
selection on harmonizable features	B20f features selected on B20f	0.82 ± 0.07	0.84 [0.78 - 0.91]	0.88 [0.79 - 0.95]	0.81 [0.71 - 0.90]	0.82 [0.72 - 0.91]	0.85 [0.77 - 0.91]
	I26f features selected on B20f	0.67 ± 0.04	0.77 [0.69 - 0.84]	0.88 [0.79 - 0.96]	0.66 [0.53 - 077]	0.72 [0.61 - 0.82]	0.79 [0.71 - 0.86]
	B20f features selected on I26f	0.71 ± 0.05	0.78 [0.70 - 0.85]	0.83 [0.73 - 0.92]	0.72 [0.61 - 0.84]	0.75 [0.64 - 0.85]	0.79 [0.70 - 0.86]
	I26f features selected on I26f	0.77 ± 0.09	0.83 [0.76 - 0.90]	0.93 [0.86 - 0098]	0.72 [0.61 - 0.83]	0.77 [0.67-0.87]	0.84 [0.77 - 0.91]

Supplementary Table 2: SVM performance on the training set
