# Supplementary material for: Can CT Image Reconstruction Parameters Impact the Predictive Value of Radiomics Features in Grading Pancreatic Neuroendocrine Neoplasms?
Source: Bioengineering (Basel). 2025 Jan 16;12(1):80. doi: 10.3390/bioengineering12010080 (PMC11763079; doi:10.3390/bioengineering12010080)
Supplement: Supplementary file 1 [file bioengineering-12-00080-s001.zip › Supplementary_Figures_and_Tables/Supplementary_TableS3.pdf]

LASSO	SVM	5-fold CV Accuracies (mean ± SD)	Accuracy [95% CI]	Sensitivity [95% CI]	Specificity [95% CI]	Precision [95% CI]	F1 Score [95% CI]
TRAIN	B20f features selected on B20f	0.79 ± 0.05	0.84 [0.77 - 0.91]	0.88 [0.79 - 0.96]	0.79 [0.69 - 0.89]	0.81 [0.71 - 0.90]	0.84 [0.77 - 0.91]
	I26f features selected on B20f	0.70 ± 0.07	0.76 [0.68 - 0.84]	0.81 [0.70 - 0.91]	0.71 [0.58 - 0.82]	0.73 [0.62 - 0.84]	0.77 [0.68 - 0.85]
	B20f features selected on I26f	0.73 ± 0.10	0.76 [0.68 - 0.84]	0.81 [0.71 - 0.90]	0.71 [0.58 - 0.82]	0.73 [0.62 - 0.84]	0.77 [0.68 - 0.85]
	I26f features selected on I26f	0.70 ± 0.09	0.76 [0.68 - 0.84]	0.90 [0.81 - 0.97]	0.62 [0.49 - 0.75]	0.70 [0.60 - 0.81]	0.79 [0.70 - 0.86]
TEST	B20f features selected on B20f	-	0.64 [0.47 - 0.78]	0.69 [0.42 - 0.93]	0.61 [0.41 - 0.80]	0.50 [0.27 - 0.73]	0.58 [0.33 - 0.77]
	I26f features selected on B20f	-	0.69 [0.53 - 0.83]	0.92 [0.75 - 1.0]	0.57 [0.36 - 0.76]	0.55 [0.33 - 0. 76]	0.69 [0.48 - 0.84]
	B20f features selected on I26f	-	0.78 [0.64 - 0.89]	0.92 [0.74 - 1.0]	0.70 [0.50 - 0.88]	0.63 [0.41 - 0.84]	0.75 [0.55 - 0.90]
	I26f features selected on I26f	-	0.61 [0.44 - 0.78]	0.85 [0.63 - 1.0]	0.49 [0.27 - 0.68]	0.48 [0.28 - 0.68]	0.61 [0.40 - 0.78]

Supplementary Table 3: SVM performance on the training and testing sets for models built using features found harmonizable before accounting for multiple testing correction.
